# From Induced-Fit Assemblies to Ternary Inclusion Complexes with Fullerenes in Corannulene-Based Molecular Tweezers

**DOI:** 10.1021/acs.joc.2c02345

**Published:** 2022-12-01

**Authors:** Adriana Sacristán-Martín, Daniel Miguel, Alberto Diez-Varga, Héctor Barbero, Celedonio M. Álvarez

**Affiliations:** GIR MIOMeT, IU CINQUIMA/Química Inorgánica, Facultad de Ciencias, Universidad de Valladolid, Valladolid E47011, Spain

## Abstract

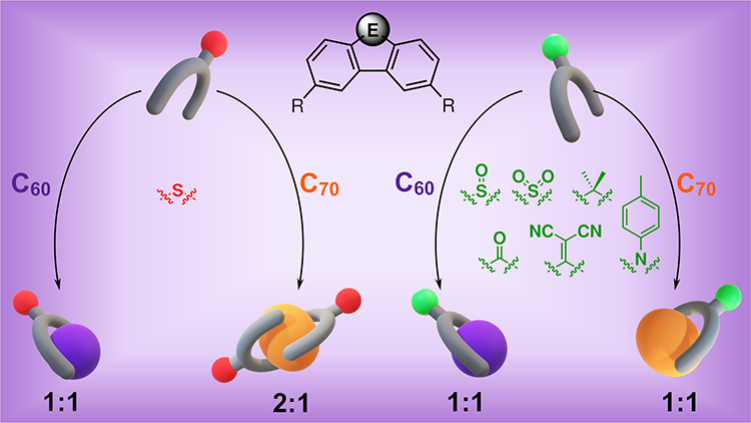

The participation of the tether moiety in fullerene recognition of corannulene-based molecular tweezers is known to be an important factor. In the present work, we describe the synthesis of a set of fullerene receptors bearing two corannulene units located at a suitable distance to effectively interact with C_60_ and C_70_. The tether comprises a fluorene-like scaffold where an assortment of different groups with variable electronic properties has been grafted. The photophysical and electrochemical properties of all final compounds have been unveiled and correlated to the donor/acceptor (DA) nature of the tether. Despite these strong variations, their affinity toward fullerenes cannot be correlated in any way to simple DA behavior as the main contribution to the interaction correspond to London dispersion forces. We found, however, that the sulfur-derived subfamily is able to adapt better to the fullerene outer surface slightly increasing the charge transfer and electrostatic attractive interactions being the most outstanding example the case of thiophene **4-S** with C_70_ as it is capable of forming a ternary 2:1 inclusion complex in solution with an electronic binding energy that offsets entropy and desolvation penalties typically associated with higher-order inclusion complexes.

## Introduction

[5]circulene or, more commonly, corannulene, nowadays constitutes one of the most studied geodesic polyarenes (or buckybowls) due to its manifold properties and applications when appropriately functionalized.^1−3^ With a chemical formula of C_20_H_10_, it is considered as a Buckminsterfullerene fragment and, therefore, it is not surprising to find in the literature a wide variety of designs containing corannulene as the key unit for fullerene recognition. The interaction between fullerenes and other chemical entities gives rise to emergent properties that could be utilized in several applications within the field of material sciences.^4−6^

The host–guest chemistry of buckybowls and fullerenes (or buckyballs) is driven by the well-known concave–convex π–π interactions between the inner face of corannulene and the outer face of the pseudo-spherical fullerene as a result of the nonsymmetrical nature of their p orbitals.^7−9^ It is also known, however, that pristine corannulene does not show appreciable affinity toward fullerene in solution.^7^ Thus, the need for a multivalency approach^10−12^ (i.e., the combination of multiple units of corannulene within the same host to provide a synergistic effect) seems reasonable. The simplest version of such a multivalency consists of a structure with a tweezer-like geometry^13^ where two corannulene motifs are grafted at both ends. This scaffold provides a cavity for an efficient association with the fullerene guest. In this regard, one can find examples such as transition-metal-based complexes,^14,15^ all-organic molecular tweezers,^16−18^ or bistable switchable hosts.^15,19,20^ But perhaps the most recognized family of molecular tweezers are Sygula’s buckycatchers,^21−24^ consisting of a rigid scaffold that maintains two corannulene groups at the right distance with an overall limited conformational mobility. Originally conceived with a cyclooctatetraene tether (Buckycatcher I),^21^ the design continued to improve to maximize the affinity toward fullerenes with a dibenzonorbornadiene spacer (Buckycatcher II)^23^ and dimethylene-bridged Klärner’s tweezers^22^ reaching what the authors called “the affinity limits” due to the high association constants these hosts showed. The tether does not only provide a convenient scaffold to graft both corannulene groups, but it also contributes to the mainly dispersive fullerene attraction. We reached a similar conclusion with our studies on corannulene-based porphyrin clips^25−28^ where up to four buckybowls cooperatively contributed to the overall interaction along with the porphyrin tether. Decoupling such a synergistic effect dramatically reduced the affinity.^27^

**Figure 1:**
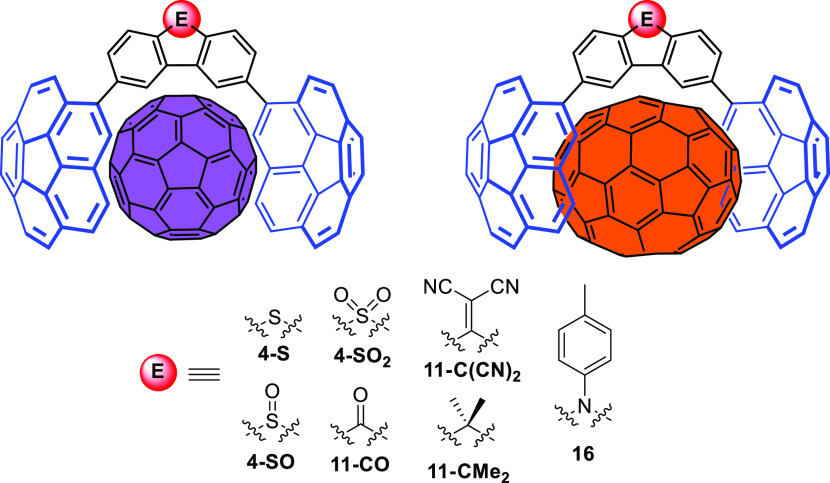
Corannulene-based molecular tweezers with a difunctionalized fluorene-like tether bearing bridgehead groups of a variable electronic nature presented in this work.

We recently reported a self-resetting bistable molecular machine that bears a redox effector whose capability to modify its affinity toward fullerenes can be efficiently modulated through an external stimulus.^20^ We however noted that, under certain conditions (see the Experimental Section), the resulting compound was dibenzothiophene **4-S** (Figure 1), which shows outstanding performance on fullerene recognition (see below). This prompted us to explore the electronic variation of the bridgehead group by synthesizing and studying the assortment of compounds depicted in Figure 1. It is known that the presence of heteroatoms perturbs the electronic properties of the whole carbon/hydrogen system in terms of electrostatics and frontier orbitals energy levels.^29,30^ In fact, corannulene chalcogenides, especially sulfur-derived corannulene compounds,^31−43^ are among the most studied given the possibility of fine-tuning their properties depending on the number of substituents, the location of the heteroatom and its oxidation state. Scott and co-workers pioneered the host–guest chemistry of sulfur-derived corannulenes with fullerenes^44−46^ obtaining moderate binding affinities, but overall outstanding considering there was only one corannulene core instead of a tweezer-like arrangement. According to their results, they concluded that the relative electron-rich orthophenylene substituents definitely contributed to the association enhancement, especially for their fly trap host,^46^ albeit a possible increased fullerene surface coverage could have had a higher impact through attractive dispersion forces.

The nature of π–π interactions is manifold^47−50^ and the importance of each contribution to the interaction energy heavily depends on the electronic nature and the geometrical requirements of the host and guest entities, being the dispersion (London) forces the main factor for relatively large polyaromatic surfaces.^51,52^ Herein, we provide a tweezer model with a fluorene-like tether where two corannulene subunits have been attached to form a well-defined molecular tweezer (Figure 1) that could serve to host fullerenes (C_60_ and C_70_ in this work). A similar motif has demonstrated good performance for a π-extended azabuckybowl.^53^ The tether has been modified so that it contains bridgehead groups with different electronic nature with the aim to test the impact on fullerene recognition by modifying the energy contributions that do not pertain to dispersion forces (charge transfer or electrostatic, mainly).

The distance between the bridgehead group and the corannulene moiety is a phenylene so the through-space effect is expected to be moderate yet trackable. As an example, in a series of sulfur-substituted corannulene, Stuparu observed that one directly attached sulfur atom (internal sulfide) is approximately equivalent to four arylthioethers with a phenylene spacer (terminal sulfide) in terms of electronic acceptance.^33^ Nonetheless, the present model is convenient due to a straightforward synthesis strategy that only uses a common corannulene precursor (see below) and tries to minimize other possible contributions to the supramolecular binding rather than the interaction energy, such as significant entropy contributions (these recognition processes are typically enthalpy-driven),^24,54^ desolvation or deformation energy penalties. Regarding the two latter contributions, none of them are expected a priori to play a major role as the structural variations are exclusively located on the tether, meaning that either their impact is expected to be practically negligible or equal throughout the whole family. For the above reasons, this design then allows us to determine the effect of the bridgehead group and the differential behavior between C_60_ and C_70_ recognition, if any. We provide a variety of motifs usually encountered in optoelectronic materials^55−57^ from strong donors to typical acceptors. From a simplistic point of view, considering exclusively the electronic donor–acceptor properties of hosts and guests and chemical intuition, the best association with fullerenes would be found in *N*-tolyl carbazole **16**, whereas the worst one in ylidenmalononitrile **11-C(CN)**_**2**_.

## Results and Discussion

### Synthesis of Corannulene-Based Hosts

**Scheme 1:**
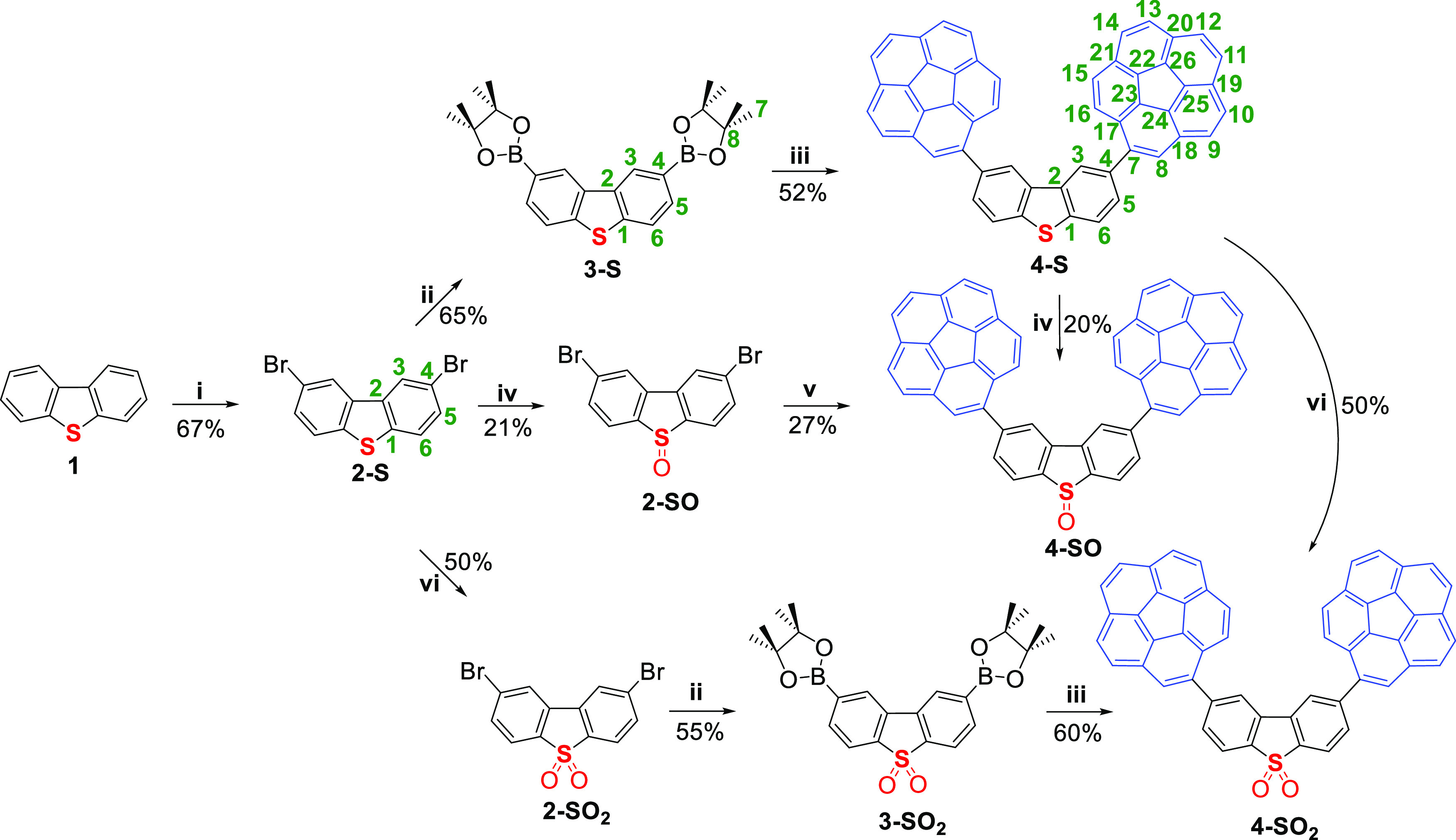
Synthesis of Sulfur-Derived Organic Hosts Containing Corannulene Reagents and conditions: (i) Br_2_, CHCl_3_; (ii) B_2_(pin)_2_, [PdCl_2_(dppf)], KOAc, dioxane, MW, 170 °C; (iii) Br-Cora, [PdCl_2_(dppf)], *^t^*BuONa, toluene, MW, 130 °C; (iv) *m*-CPBA, CH_2_Cl_2_; (v) BpinCora, [PdCl_2_(dppf)], *^t^*BuONa, toluene, MW, 130 °C; (vi) H_2_O_2_, acetic acid, 100 °C. Atom numbering has been altered for consistency purposes throughout the whole family.

As stated above, the synthetic strategy relies on only one corannulene precursor (1-bromocorannulene) and three scaffolds (**1**, **5**, and **12**) to generate all of the members of the family. For S-derived compounds, the sequence starts with the bromination of dibenzothiophene **1** to give compound **2-S** that serves as the common intermediate. From that point, a double Miyaura reaction to provide diboronate **3-S** which is subsequently subjected to a Suzuki C–C cross-coupling allowed us to obtain the final molecular tweezer **4-S** with moderate yield (Scheme 1). We found this strategy to be the most convenient for almost all of the systems reported herein as the alternative (formation of the boronate on the corannulene scaffold and perform the coupling with the dibrominated fluorene-like tether) did not appreciably increase the yield and the Miyaura reaction had to be done onto otherwise precious 1-bromocorannulene.

Access to the fully oxidized version (**4-SO**_**2**_) was granted, again with moderate yields, thanks to the same strategy with an additional oxidation step of intermediate **2-S** to give **2-SO**_**2**_. The case of host **4-SO** is unique within this family since it forced us to use BpinCora as the coupling partner of compound **2-SO**. The usual protocol led to intractable crudes formed by partial couplings, homocouplings, and unidentified decomposition products. In fact, yields dramatically decreased, most likely due to the intrinsic nature of the sulfoxide, which is prone to be easily reduced or oxidized to the corresponding thioether and sulfone, as it happened to us because evidence of the other hosts **4-S** and **4-SO**_**2**_ were found. Additionally, hosts **4-SO** and **4-SO**_**2**_ might be obtained through oxidation of the parent compound **4-S**,^33^ but yields were of the same order, concluding that either protocol is valid.

**Scheme 2:**
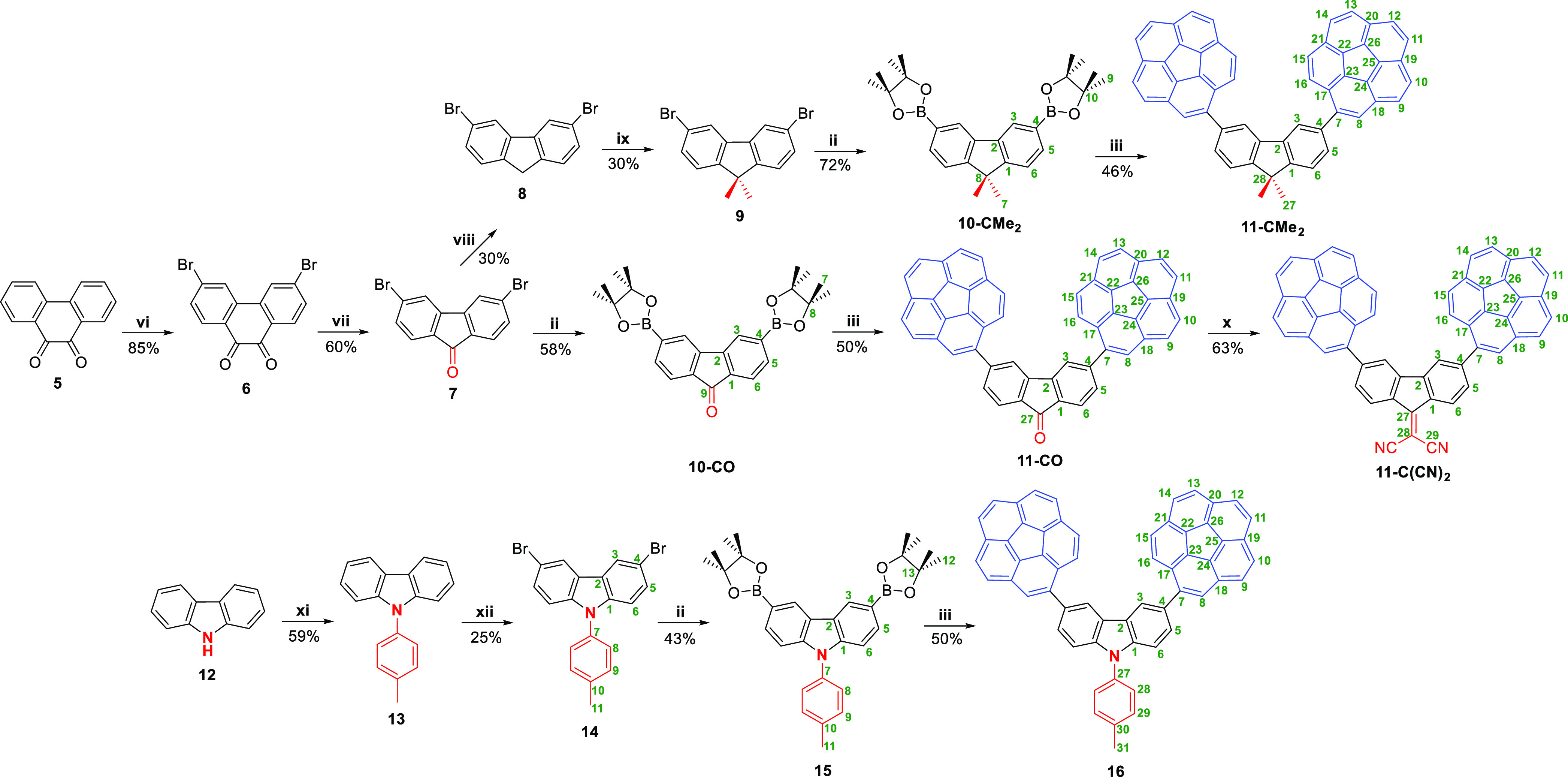
Synthetic Steps toward Corannulene-Derived Electronically Neutral Molecular Tweezer **11-CMe**_**2**_, Hosts with Strong EWG (**11-CO** and **11-C(CN)**_**2**_) and Host with EDG **16** Reagents and conditions: (ii) B_2_(pin)_2_, [PdCl_2_(dppf)], KOAc, dioxane, MW, 170 °C; (iii) Br-Cora, [PdCl_2_(dppf)], *^t^*BuONa, toluene, MW, 130 °C; (vi) Br_2_, BPO, PhNO_2_; (vii) KMnO_4_, KOH, H_2_O; (viii) N_2_H_4_·H_2_O, KOH, ethylene glycol; (ix) MeI, *^t^*BuONa, tetrahydrofuran (THF); (x) malonitrile, pyridine, 80 °C; (xi) *p*-bromotoluene, NaH, CuI, 18-Crown-6, dichlorobenzene; (xii) *N*-bromosuccinimide (NBS), CHCl_3_. Atom numbering has been altered for consistency purposes throughout the whole family.

The next stage consisted of the synthesis of electronically neutral host **11-CMe**_**2**_ and two hosts with strong EWG (**11-CO** and **11-C(CN)**_**2**_) (Scheme 2). All three share the common precursor phenanthrene-9,10-dione (**5**) which, after dibromination and oxidation, furnished 3,6-dibromo-fluoren-9-one (**7**). At this stage, the sequence branches into two sets of steps. On the one hand, the reduction of ketone **7** with a Wolff–Kishner protocol gave rise to 3,6-dibromo-9*H*-fluorene (**8**) which was dimethylated and subjected to already described Suzuki–Miyaura process yielding compound **11-CMe**_**2**_ with moderate yield. On the other hand, borylation of ketone **7** followed by Suzuki coupling gave tweezer **11-CO**. The latter could be condensed in a Knoevenagel reaction with malonitrile to provide the final compound **11-C(CN)**_**2**_ in good yield. Finally, to obtain *N*-tolyl compound **16**, the synthesis starts with carbazole (**12**), which, after *N*-arylation and bromination leading to intermediate **14**, it could be subjected to the typical corannulene grafting through C–C cross-coupling to furnish final host **16** in moderate yield (Scheme 2).

### Characterization of Synthesized Species

All new compounds have been fully characterized by means of one-dimensional (1D) and two-dimensional (2D) NMR (in CDCl_3_) techniques in solution, and their structural integrity has also been confirmed by high-resolution mass spectrometry (HRMS) measurements (Figures S1–S126).

Regarding the final compounds, typically, the fluorene-like tether signals appear at the lowest observed field as doublets or double doublets, being H^3^ the most deshielded proton in the majority of cases. For instance, regarding the sulfur-derived subfamily **4-S**, **4-SO**, and **4-SO**_**2**_, those aromatic signals show up between 7.90 and 8.65 ppm. Proton H^6^ is highly sensitive to the electronic nature of the bridgehead group, as expected due to its proximity to it. In the case of compound **11-CMe**_**2**_, H^6^ resonates at 7.67 ppm, whereas it appears at 8.63 ppm in host **11-C(CN)**_**2**_. The more withdrawing ability of the bridgehead group, the more deshielded H^6^ is. On the other hand, corannulene singlet H^8^ appears as the most deshielded sharp signal of the corannulene moiety around 8 ppm. All of the other corannulene protons resonate at higher field in a rather complicated second-order spin pattern we were able to solve thanks to 2D experiments (see the Supporting Information). These features are similar in toluene-*d*_8_ and allowed us to select the appropriate signals for supramolecular titration (see below).

### UV–Vis Absorption and Emission Properties

**Figure 2:**
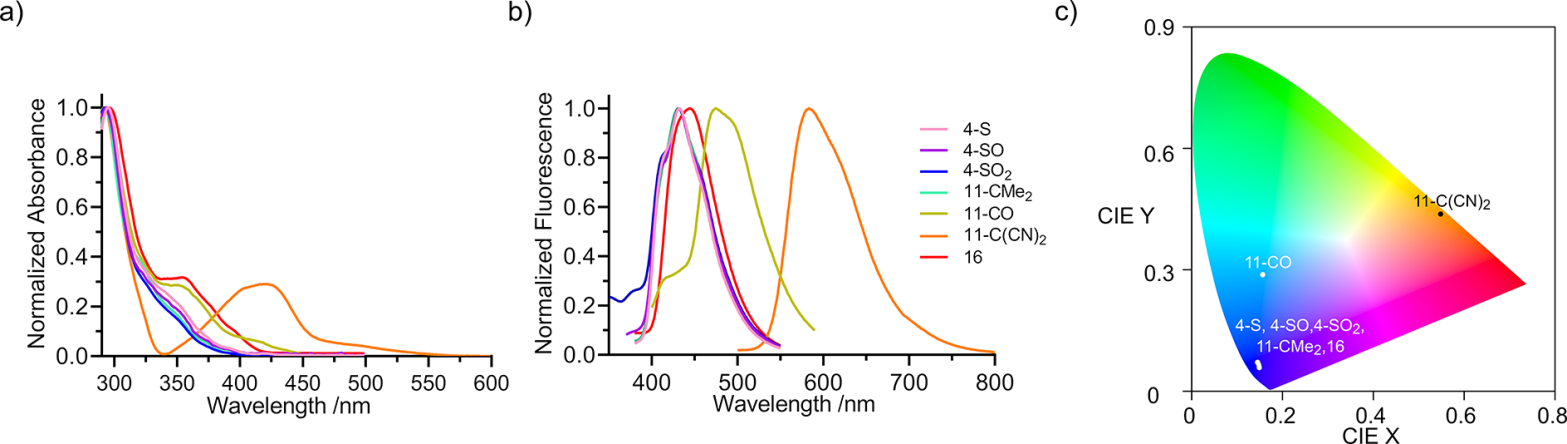
(a) UV–vis absorption spectra of compounds **4**, **11**, and **16** in toluene at a concentration of 10^–5^ M; (b) their emission spectra under the same conditions; (c) location of the emission properties of all of the compounds under study in the CIE 1931 color space.

**Figure 3:**
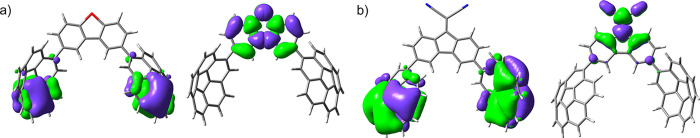
Frontier natural localized molecular orbitals (NLMOs) of selected molecules (see the Supporting Information for details on the level of theory). Isodensities plotted at 0.02 e·au^–3^. (a) Quasi-degenerated HOMO/HOMO–1 and LUMO/LUMO+1 of host **4-S**. (b) Quasi-degenerated HOMO/HOMO-1 to -5 and lowest unoccupied molecular orbital (LUMO) of host **11-C(CN)**_**2**_.

Compounds pertaining to the S-derived subfamily as well as compound **11-CMe**_**2**_ show a broad absorption band in the UV region (in toluene) between 290 and 400 nm typically ascribed to corannulene transitions (Figure 2a, Table 1, and Figures S127 and S128).^20^ The absence of absorption at higher wavelengths as well as the lack of sensitivity to solvent polarity suggest that the absorbing state is localized either at the corannulene moiety or at the tether with a neutral character.^58^ It appears to not belong to the direct transition between frontier orbitals (see Figures 3 and S170) and, therefore, no intramolecular charge transfer (ICT), understood as a CT between corannulene groups and tether, exists in the ground state of these species. No difference with respect to the oxidation state of the sulfur atom is observed either.^58^ Conversely, absorption spectra of hosts **11-CO**, **11-C(CN)**_**2**_, and **16** show an additional band with lower intensity at 350, 421, and 354 nm, respectively, being more pronounced for host **11-C(CN)**_**2**_, which suggests its ICT nature (Figures 2a and 3b, Table 1, and Figures S128 and S170). With regard to emission properties, again, all of the members of the sulfur-derived subfamily have a partially structured emission band with maxima around 425 nm in a nonpolar solvent such as toluene,^58^ consistent with its blue color (Figure 2b,c, Table 1, and Figures S129–S131). A similar observation can be done for compounds **11-CMe**_**2**_ and **16**, although their emission bands are broader. However, for hosts containing EWG as bridgeheads (**11-CO** and **11-C(CN)**_**2**_), their structureless emission bands appear at 475 and 583 nm, respectively with a notable change in color (Figure 2b,c, Table 1, and Figures S130 and S131). These bathochromic shifts with respect to the other members of the family suggest the presence of ICT in the excited state. Moreover, their polar nature can be confirmed by solvatochromism experiments (Figure S132) since λ_em_ shifts to lower wavelengths as the solvent polarity increases, reaching a maximum bathochromic shift of 81 nm in MeOH, indicating a more stabilized excited state. Emission quantum yields (QY, represented by Φ), carried out in the same solvent, are around 20% for the sulfur-derived subfamily and the electronically neutral compound **11-CMe**_**2**_, whereas it reaches 53% for compound **16**. On the other hand, hosts **11-CO** and **11-C(CN)**_**2**_ show a negligible QY (Table 1). There is a trend of a lower QY as the bridgehead group becomes more acceptor. Measured decay lifetimes (τ) range from 5.08 to 10.54 ns, fitted with a mono-exponential function, for most of the hosts, which are typical values for this kind of compounds,^20^ confirming the existence of a fluorescent singlet excited state (Table 1). Interestingly, hosts **11-CO** and **11-C(CN)**_**2**_ display a different behavior with respect to the rest of the family. Their lifetimes are below 1 ns, suggesting a very efficient relaxation process, exclusively due to the acceptor character of the bridgehead group. However, this result is not surprising as it is known, for instance, that fluoren-9-yliden malononitriles have decay lifetimes of just hundreds of picoseconds (below 0.5 ns) in toluene as there is a nonradiative deactivation process occurring right after photoexcitation and formation of a CT state through a geometry deformation consisting of a double-bond twist of the dicyanovinyl group encompassing a pyramidalization of the quaternary carbon that is substituted by nitrile groups.^59^

**Table 1:** Spectrophotometric Parameters of Hosts **4**, **11**, and **16** in Toluene^a^

compound	λ_abs_/nm	ε·10^4^/M^–1^·cm^–1^	λ_em_/nm	Φ	τ/ns
**4-S**	295	7.77	415/432/456	0.23	8.51
**4-SO**	294	4.98	413/432/457	0.17	9.15
**4-SO**_**2**_	293	8.48	413/432/458	0.19	10.54
**11-CMe**_**2**_	293	7.50	430	0.20	9.17
**11-CO**	292/350	8.28/2.37	475	0.04	b
**11-C(CN)**_**2**_	292/421	8.51/2.47	583	0.01	b
**16**	296/354	7.02/2.22	444	0.53	5.08

a At a concentration of 10^–5^ M.

b Below the instrument lower detection limit (1 ns).

### Electrochemical Properties

**Figure 4:**
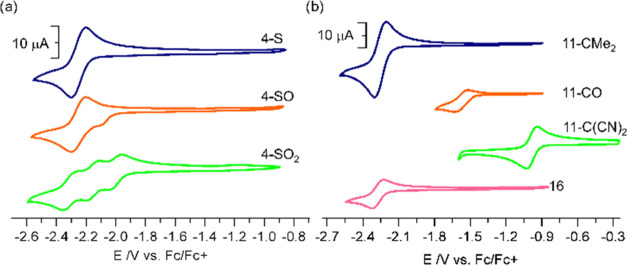
Cyclic voltammograms carried out at room temperature in a one-compartment cell equipped with a glassy carbon electrode, a silver wire counter electrode, and a Ag/AgCl wire as pseudo-reference electrode in deareated DMF at a scan rate of 100 mV·s^–1^ and a solution of NBu_4_PF_6_ (0.1 M) as the electrolyte of (a) compounds **4** and (b) compounds **11** and **16** at 1 mM. Potentials are referenced against *F*_c_/*F*_c_^+^ and plotted using IUPAC convention.

As expected, the photophysical properties of studied hosts decisively depend on the intrinsic nature of the bridgehead group since ICT is more pronounced as the strength of the EWG increases, but there is still a lack of clear evidence of the impact on the corannulene moiety properties due to the fact that bridgehead groups are not directly attached to the polycyclic hydrocarbon and, as stated before, the effect is supposed to decrease with the distance. We therefore studied the electrochemical features of synthesized compounds by cyclic voltammetry (CV) in *N*,*N*-dimethylformamide (DMF) deareated solutions (Figure 4, Table 2, and Figure S133). To simplify the analysis, we focused on the reduction part of the voltammograms, especially with regard to the first reduction potential, as it typically belongs to the reversible first reduction of corannulene moiety. Within the S-derived family, a slight anodic shift with respect to corannulene is observed and the magnitude is in the range of what is expected.^33,41,43^ Not surprisingly, the highest shift is observed for the most oxidized compound **4-SO**_**2**_, ca. 0.3 V more positive than pristine corannulene.

**Table 2:** Reduction Potentials of Hosts **4**, **11**, and **16** in DMF^a^

compound	*E*^1^/V	*E*^2^/V	*E*^3^/V	*E*^4^/V
**Corannulene**	–2.31^b^	–2.86^b^		
**4-S**	–2.24	–2.73^c^		
**4-SO**	–2.23	–2.73	–3.10	
**4-SO**_**2**_	–2.00	–2.15	–2.29	–2.67
**11-CMe**_**2**_	–2.26	–2.76^c^	–3.45^c^	
**11-CO**	–1.49	–2.24^c^		
**11-C(CN)**_**2**_	–0.98	–1.64	–2.42	–3.04^c^
**16**	–2.28	–2.83^c^		

a A solution of NBu_4_PF_6_ (0.1 M) was used as the electrolyte. Scan rate of 100 mV·s^–1^. Potentials are referenced against *F*_c_/*F*_c_^+^.

b According to ref (33).^33^

c Measured by square-wave voltammetry (SWV).

On the other hand, electronically neutral and donor hosts **13-CMe**_**2**_ and **16**, respectively, do not show a substantial change in the first reduction potential of the buckybowl, suggesting a very mild influence of these groups, as also observed in the UV–vis absorption and emission experiments. Conversely, hosts **11-CO** and **11-C(CN)**_**2**_, show a dramatic anodic shift of their first reduction potentials by 0.82 and 1.33 V, respectively (Figure 4, Table 2). The presence of EWG, albeit, moderately far, significantly modify the electronic properties of corannulene,^30^ turning it into a more efficient electron acceptor, by lowering the energy of its LUMO.

Thanks to both photophysical and electrochemical experiments, it is possible to estimate the energy of the frontier orbitals and their corresponding gaps (Table 3). The results point toward what has been discussed so far. Again, within the sulfur-derived subfamily, the LUMO energy decreases as the oxidation of the heteroatom increases (more electron acceptor), with a concomitant highest occupied molecular orbital (HOMO) stabilization, slightly increasing the gap. Hosts **11-CMe**_**2**_ and **16** show similar energy levels. As expected, the latter has the least stabilized HOMO given its donor properties. On the other hand, acceptors **11-CO** and **11-C(CN)**_**2**_ show the most stabilized LUMOs (close to the LUMO energy of C_60_ and C_70_, confirming its good acceptor properties) (Figure S134). Moreover, compound **11-C(CN)**_**2**_ has a particularly stabilized LUMO and destabilized HOMO, showing the smallest gap between frontier orbitals of the whole series. Computed *E*_HOMO_ and *E*_LUMO_ values by density functional theory (DFT) compare very well with the absolute values obtained by experimental techniques (Table 3). Theoretical HOMO values deviate more than their LUMO counterparts, but the exact same trend is observed in all cases.

**Table 3:** Estimated HOMO and LUMO Levels of Compounds **4**, **11**, and **16** from Experimental Absorption and Electrochemical Data

compound	λ_onset_/nm	*E*_gap_^opt^/eV^a^	*E*^1^/V	*E*_LUMO_/eV^b^	*E*_LUMO_^DFT^/eV^d^	*E*_HOMO_/eV^c^	*E*_HOMO_^DFT^/eV^d^
**4-S**	386	3.21	–2.24	–2.22	–2.29	–5.43	–4.92
**4-SO**	379	3.27	–2.23	–2.34	–2.53	–5.61	–5.13
**4-SO**_**2**_	377	3.29	–2.00	–2.48	–2.66	–5.77	–5.30
**11-CMe**_**2**_	378	3.28	–2.26	–2.23	–2.24	–5.51	–4.98
**11-CO**	403	3.08	–1.49	–2.93	–3.04	–6.01	–5.19
**11-C(CN)**_**2**_	556	2.23	–0.98	–3.48	–3.70	–5.71	–5.30
**16**	412	3.01	–2.28	–2.21	–2.20	–5.22	–4.58

a Estimated by the absorption onset values: *E*_gap_^opt^ = 1240/λ_onset_.

b Estimated based on the onset of the first reduction potential measured by CV: *E*_LUMO_ = −4.4 – (*E*_red_^onset^ – *E*_1/2_^Fc^).

c Estimated according to: *E*_HOMO_ = *E*_LUMO_ – *E*_gap_^opt^.

d Calculated by DFT at the level of theory: B97D3/6-31G(d,p)/PCM(toluene).

### Fullerene Recognition Properties

**Figure 5:**
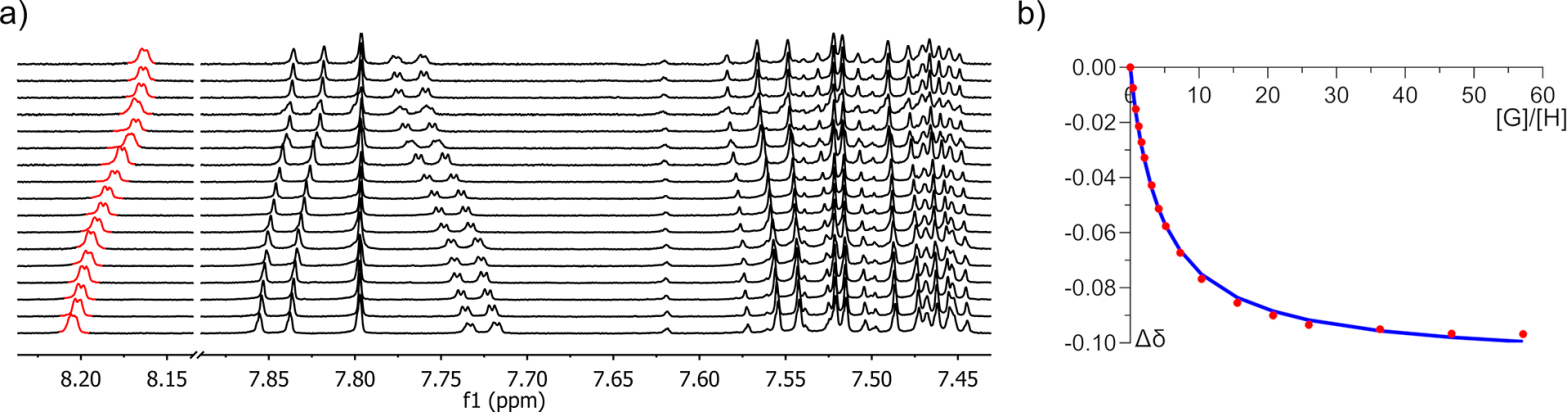
(a) Aromatic region of the ^1^H NMR spectrum of host **11-CMe**_**2**_ in toluene-*d*_8_ at 298 K in a typical titration experiment with C_60_. (b) Example of a binding isotherm of the chemical shift highlighted in red. The blue line corresponds to the fit obtained by nonlinear regression with a 1 to 1 model.

Finally, we conducted supramolecular titrations in solution to estimate the binding affinity of all hosts under study. We finally concluded that NMR is the most appropriate instrumental technique because (1) several aromatic signals are relatively isolated in the ^1^H NMR spectrum, allowing easy tracking, and (2) association constants are not expected to be high enough to observe changes in UV–vis absorption considering our late previous results.^15,20^ In fact, no CT band has been observed upon fullerene addition to a solution of the host. In contrast, emission quenching is indeed effectively present (Figures S168–S169). However, the low concentrations required for such experiments (10^–5^–10^–6^ M) are far beyond the reciprocal expected constant (ca. 10^3^ M^–1^), complicating the further analysis and providing large errors in the final nonlinear regression. Hence, the addition of aliquots of C_60_ provokes the change of the chemical shift of several protons. As commented above, luckily, protons H^3^, H^5^, and H^6^ pertaining to the tether, as well as peripheral protons H^8^ and H^16^ from the corannulene moiety experienced the most remarkable ones. They were used to plot such a change against the molar fraction of the guest and fit the binding isotherm through a nonlinear regression. Particularly, proton H^3^ typically experiments an upfield shift, whereas protons H^5^ and H^6^ experience the opposite trend (Figure 5a). This is an indirect confirmation of the differential behavior due to the presence of the shielding effect of the fullerene as hydrogen H^3^ points toward the cavity formed by both corannulene subunits, whereas the other hydrogens of the tether do not. Experimental datapoints were fitted to a standard 1 to 1 stoichiometry as well as all of the flavors of 2 to 1 stoichiometries according to modern methods^60^ (Figure 5b). In all cases, neither ternary model gave a variance of fit ratio (cov_fit_ factor), a parameter that reliably assesses the relative quality of the fits, over the accepted threshold of 3,^60,61^ being 1.57 for the best one with association constants having physical meaning (see the Supporting Information for detailed values). Therefore, all supramolecular complexes studied in this work seem to show a 1:1 stoichiometry with values summarized in Table 4.

**Table 4:** Association Constants of Hosts **4**, **11**, and **16** with C_60_ Obtained by NMR Titrations in Toluene-*d*_8_ at 298 K Considering a 1:1 Stoichiometry

compound	*K*_a_/M^–1^	Δ*G*_a_/kJ·mol^–1^
**4-S**	(1.28 ± 0.02) × 10^3^	–17.73 ± 0.04
**4-SO**	(1.04 ± 0.01) × 10^3^	–17.22 ± 0.02
**4-SO**_**2**_	(1.30 ± 0.03) × 10^3^	–17.77 ± 0.06
**11-CMe**_**2**_	(5.20 ± 0.03) × 10^2^	–15.5 ± 0.1
**11-CO**	(5.07 ± 0.08) × 10^2^	–15.4 ± 0.4
**11-C(CN)**_**2**_	(7.28 ± 0.05) × 10^2^	–16.3 ± 0.2
**16**	(5.28 ± 0.04) × 10^2^	–15.5 ± 0.2

**Figure 6:**
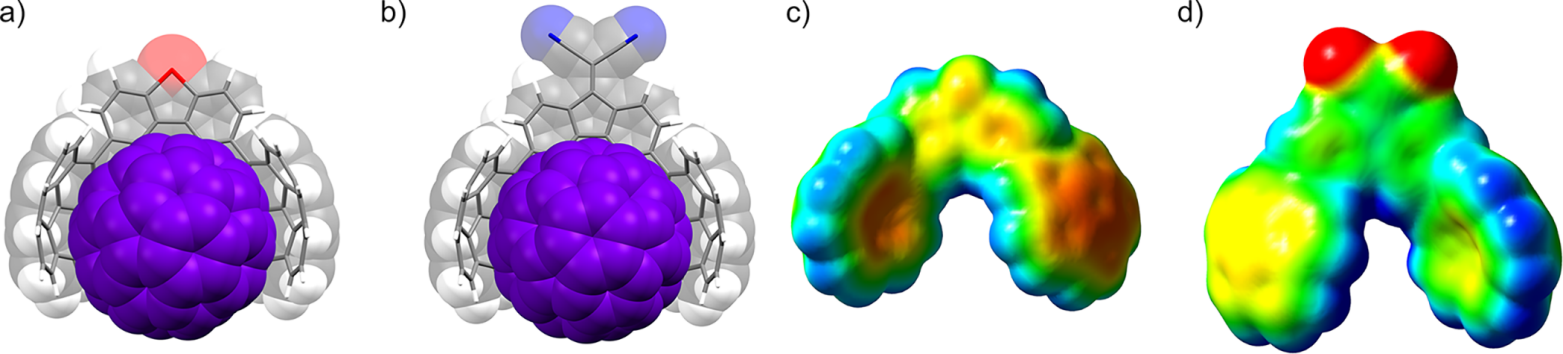
DFT-optimized structures of assemblies C_60_@**4-S** (a) and C_60_@**11-C(CN)**_**2**_ (b) at the B97D3/6-31G(d,p)/PCM(toluene) level of theory showing similar geometries. Electrostatic surface potential (ESP) of compounds **4-S** (c) and **11-C(CN)**_**2**_ (d) synthesized in this work plotted as a mapped surface over the computed DFT density (isovalue: 0.0004 e·au^–3^) showing the differential charge distribution over corannulene moieties (red means a concentration of negative charge, whereas blue means a concentration of positive charge).

As commented in the Introduction section, from a simplistic point of view, accounting for the donor–acceptor properties and considering C_60_ as a good acceptor molecule, one would consider host **16** to have the best association constant since it possesses the most destabilized HOMO (Table 3 and Figure S134), whereas host **11-CO** would show the worst recognition capability considering the effect of the bridgehead group is substantial. Nonetheless, both show a comparable association constant. In fact, all of the hosts, except for the sulfur-derived subfamily show very similar constants without absolutely any trend with respect to their donor–acceptor properties (Table 4). On the other hand, the S-derived subfamily show constants around 10^3^ M^–1^ doubling the value of the other members of the series in almost all cases. This is a non-negligible effect worth discussing. Experimental association constants are in the expected range of other similar corannulene-based molecular pincers.^15,19,20,62^ But the difference in this case is not immediate to grasp a priori as all of the hosts are not expected to experience great variations in the structure of their host–guest complexes as the tweezer scaffold is generally maintained throughout the whole series. Correlation between experimental Gibbs free energy of association (Δ*G*_a_ in Table 4) and Hammett constants^63−65^ is nonexistent either (Figure S136), suggesting that the electrostatic interactions do not play a major role despite the great influence of the bridgehead group on the electrostatic surface of corannulene moieties (Figures 6c,d and S171).^66^ By ruling out those forces, it appears dispersion interactions are the most dominant contributions.

To get a better understanding, we carried out DFT studies on all of the 1 to 1 inclusion complexes with C_60_ studied so far. Optimized structures reveal, as anticipated, an almost equal arrangement of the tweezer structure, locating the fullerene molecule in the cavity formed by both corannulene units on one side of the host establishing interactions mainly with one hemisphere of the fullerene (Figure 6a,b). This arrangement is typically set to avoid internal hydrogen repulsion (H^3^ in this case). This is very common in tethers with a short spacer group and a lack of conformational mobility (see the Supporting Information for the full set of optimized structures).^15,19,20,62^ In a closer inspection to computed geometries, key structural factors such as penetration depths (distance between the centroid of the corannulene bowl and the centroid of the fullerene moiety) and clamping depth (distance between the midpoint of the bond connecting both phenyl units in the tether and the centroid of the fullerene moiety)^67^ reveal that, while the location of C_60_ between two corannulenes is kept throughout the whole series (6.86 Å on average) it is farther within the S-derived subfamily than in the other hosts (7.40 *vs* 7.18 Å on average) (Figure S172 and Table S8). This seems to be a consequence of the longer S–C^1^ bond distance (1.80 *vs* 1.48 Å on average) (Table S8) pointing toward a better adaptation ability of the tether within hosts **4**. We then performed an Energy Decomposition Analysis (EDA)^68^ to search the different energy contributions in the supramolecular association. The interaction energy is split into three attractive factors (namely, electrostatic, orbital or charge transfer, and dispersion interactions) and a repulsion factor (Pauli) (see the Supporting Information for details about the calculation). Again, the results reveal a different behavior between hosts **4** and **11**, **16** (Table S9). First, in all cases, the dispersion contribution is essentially equal in relative terms and is the highest of all of the attractive forces, being the most responsible for the interaction (46%). However, it also shows a slightly higher contribution of the electrostatic and orbital (charge transfer) in the sulfur-derived subfamily with respect to the other hosts (33 to ca. 35% and 19%, respectively). Moreover, repulsion is slightly higher as well. This behavior is a consequence of mentioned differential adaptability of the tether when forming the inclusion complex with the fullerene. Interaction energies provide a trend that partially matches with the experimental observations, but it fails when predicting the recognition properties of hosts **11-CMe**_**2**_ and **16** as they are higher than expected. Consequently, we evaluated the total electronic binding energy by including the deformation energy penalty of the host accounting for its conformational freedom and the preorganization (or lack thereof)^69^ and considered other schemes such as Natural Decomposition Analysis (NEDA)^70^ and Counterpoise^71^ (Table S10). Surprisingly, hosts **4** show a negative deformation energy, meaning that they become more stable when forming the inclusion complex with fullerenes (Table 5). The case of induced-fit host–guest chemistry is known in artificial systems, however.^72−74^ Resulting electronic binding energies show a much more reliable trend according to the experimental results, displaying a higher value for the S-derived family. Conversely, compound **16** seems to be overestimated with this approach; however, a possible stronger influence of entropic penalty could be responsible for the observed moderate experimental Gibbs free energy.

**Table 5:** Computed Electronic Interaction and Binding Energies of Studied Inclusion Complexes with C_60_

adduct	*E*_int_/kJ·mol^–1^^a^	*E*_def_/kJ·mol^–1^^b^	*E*_bind_/kJ·mol^–1^
C_60_@**4-S**	–139.66	–0.69	–140.35
C_60_@**4-SO**	–139.54	–1.58	–141.12
C_60_@**4-SO**_**2**_	–139.08	–0.67	–139.74
C_60_@**11-CMe**_**2**_	–141.84	3.83	–138.01
C_60_@**11-CO**	–139.33	1.82	–137.51
C_60_@**11-C(CN)**_**2**_	–139.58	1.44	–138.14
C_60_@**16**	–142.51	3.33	–139.17

a According to the Counterpoise scheme.

b Deformation energy of host only (see the Supporting Information (SI) for full details).

**Figure 7:**
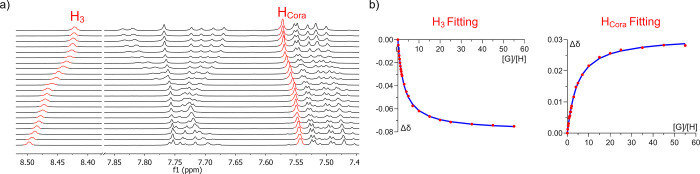
(a) Aromatic region of the ^1^H NMR spectrum of host **4-S** in toluene-*d*_8_ at 298 K in a titration experiment with C_70_. (b) Example of two binding isotherms of the chemical shifts highlighted in red. The blue line corresponds to the fit obtained by nonlinear regression with a 2 to 1 model.

**Figure 8:**
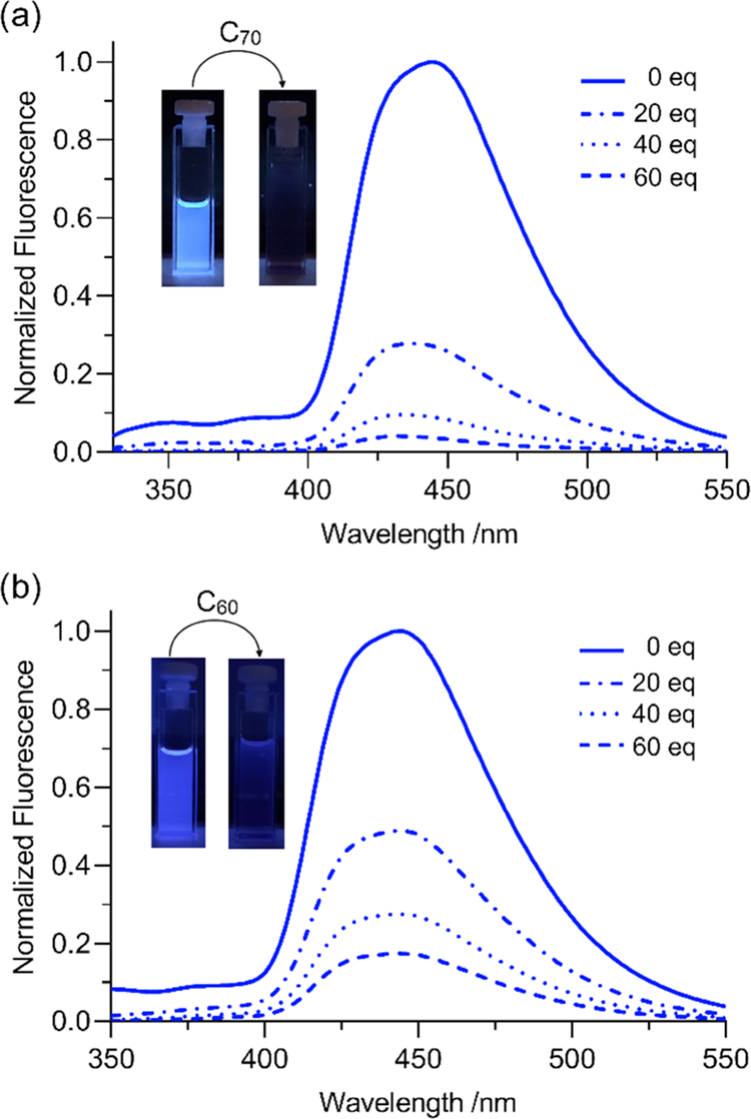
Example of a normalized quenching emission in toluene of host **16** (10^–6^ M) (λ_exc_ = 302 nm) upon addition of C_70_ (a) and its comparison with C_60_ (b).

**Figure 9:**
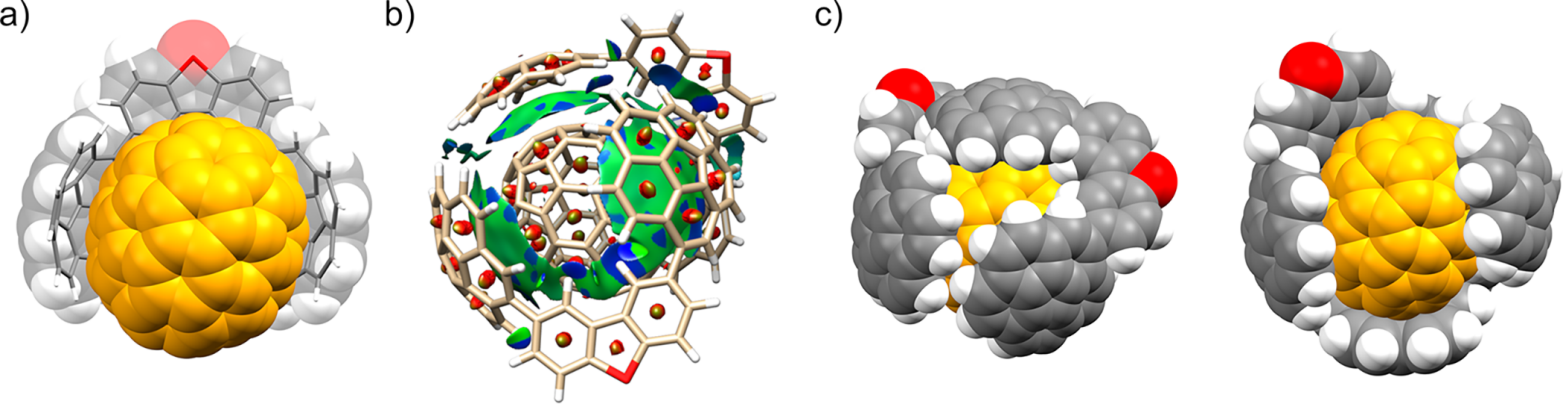
(a) DFT-optimized structure of assembly C_70_@**4-S** at the B97D3/6-31G(d,p) level. (b) Noncovalent interactions represented as gradient isosurfaces (with an isovalue of 0.3 a.u.) for supramolecular assembly C_70_@(**4-S**)_2_ with an RGB pattern: red indicates repulsion, green indicates weak attraction, and blue indicates strong attraction. (c) Space-filling models of the inclusion complex C_70_@(**4-S**)_2_ showing two orientations with the most (left) and least (right) covered fullerene surface.

We then assessed the affinity capabilities toward C_70_ by employing the same protocol we used for C_60_. Again, several isolated aromatic signals could be tracked upon fullerene addition so the change in chemical shift was plotted against the molar fraction of the guest to provide a binding isotherm to be fit. Such changes in chemical shift were very similar to the ones observed for C_60_ in terms of directionality (Figure 7a,b). Additionally, emission quenching was also tested in these systems (Figures 8a,b and S168–S169). It appears that it is effective after the addition of a moderate amount of guest at micromolar concentrations and is more pronounced than the experiment performed for C_60_ (Figure 8b). A more efficient design could increase this property to, perhaps, provide good fluorescent fullerene sensors. Fitted values are shown in Table 6. Remarkable results were obtained with this new guest. Generally, the affinity for C_70_ is higher, which is the case for all 1:1 stoichiometries, being the highest for the S-derived family, suggesting the same conclusions found for C_60_. However, values are not much higher than the previous guest counterparts, as they are 3 times better in the best case (they are typically expected to be one order of magnitude better). This result indicates just a mild preference for C_70_ rather than C_60_. All binding isotherms were fitted to all of the flavors of 2:1 stoichiometries.^60^ Surprisingly, host **4-S** shows a divergent behavior with respect to all of the other members of the series (Table 6). The above-mentioned cov_fit_ factor is now 17.3, quite higher than the “3 threshold”^60^ and within the order of magnitude of strong acceptors with similar stoichiometries.^61^ This ensures that the 2:1 model has a much better quality fit than the common 1:1 model. Noncooperative flavor (assuming that *K*_1_ = 4*K*_2_ and, therefore, α = 1) has a very similar quality to the full flavor (with no assumptions) and both are much better than the other two. An *F*-test was performed followed by the calculation of the subsequent *P*-value^60^ at the 95% level of confidence, returning a value of 1.0 which is clearly higher than 0.05. This allows us to accept the null hypothesis and, therefore, the simpler noncooperative model is inferred as it fits the data better. Additionally, statistical errors for the 2:1 noncooperative model (SS_y_ and χ^2^)^60^ are among the lowest of the series (see the Supporting Information for all of the values). This means that there is absolutely no cooperativity in the recognition process of the second host. Fortunately, despite the moderate absolute value of the individual association constants (although the overall binding constant β, calculated as *K*_1_ × *K*_2_, is (1.93 ± 0.02) × 10^6^ M^–2^), we were able to confirm the existence of the 2:1 adduct by MS detection (Figure S165). Signals coming from any 1:1 inclusion complex (including C_60_ adducts) could not be obtained under the same experimental conditions, confirming the strength of assembly C_70_@(**4-S**)_2_. With regard to the other hosts, none of them gathers all of the requirements to consider any of the 2:1 flavors due to very improbable association constants (extremely low or even negative), large statistical errors, or cov_fit_ factors lower than 3 (Table 6). Nonetheless, noncooperative flavor results are also shown in such a table (see the Supporting Information for a full disclosure of all values). Geometries of supramolecular adducts C_70_@**4-S** and C_70_@(**4-S**)_2_ were minimized with the same level of theory used for C_60_ inclusion complexes (see the Supporting Information for details about the protocol we followed). Regarding the former, the structure is generally very similar to the Buckminsterfullerene counterpart, suggesting the same behavior as discussed before for C_60_ (Figure 9a). In this case, however, deformation energy becomes penalizing by 10.33 kJ·mol^–1^, nearly 3 times more penalizing than the worst case of C_60_ assemblies (Table 5). For complex C_70_@(**4-S**)_2_, it is possible to observe the practically full coverage of the fullerene outer surface, albeit not in a homogeneous manner due to the dissymmetric nature of host **4-S** (Figure 9c). Repulsion between both hosts seems to be minimized while interactions with all four corannulene subunits and both tethers are maximized due to the better adaptability of this host. The NCI plot^75^ reveals an extended attraction surface covering the guest as well as mild CH−π interactions between hydrogens from the corannulene group of one host and the thiophene tether of the other host (Figures 9b and S174, S175).

**Table 6:** Association Constants of Hosts **4**, **11**, and **16** with C_70_ Obtained by NMR Titrations in Toluene-*d*_8_ at 298 K Considering 1:1 and 2:1 Stoichiometries

compound	*K*_a_/M^–1^^a^	Δ*G*_a_/kJ·mol^–1^	*K*_1_/M^–1^^b^	*K*_2_/M^–1^^b^	Δ*G*_1_/kJ·mol^–1^	Δ*G*_2_/kJ·mol^–1^	cov_fit_ factor^c^
**4-S**	(1.98 ± 0.05) × 10^3^	–18.82 ± 0.06	(2.78 ± 0.02) × 10^3^	(6.96 ± 0.02) × 10^2^	–19.66 ± 0.02	–16.22 ± 0.07	17.3
**4-SO**	(2.14 ± 0.03) × 10^3^	–19.01 ± 0.03	(5.09 ± 0.02) × 10^2^	(1.27 ± 0.02) × 10^2^	–15.5 ± 0.1	–12.0 ± 0.4	1.63
**4-SO**_**2**_	(1.95 ± 0.03) × 10^3^	–18.78 ± 0.04	21.0 ± 0.1	5.2 ± 0.1	–8 ± 1	–4 ± 4	6.41
**11-CMe**_**2**_	(1.55 ± 0.02) × 10^3^	–18.21 ± 0.03	68.8 ± 0.2	17.2 ± 0.2	–10.5 ± 0.7	–7 ± 3	3.43
**11-CO**	(1.23 ± 0.01) × 10^3^	–17.64 ± 0.02	d	d	d	d	d
**11-C(CN)**_**2**_	(1.69 ± 0.01) × 10^3^	–18.42 ± 0.01	(2.94 ± 0.01) × 10^2^	73.4 ± 0.1	–14.09 ± 0.08	–10.7 ± 0.3	3.08
**16**	(1.72 ± 0.02) × 10^3^	–18.47 ± 0.03	(2.70 ± 0.01) × 10^2^	67.4 ± 0.1	–13.88 ± 0.09	–10.4 ± 0.4	2.64

a 1:1 stoichiometry.

b 2:1 stoichiometry with noncooperative flavor (*K*_1_ = 4*K*_2_ and α = 1).

c Calculated as the ratio between the raw covariance of the 2:1 model versus the raw covariance of the simplest 1:1 model.^60^ Covariance is obtained as cov_fit_ = variance(*y*_calc_)/variance(*y*_data_).

d This fit did not provide any association constant with physical meaning and a cov_fit_ value over 3.

Mentioned CH−π interactions were estimated to be −33.72 kJ·mol^–1^, a respectful and non-negligible value^50^ (see the Supporting Information for more details). As expected, the interaction energy becomes more negative as it is −145.77 kJ·mol^–1^ for complex C_70_@**4-S** and −278.82 kJ·mol^–1^ for complex C_70_@(**4-S**)_2_. It is important to point out that the difference in energy is not twice more negative (−291.54 kJ·mol^–1^ theoretically). This means that the arrangement of hosts and guest differs from the parent 1:1 assembly, resulting in a lower expected interaction energy. The origin of the favorable ternary complex formation is thus somewhere else. Again, the most interesting result arises from the deformation energy, being extraordinarily favorable (−29.14 kJ·mol^–1^), resulting in an electronic binding energy of −307.97 kJ·mol^–1^. This is −37.09 kJ·mol^–1^ (or −37.03 kJ·mol^–1^ if calculated with a different approach, see the Supporting Information for more details) more favorable than twice as much the interaction energy of adduct C_70_@**4-S**. The induced-fit effect is remarkable in this case as both hosts are more stable than their free counterparts or forming the 1:1 assembly C_70_@**4-S** (see Table S11 for a summary of all computed energies). Albeit the computed electronic binding energy is very high in absolute value, it is worth mentioning that, in this particular supramolecular inclusion complex, a penalizing cost in the form of entropy and desolvation effects is paid, resulting in more moderate, yet important, observed experimental binding Gibbs free energies (Table 6). To confirm this hypothesis, we carried out NRM titrations for the formation of assemblies C_60_@**4-S** and C_70_@(**4-S**)_2_ at different temperatures (288, 298, and 308 K) (Figures S166 and S167, Table S7).^54^ A very mild dependence with temperature was found for the former (Gibbs free energy is ca. 18 kJ/mol in all cases), whereas stronger for the latter. As foreseen in the Introduction section, this association is clearly enthalpy-driven^24,54^ and mildly penalizing entropically for inclusion complex C_60_@**4-S** (−25.7 ± 0.2 and −0.027 ± 0.002 kJ·mol^–1^ for Δ*H* and Δ*S*, respectively). Although Sygula’s Buckycatcher I shows a particularly favorable entropy contribution in toluene-*d*_8_,^54^ in this case, the behavior of host **4-S** lies within the expected trends, most likely due to a higher configurational entropy that compensates a possible solvation entropy gain. Moreover, the formation of C_70_@(**4-S**)_2_ was found to possess a much more prominent entropic penalty. As the enthalpy variation is highly negative (−203 ± 2 kJ·mol^–1^) it is strongly compensated by an unfavorable entropy (−0.57 ± 0.08 kJ·mol^–1^). In other words, ca. 80% of the enthalpic contribution is canceled out by entropy changes, confirming the observed moderate experimental Gibbs free energy.

## Conclusions

In summary, a series of corannulene-substituted molecular tweezers bearing a fluorene-like tether with a variety of groups having different electronic natures has been synthesized and fully characterized. Their photophysical and electrochemical properties have been assessed demonstrating the impact of the bridgehead group in their behavior, showing a characteristic donor nature for *N*-tolyl carbazole **16**, whereas a particular acceptor nature for fluorenone **11-CO** and ylidenmalononitrile **11-C(CN)**_**2**_ has been found. The latter seems to have a very similar HOMO/LUMO levels and frontier orbitals gap than those for fullerenes C_60_ and C_70_, paving the way toward promising corannulene-based nonfullerene acceptors.^30,33^ The supramolecular recognition capabilities toward fullerenes C_60_ and C_70_ have been studied as a function of the bridgehead group electronic nature. Experimental measurements provided moderate to high association constant values. No correlation between the donor/acceptor behavior and the experimental binding Gibbs free energy has been found, suggesting that charge transfer and electrostatic interactions are not relevant when London dispersion forces become dominant (46% of the interaction energy belongs to dispersion according to DFT results). However, the thiophene subfamily (**4-S**, **4-SO**, and **4-SO**_**2**_) showed the highest affinities as an induced-fit effect is revealed, according to theoretical calculations, which in turn enhances the charge transfer and electrostatic interactions due to a better adaptation of the host when forming the inclusion complex. In the case of fullerene C_70_, this effect is extraordinarily exacerbated permitting the formation of a ternary 2 to 1 assembly C_70_@(**4-S**)_2_ not observed for its oxidized versions. These findings are not totally surprising as there are other examples of relatively flexible sulfur-based nonplanar hosts for fullerenes able to form this kind of supramolecular inclusion complexes with C_70_.^69,76^ All of the results reported in this work have established a clear roadmap we plan to exploit in the near future regarding the efficient design of semiflexible sulfur-based molecular tweezers or clips bearing multiple units of corannulene to find the maximum fullerene coverage.

## Experimental Section

### General Methods

Reagents were purchased from regular suppliers and used without further purification. 1-Bromocorannulene (Br-cora) was acquired from Synoi Chemicals (http://synoichemicals.uva.es/), whereas 2,2′-bis(methylthio)-5,5′-bis(corannulyl)-1,1′-biphenyl,^20^ 3,6-dibromophenanthrene-9,10-dione (**6**),^77^ 3,6-dibromo-fluoren-9-one (**7**),^77^ 3,6-dibromo-9*H*-fluorene (**8**),^77^ and 3,6-dibromo-9,9-dimethyl-fluorene (**9**)^77^ were prepared according to the reported procedure. Solvents were of analytical grade or spectrophotometric grade. They were either used as purchased or dried according to procedures described elsewhere.^78,79^ Microwave reactions were carried out with an Anton Paar Monowave 300 Reactor using tightly capped flasks G10 (for volumes up to 10 mL) specially designed for the apparatus. All reactions under inert atmosphere (when needed) were performed with standard Schlenk techniques. They were also used as a preliminary step for degassing microwave flasks when inert atmosphere was necessary in microwave reactions. Column chromatography separations were carried out using Silica gel 60 (particle size 0.040–0.063 mm; 230–400 mesh; Merck, Germany) as the stationary phase and thin-layer chromatography (TLCs) were performed on precoated silica gel plates (0.25 mm thick, 60 F254, Merck, Germany) and observed under UV light. Purifications by centrifugation were performed in an Ortoalresa UNICEN centrifuge. NMR spectra were recorded on Agilent DD2 500 and Agilent MR 400 instruments. ^1^H and ^13^C NMR chemical shifts are reported in parts per million (ppm) and are referenced to TMS, using residual solvent peak as an internal reference. Coupling constants (*J*) are reported in hertz (Hz). Standard abbreviations used to indicate multiplicity: s = singlet, d = doublet, m = multiplet, dd = doublet of doublets. ^1^H and ^13^C assignments were performed by utilizing 2D NMR methods (gDQFCOSY, band selective heteronuclear single quantum coherence (HSQC), band selective heteronuclear multiple bond correlation (HMBC), gradient crisis HSQC and gradient crisis HMBC) Structural assignments were made with additional information from gCOSY, gHSQC, and gHMBC experiments. High-resolution mass spectra were recorded at mass spectrometry service of the Laboratory of Instrumental Techniques of the University of Valladolid (L.T.I., www.laboratoriotecnicasinstrumentales.es). A MALDI-TOF system (MALDI-TOF) Bruker Autoflex Speed (N2 laser (337 nm, pulse energy 100 μJ, 1 ns), acceleration voltage 19 kV, reflector and linear positive mode) was used. Trans-2-[3-(4-*tert*-butylphenyl)-2-methyl-2-propenylidene] malonitrile (DCTB) and 1,8-dihydroxy-9(10*H*)-anthracenone (dithranol) were used as matrixes. A UPLC-MS system (UPLC: Waters ACQUITY H-class UPLC; MS: Bruker Maxis Impact) by electrospray ionization (ESI positive and negative) was utilized as well. HRMS spectra were analyzed using Bruker DataAnalysis 4.1© (www.bruker.com). Steady-state UV/vis absorption spectroscopy was carried out on a PerkinElmer Lambda 265 spectrophotometer, whereas emission spectroscopy was performed on a Cary Eclipse Fluorescence, using quartz cuvettes with a path length of 1.0 cm in spectrophotometric grade toluene as the solvent (along with THF, CHCl_3_, and MeOH for solvatochromism measurements). Time-resolved fluorescence measurements were carried out using the single-photon counting technique with ns time resolution. A high repletion pulsed light source is used to excite the sample and the photons emitted are processed using the TCC1 card in the computer. Fluorescence decays were obtained with the time-correlated single-photon counting (TCSPC) and MCP-PMT counter module (TCC2) of the FLS980 spectrometer (Edinburgh Instruments). The excitation source was a diode laser with excitation wavelengths 280 and 405 nm. EPLs-lasers produce picosecond duration pulses <1 ns at repetition rates up to 20 MHz (50 ns). Emission slit used was 4 nm. Fluorescence decays were analyzed with the method of nonlinear least squares iterative deconvolution and the quality of the fits was judged by the values of the reduced Chi-square (χ^2^) and the autocorrelation function of the residuals using the FAST (Advanced Fluorescence Lifetime Analysis Software) program provided by the equipment. To measure the photoluminescence quantum yield (QY) the FLS980 fluorescence spectrometer is equipped with an integrating sphere. A rectangular 10 mm cuvette was used for the fluorescence measurements and spectrophotometric grade toluene as the solvent. All data were measured at 25 °C. Cyclic voltammetry was carried out at room temperature using a PalmSens4 potentiostat, with a 0.10 M solution of tetrabutylammonium hexafluorophosphate (NBu_4_PF_6_) as the supporting electrolyte in DMF as the solvent at a scan rate of 100 mV/s in all of the experiments. The analyte concentration was 1 mM. Solutions were deaerated with a nitrogen stream prior to each measurement. Experiments were performed in a one-compartment cell equipped with a round glassy carbon electrode (diameter of 3 mm), a silver wire counter electrode, and a Ag/AgCl wire as pseudo-reference electrode. The working electrode was cleaned using mechanical polishing on a surface with a water-alumina slurry.^80^ All potentials were referenced against the ferrocene/ferrocenium couple (*F*_c_/*F*_c_^+^) after each experiment and plotted with IUPAC convention. Diffraction data were collected using an Oxford Diffraction Supernova diffractometer equipped with an Atlas CCD area detector and a four-circle κ goniometer. For the data collection, a Mo-microfocused source with multilayer optics were used. Data integration, scaling, and empirical absorption correction were performed using the CrysAlisPro software package and the structure was solved and refined with SHELX in OLEX2. Graphics were made with MERCURY.

#### 4,4,5,5-Tetramethyl-2-corannulyl-1,3,2-dioxaborolane (BpinCora)

1-Bromocorannulene (0.30 g, 0.91 mmol) was dissolved in dry THF (4.0 mL) under nitrogen atmosphere. BuLi (0.4 mL, 1.0 mmol) was added dropwise at −78 °C. After stirring for 10 min, a solution of 2-isopropoxy-4,4,5,5-tetramethyl-1,3,2-dioxaborolane (0.23 mL, 1.1 mmol) in dry THF (2.0 mL) was added at −78 °C. After 15 min stirring, the solution was warmed up to room temperature and stirred for another 30 min. The solution turned orange. It was stirred until there was no presence of starting material in the mixture, according to the ^1^H NMR spectrum of an aliquot. The solvent was removed under vacuum and the crude was dissolved in CH_2_Cl_2_ (10 mL) and washed with H_2_O (3 × 10 mL), dried with MgSO_4_, filtered, and the solvent was evaporated under vacuum to give the pure compound as a yellow solid (0.27 g, 79% yield). Spectral data were in agreement with those previously reported.^81^

#### 2,8-Dibromodibenzo[*b*,*d*]thiophene (**2-S**)

Compound **2-S** was prepared according to the reported procedure, and the spectral data were in agreement with those previously reported.^58^^1^H NMR (400 MHz, CDCl_3_) δ 8.24 (d, *J* = 1.9 Hz, 2H, H^3^), 7.71 (d, *J* = 8.5 Hz, 2H, H^6^), 7.58 (dd, *J* = 8.5, 1.9 Hz, 2H, H^5^). ^13^C{H} NMR (101 MHz, CDCl_3_) δ 138.6 (C^1^), 136.2 (C^2^), 130.3 (C^5^), 124.7 (C^3^), 124.2 (C^6^), 118.6 (C^4^).

#### 2,8-Dibromodibenzo[*b*,*d*]thiophene 5-oxide (**2-SO**)

Compound **2-SO** was prepared according to the reported procedure, and the spectral data were in agreement with those previously reported.^82^ A substantial fraction from the column appeared to be an intractable mixture of derivatives **2-S**, **2-SO**, and **2-SO**_**2**_. ^1^H NMR (500 MHz, CDCl_3_) δ 7.94 (d, *J* = 1.7 Hz, 2H, H^3^), 7.86 (d, *J* = 8.1 Hz, 2H, H^6^), 7.67 (dd, *J* = 8.1, 1.7 Hz, 2H, H^5^). ^13^C{H} NMR (101 MHz, CDCl_3_) δ 144.6 (C^1^), 137.9 (C^2^), 133.3 (C^5^), 129.1 (C^6^), 127.8 (C^4^), 125.6 (C^3^). HRMS (ESI-TOF): *m*/*z* = 378.8410 [M + Na]^+^ calculated 378.8398 for C_12_H_6_Br_2_NaOS.

#### 2,8-Dibromodibenzo[*b*,*d*]thiophene 5,5-dioxide (**2-SO**_**2**_)

Compound **2-SO**_**2**_ was prepared according to the reported procedure, and the spectral data were in agreement with those previously reported.^83^^1^H NMR (500 MHz, CDCl_3_) δ 7.93 (m, 2H, H^3^), 7.70 (m, 4H, H^5^, H^6^). ^13^C{H} NMR (101 MHz, CDCl_3_) δ 137.0 (Cq), 134.1 (C^5^), 132.4 (Cq), 129.0 (Cq), 125.3 (C^3^), 123.8 (C^4^). HRMS (ESI-TOF): *m*/*z* = 394.8357 [M + Na]^+^ calculated 394.8347 for C_12_H_6_Br_2_NaO_2_S.

#### 9-(*p*-Tolyl)-carbazole (**13**)

Compound **13** was synthesized following the literature protocol with modifications.^84^ Carbazole (**12**) (0.50 g, 3.0 mmol), 4-bromotoluene (1.5 mL, 12 mmol), NaH (0.10 g, 4.5 mmol), 18-crown-6 (0.13 g, 0.50 mmol), and CuI (76 mg, 0.40 mmol) were refluxed in an oil bath under inert atmosphere in dichlorobenzene (3.0 mL) at 180 °C for 35 h. Then, it was cooled to room temperature and quenched by the addition of an aqueous saturated solution of NH_4_Cl (5.0 mL). The crude was extracted with CH_2_Cl_2_ (10 mL) and H_2_O (3 × 10 mL) dried with MgSO_4_, filtered, and concentrated under vacuum before subjecting the resulting crude to purification by column chromatography on silica gel (10:1 *n*-hexane/AcOEt) to give the pure compound as a white solid (0.45 g, 59% yield). Spectral data were in agreement with those previously reported.^85^

#### 3,6-Dibromo-9-(*p*-tolyl)-carbazole (**14**)

Compound **13** (0.45 g, 1.7 mmol) and *N*-bromosuccinimide (0.94 g, 5.3 mmol) were stirred in 15 mL of CHCl_3_ and 3.0 mL of acetic acid in the dark for 16 h. The solvent was removed under vacuum and then dissolved in CH_2_Cl_2_ (15 mL) and extracted with an aqueous saturated solution of NaHCO_3_ (3 × 10 mL), dried with MgSO_4_, filtered, and concentrated under vacuum. Pure compound was obtained as a white solid (0.18 g, 25% yield). ^1^H NMR spectral data were in agreement with those previously reported.^86^^1^H NMR (500 MHz, CDCl_3_) δ 8.19 (d, *J* = 1.9 Hz, 2H, H^3^), 7.49 (dd, *J* = 8.7, 1.9 Hz, 2H, H^5^), 7.40 (d, *J* = 8.1 Hz, 2H, H^9^), 7.36 (d, *J* = 8.1 Hz, 2H, H^8^), 7.22 (d, *J* = 8.7 Hz, 2H, H^6^), 2.49 (s, 3H H^11^). ^13^C{H} NMR (101 MHz, CDCl_3_) δ 140.2 (C^1^), 138.3 (C^10^), 134.2 (C^7^), 130.8 (C^9^), 129.5 (C^5^), 127.0 (C^8^), 124.0 (C^2^), 123.3 (C^3^), 113.0 (C^4^), 111.7 (C^6^), 21.4 (C^11^). HRMS (MALDI-TOF): *m*/*z* = 412.9410 [M]^+^ calculated 412.9409 for C_19_H_13_Br_2_N.

### General Procedure for Miyaura Reaction

Aryl halide (1 equiv, 0.50 mmol), bis(pinacolato)diboron (B_2_(pin)_2_) (3 equiv), [PdCl_2_(dppf)] (5–10%), and potassium acetate (6 equiv) were mixed in a microwave flask under inert atmosphere. Dry dioxane was added so that the concentration of the aryl halide was 0.10 M, and the mixture was degassed. It was then irradiated in a microwave reactor at 170 °C for 30–45 min with stirring at 600 rpm. The solvent was removed before further purification.

#### 2,8-bis(4,4,5,5-Tetramethyl-1,3,2-dioxaborolan-2-yl)dibenzo[*b*,*d*]thiophene (**3-S**)

Purification by flash column chromatography on silica gel (2:1 *n*-hexane/AcOEt) and subsequent precipitation in CHCl_3_/CH_3_OH (2.0 mL/5.0 mL). The precipitate was centrifuged and dried giving the expected compound as a white solid (0.14 g, 65% yield). ^1^H NMR (400 MHz, CDCl_3_) δ 8.72 (dd, *J* = 1.1, 0.8 Hz, 2H, H^3^), 7.88 (dd, *J* = 8.0, 1.1 Hz, 2H, H^5^), 7.85 (dd, *J* = 8.0, 0.8 Hz, 2H, H^6^), 1.41 (s, 24H, H^7^). ^13^C{H} NMR (101 MHz, CDCl_3_) δ 142.6 (C^1^), 135.3 (C^2^), 132.7 (C^5^), 128.7 (C^3^), 122.2 (C^6^), 84.1 (C^8^), 25.1 (C^7^). HRMS (ESI-TOF): *m*/*z* = 459.1961 [M + Na]^+^ calculated 459.1952 for C_24_H_30_B_2_NaO_4_S.

#### 2,8-bis(4,4,5,5-Tetramethyl-1,3,2-dioxaborolan-2-yl)dibenzo[*b*,*d*]thiophene 5,5-dioxide (**3-SO**_**2**_)

Purification by extraction of the crude with CH_2_Cl_2_ (15 mL) followed by H_2_O washes (3 × 15 mL). The organic phase was dried with MgSO_4_, filtered, and concentrated under vacuum. A mixture of CH_2_Cl_2_ (3.0 mL) and *n*-hexane (10 mL) was needed to afford the precipitation of a white solid, which was separated by centrifugation and washed with *n*-hexane (5.0 mL). The solid was finally dried to obtain the pure compound as a white solid (0.13 g, 55% yield). ^1^H NMR (500 MHz, CDCl_3_) δ 8.29 (dd, *J* = 0.9, 0.7 Hz, 2H, H^3^), 7.95 (dd, *J* = 7.6, 0.9 Hz, 2H, H^5^), 7.80 (dd, *J* = 7.6, 0.7 Hz, 2H, H^6^), 1.39 (s, 24H, H^7^). ^13^C{H} NMR (101 MHz, CDCl_3_) δ 140.0 (C^1^), 136.8 (C^5^), 131.0 (C^2^), 128.0 (C^3^), 121.4 (C^6^), 84.8 (C^8^), 25.1 (C^7^). HRMS (ESI-TOF): *m*/*z* = 491.1859 [M + Na]^+^ calculated 491.1850 for C_24_H_30_B_2_NaO_6_S.

#### 2,2′-(9,9-Dimethyl-fluorene-3,6-diyl)bis(4,4,5,5-tetramethyl-1,3,2-dioxaborolane) (**10-CMe**_**2**_)

Purification by column chromatography on silica gel (10:1 *n*-hexane/AcOEt) to give the expected compound as a white solid (0.16 g, 0. 72% yield). ^1^H NMR (500 MHz, CDCl_3_) δ 8.28 (s, 2H, H^3^), 7.78 (d, *J* = 7.5 Hz, 2H, H^5^), 7.45 (d, *J* = 7.5 Hz, 2H, H^3^), 1.48 (s, 6H, H^7^), 1.37 (s, 24H, H^9^). ^13^C{H} NMR (101 MHz, CDCl_3_) δ 156.9 (C^1^), 138.8 (C^2^), 134.0 (C^5^), 128.0 (C^4^), 126.9 (C^3^), 122.1 (C^6^), 83.9, (C^9^) 47.4 (C^8^), 27.1 (C^7^), 25.1 (C^9^). HRMS (MALDI-TOF): *m*/*z* = 446.2804 [M]^+^ calculated 446.2804 for C_27_H_36_B_2_O_4_.

#### 3,6-bis(4,4,5,5-Tetramethyl-1,3,2-dioxaborolan-2-yl)-fluoren-9-one (**10-CO**)

Purification by column chromatography on silica gel (10:1 to 5:1 *n*-hexane/AcOEt) to give the expected compound as a white solid (0.12 g, 58% yield). ^1^H NMR (400 MHz, CDCl_3_) δ 8.01 (s, 2H, H^3^), 7.74 (d, *J* = 7.3 Hz, 2H, H^5^), 7.64 (d, *J* = 7.3 Hz, 2H, H^6^), 1.37 (s, 24H, H^7^). ^13^C{H} NMR (101 MHz, CDCl_3_) δ 194.6 (C^9^), 143.9 (C^2^), 136.5 (C^1^), 135.9 (C^5^), 126.6 (C^3^), 123.5 (C^6^), 84.4 (C^8^), 25.1 (C^7^). HRMS (MALDI-TOF): *m*/*z* = 432.2324 [M]^+^ calculated 432.2283 for C_25_H_30_B_2_O_5_.

#### 3,6-bis(4,4,5,5-Tetramethyl-1,3,2-dioxaborolan-2-yl)-9-(*p*-tolyl)-carbazole (**15**)

Purification by column chromatography on silica gel (10:1 *n*-hexane/AcOEt) to give the expected compound as a white solid (0.11 g, 43% yield). ^1^H NMR (500 MHz, CDCl_3_) δ 8.70 (dd *J* = 1.2, 0.7 Hz, 2H, H^3^), 7.83 (dd, *J* = 8.2, 1.2 Hz, 2H, H^5^), 7.41 (s, 4H, H^8^, H^9^), 7.33 (dd, *J* = 8.2, 0.7 Hz, 2H, H^6^), 2.49 (s, 3H, H^11^), 1.39 (s, 24H, H^12^). ^13^C{H} NMR (101 MHz, CDCl_3_) δ 143.4 (C^1^), 137.8 (C^7^ or C^10^), 134.8 (C^7^ or C^10^), 132.4 (C^5^), 130.7 (C^9^), 128.2 (C^3^), 127.1 (C^8^), 123.3 (C^2^), 119.8 (C^4^, indirectly detected), 109.3 (C^16^), 83.7 (C^13^), 25.1 (C^12^), 21.4 (C^11^). HRMS (MALDI-TOF): *m*/*z* = 509.2919 [M]^+^ calculated 509.2914 for C_31_H_37_B_2_NO_4_.

### General Procedure for Suzuki Coupling

Diboronate (1 equiv, 50 μmol), 1-bromocorannulene (2.1 equiv), [PdCl_2_(dppf)] (10%), and *^t^*BuONa (6 equiv) were mixed in a microwave flask under inert atmosphere. Dry and degassed toluene (3.0 mL) was then added. The solution was irradiated in a microwave reactor at 130 °C for 30 min with stirring at 600 rpm. The solvent was removed under vacuum before subjecting the resulting crude to a further purification.

#### 2,8-Dicorannulyldibenzo[*b*,*d*]thiophene (**4-S**)

The crude was diluted in CH_2_Cl_2_ (40 mL) and filtered over kieselguhr. The solvent was concentrated under vacuum until 1 mL was left and *n*-hexane was added (10 mL) to obtain a yellow precipitate. The solvent was removed by centrifugation. The pure compound was isolated as a pale-yellow solid after drying (18 mg, 52% yield).

Alternatively, this compound can be prepared as follows: a mixture of 2,2′-bis(methylthio)-5,5′-bis(corannulyl)-1,1′-biphenyl,^20^ (25 mg, 30 μmol), *^t^*BuSNa (0.38 g, 0.34 mmol) in dry DMF (0.50 mL) was heated in an oil bath under inert atmosphere at 160 °C for 1 h. The reaction color turned red after 1 h and was allowed to cool down to room temperature. HCl (35%) (1.0 mL) was added to the mixture to quench the reaction and a pale-yellow solid appeared. The solid was separated by centrifugation. Subsequently, it was dissolved in 10 mL of CH_2_Cl_2_ and washed with H_2_O (3 × 10 mL). The organic phase was dried with MgSO_4_, filtered, and concentrated at reduced pressure until 1.0 mL of CH_2_Cl_2_ was left. An addition of 3.0 mL of *n*-hexane was needed to afford precipitation. The resulting solid was then centrifuged to obtain the pure expected compound as a pale-yellow solid after drying (14 mg, 61% yield).

^1^H NMR (500 MHz, CDCl_3_) δ 8.62 (dd, *J* = 1.8, 0.6 Hz, 2H, H^3^), 8.10 (dd, *J* = 8.3, 0.6 Hz, 2H, H^6^), 8.01 (s, 2H, H^8^), 7.96 (dd, *J* = 8.3, 1.8 Hz, 2H, H^5^), 7.88 (d, *J* = 8.7 Hz, 2H, H^9^), 7.85 (d, *J* = 8.7 Hz, 2H, H^10^), 7.85 (d, *J* = 8.8 Hz, 2H, H^16^) 7.84–7.81 (m, 6H, H^11^, H^13^ and H^12^), 7.79 (d, *J* = 8.8 Hz, 2H, H^14^), 7.77 (d, *J* = 8.8 Hz, 2H, H^15^). ^13^C{H} NMR (101 MHz, CDCl_3_) δ 141.5 (C^7^), 139.5 (C^1^), 136.45 (C^4^), 136.40 (C^23^), 136.2 (C^26^ or C^25^), 136.1 (C^2^), 135.9 (C^22^), 135.4 (C^25^ or C^26^), 135.1 (C24), 131.0 (C^20^), 130.90 (C^21^ or C^18^), 130.89 (C^21^ or C^18^), 130.8 (C^19^), 129.8 (C^17^), 129.0 (C^5^), 127.6 (C^15^), 127.4 (C^16^), 127.3 (C^12^), 127.1 (C^11^), 127.02 (C^9^), 127.00 (C^10^), 127.97 (C^13^), 126.91 (C^14^), 126.1 (C^8^), 123.2 (C^6^), 123.0 (C^3^). HRMS (MALDI): *m*/*z* = 680.1614 [M]^+^ calculated 680.1593 for C_52_H_24_S.

#### 2,8-Dicorannulyldibenzo[*b*,*d*]thiophene 5,5-dioxide (**4-SO**_**2**_)

Purification by column chromatography on silica gel (3:2 *n*-hexane/AcOEt) to give the pure compound as a pale-yellow solid (21 mg, 60% yield).

Alternatively, this compound can be prepared as follows: Host **4-S** (50 mg, 60 μmol), acetic acid (2.0 mL), and H_2_O_2_ (0.50 mL, 30%) were placed in a round-bottom flask and stirred at 120 °C using an oil bath for 15 min. After that period, 0.50 mL of H_2_O_2_ was added and the mixture was refluxed for another 15 min. Then, it was cooled down to room temperature and a yellowish solid appeared. It was filtered and washed with H_2_O and CH_3_OH and dried. The resulting crude was subjected to purification by column chromatography on silica gel (3:2 *n*-hexane/AcOEt) to give the pure compound as a pale-yellow solid (21 mg, 50% yield).

^1^H NMR (500 MHz, CDCl_3_) δ 8.25 (dd, *J* = 1.5, 0.6 Hz, 2H, H^3^), 8.07 (dd, *J* = 7.9, 0.6 Hz, 2H, H^6^), 7.98 (dd, *J* = 7.9, 1.5 Hz, 2H, H^5^), 7.96 (s, 2H, H^8^), 7.86 (s, 4H, H^10^, H^9^), 7.84 (d, *J* = 8.7 Hz, 2H, H^13^), 7.83 (s, 4H, H^11^, H^12^), 7.82–7.78 (m, 4H, H^15^, H^14^), 7.75 (d, *J* = 8.8 Hz, 2H, H^16^). ^13^C{H} NMR (126 MHz, CDCl_3_) δ 146.2 (C^4^), 139.3 (C^7^), 137.4 (C^1^), 136.45 (C^26^), 136.42 (C^23^), 136.0 (C^24^), 135.95 (C^22^), 135.5 (C^25^), 132.37 (C^5^), 132.34 (C^2^), 131.29 (C^19^ or C^20^ or C^21^), 131.27 (C^19^ or C^20^ or C^21^), 131.21 (C^19^ or C^20^ or C^21^), 130.6 (C^18^), 128.9 (C^17^), 128.2 (C^15^), 127.9 (C^16^), 127.7 (C^13^), 127.6 (C^12^), 127.4 (C^11^), 127.2 (C^14^), 127.1 (C^9^), 127.0 (C^8^), 126.2 (C^16^), 123.2 (C^3^), 122.8 (C^6^). HRMS (ESI-TOF): *m*/*z* = 735.1395 [M + Na]^+^ calculated 735.1389 for C_52_H_24_NaO_2_S.

#### 3,6-Dicorannulyl-9,9-dimethyl-fluorene (**11-CMe**_**2**_)

Purification by column chromatography on silica gel (5:1 *n*-hexane/AcOEt) to give the pure compound as a yellow solid (16 mg, 46% yield). ^1^H NMR (500 MHz, CDCl_3_) δ 8.19 (dd, *J* = 1.6, 0.6 Hz, 2H, H^3^), 7.97 (s, 2H, H^8^), 7.91 (d, *J* = 8.9 Hz, 2H, H^16^), 7.87 (d, *J* = 8.7 Hz, 2H, H^9^), 7.85 (d, *J* = 8.7 Hz, 2H, H^10^), 7.84–7.81 (m, 6H, H^11^, H^13^, H^12^), 7.81–7.76 (m, 6H, H^14^, H^5^, H^15^), 7.67 (dd, *J* = 7.8, 0.6 Hz, 2H, H^6^), 1.71 (s, 6H, H^27^). ^13^C{H} NMR (126 MHz, CDCl_3_) δ 153.7 (C^n^), 142.2 (C^7^), 139.8 (C^2^), 139.0 (C^4^), 136.5 (C^23^), 136.4 (C^26^), 136.0 (C^22^), 135.6 (C^25^), 135.5 (C^24^), 131.12 (C_q_), 131.08 (C_q_), 131.0 (C^18^), 130.9 (C_q_), 130.0 (C^17^), 129.7 (C^5^), 127.54 (C^15^), 127.50 (C^10^), 127.4 (C^12^ and C^16^), 127.3 (C^11^), 127.2 (C^9^), 127.11 (C^13^), 127.08 (C^14^), 126.0 (C^8^), 123.1 (C^6^), 121.7 (C^3^), 47.0 (C^28^), 27.5 (C^1^). HRMS (MALDI-TOF): *m*/*z* = 690.2361 [M]^+^ calculated 690.2342 for C_55_H_30_.

#### 3,6-Dicorannulyl-fluoren-9-one (**11-CO**)

The crude was diluted in CH_2_Cl_2_ (20 mL) and filtered over kieselguhr. The solvent was concentrated under vacuum until 1 mL was left and *n*-hexane was added (10 mL) to obtain a yellow precipitate. The solvent was removed by centrifugation. The pure compound was isolated as a bright yellow solid after drying (17 mg, 50% yield). ^1^H NMR (500 MHz, CDCl_3_) δ 8.04 (dd, *J* = 1.6, 0.6 Hz, 2H, H^3^), 8.00 (s, 2H, H^8^), 7.92 (dd, *J* = 7.6, 0.6 Hz, 2H, H^6^), 7.87 (m, 4H, H^9^, H^10^), 7.86–7.83 (m, 8H, H^16^, H^13^, H^11^, H^12^), 7.82–7.80 (m, 4H, H^14^, H^15^), 7.78 (dd, *J* = 7.6, 1.6 Hz, 2H, H^5^). ^13^C{H} NMR (101 MHz, CDCl_3_) δ 193.3 (C^27^), 146.7 (C^4^), 145.0 (C^2^), 140.7 (C^1^), 136.5 (C^23^), 136.4 (C^26^), 136.0 (C^22^), 135.9 (C^24^), 135.5 (C^24^), 134.1 (C^7^), 131.25 (C^5^), 131.23 (C^20^ or C^21^ or C^19^), 131.17 (C^20^ or C^21^ or C^19^), 130.7 (C^18^), 129.1 (C^17^), 127.9 (C^15^), 127.8 (C^10^), 127.6 (C^13^), 127.44 (C^12^ or C^11^), 127.37 (C^12^ or C^11^), 127.14 (C^14^), 127.11 (C^9^), 126.7 (C^16^), 126.5 (C^8^), 124.9 (C^6^), 122.2 (C^3^). HRMS (MALDI-TOF): *m*/*z* = 676.1814 [M]^+^ calculated 676.1822 for C_53_H_24_O.

#### 3,6-Dicorannulyl-9-(*p*-tolyl)-carbazole (**16**)

Purification by column chromatography on silica gel (5:1 *n*-hexane/AcOEt) to give the pure compound as a yellow solid (19 mg, 50% yield). ^1^H NMR (500 MHz, CDCl_3_) δ 8.61 (dd, *J* = 1.8, 0.6 Hz, 2H, H^3^), 8.02 (s, 2H, H^8^), 7.92 (m, 4H, H^5^, H^16^), 7.89 (d, *J* = 8.8 Hz, 2H, H^9^), 7.85 (d, *J* = 8.8 Hz, 2H, H^10^), 7.84–7.80 (m, 8H, H^11^, H^13^, H^12^, H^14^), 7.78 (d, *J* = 8.9 Hz, 2H, H^15^), 7.67–7.57 (m, 4H, H^28^, H^6^), 7.53–7.48 (m, 2H, H^29^), 2.55 (s, 3H, H^31^). ^13^C{H} NMR (126 MHz, CDCl_3_) δ 142.7 (C^7^), 141.4 (C^1^), 138.0 (C^30^), 136.7 (C^23^), 136.4 (C^26^), 136.1 (C^22^), 135.6 (C^25^), 135.3 (C^24^), 135.0 (C^27^), 132.0 (C^4^), 131.2 (C^18^), 131.1 (C^19^ or C^20^), 131.0 (C^21^), 130.85 (C^29^), 130.77 (C^19^ or C^20^), 130.6 (C^17^), 128.7 (C^5^), 127.6 (C^16^), 127.52 (C^15^), 127.48 (C^10^), 127.4 (C^13^), 127.24 (C^11^), 127.22 (C^9^), 127.1 (C^28^), 127.05 (C^14^), 127.00 (C^12^), 125.7 (C^8^), 124.0 (C^2^), 121.9 (C^3^), 110.4 (C^6^), 21.5 (C^31^). HRMS (MALDI-TOF): *m*/*z* = 753.2451 [M]^+^ calculated 753.2451 for C_59_H_31_N.

#### 2,8-Dicorannulyldibenzo[*b*,*d*]thiophene 5-Oxide (**4-SO**)

The preparation of this compound has been carried out by swapping the functionalization pattern of both reactants. Compound **2-SO** (24 mg, 70 μmol), Bpin-corannulene (50 mg, 0.13 mmol), [PdCl_2_(dppf)] (4.8 mg, 7.0 μmol), and *^t^*BuONa (38 mg, 0.39 mmol) were mixed in a microwave flask under inert atmosphere. Dry and degassed toluene (3.0 mL) was then added. The solution was irradiated in a microwave reactor at 130 °C for 30 min with stirring at 600 rpm. The solvent was removed under vacuum before subjecting the resulting crude to a purification by column chromatography on silica gel (5:1 to 2:1 to 1:1 *n*-hexane/AcOEt) to give the pure compound as a pale-yellow solid (12 mg, 27% yield).

Alternatively, this compound can be prepared as follows: Host **4-S** (40 mg, 60 μmol) and *m*-CPBA (12 mg, 70 μmol) were dissolved in CH_2_Cl_2_ (15 mL) in a round-bottom flask. The solution was stirred for 1 h. The solvent was evaporated, and the resulting crude was purified by column chromatography on silica gel (5:1 to 2:1 to 1:1 *n*-hexane/AcOEt) to give the expected molecule as a pale-yellow solid (9 mg, 20% yield).

^1^H NMR (500 MHz, CDCl_3_) δ 8.28 (d, *J* = 1.0 Hz, 2H, H^3^), 8.24 (d, *J* = 7.9 Hz, 2H, H^6^), 7.98 (s, 2H, H^8^), 7.96 (dd, *J* = 7.9, 1.0 Hz, 2H, H^5^), 7.87 (m, 4H, H^9^, H^10^), 7.84 (d, *J* = 8.7 Hz, 2H, H^13^), 7.83 (s, 4H, H^11^, H^12^), 7.82–7.79 (m, 4H, H^14^, H^15^), 7.77 (d, *J* = 8.8 Hz, 2H, H^16^). ^13^C{H} NMR (101 MHz, CDCl_3_) δ 145.0 (C^1^), 144.8 (C^4^), 140.0 (C^7^), 137.9 (C^2^), 136.4 (C^23^ or C^26^), 136.0 (C^22^), 135.9 (C^24^), 135.5 (C^25^), 131.6 (C^5^), 131.25 (C^21^ or C^20^ or C^19^), 131.22 (C^21^ or C^20^ or C^19^), 131.18 (C^21^ or C^20^ or C^19^), 130.7 (C^18^), 129.1 (C^17^), 128.11 (C^6^), 128.09 (C^15^), 127.8 (C^10^), 127.7 (C^13^), 127.5 (C^12^), 127.4 (C^11^), 127.14 (C^14^), 127.09 (C^9^), 126.8 (C^8^), 126.4 (C^16^), 123.6 (C^3^). HRMS (MALDI-TOF): *m*/*z* = 697.1615 [M]^+^ calculated 697.1621 for C_52_H_24_OS.

#### 2-(3,6-Dicorannulyl-fluoren-9-ylidene)malononitrile (**11-C(CN)**_**2**_)

Compound **11-CO** (25 mg, 37 μmol) and freshly crystallized malonitrile (10 mg, 0.37 mmol) were dissolved in dry pyridine (1.0 mL) under inert atmosphere becoming orange in color. It was heated in an oil bath at 80 °C under inert atmosphere for 30 min. After such a time, EtOH (10 mL) was added, and a red precipitate appeared. The solvent was removed by centrifugation, and the solid was washed with EtOH (3 × 10 mL). A dark red solid was obtained (17 mg, 63% yield). ^1^H NMR (500 MHz, CDCl_3_) δ 8.63 (d, *J* = 8.2 Hz, 2H, H^6^), 8.09 (d, *J* = 1.2 Hz, 2H, H^3^), 8.01 (s, 2H, H^8^), 7.87 (s, 4H, H^9^, H^10^), 7.85–7.79 (m, 12H, H^11^, H^12^, H^13^, H^14^, H^15^, H^16^), 7.80 (dd, *J* = 8.2, 1.2 Hz, 2H, H^5^). ^13^C{H} NMR (126 MHz, CDCl_3_) δ 161.0 (C^27^), 146.4 (C^4^), 142.9 (C^2^), 140.0 (C^1^), 136.50 (C^23^), 136.47 (C^22^), 136.04 (C^24^), 135.96 (C^26^), 135.5 (C^25^), 134.1 (C^7^), 131.3 (C^19^ or C^20^ or C^21^), 131.27 (C^19^ or C^20^ or C^21^), 131.24 (C^19^ or C^20^ or C^21^), 131.1 (C^5^), 130.6 (C^18^), 128.8 (C^17^), 128.1 (C^12^), 127.9 (C^10^), 127.7 (CHCora), 127.55 (CHCora), 127.48 (C^6^), 127.4 (C^11^), 127.14 (CHCora) 127.07 (C^9^), 126.7 (C^8^), 126.5 (C^16^), 122.4 (C^3^), 113.8 (C^29^). HRMS (MALDI-TOF): *m*/*z* = 724.1914 [M]^+^ calculated 724.1934 for C_56_H_24_N_2_.

## Data Availability

The data underlying this study are available in the published article and its online supplementary material.

## Supporting Information Available

Full characterization by 1D and 2D NMR along with HRMS spectra; UV–vis and emission experiments details including decay lifetimes; quantum yields and solvatochromism measurements; cyclic voltammetry experiments; HOMO/LUMO levels estimation; single-crystal X-ray diffraction analysis; full disclosure of association constant measurements; Hammett parameters correlation; emission quenching experiments upon addition of C_70_; and a comprehensive description of all computational calculations (PDF)
