# Supplementary material for: From Induced-Fit Assemblies to Ternary Inclusion Complexes with Fullerenes in Corannulene-Based Molecular Tweezers
Source: J Org Chem. 2022 Dec 1;87(24):16691–706. doi: 10.1021/acs.joc.2c02345 (PMC9764357; doi:10.1021/acs.joc.2c02345)
Supplement: Supplementary file 1 — jo2c02345_si_001.pdf [file jo2c02345_si_001.pdf]

SUPPORTING INFORMATION

From Induced-Fit Assemblies to Ternary Inclusion Complexes with Fullerenes in Corannulene-Based Molecular Tweezers

Adriana Sacristán-Martín, Daniel Miguel, Alberto Diez-Varga, Héctor Barbero* and Celedonio M. Álvarez*

Table of Contents

NMR and HR-MS spectra S2

UV-Vis absorption and emission spectra S66

Fluorescence decay lifetimes and Quantum Yields S69

Cyclic Voltammograms S69

X-ray Crystallographic Tables S71

Association constants measurements S73

Quenching Experiments upon fullerene binding S100

Computational Calculations details S102

References S122

NMR and HR-MS spectra

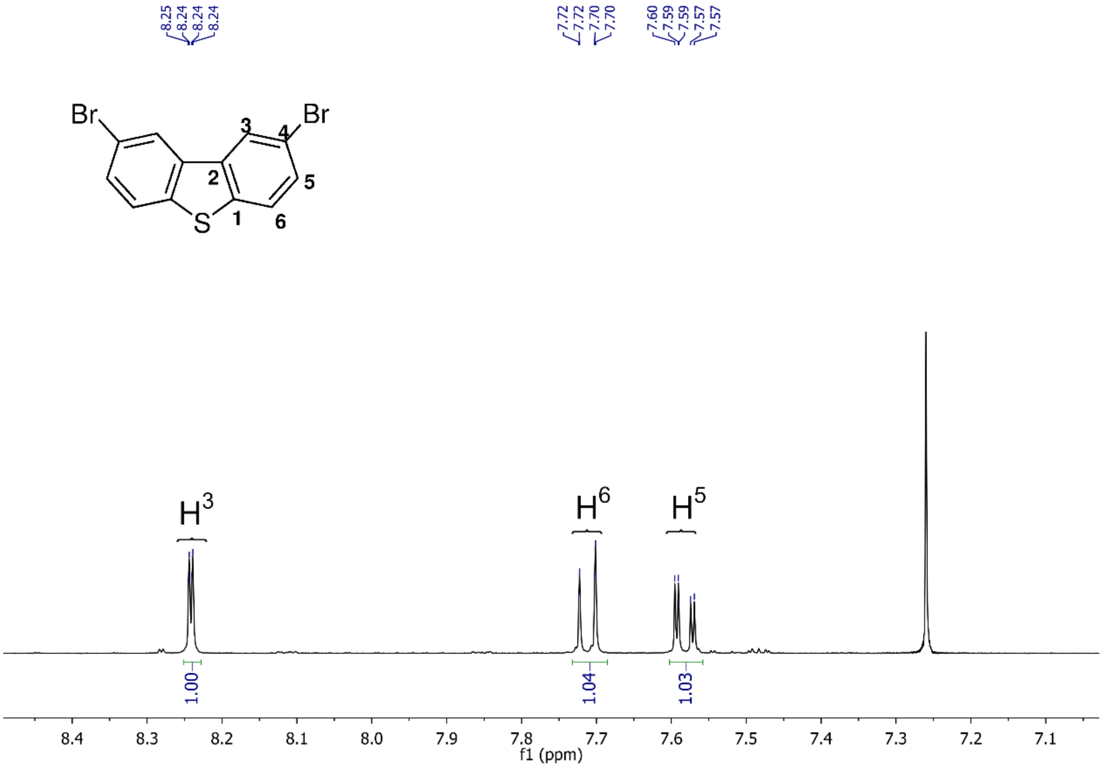

Figure S 1. ^{1}H-NMR (400 MHz, $CDCl_3$) spectrum of compound **2-S**.

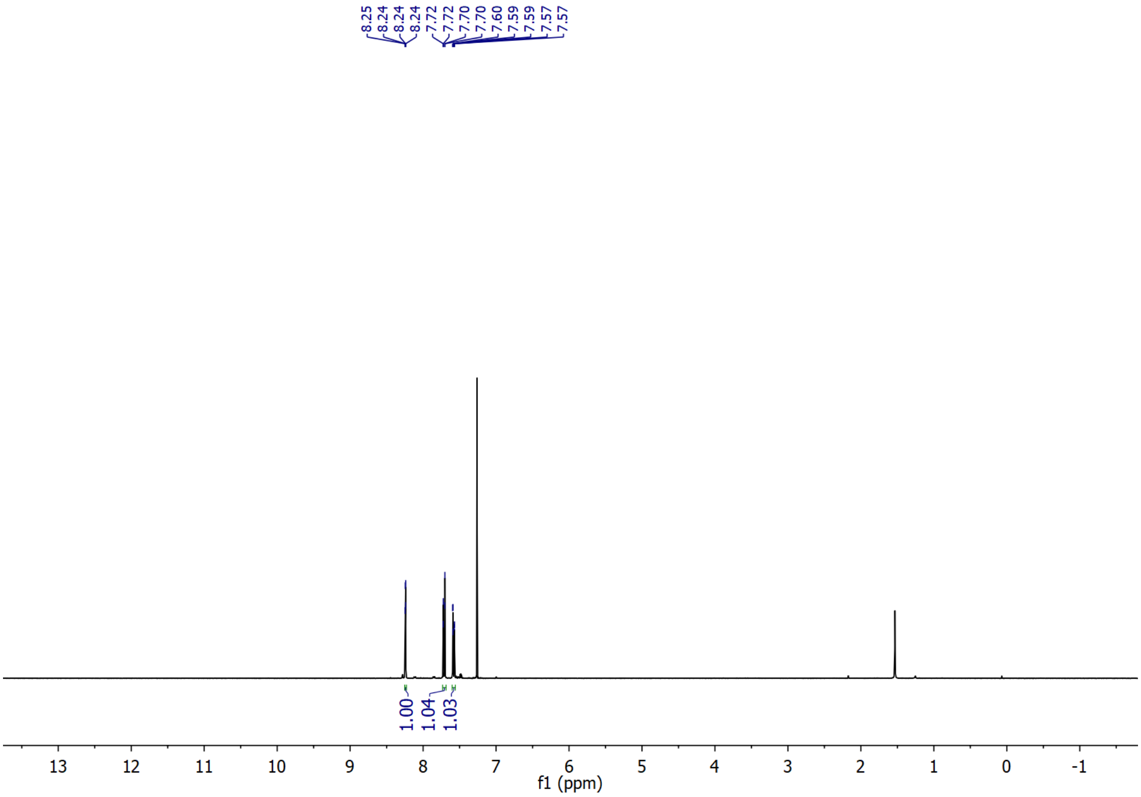

Figure S 2. Full ^{1}H-NMR (400 MHz, $CDCl_3$) spectrum of compound **2-S**.

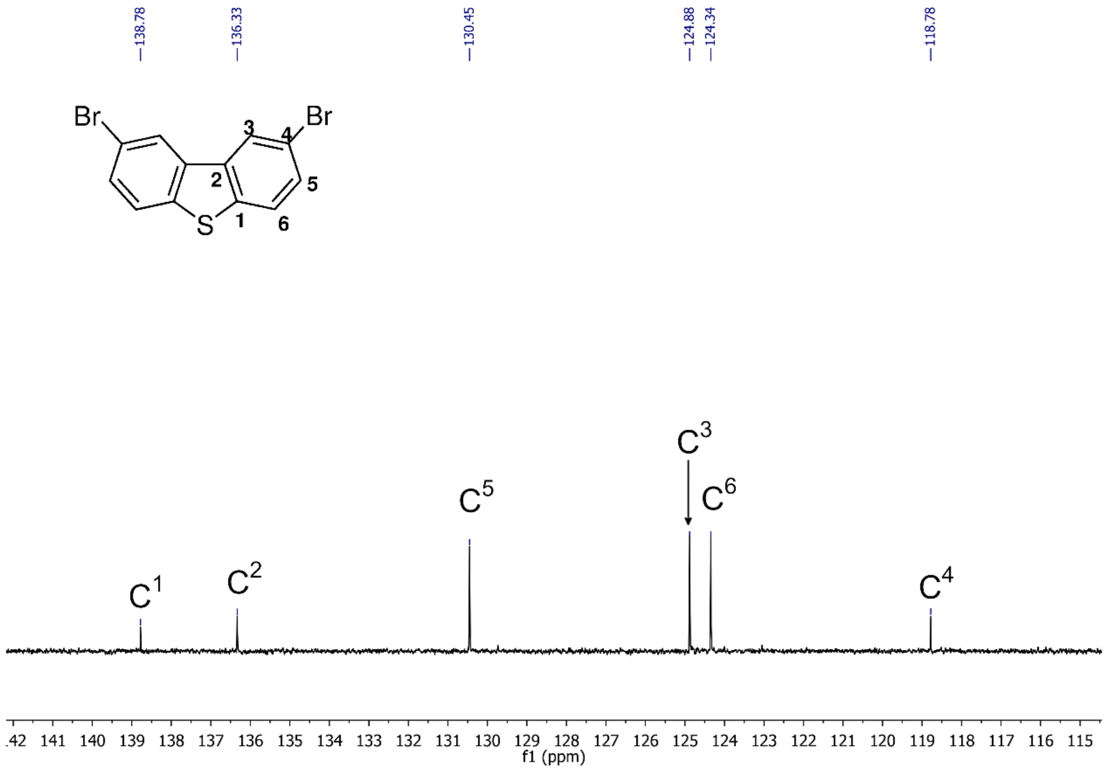

Figure S 3. $^{13}C\{^{1}H\}$-NMR (101 MHz, $CDCl_3$) spectrum of compound **2-S**.

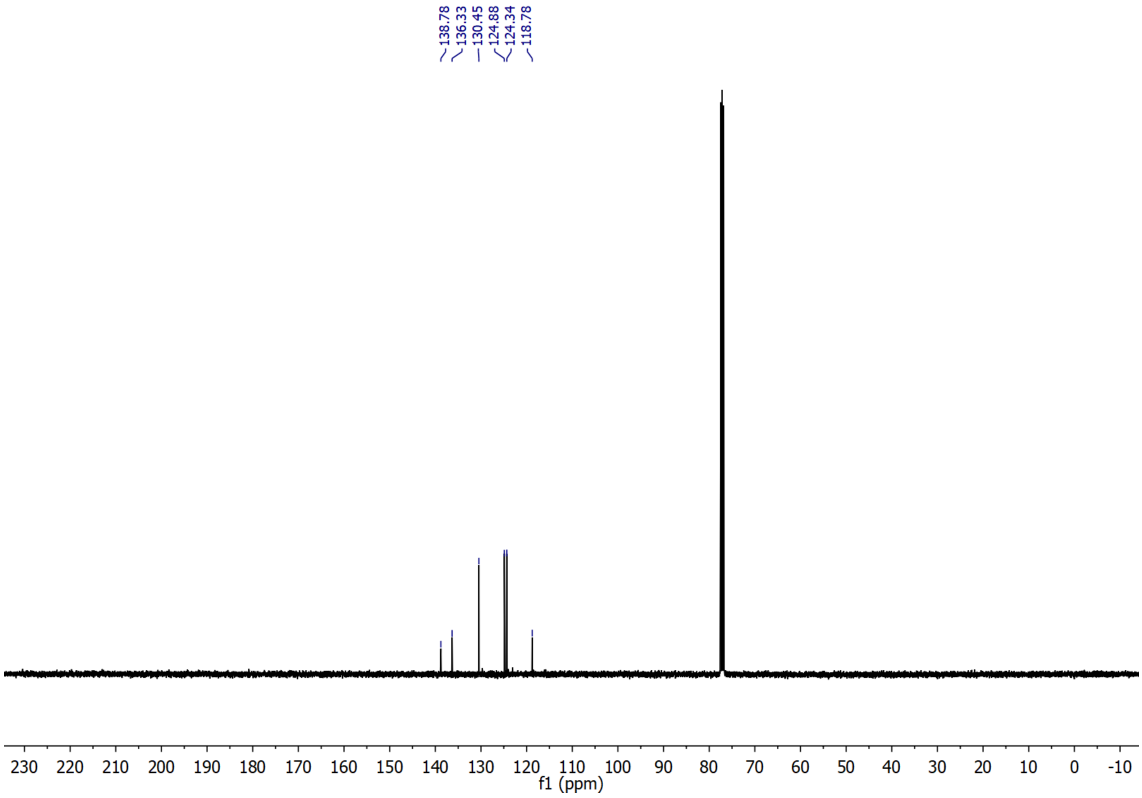

Figure S 4. Full $^{13}C\{^{1}H\}$-NMR (101 MHz, $CDCl_3$) spectrum of compound **2-S**.

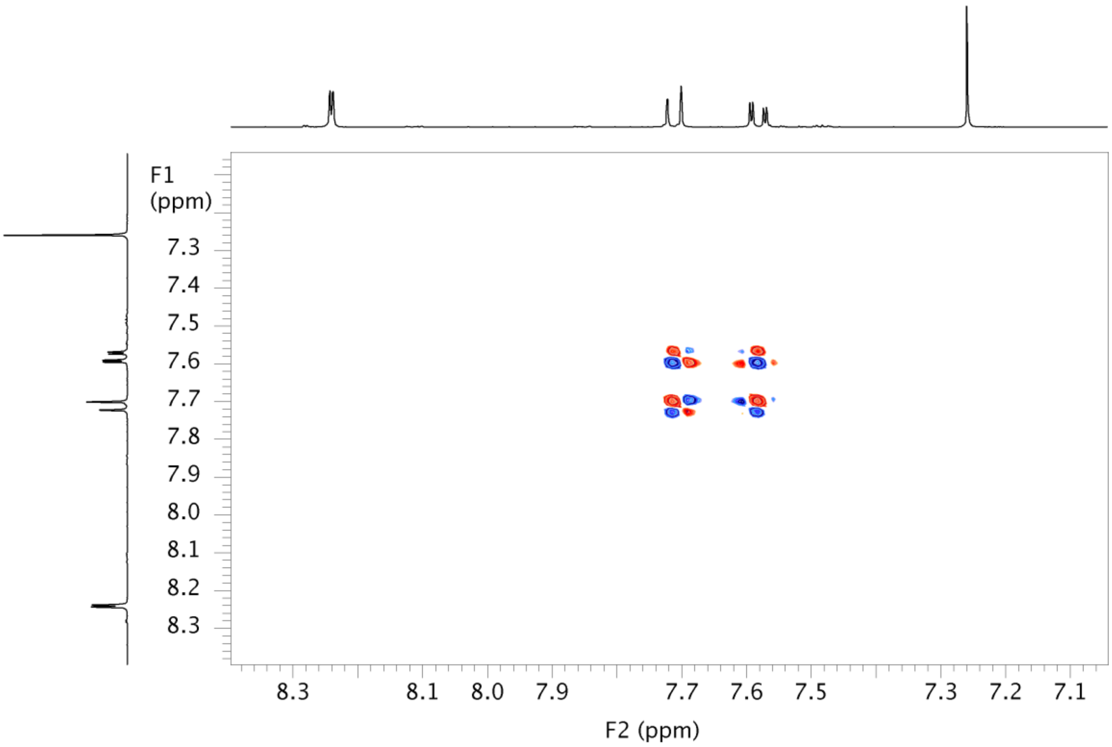

Figure S 5. ^{1}H-^{1}H gDQFCOSY (400 MHz, $CDCl_3$) spectrum of compound **2-S**.

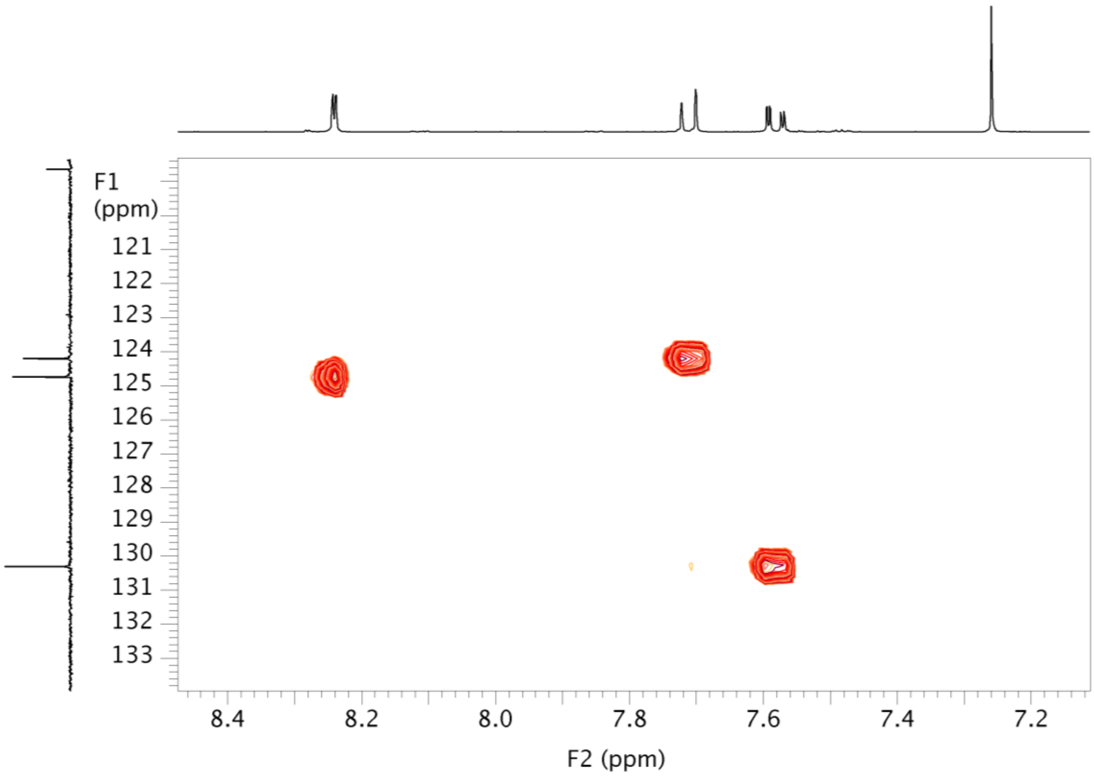

Figure S 6. ^{1}H-^{13}C gc2HSQC (400 MHz, $CDCl_3$) spectrum of compound **2-S**.

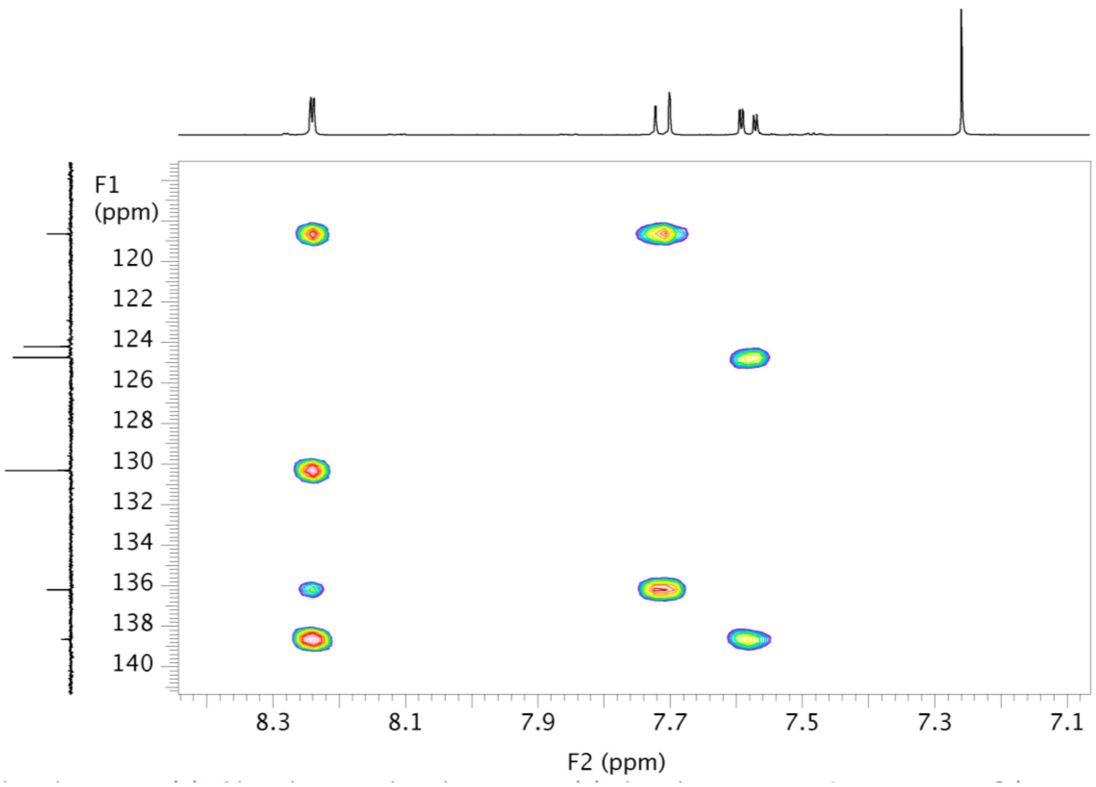

Figure S 7. ^{1}H-^{13}C gc2HMBC (400 MHz, $CDCl_3$) spectrum of compound **2-S**.

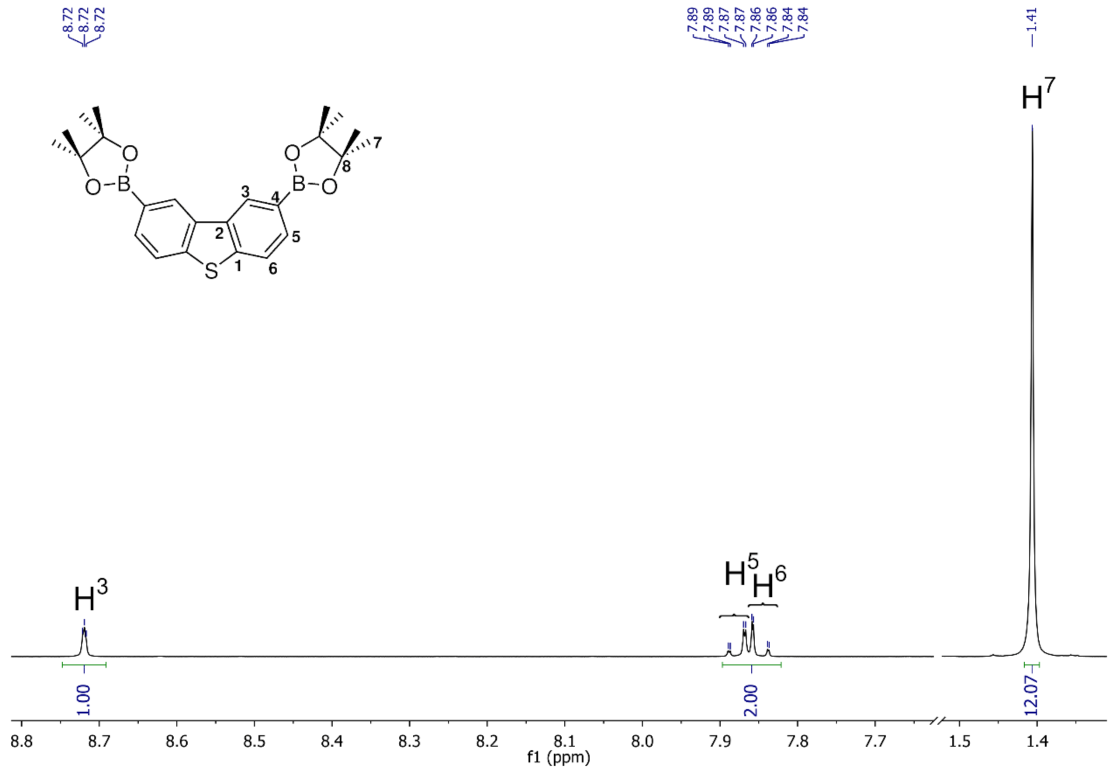

Figure S 8. ^{1}H-NMR (400 MHz, $CDCl_3$) spectrum of compound **3-S**.

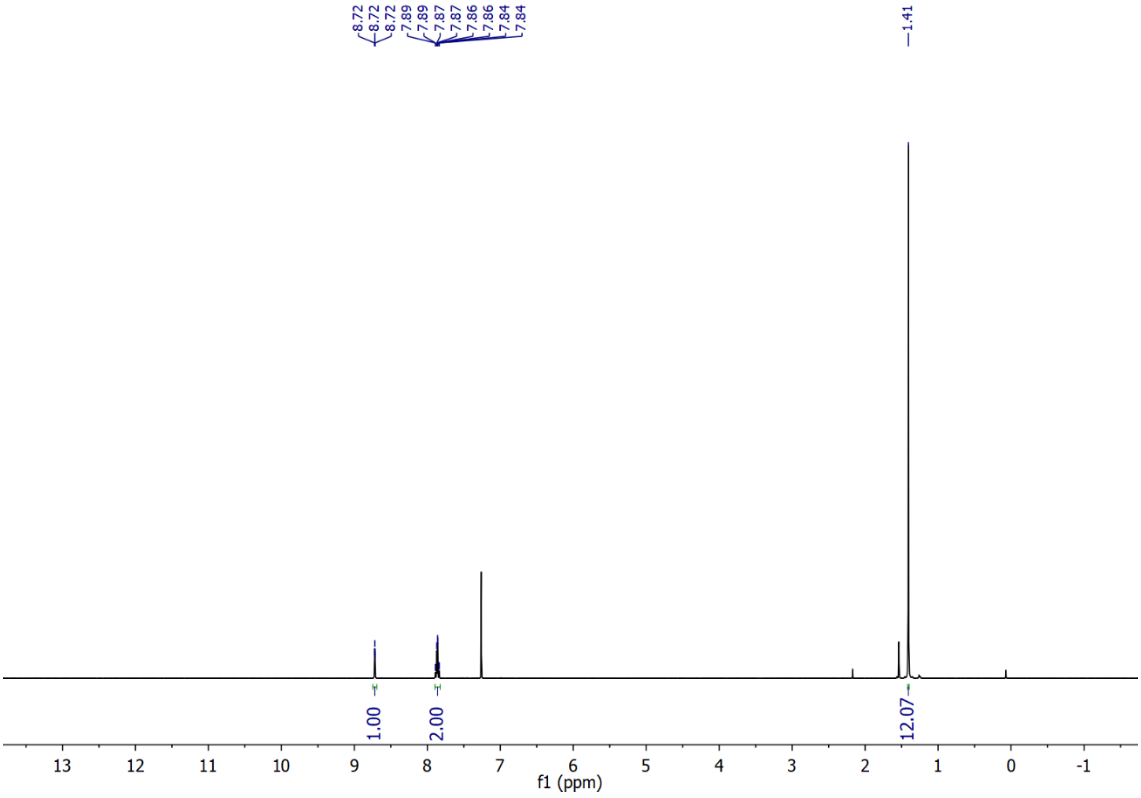

Figure S 9. Full ^{1}H-NMR (400 MHz, $CDCl_3$) spectrum of compound **3-S**.

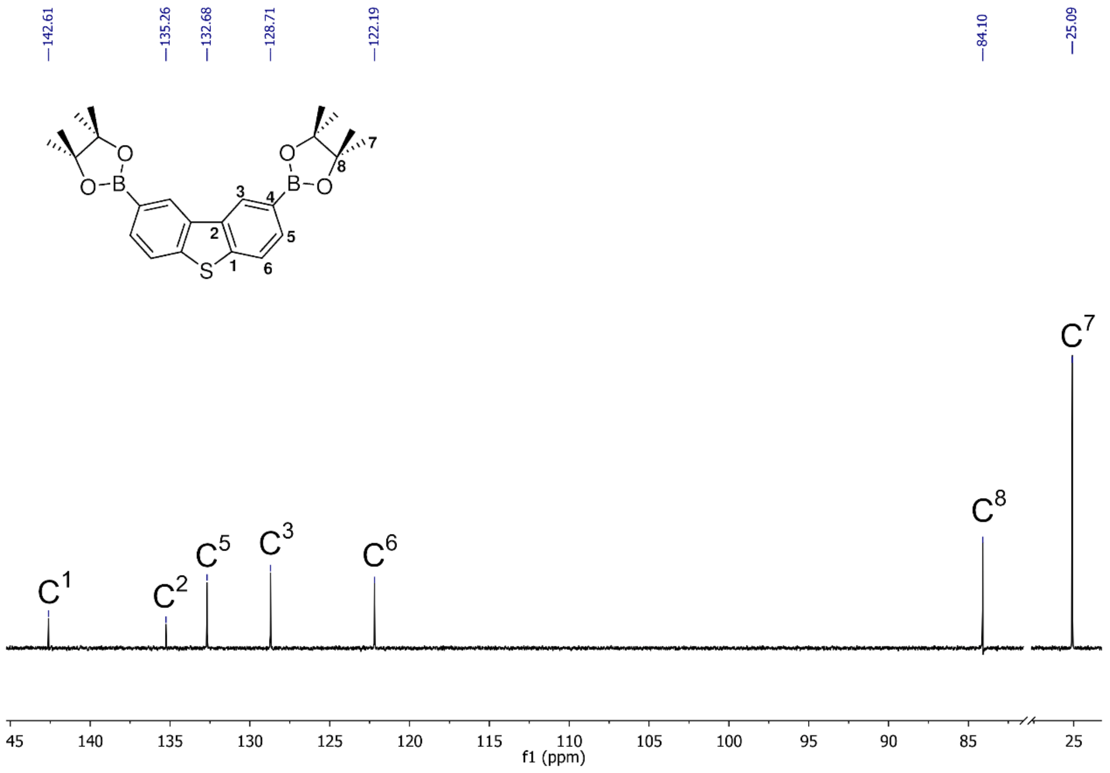

Figure S 10. $^{13}C\{^{1}H\}$-NMR (101 MHz, $CDCl_3$) spectrum of compound **3-S**.

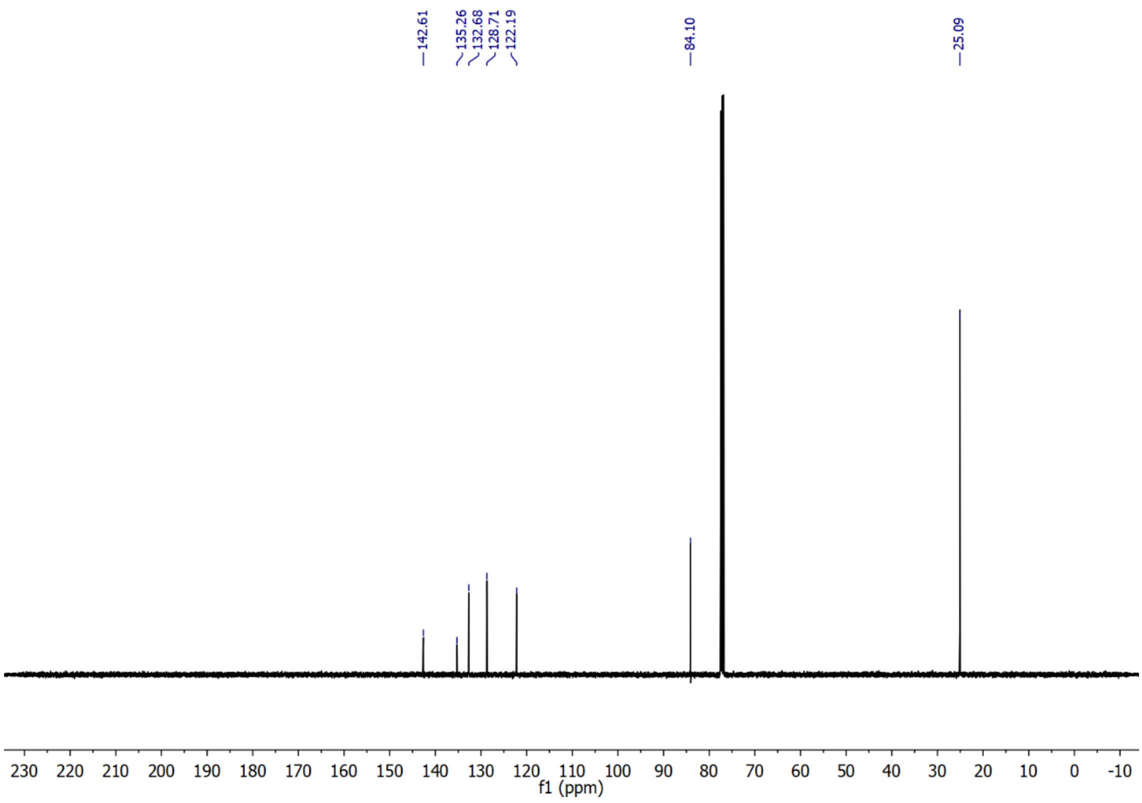

Figure S 11. Full $^{13}C\{^{1}H\}$-NMR (101 MHz, $CDCl_3$) spectrum of compound **3-S**.

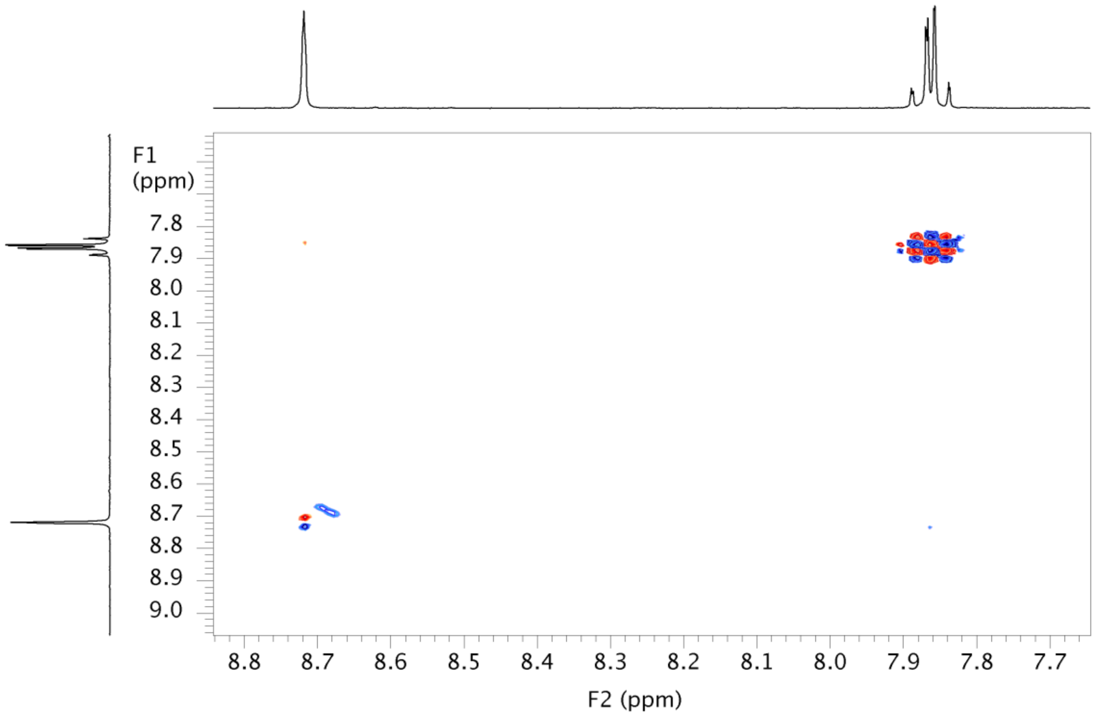

Figure S 12. ^{1}H-^{1}H gDQFCOSY (400 MHz, $CDCl_3$) spectrum of compound **3-S**.

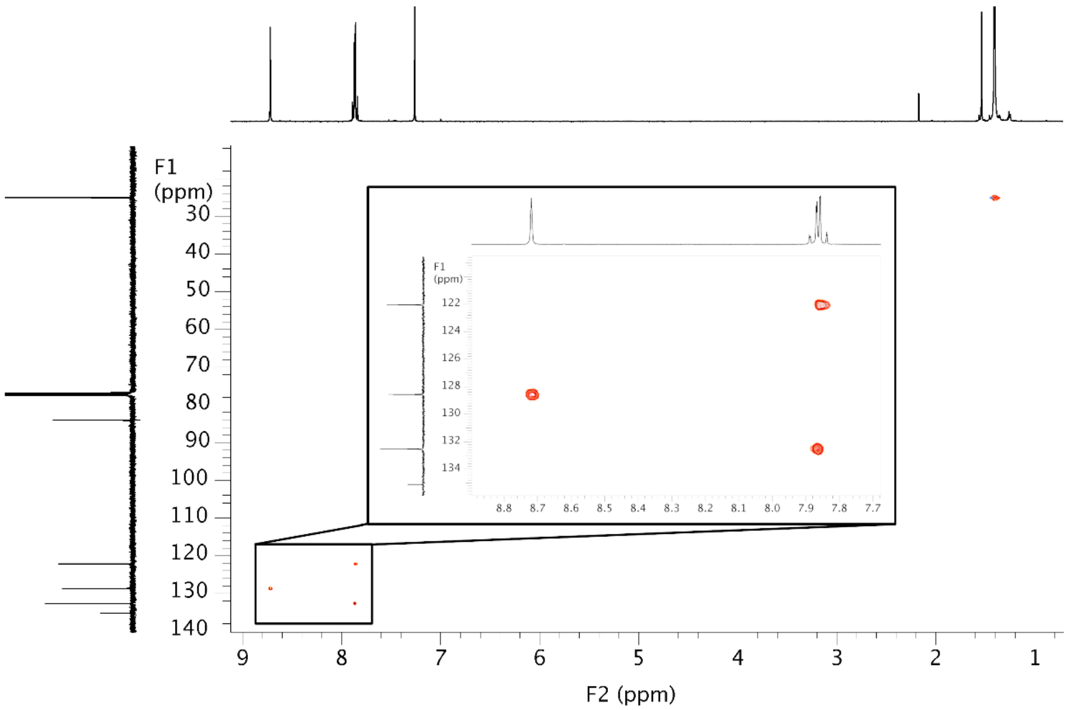

Figure S 13. ^{1}H-^{13}C gc2HSQC (400 MHz, $CDCl_3$) spectrum of compound **3-S**.

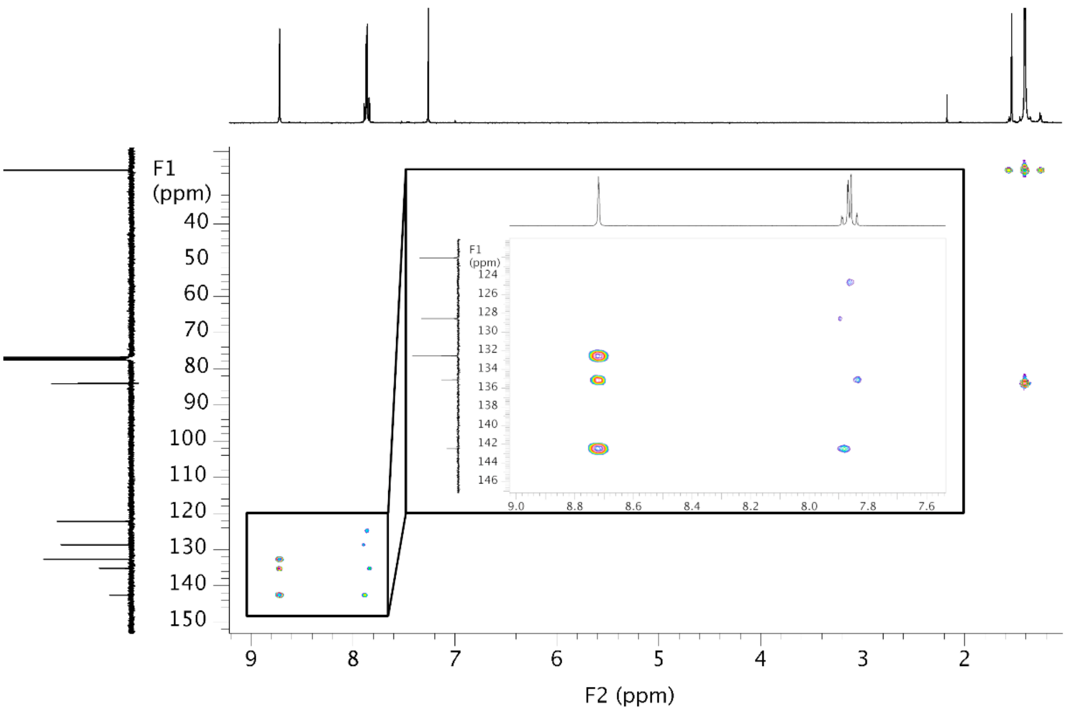

Figure S 14. ^{1}H-^{13}C gc2HMBC (400 MHz, $CDCl_3$) spectrum of compound **3-S**.

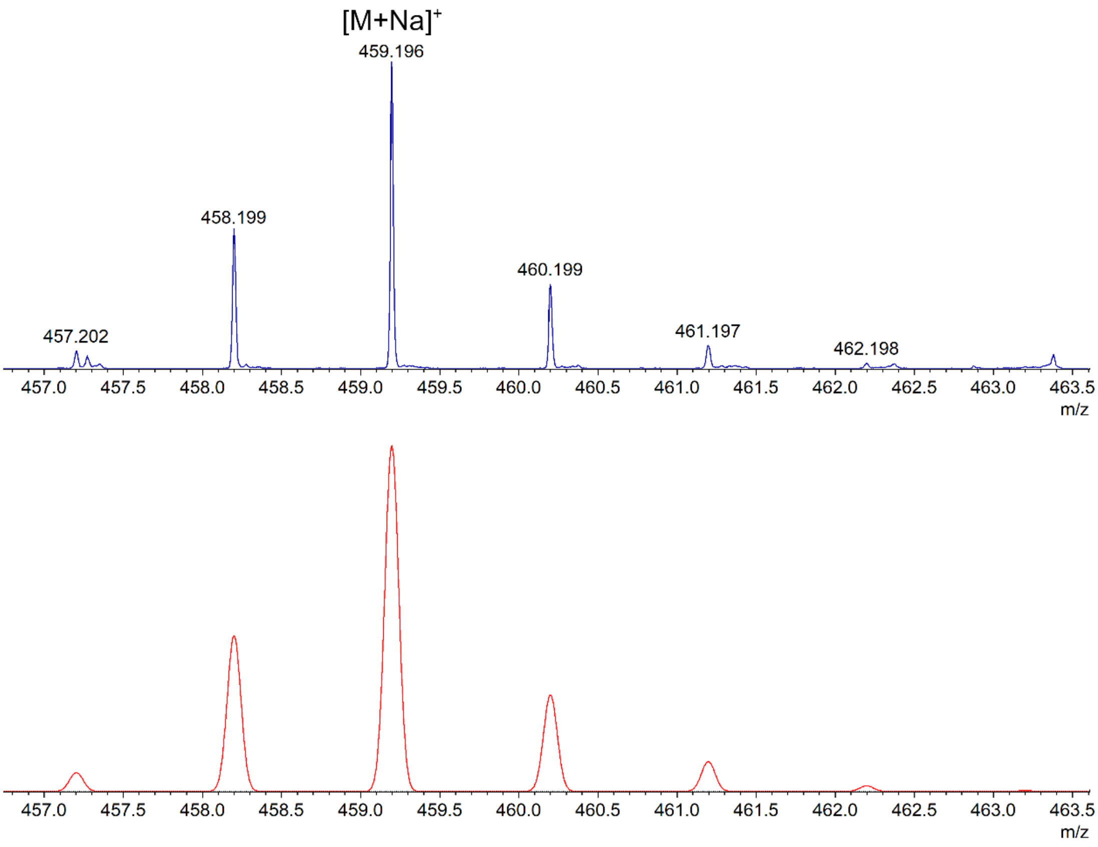

Figure S 15. HRMS (ESI-TOF) of compound **3-S**, $[M+Na]^+$. Calculated (red), measured (blue).

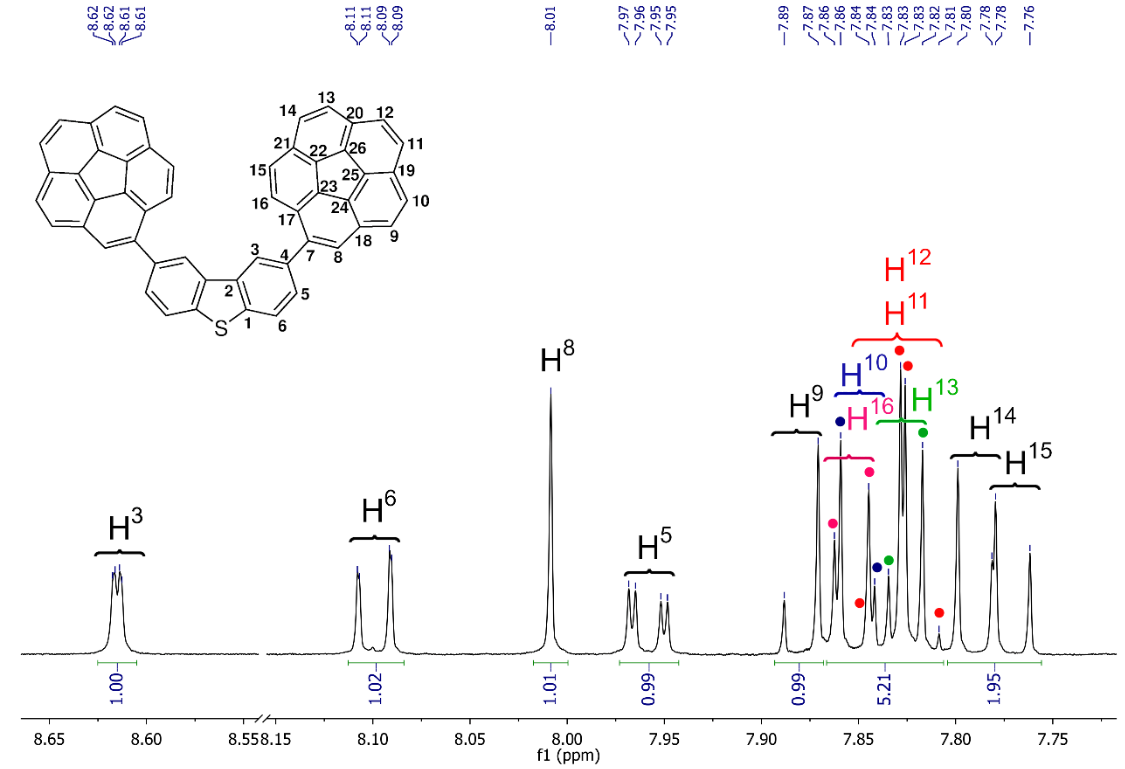

Figure S 16. ^{1}H-NMR (500 MHz, $CDCl_3$) spectrum of compound **4-S**.

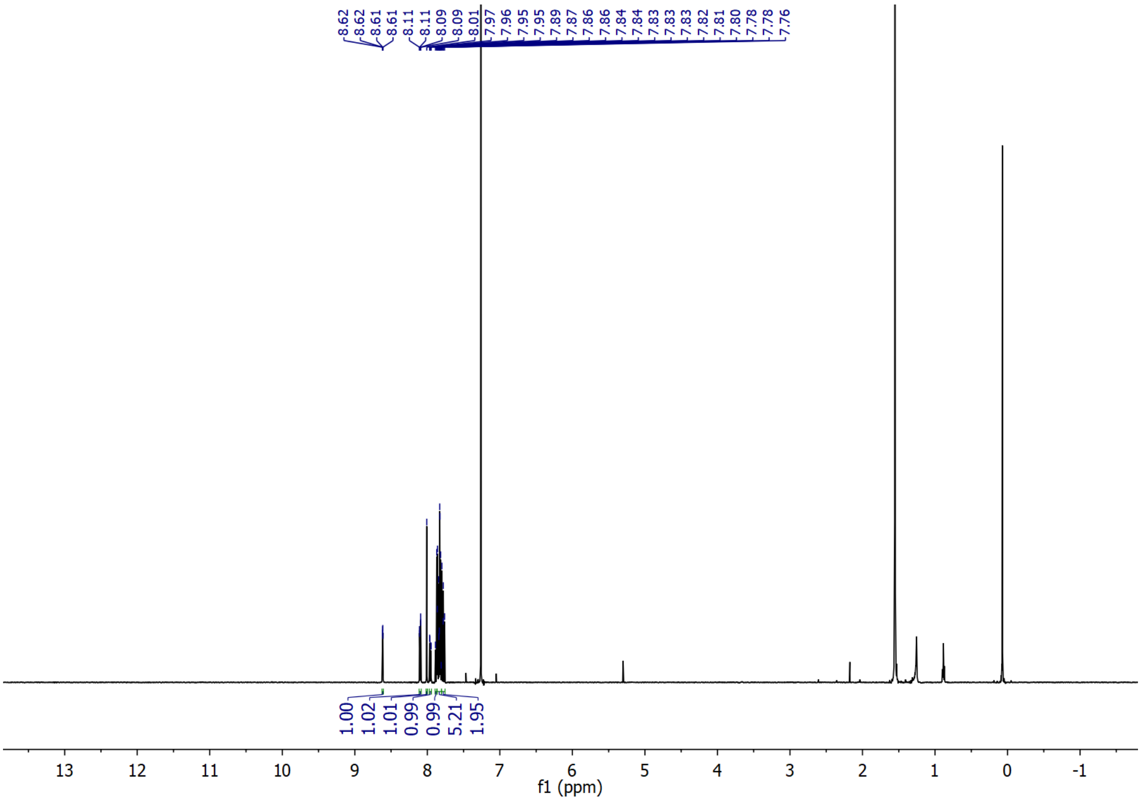

Figure S 17. Full ^{1}H-NMR (500 MHz, $CDCl_3$) spectrum of compound **4-S**.

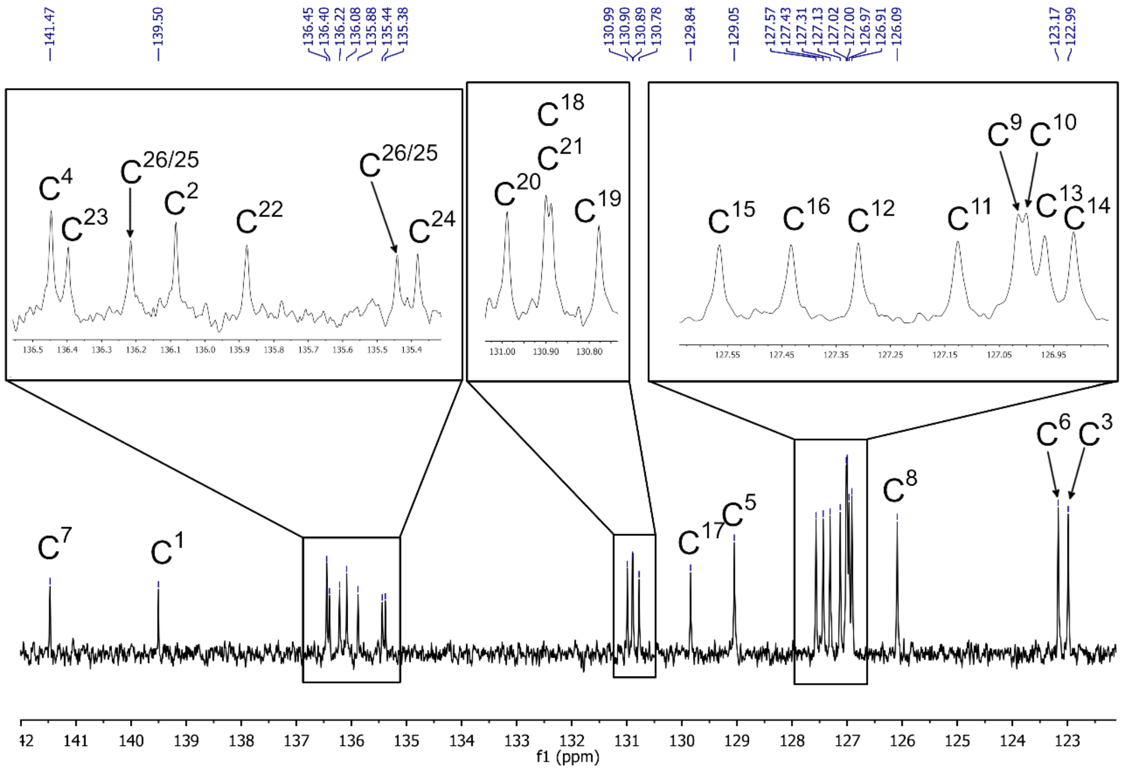

Figure S 18. $^{13}C\{^1H\}$-NMR (101 MHz, $CDCl_3$) spectrum of compound **4-S**.

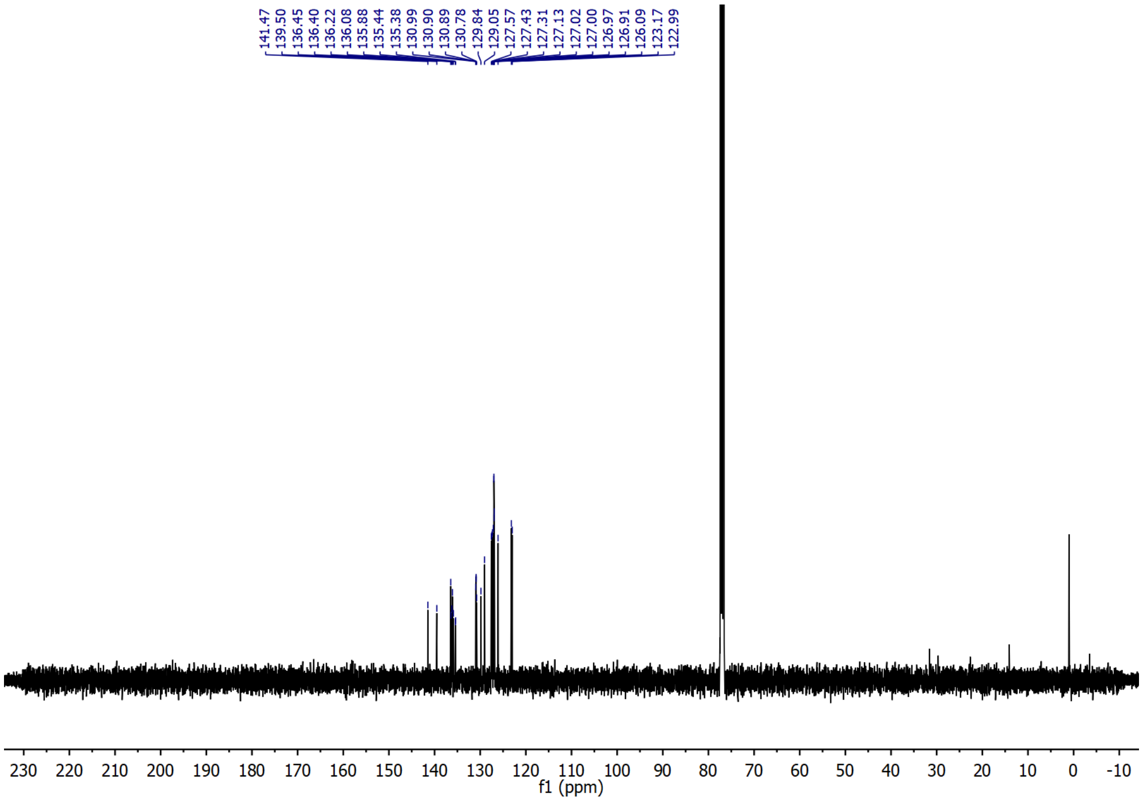

Figure S 19. Full $^{13}C\{^1H\}$-NMR (101 MHz, $CDCl_3$) spectrum of compound **4-S**.

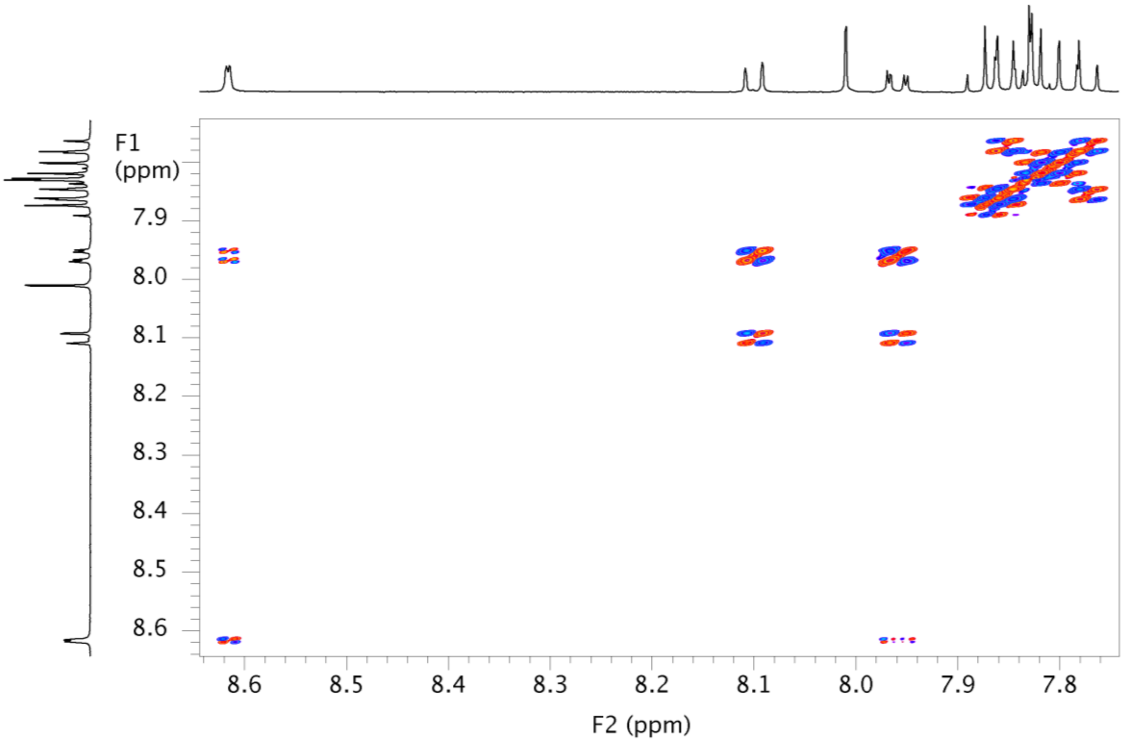

Figure S 20. ^{1}H-^{1}H gDQFCOSY (500 MHz, $CDCl_3$) spectrum of compound **4-S**.

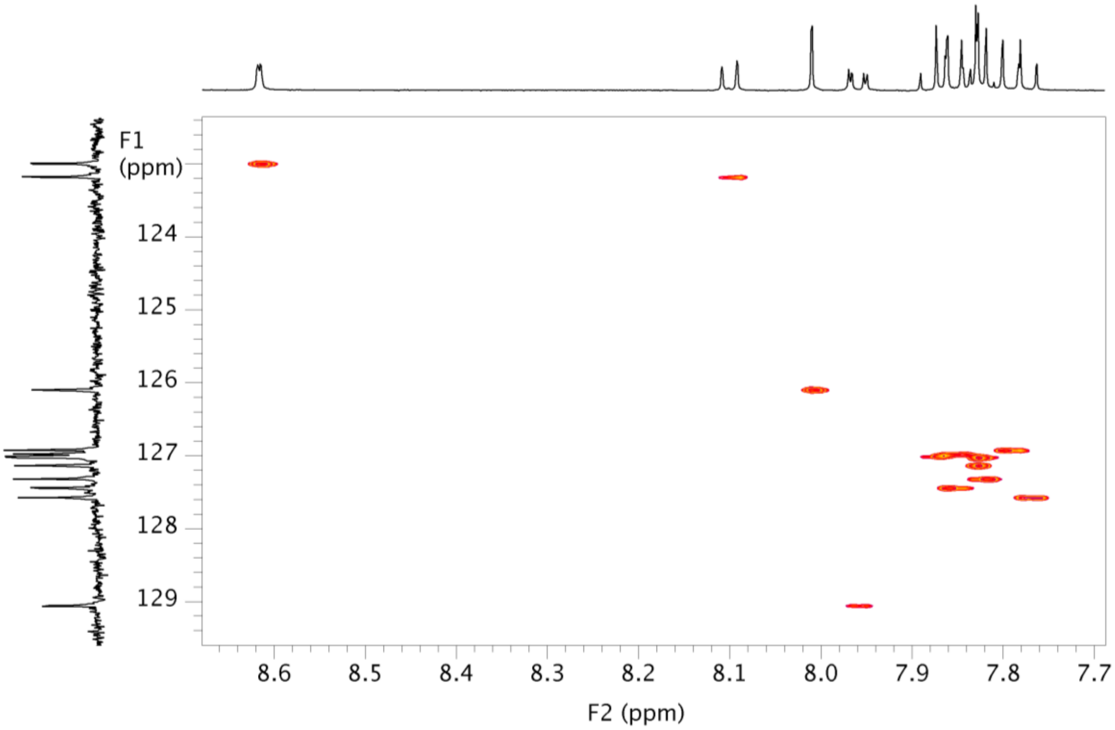

Figure S 21. ^{1}H-^{13}C bsgHSQCAD (500 MHz, $CDCl_3$) spectrum of compound **4-S**.

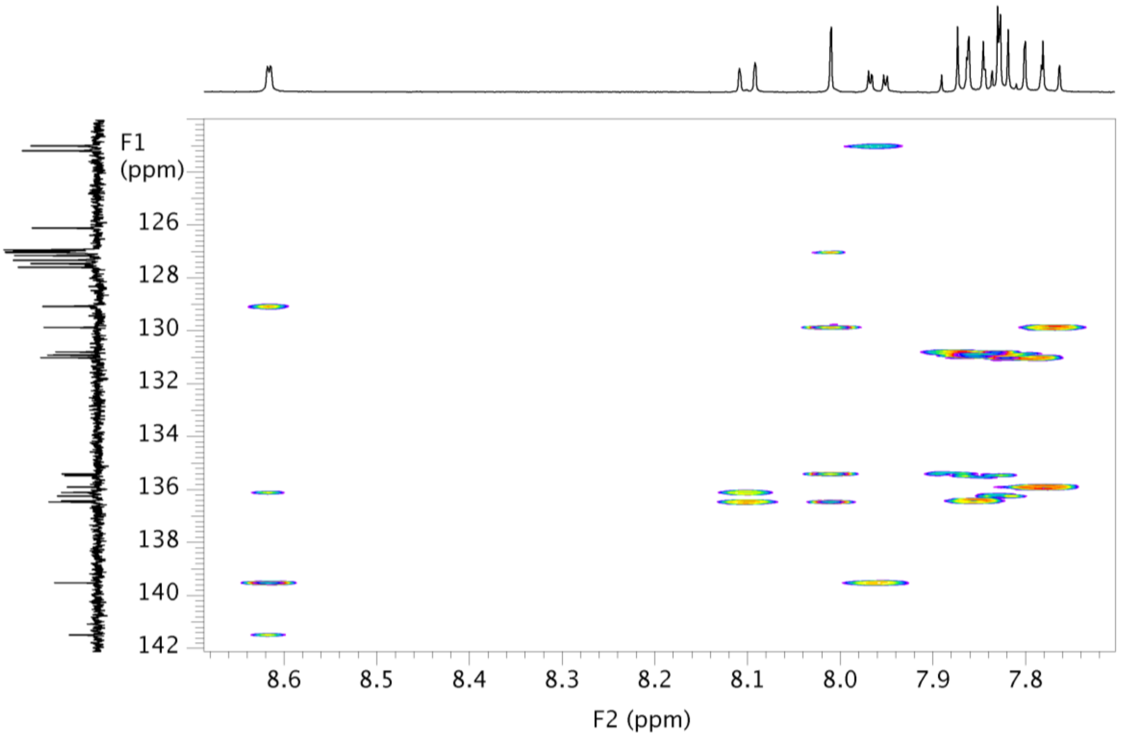

Figure S 22. ^{1}H-^{13}C bsgHMBC (500 MHz, $CDCl_3$) spectrum of compound **4-S**.

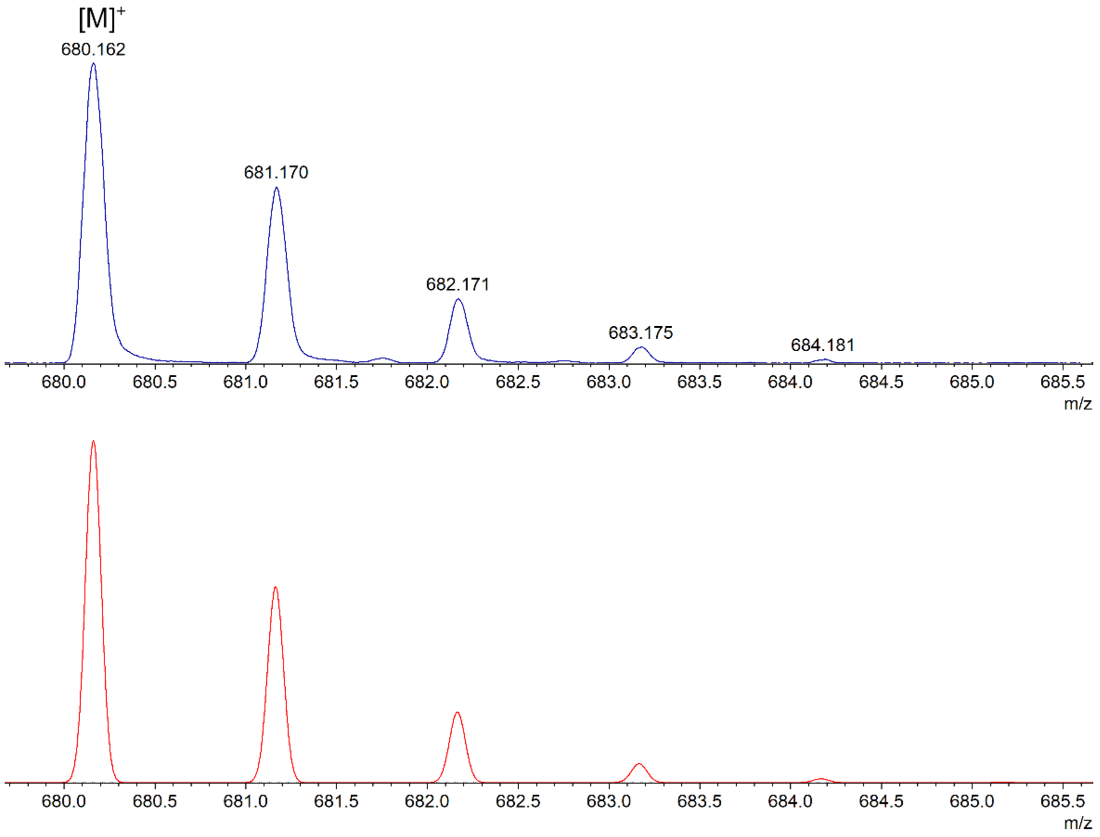

Figure S 23. HRMS (MALDI-TOF) of compound **4-S**, $[M]^{+}$. Calculated (red), measured (blue).

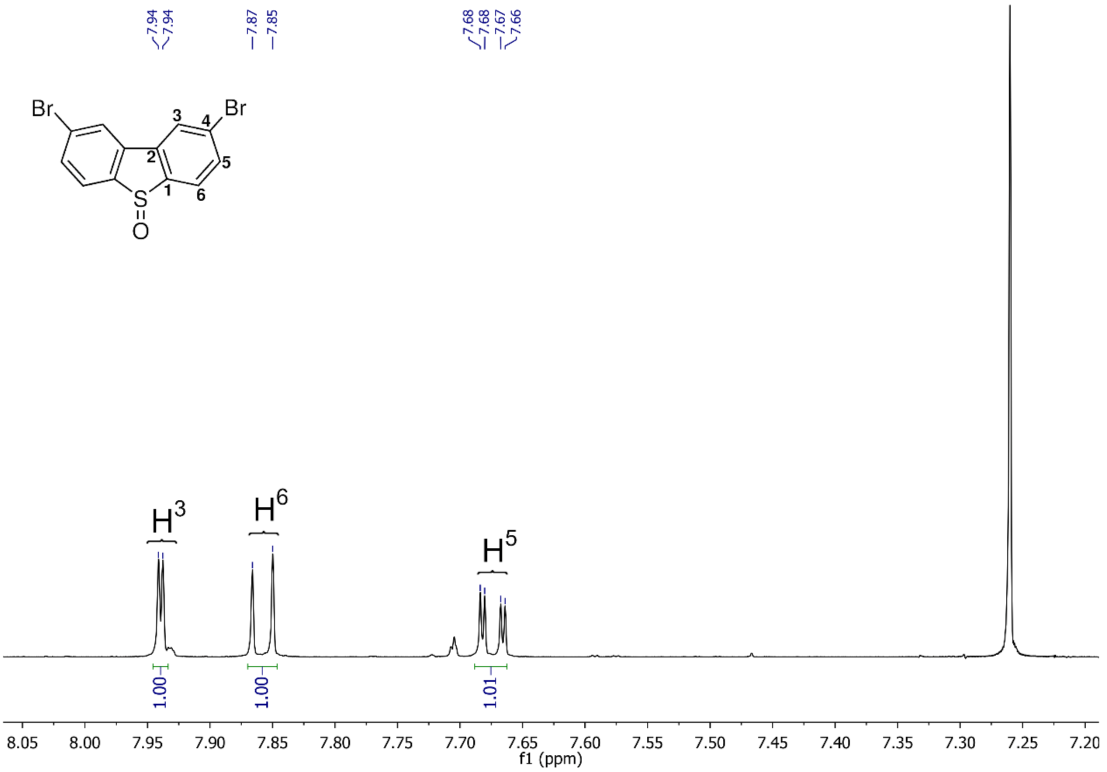

Figure S 24. ^{1}H-NMR (500 MHz, $CDCl_3$) spectrum of compound **2-SO**.

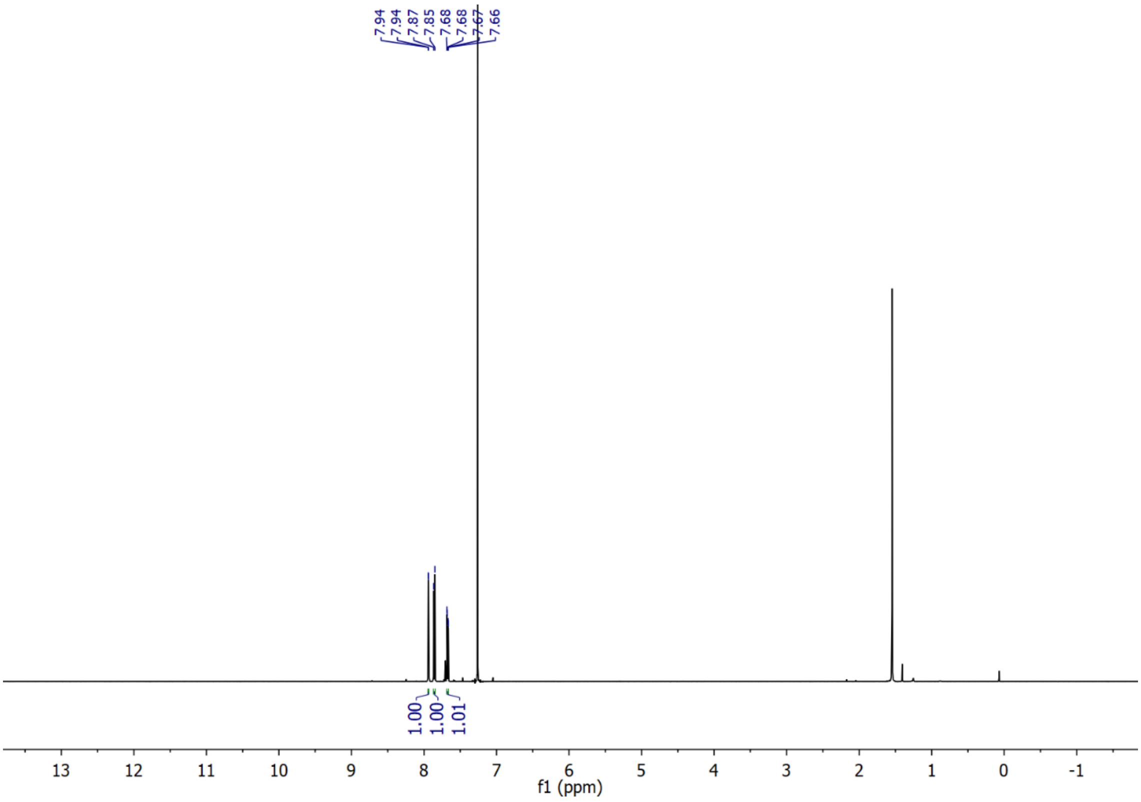

Figure S 25. Full ^{1}H-NMR (500 MHz, $CDCl_3$) spectrum of compound **2-SO**.

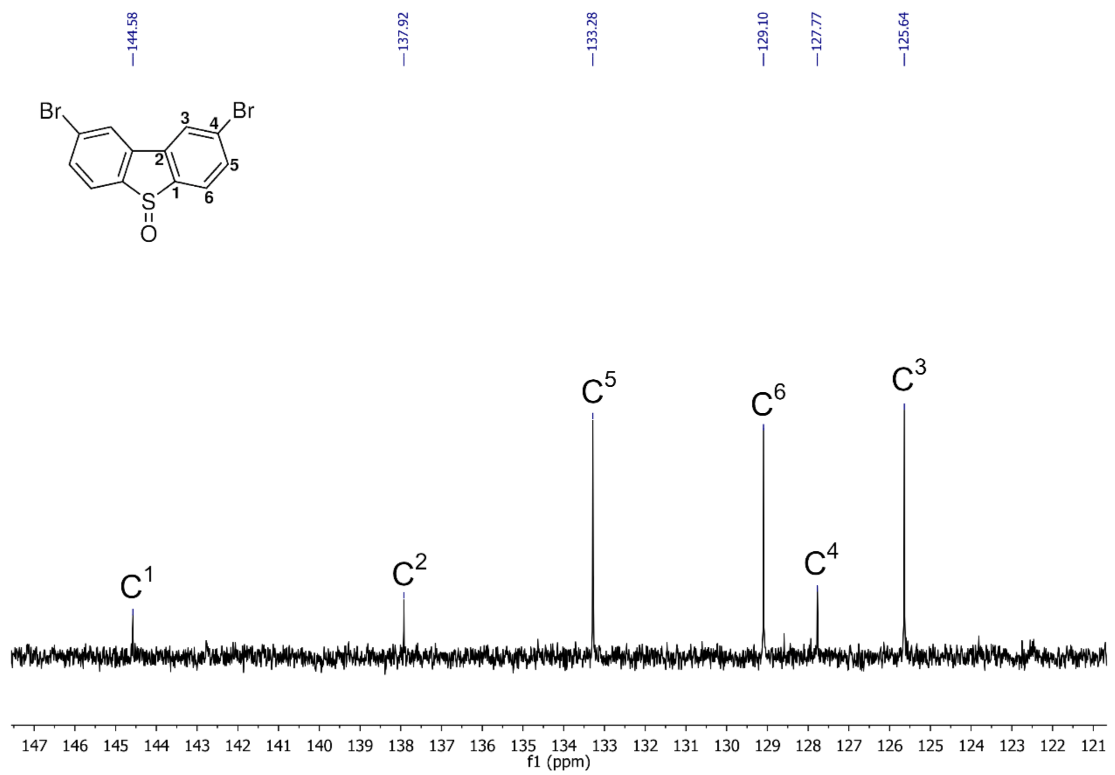

Figure S 26. $^{13}C\{^{1}H\}$-NMR (101 MHz, $CDCl_3$) spectrum of compound **2-SO**.

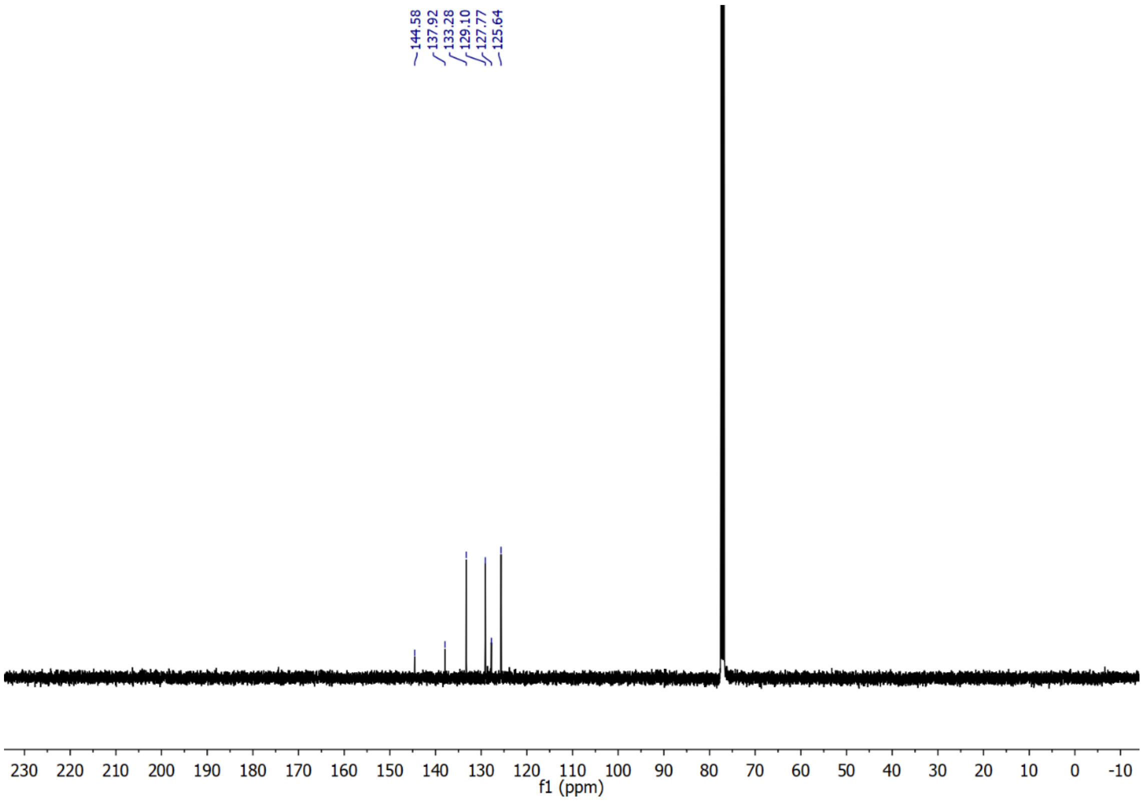

Figure S 27. Full $^{13}C\{^{1}H\}$-NMR (101 MHz, $CDCl_3$) spectrum of compound **2-SO**.

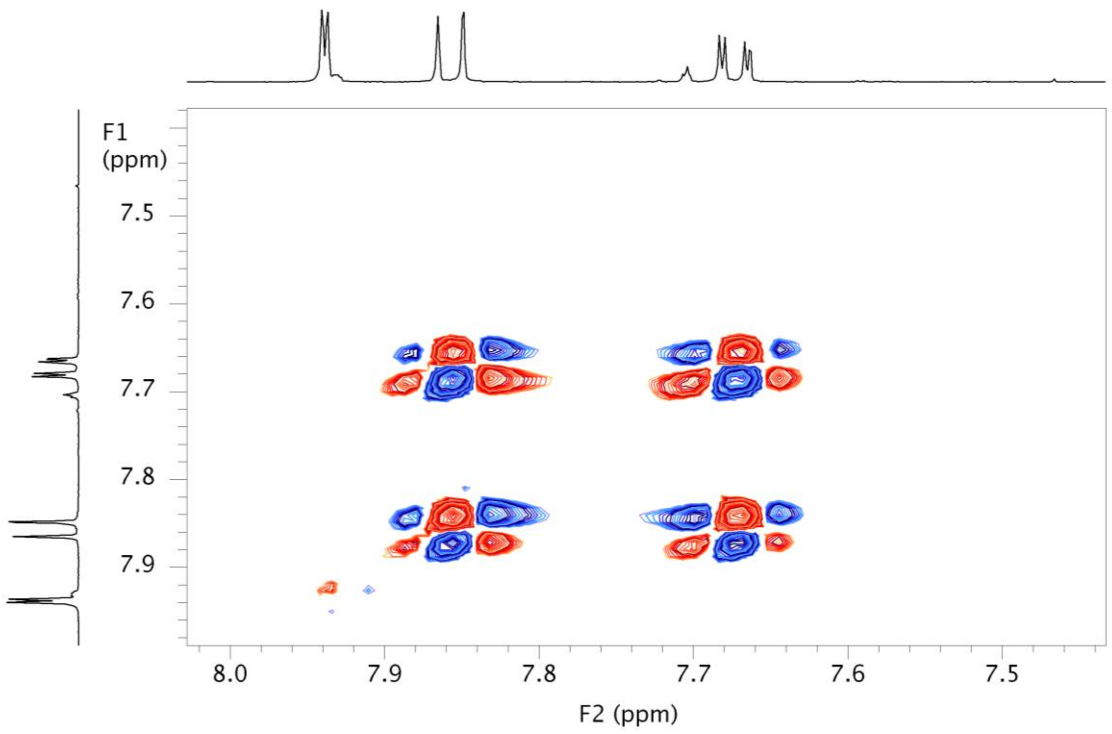

Figure S 28. ^{1}H-^{1}H gDQFCOSY (400 MHz, $CDCl_3$) spectrum of compound **2-SO**.

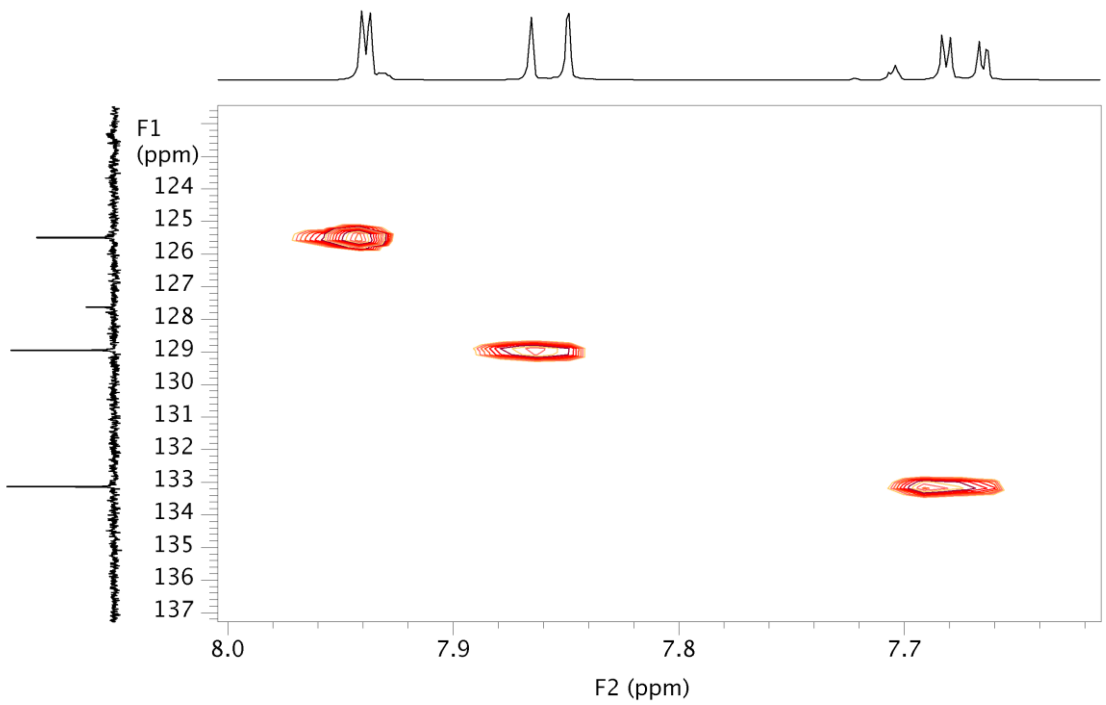

Figure S 29. ^{1}H-^{13}C gc2HSQC (400 MHz, $CDCl_3$) spectrum of compound **2-SO**.

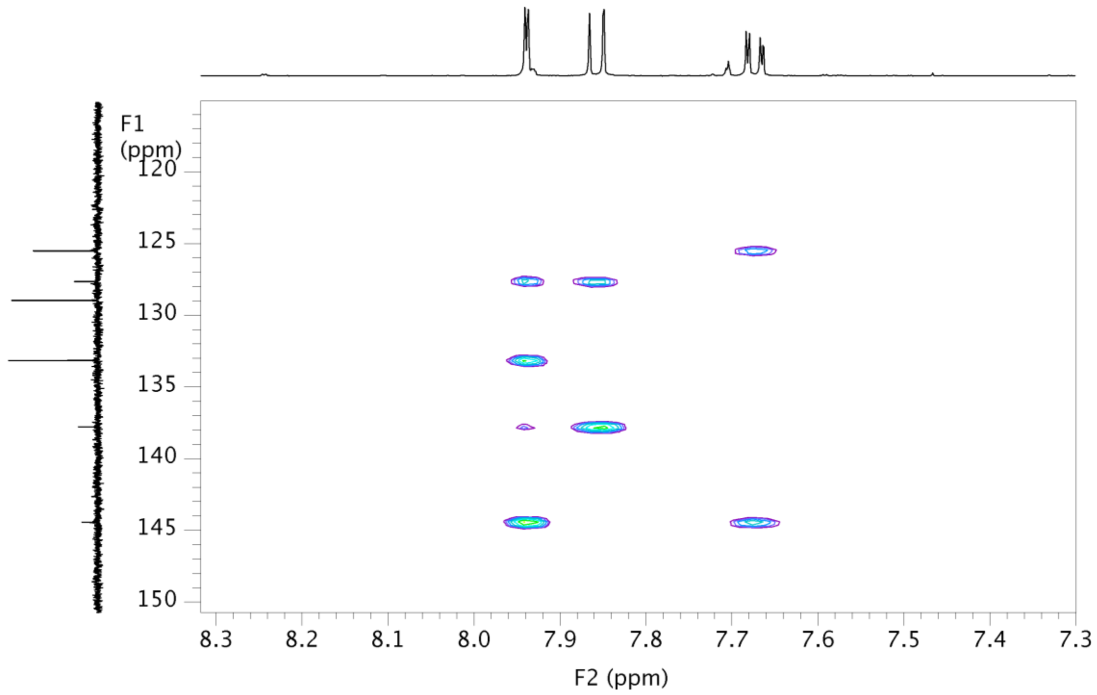

Figure S 30. ^{1}H-^{13}C gc2HMBC (400 MHz, $CDCl_3$) spectrum of compound **2-SO**.

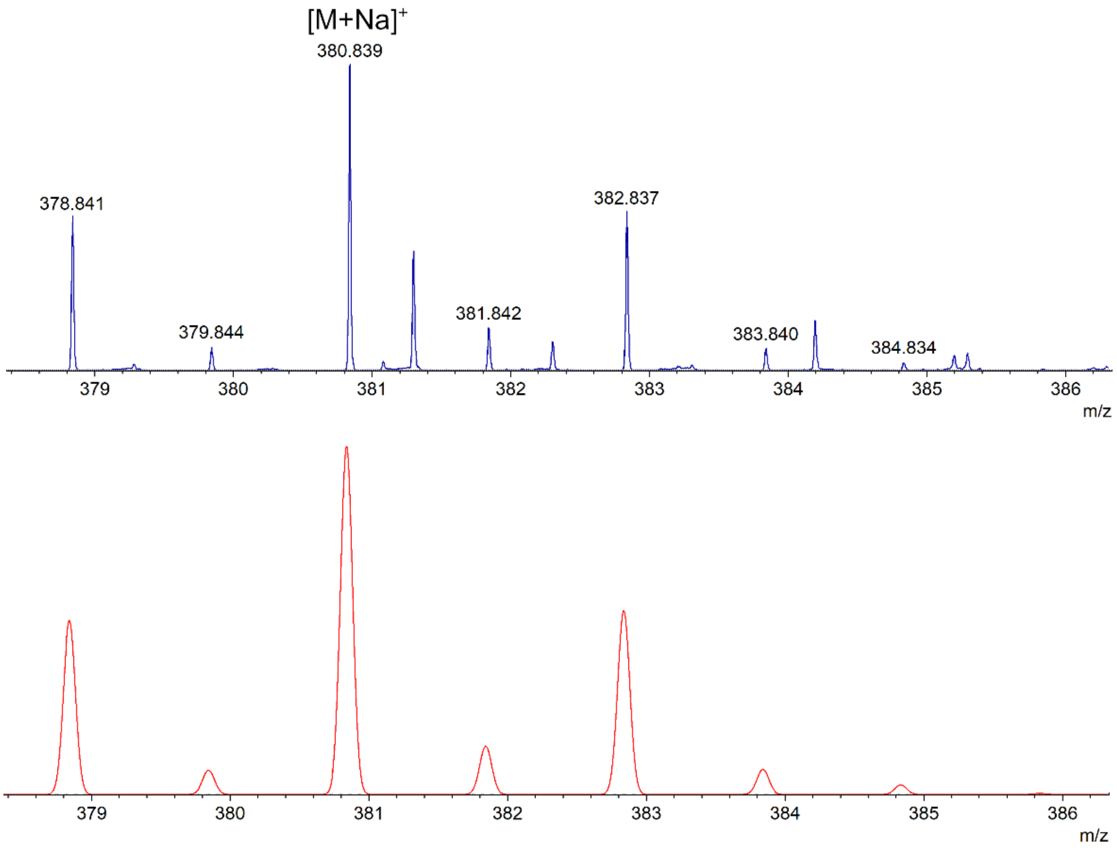

Figure S 31. HRMS (ESI-TOF) of compound **2-SO**, $[M+Na]^{+}$. Calculated (red), measured (blue).

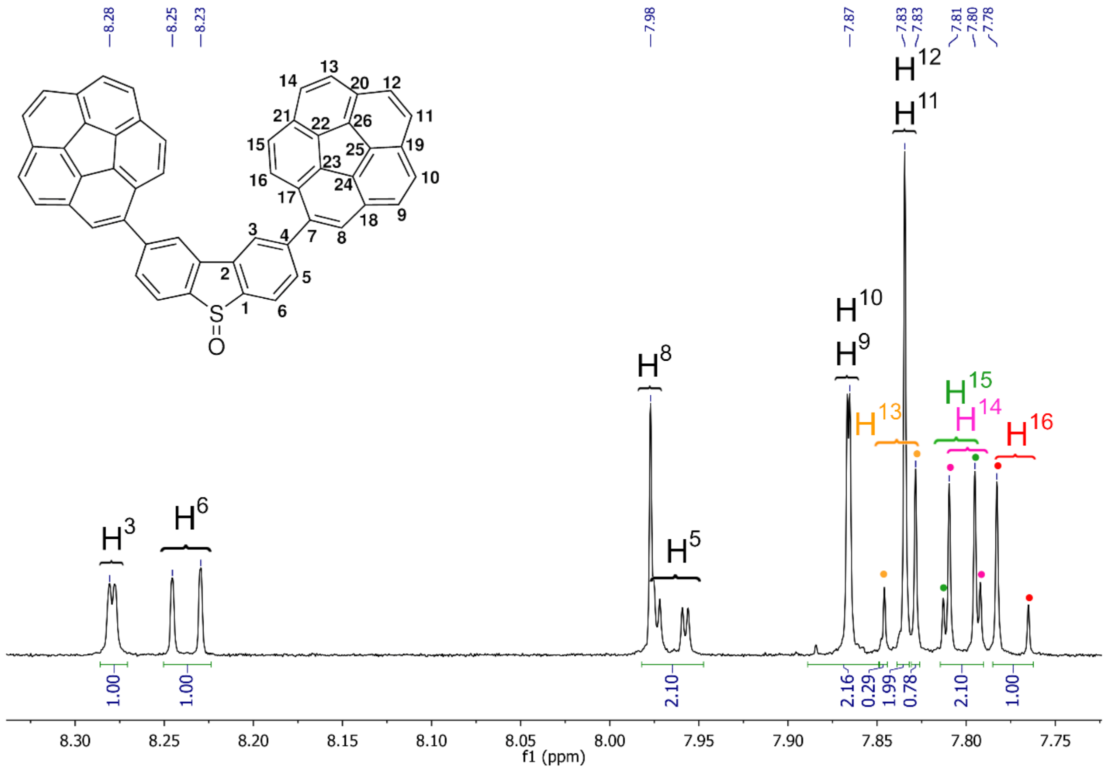

Figure S 32. ^{1}H-NMR (500 MHz, $CDCl_3$) spectrum of compound **4-SO**.

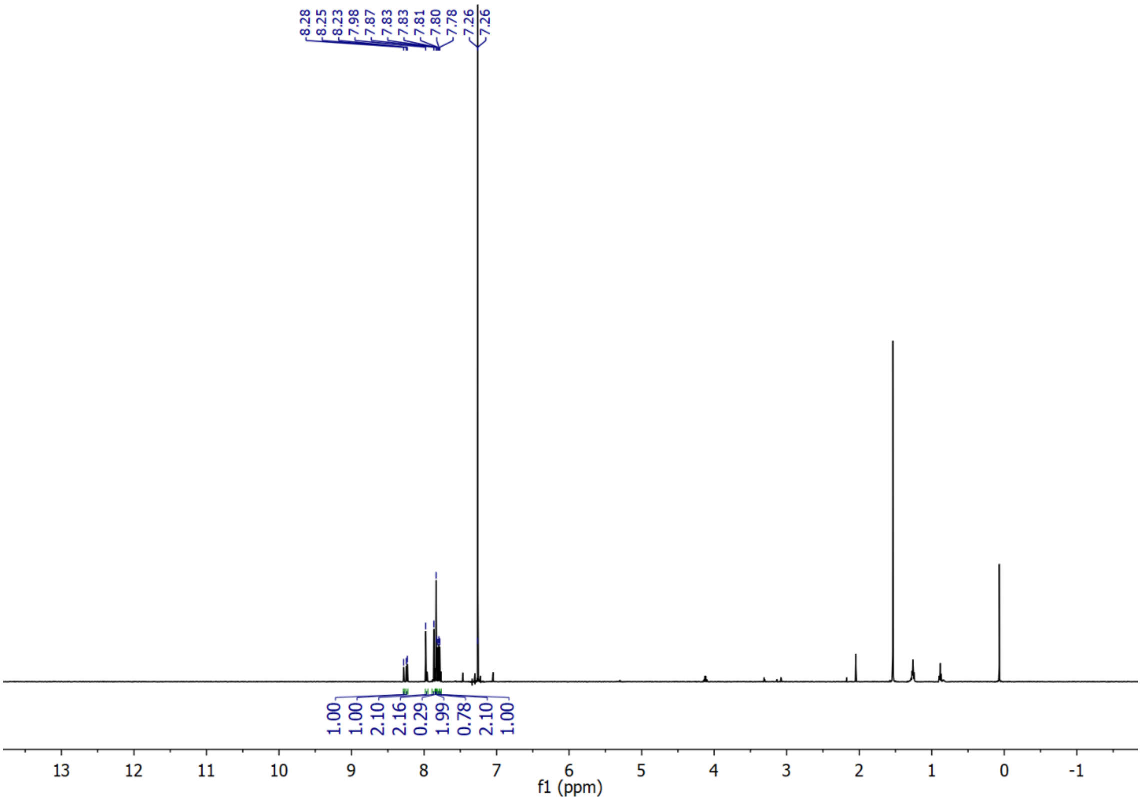

Figure S 33. Full ^{1}H-NMR (500 MHz, $CDCl_3$) spectrum of compound **4-SO**.

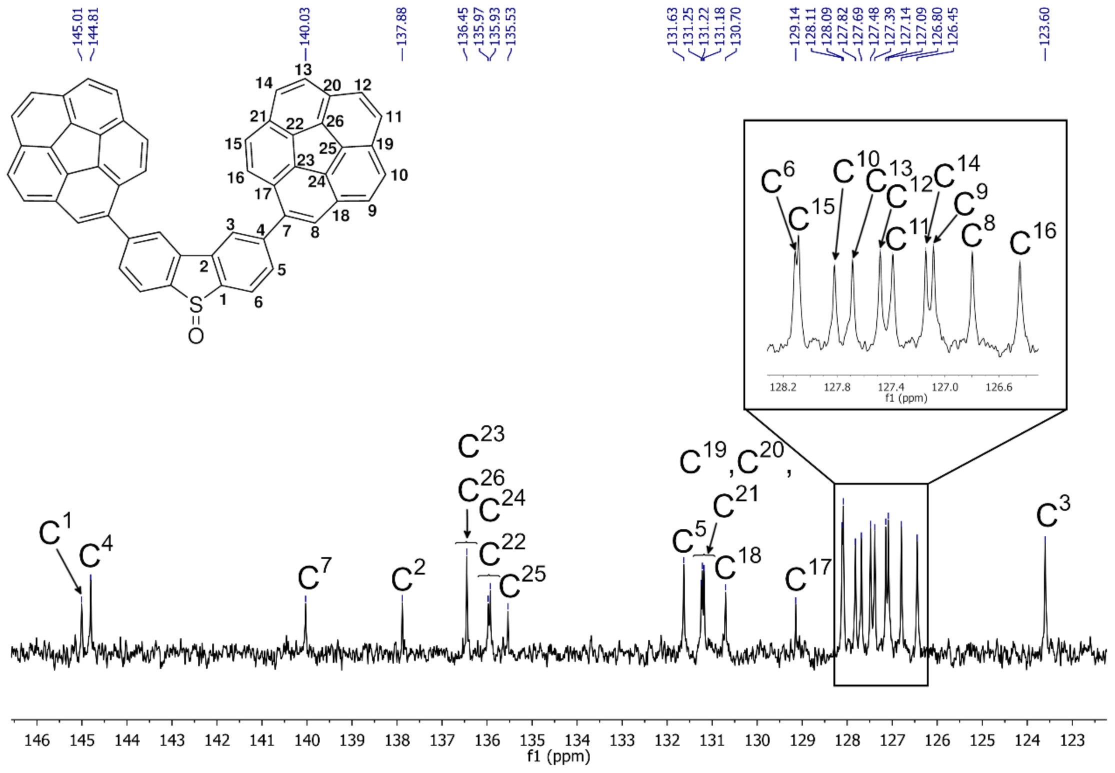

Figure S 34. $^{13}C\{^1H\}$-NMR (101 MHz, $CDCl_3$) spectrum of compound **4-SO**.

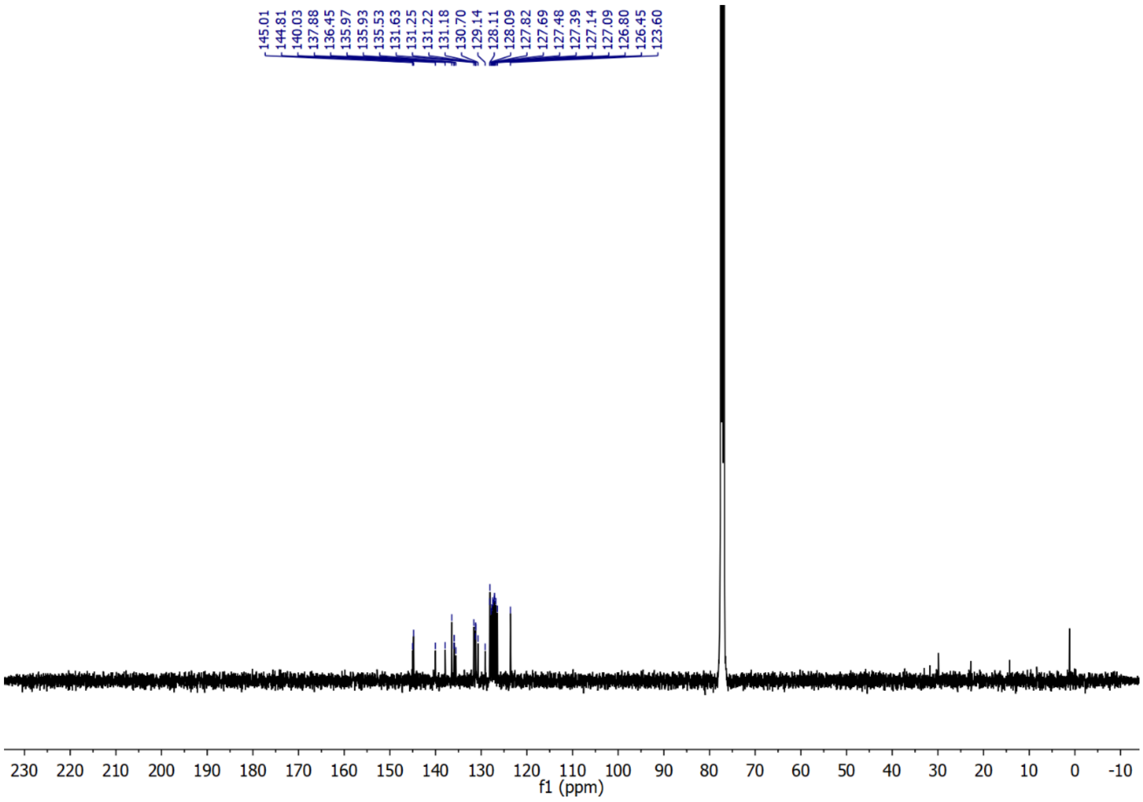

Figure S 35. Full $^{13}C\{^1H\}$-NMR (101 MHz, $CDCl_3$) spectrum of compound **4-SO**.

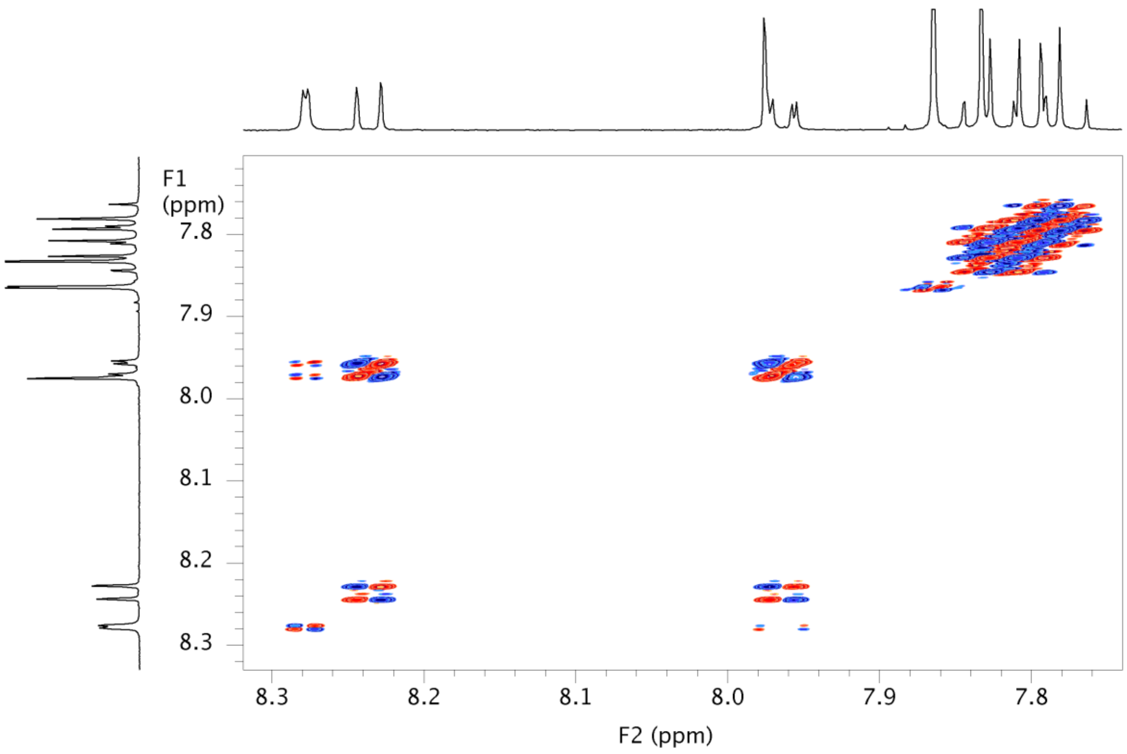

Figure S 36. ^{1}H-^{1}H gDQFCOSY (500 MHz, $CDCl_3$) spectrum of compound **4-SO**.

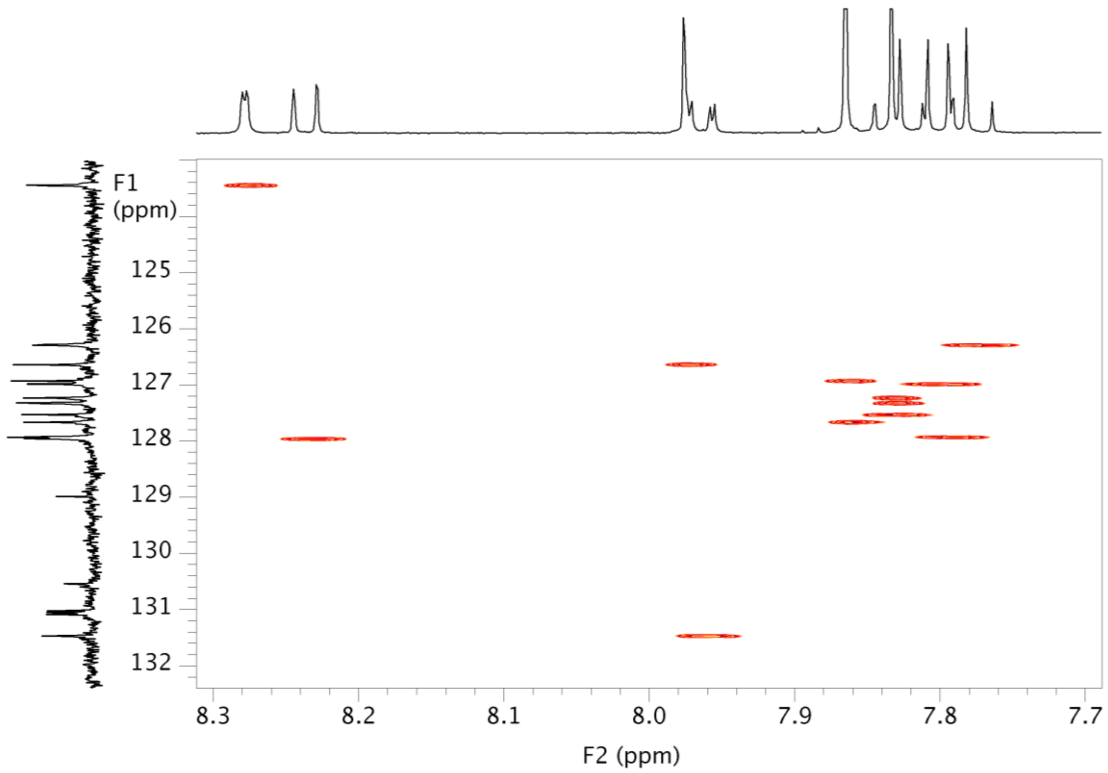

Figure S 37. ^{1}H-^{13}C bsgHSQCAD (500 MHz, $CDCl_3$) spectrum of compound **4-SO**.

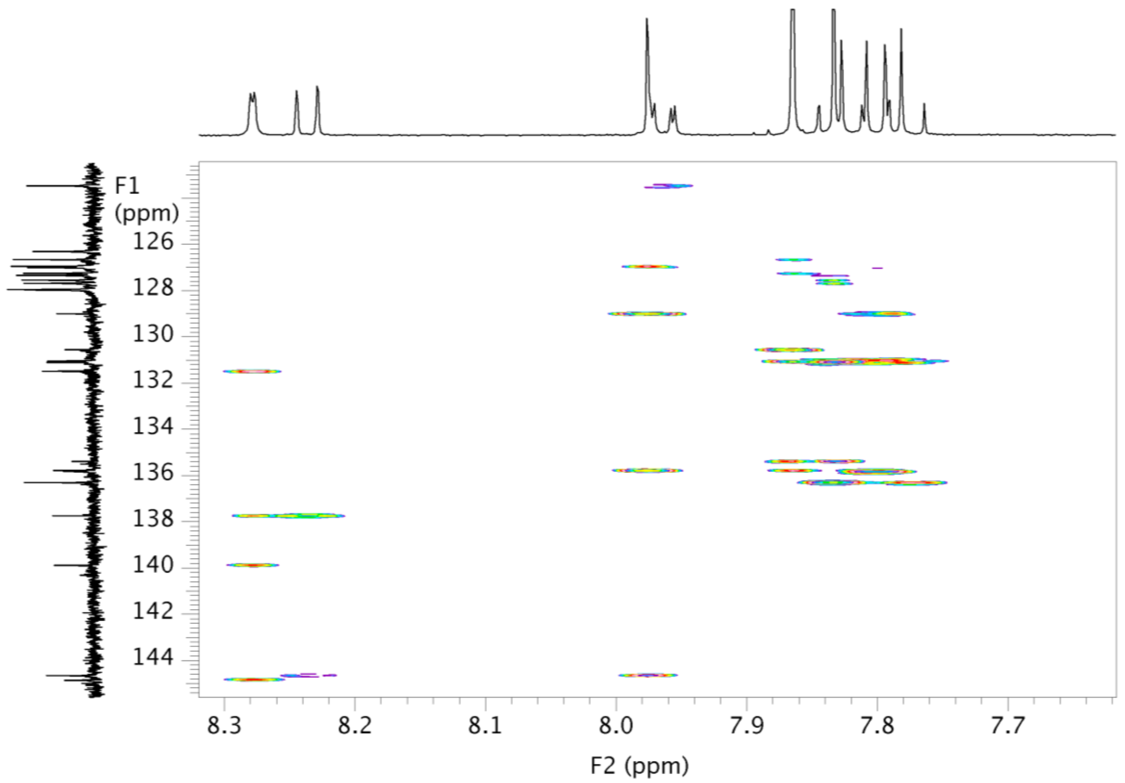

Figure S 38. ^{1}H-^{13}C bsgHMBC (500 MHz, $CDCl_3$) spectrum of compound **4-SO**.

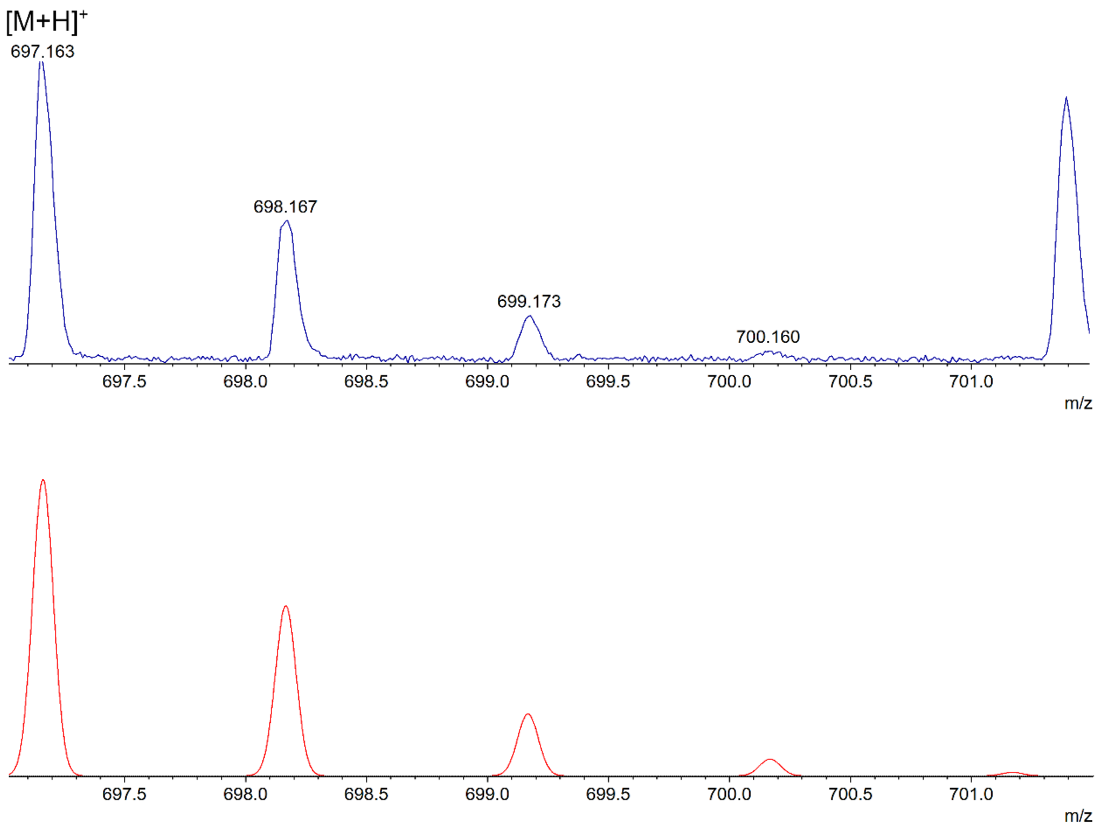

Figure S 39. HRMS (MALDI-TOF) of compound **4-SO**, $[M+H]^+$. Calculated (red), measured (blue).

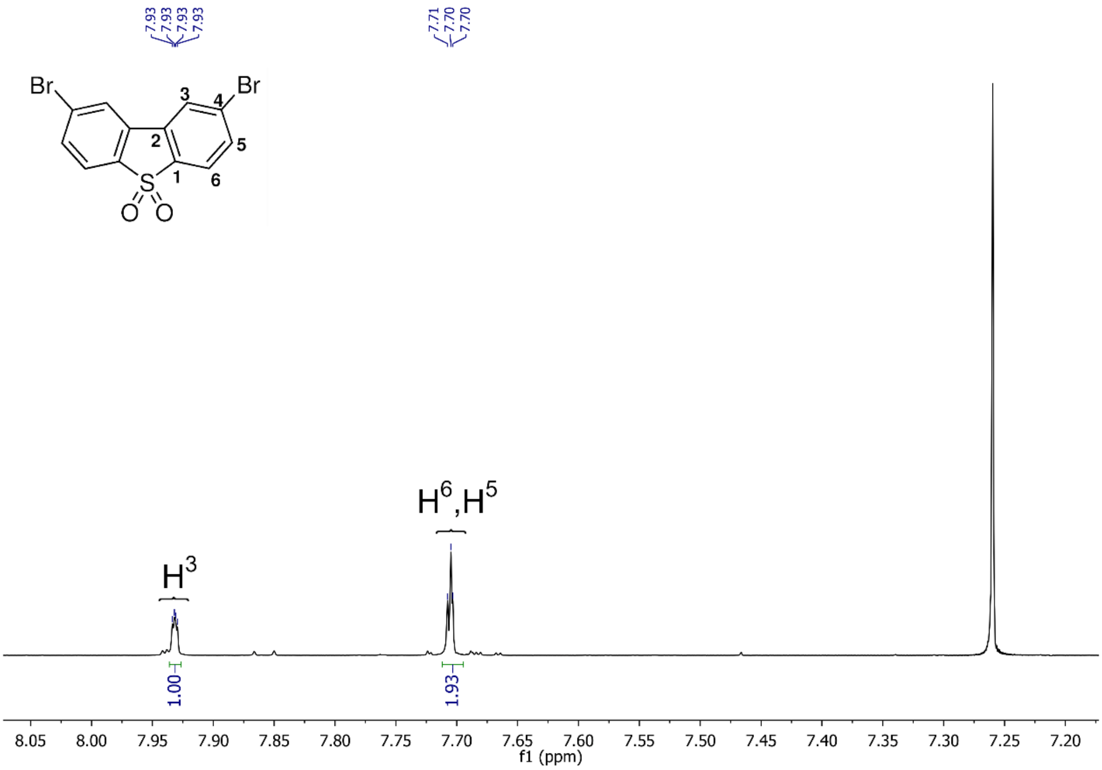

Figure S 40. ^{1}H-NMR (500 MHz, $CDCl_3$) spectrum of compound **2-SO₂**.

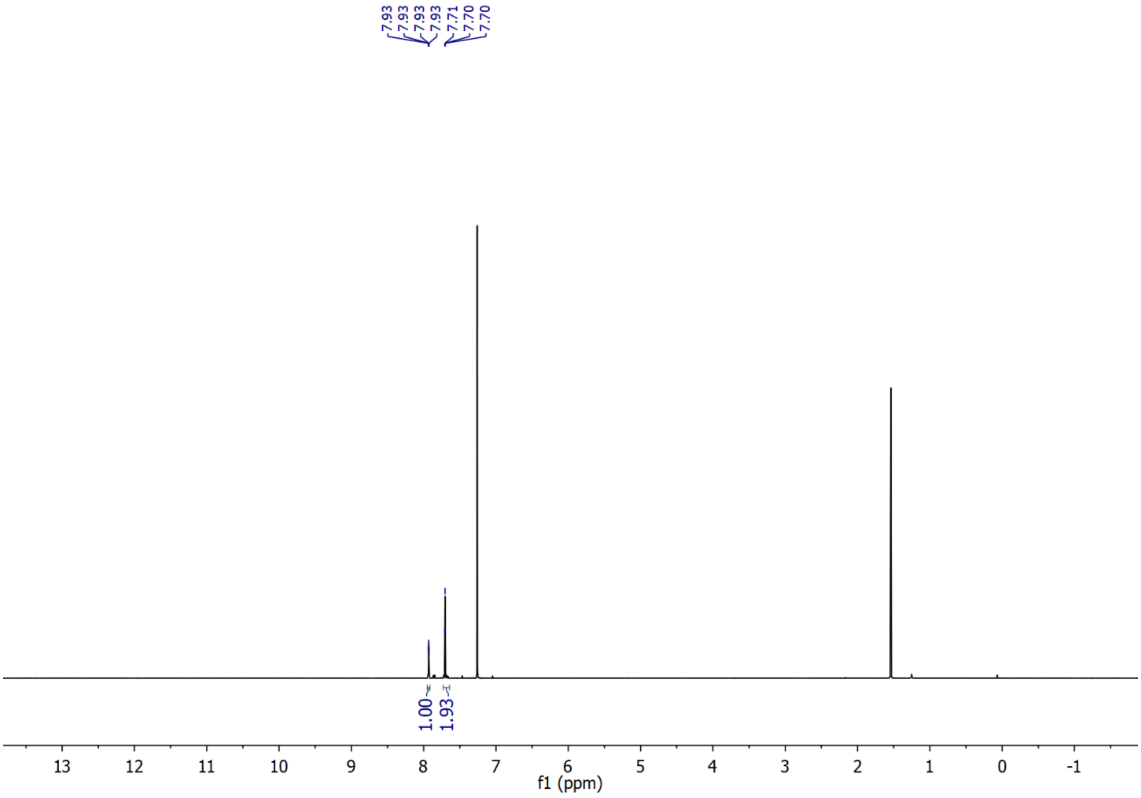

Figure S 41. Full ^{1}H-NMR (500 MHz, $CDCl_3$) spectrum of compound **2-SO₂**.

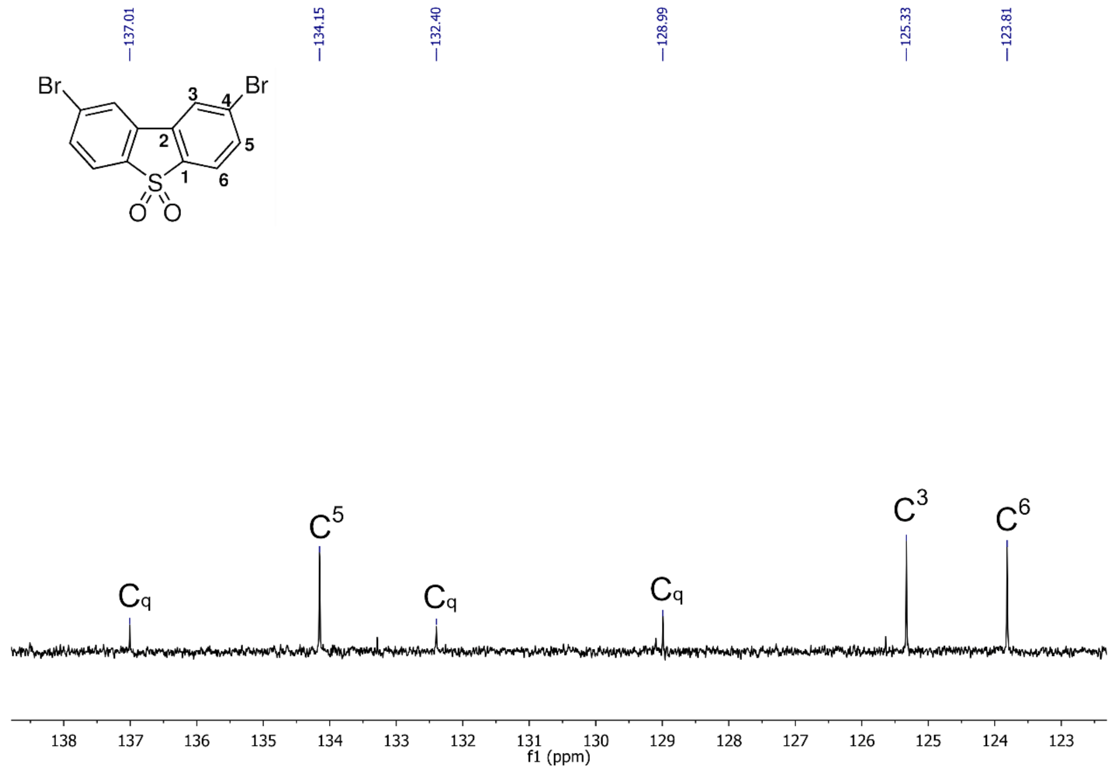

Figure S 42. $^{13}C\{^{1}H\}$-NMR (101 MHz, $CDCl_3$) spectrum of compound **2-SO₂**.

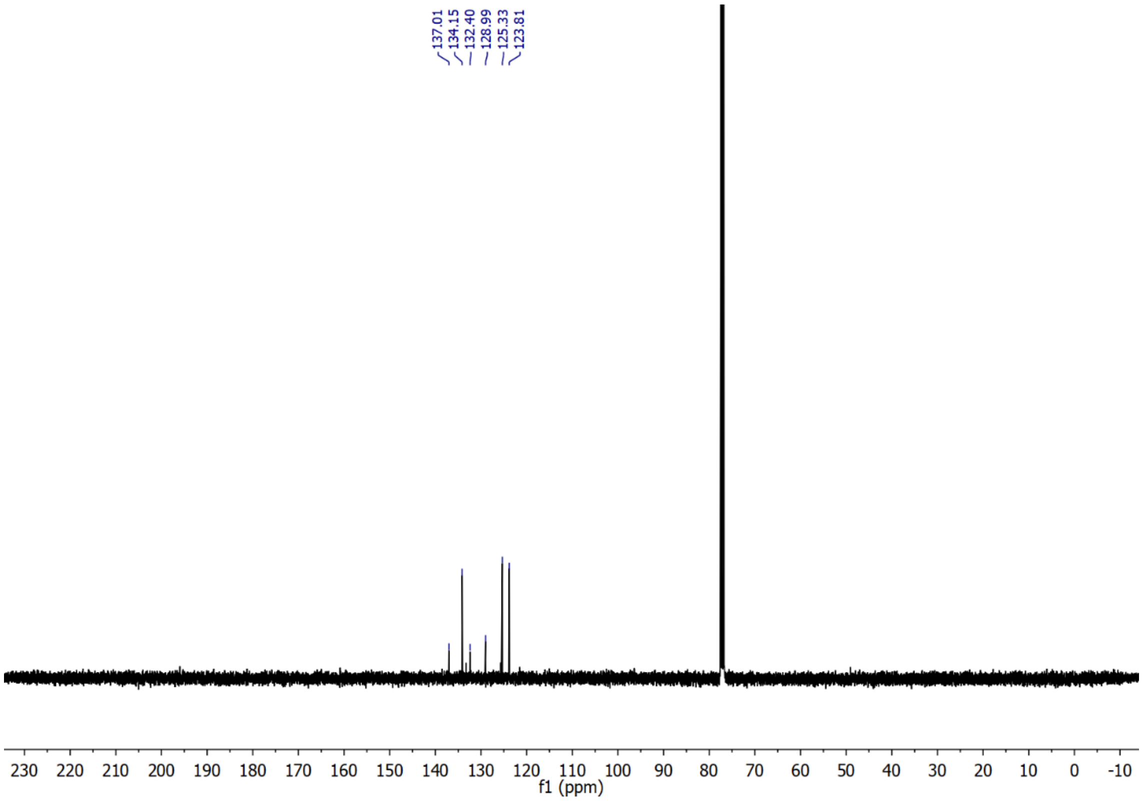

Figure S 43. Full $^{13}C\{^{1}H\}$-NMR (101 MHz, $CDCl_3$) spectrum of compound **2-SO₂**.

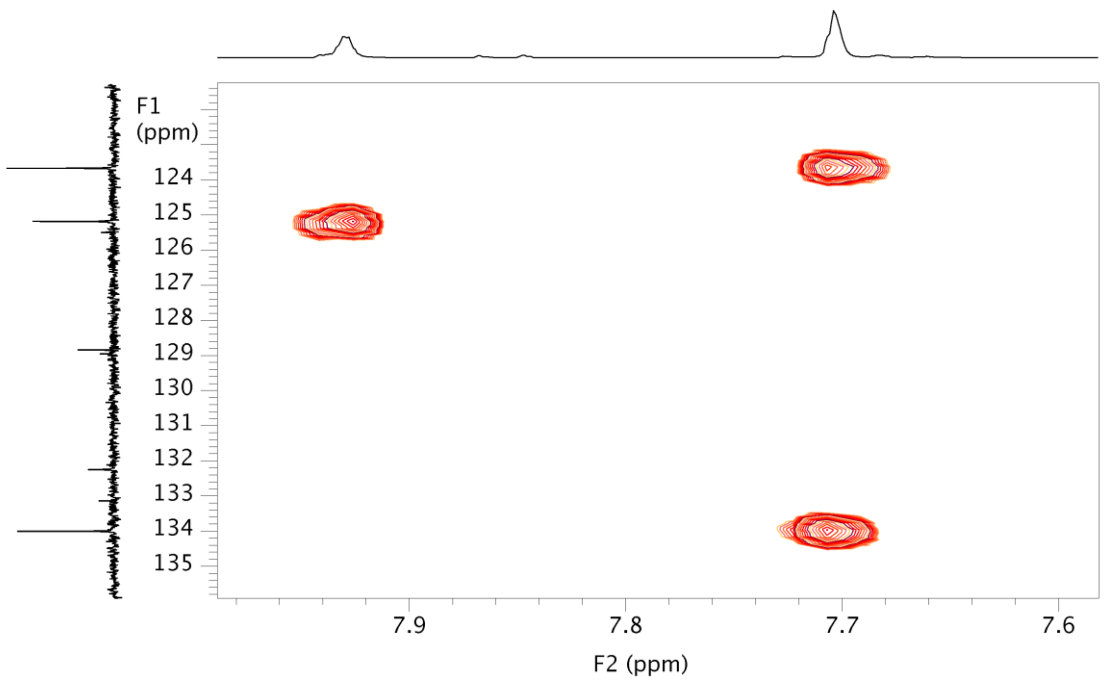

Figure S 44. ^{1}H-^{13}C gc2HSQC (400 MHz, $CDCl_3$) spectrum of compound **2-SO₂**.

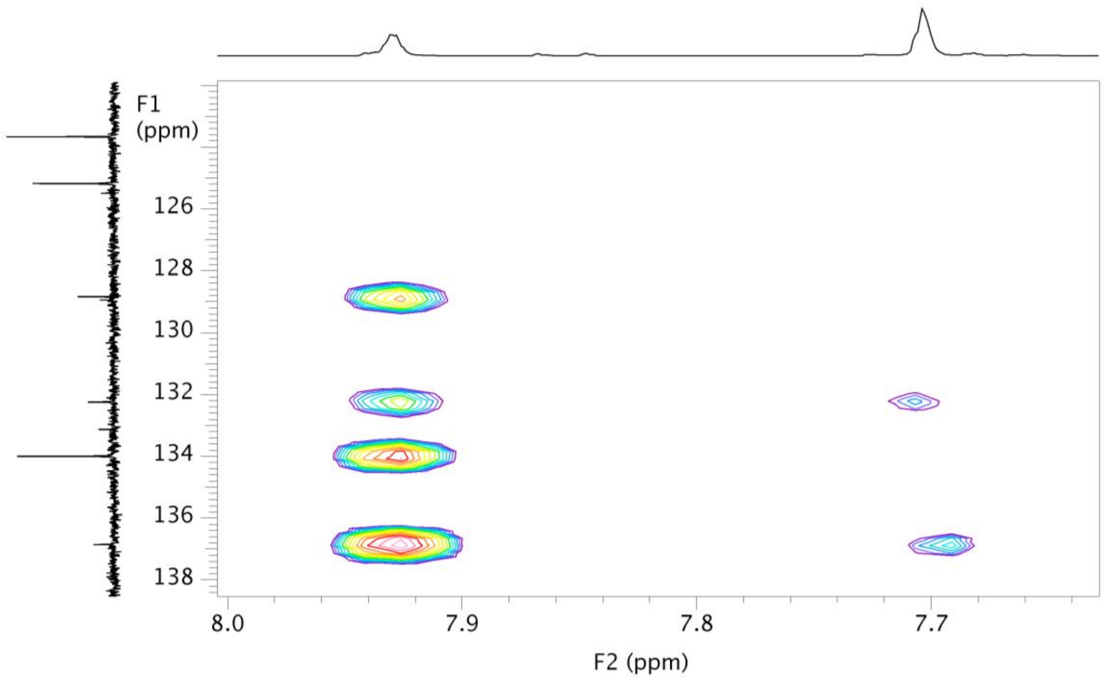

Figure S 45. ^{1}H-^{13}C gc2HMBC (400 MHz, $CDCl_3$) spectrum of compound **2-SO₂**.

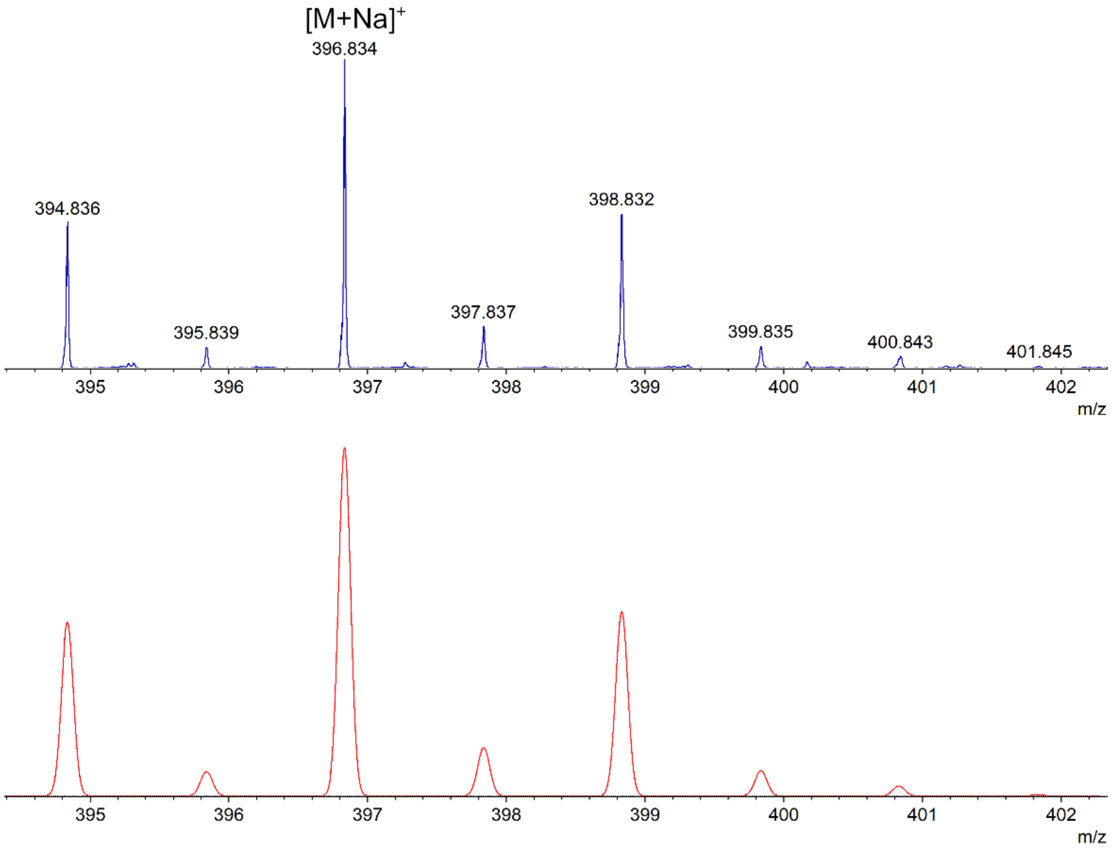

Figure S 46. HRMS (ESI-TOF) of compound **2-SO_2**, $[M+Na]^+$. Calculated (red), measured (blue).

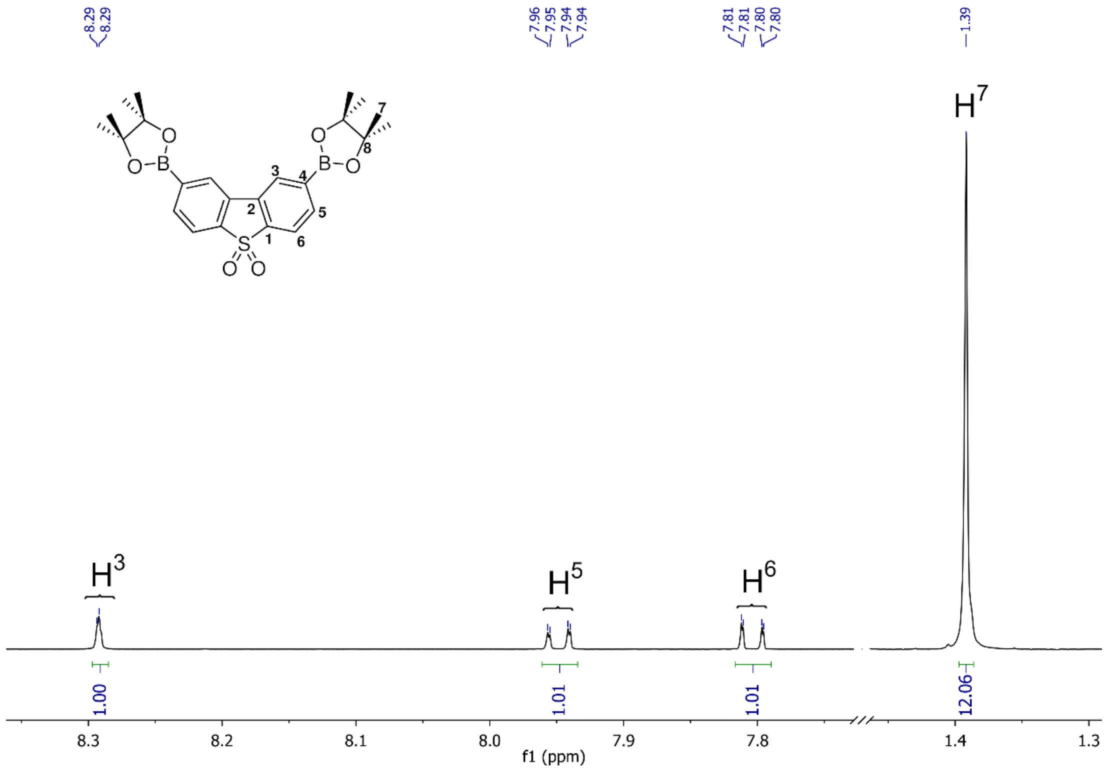

Figure S 47. ^{1}H-NMR (500 MHz, $CDCl_3$) spectrum of compound **3-SO$_2$**.

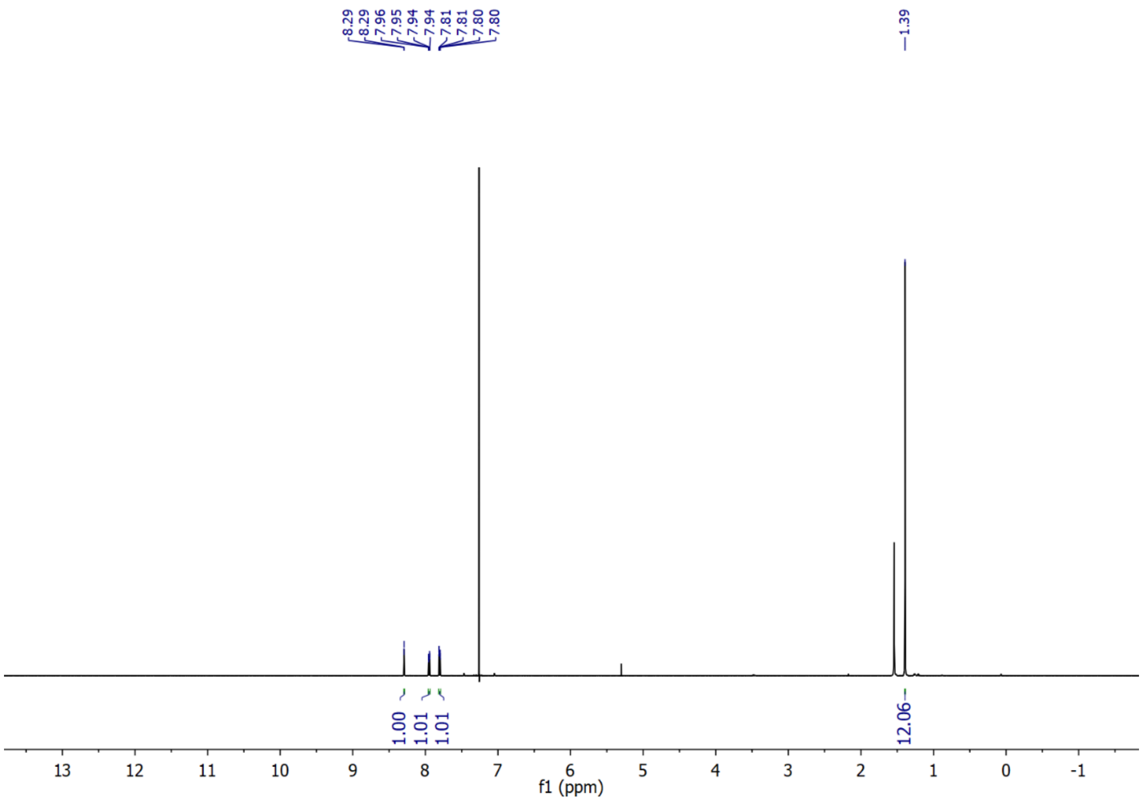

Figure S 48. Full ^{1}H-NMR (500 MHz, $CDCl_3$) spectrum of compound **3-SO$_2$**.

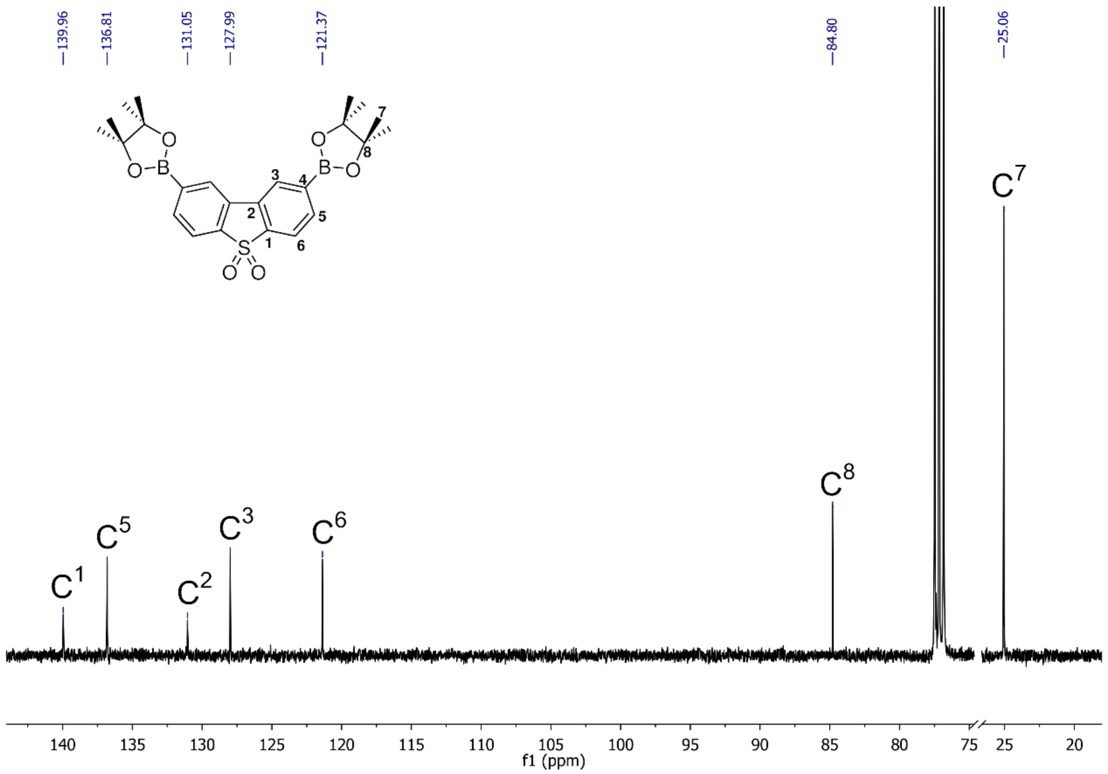

Figure S 49. $^{13}C\{^{1}H\}$-NMR (101 MHz, $CDCl_3$) spectrum of compound **3-SO₂**.

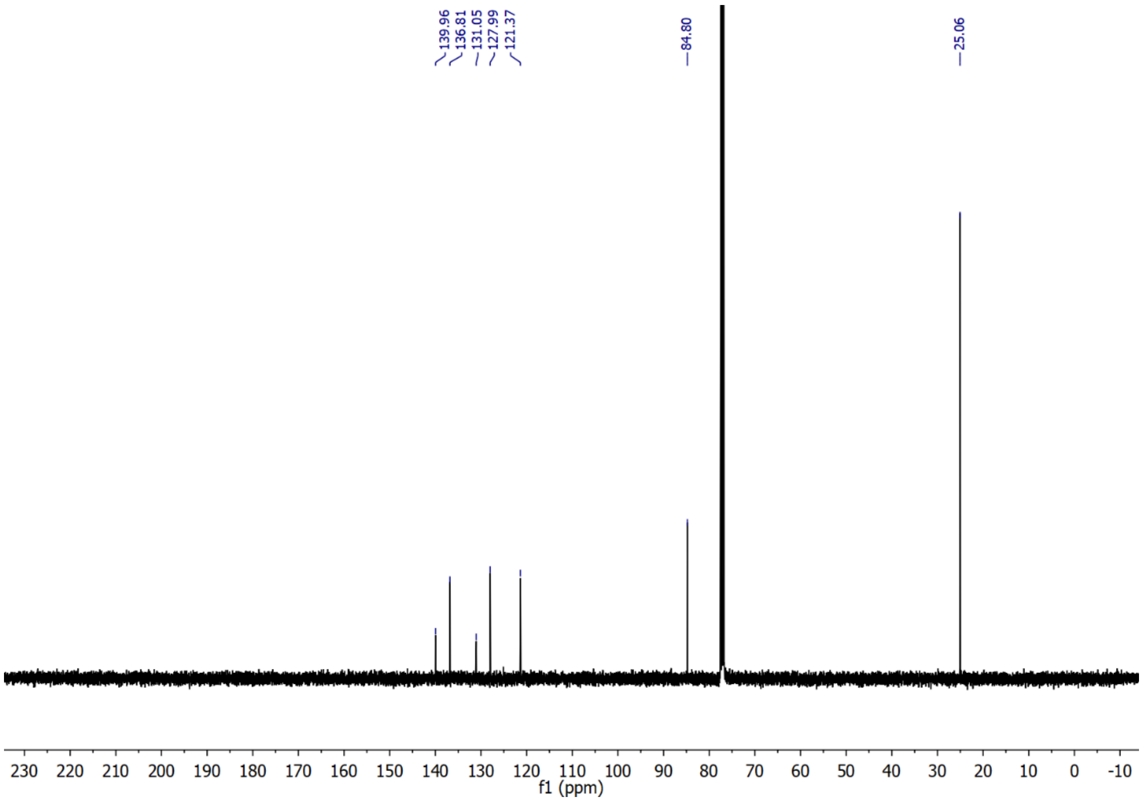

Figure S 50. Full $^{13}C\{^{1}H\}$-NMR (101 MHz, $CDCl_3$) spectrum of compound **3-SO₂**.

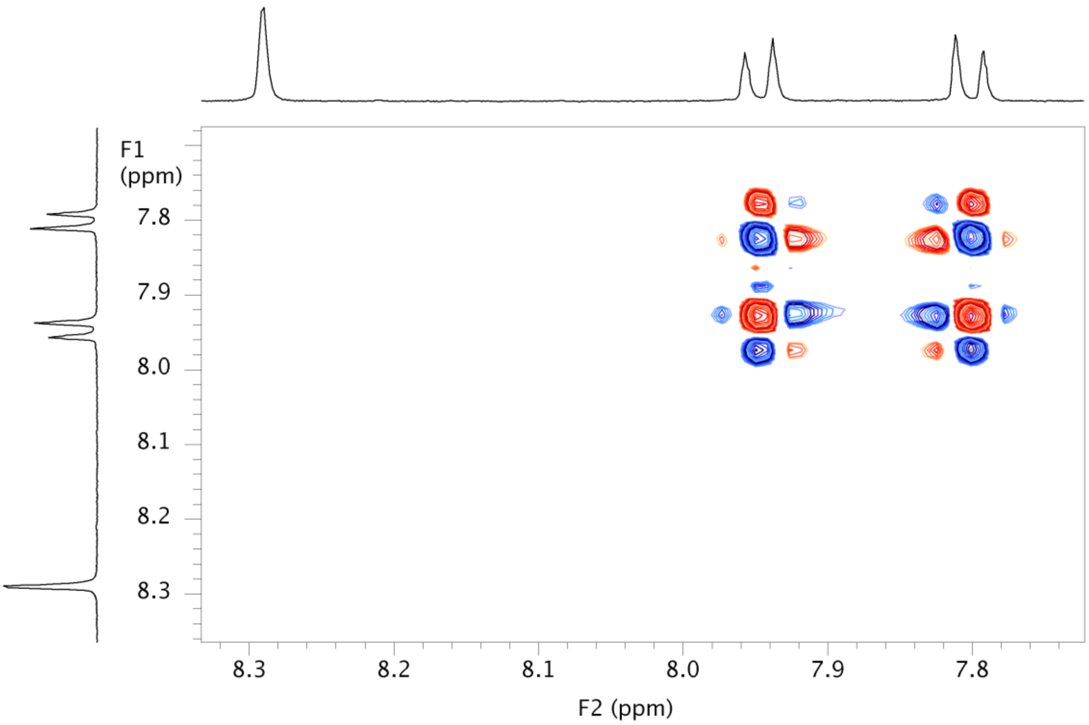

Figure S 51. ^{1}H-^{1}H gDQFCOSY (400 MHz, $CDCl_3$) spectrum of compound **3-SO₂**.

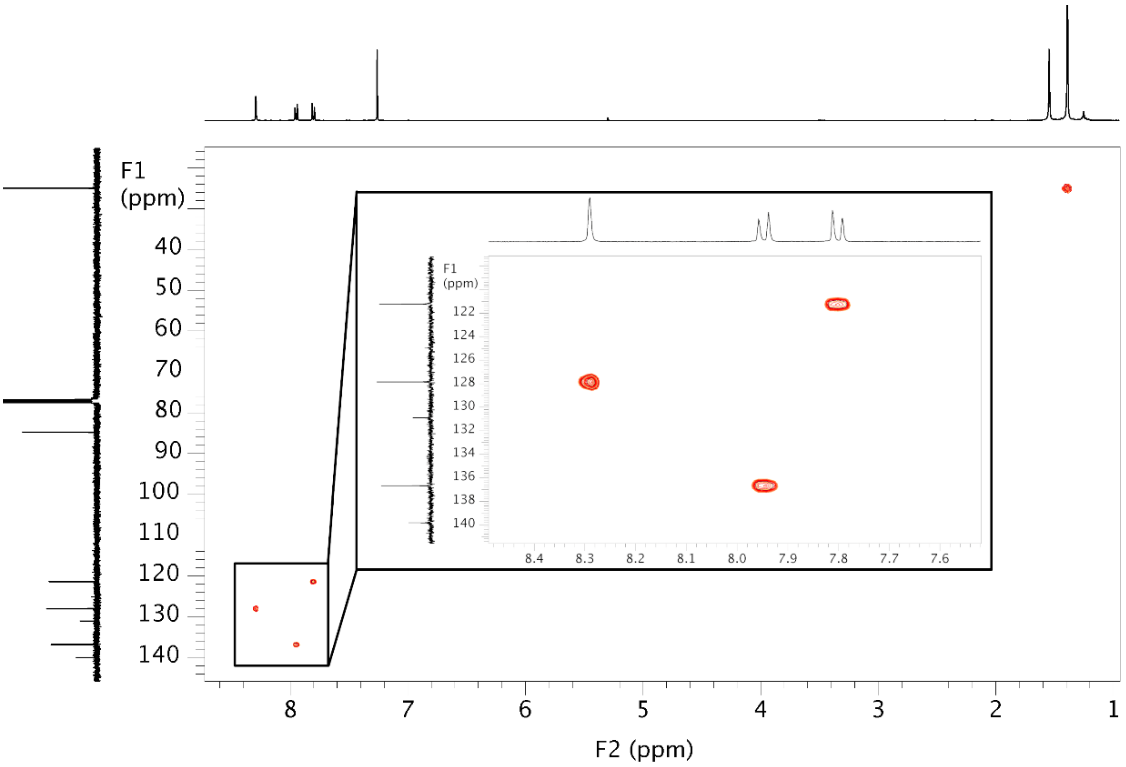

Figure S 52. ^{1}H-^{13}C gc2HSQC (400 MHz, $CDCl_3$) spectrum of compound **3-SO₂**.

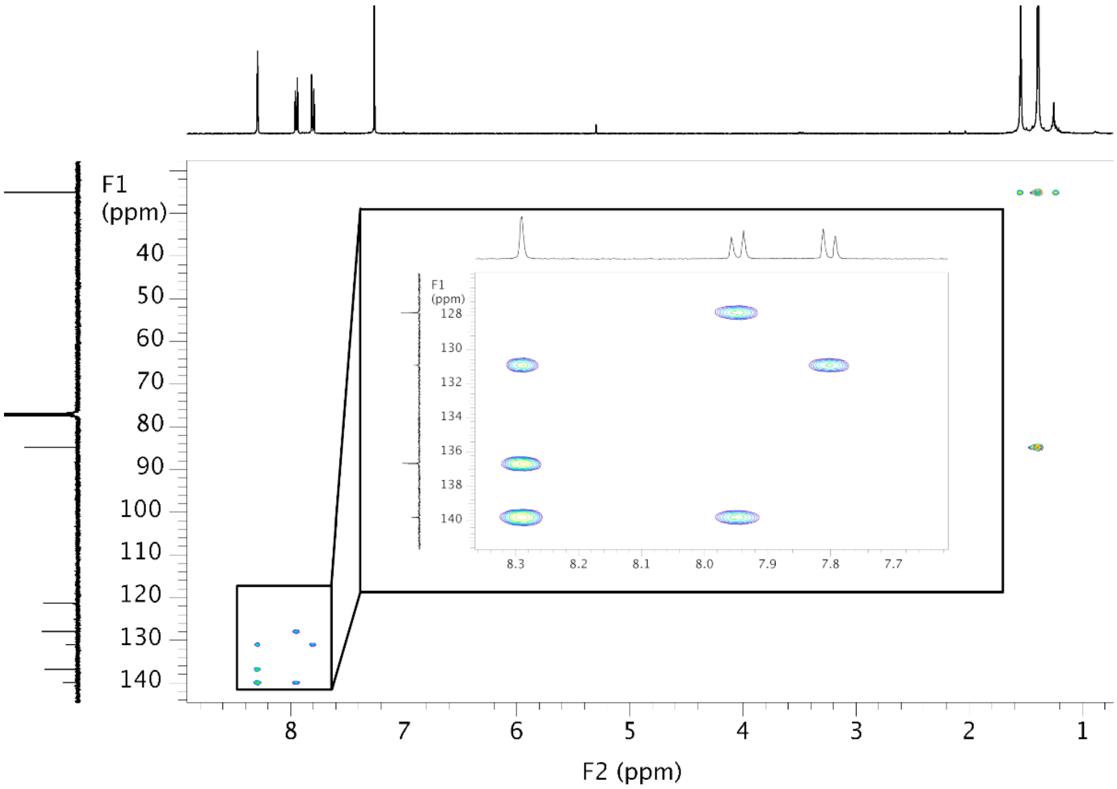

Figure S 53. ^{1}H-^{13}C gc2HMBC (400 MHz, $CDCl_3$) spectrum of compound **3-SO₂**.

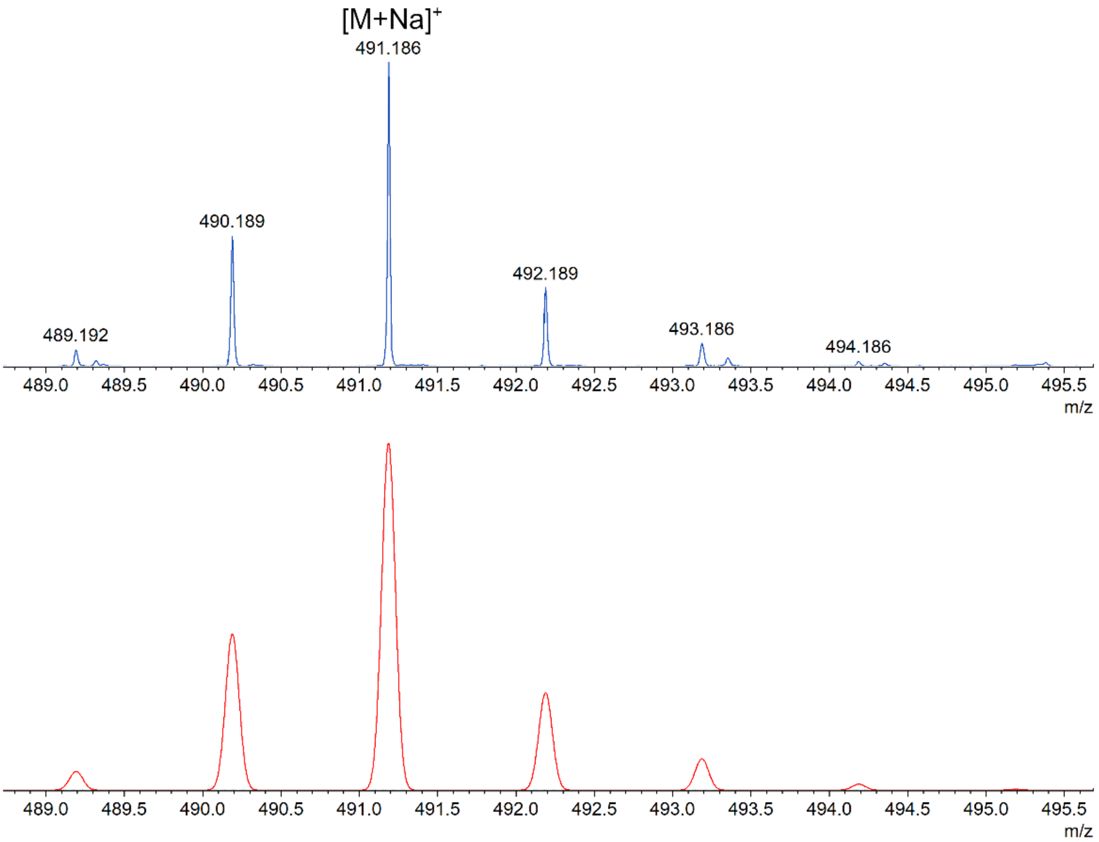

Figure S 54. HRMS (ESI-TOF) of compound **3-SO₂**, $[M+Na]^+$. Calculated (red), measured (blue).

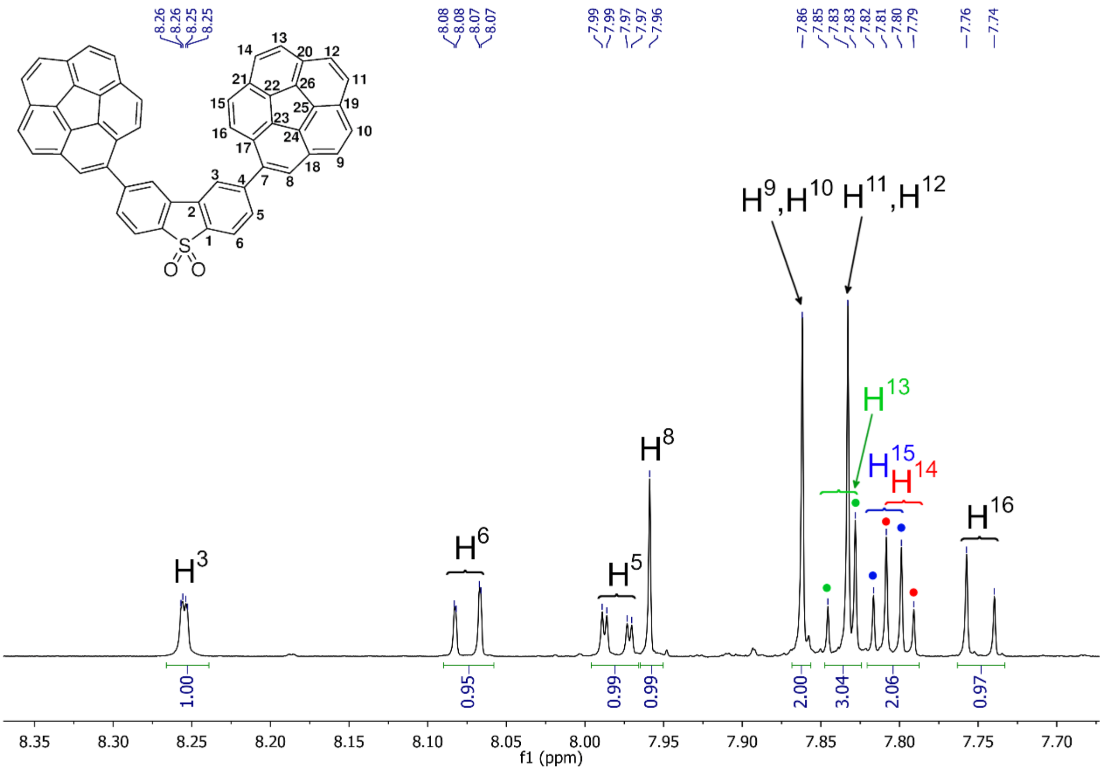

Figure S 55. ^{1}H-NMR (500 MHz, $CDCl_3$) spectrum of compound **4-SO₂**.

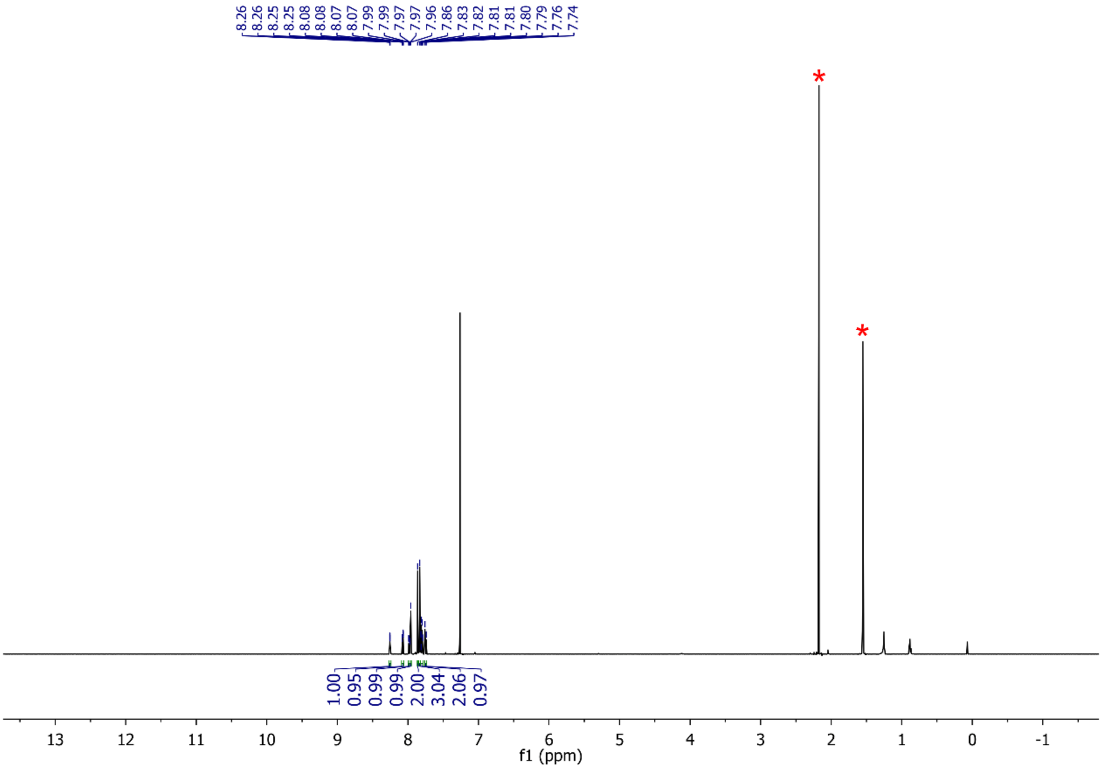

Figure S 56. Full ^{1}H-NMR (500 MHz, $CDCl_3$) spectrum of compound **4-SO₂**. Solvents residual signals (acetone and water) are highlighted with red asterisks)

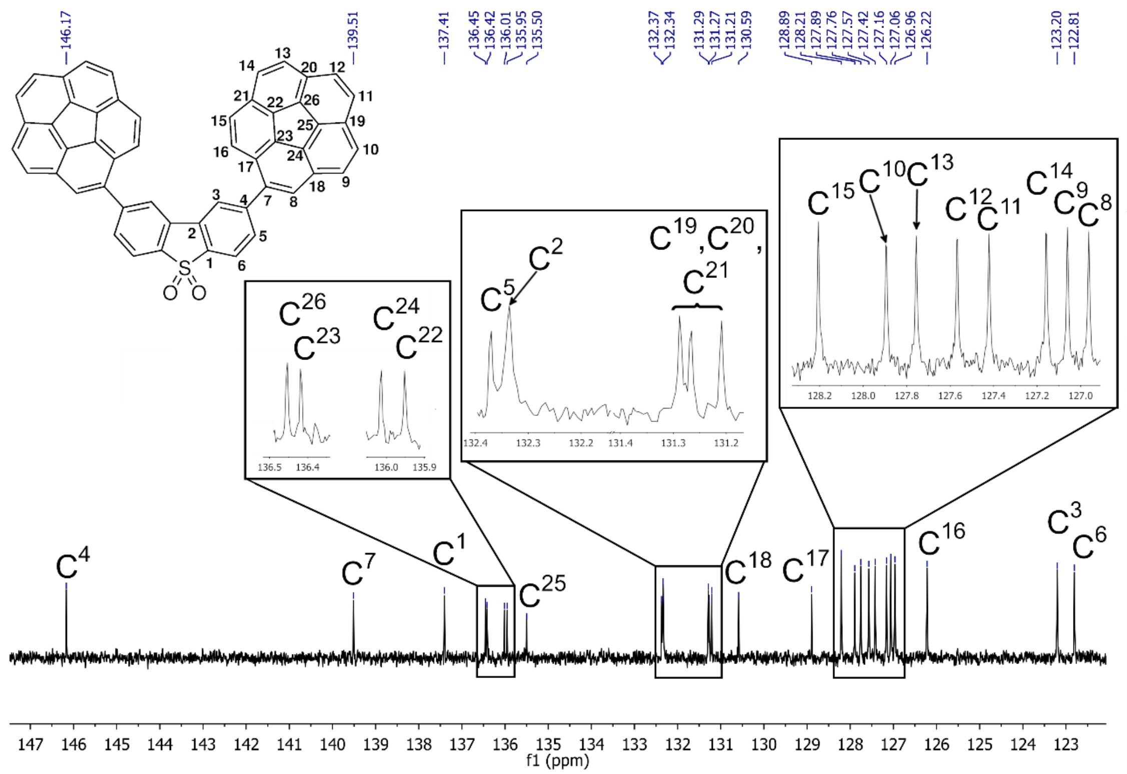

Figure S 57. $^{13}C\{^{1}H\}$-NMR (126 MHz, $CDCl_3$) spectrum of compound **4-SO₂**.

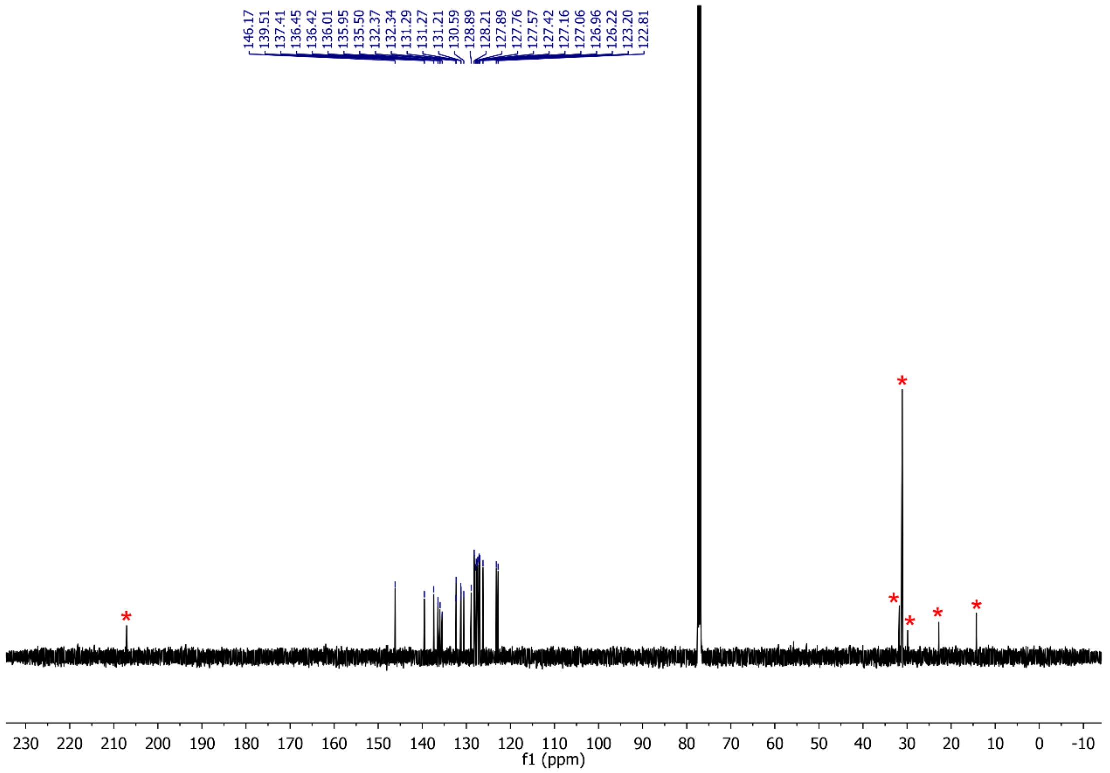

Figure S 58. Full $^{13}C\{^{1}H\}$-NMR (126 MHz, $CDCl_3$) spectrum of compound **4-SO₂**. Solvents residual signals (acetone and hexane) are highlighted with red asterisks.

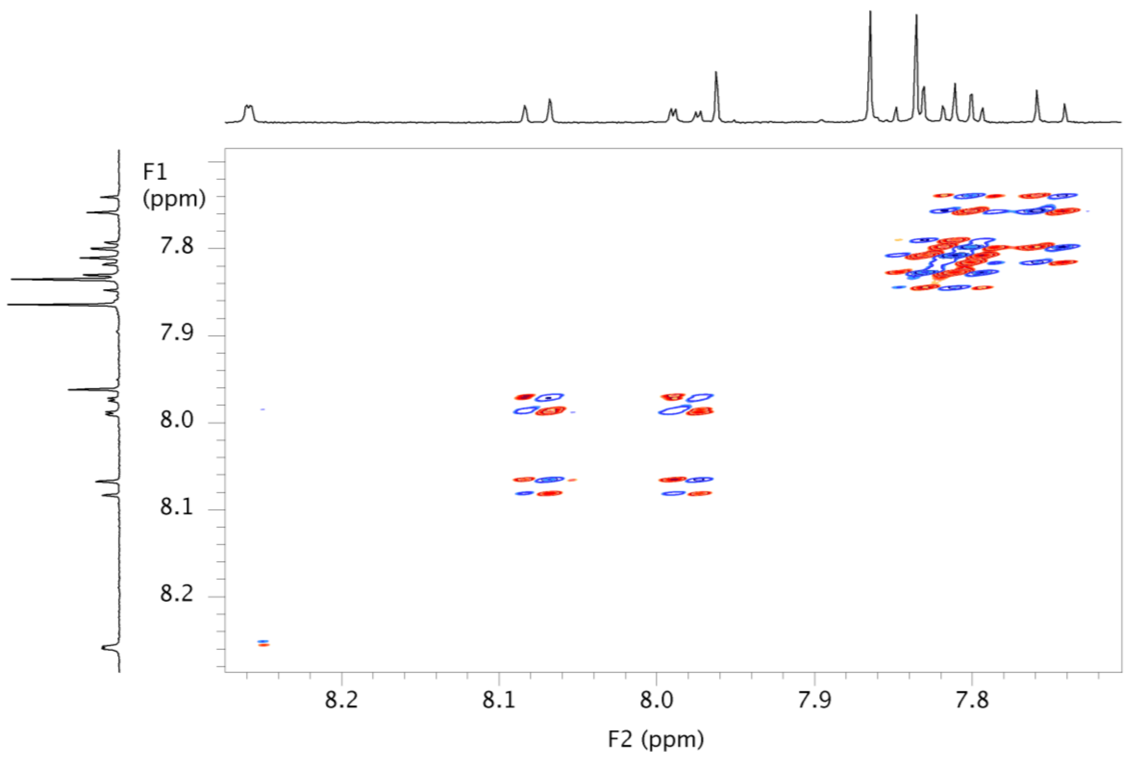

Figure S 59. ^{1}H-^{1}H gDQFCOSY (500 MHz, $CDCl_3$) spectrum of compound **4-SO$_2$**.

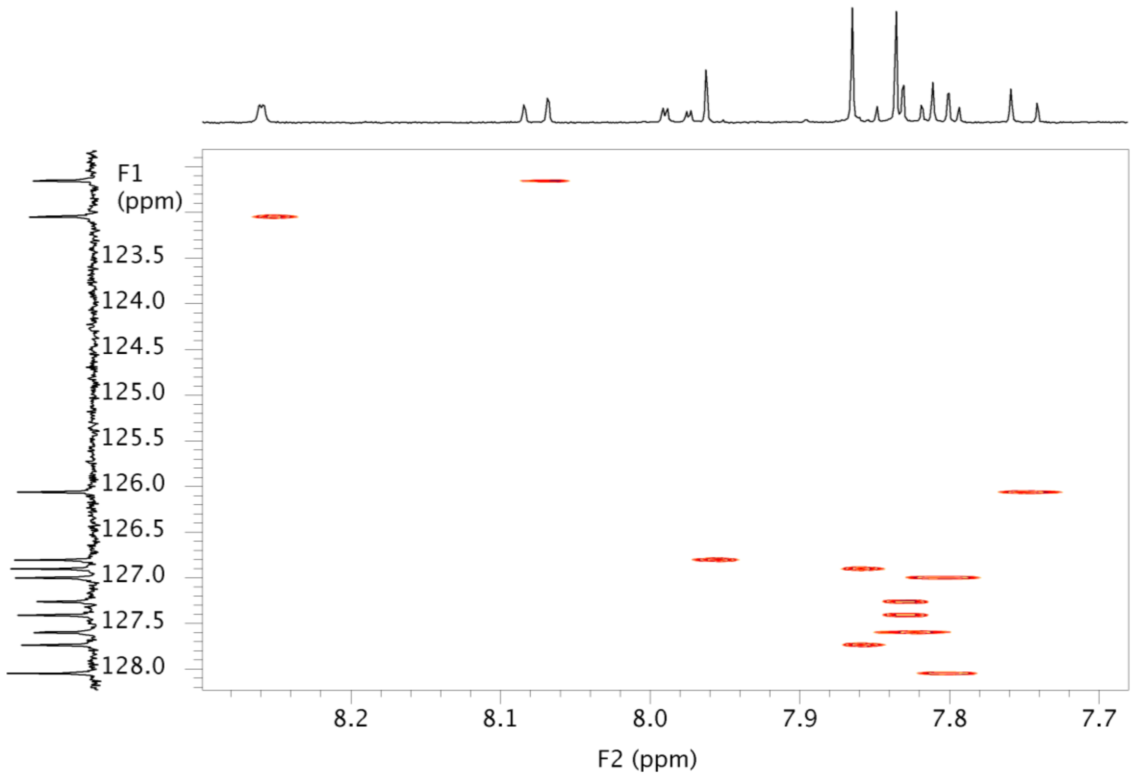

Figure S 60. ^{1}H-^{13}C bsgHSQCAD (500 MHz, $CDCl_3$) spectrum of compound **4-SO$_2$**.

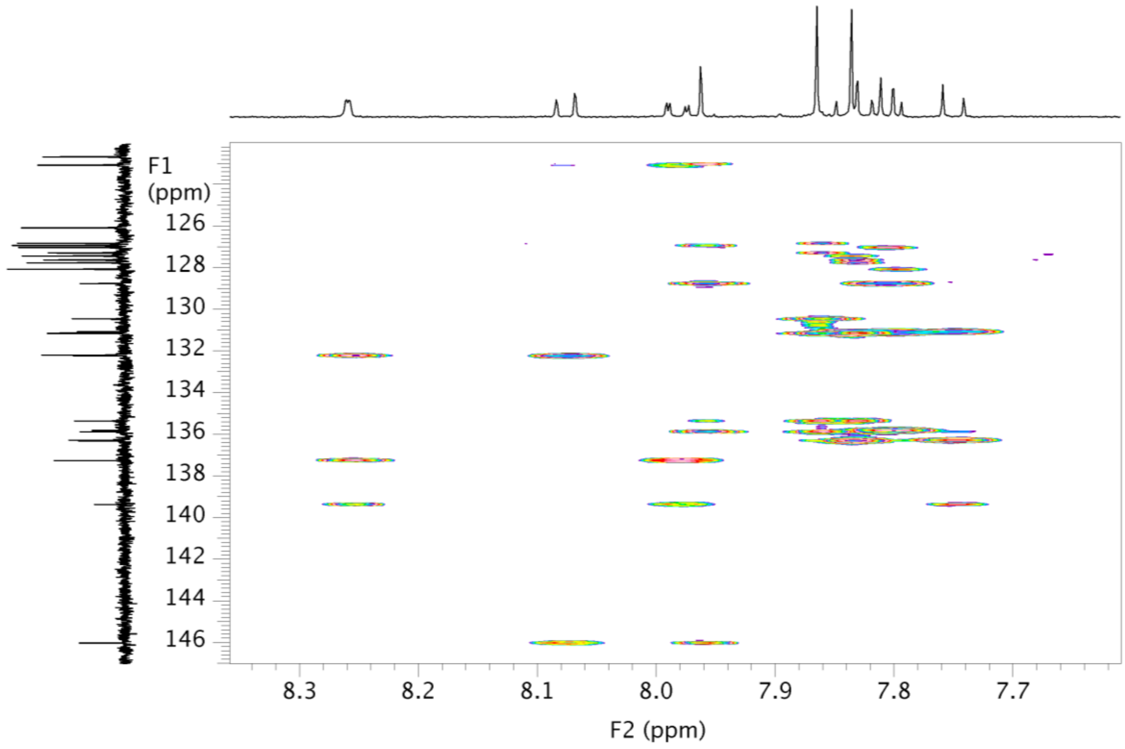

Figure S 61. ^{1}H-^{13}C bsgHMBC (500 MHz, $CDCl_3$) spectrum of compound **4-SO₂**.

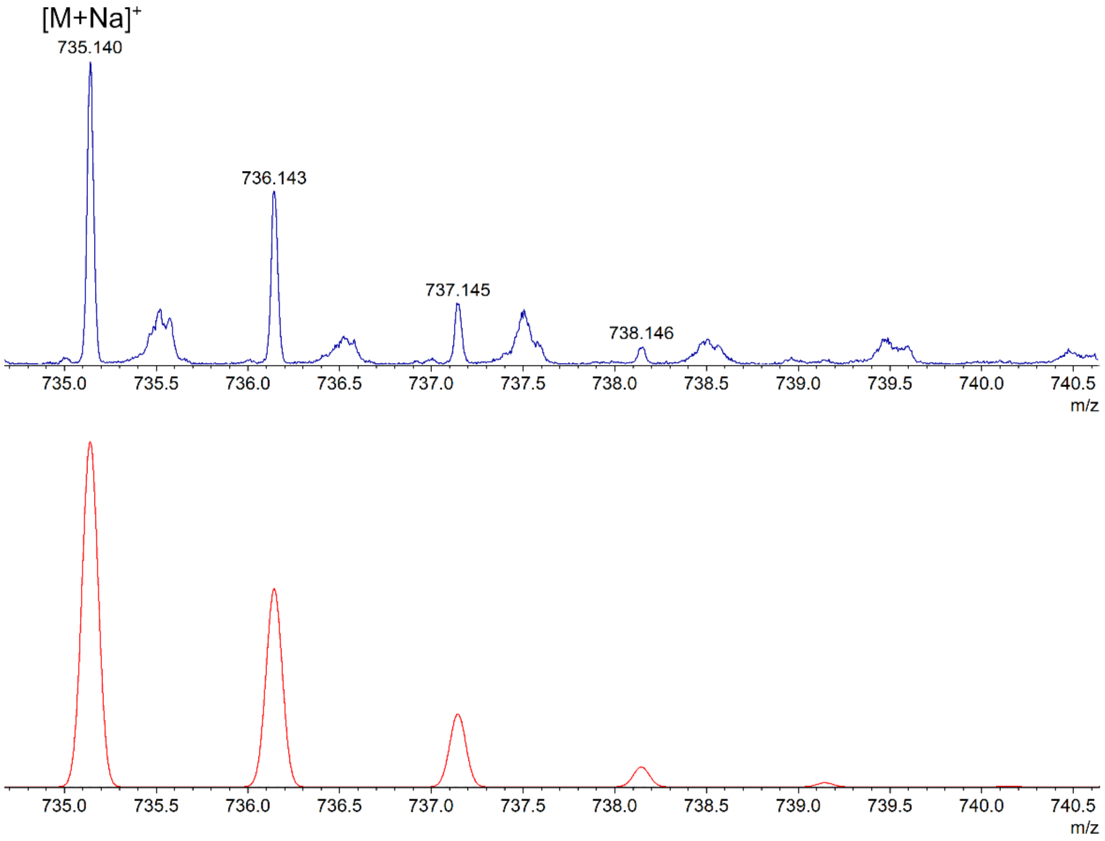

Figure S 62. HRMS (ESI-TOF) of compound **4-SO₂**, $[M+Na]^+$. Calculated (red), measured (blue).

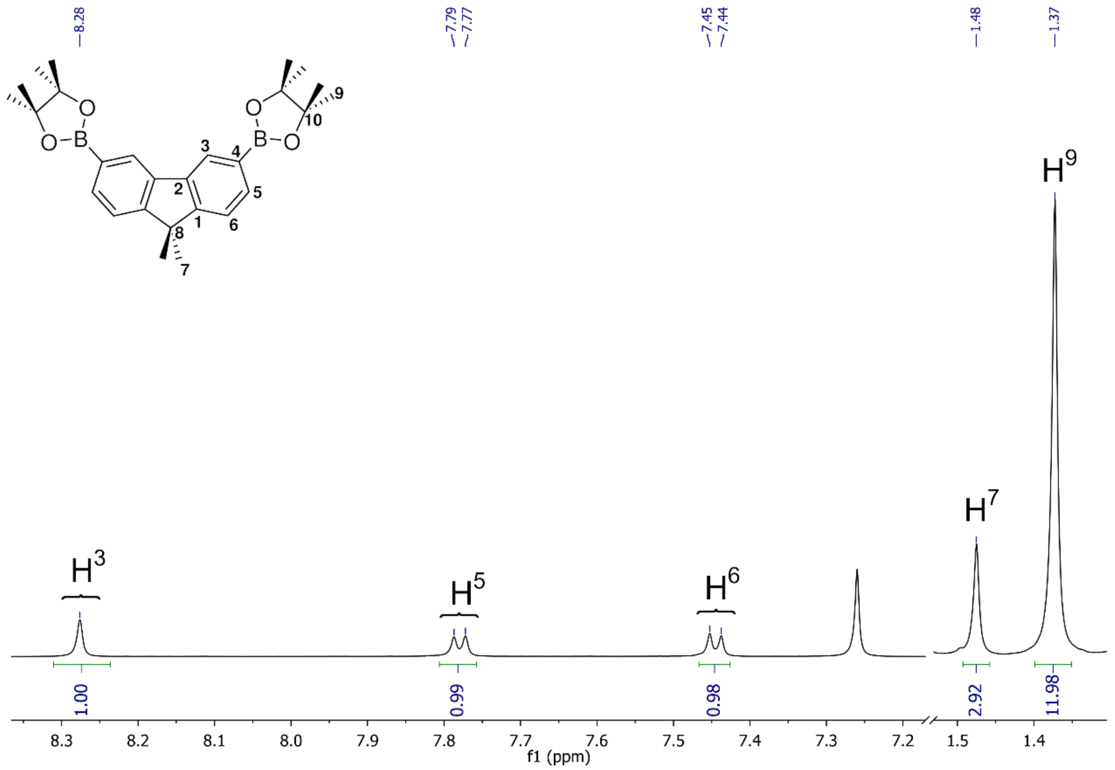

Figure S 63. ^{1}H-NMR (500 MHz, $CDCl_3$) spectrum of compound **10-CMe$_2$**.

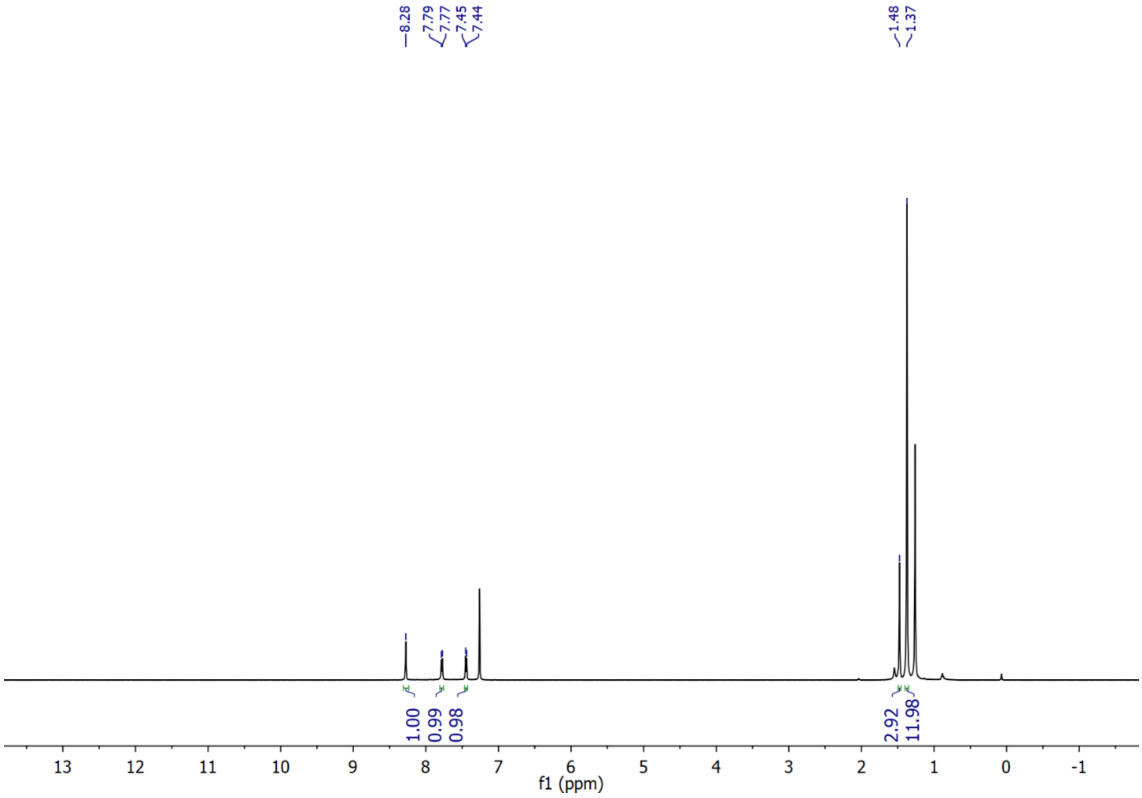

Figure S 64. Full ^{1}H-NMR (500 MHz, $CDCl_3$) spectrum of compound **10-CMe$_2$**.

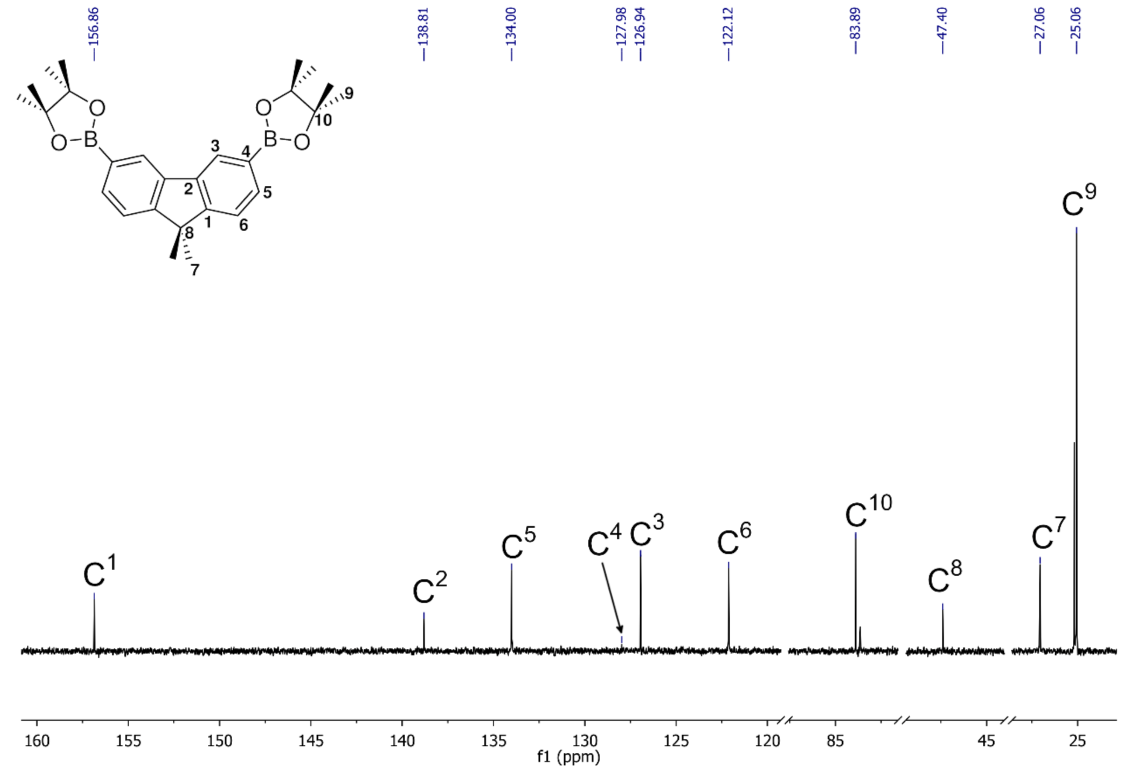

Figure S 65. $^{13}C\{^{1}H\}$-NMR (101 MHz, $CDCl_3$) spectrum of compound **10-CMe$_2$**.

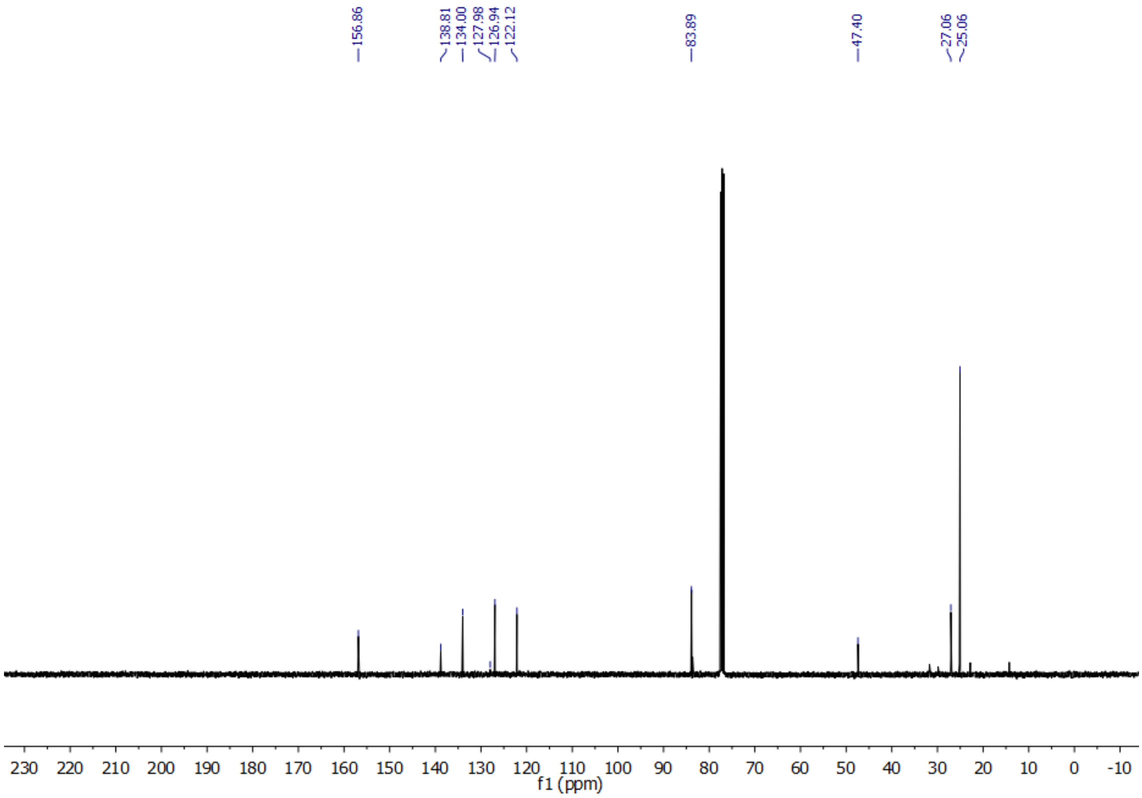

Figure S 66. Full $^{13}C\{^{1}H\}$-NMR (101 MHz, $CDCl_3$) spectrum of compound **10-CMe$_2$**.

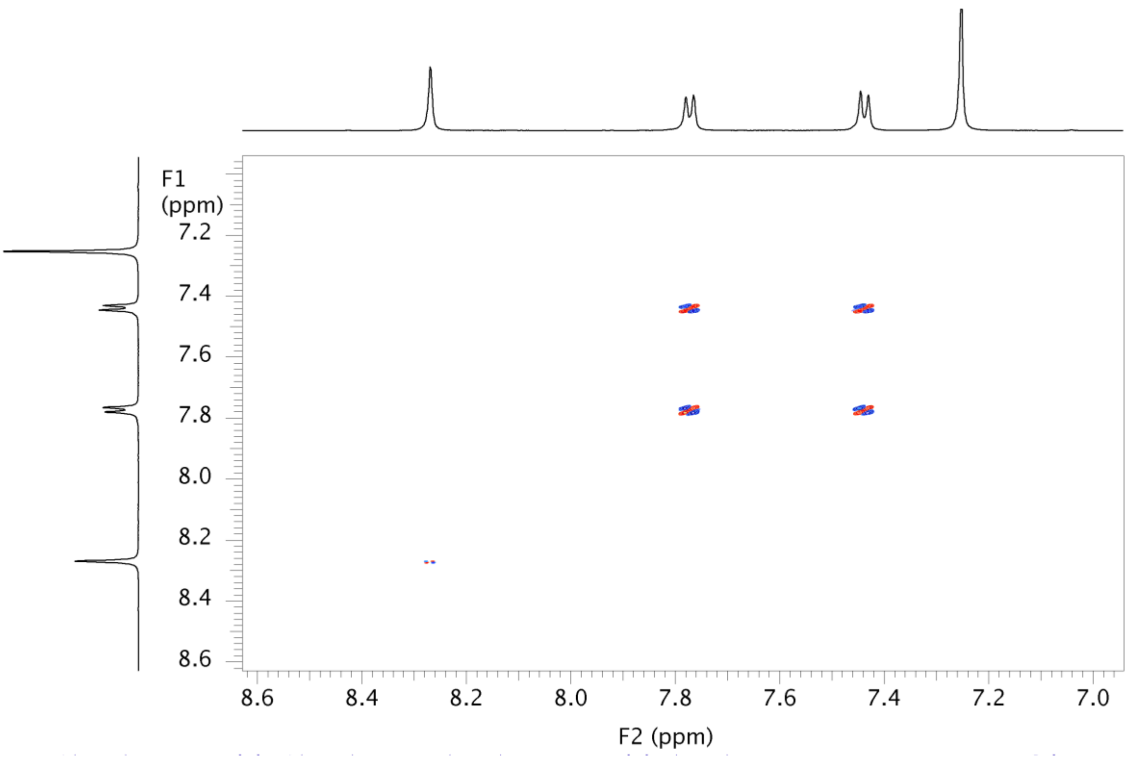

Figure S 67. ^{1}H-^{1}H gDQFCOSY (500 MHz, $CDCl_3$) spectrum of compound **10-CMe₂**.

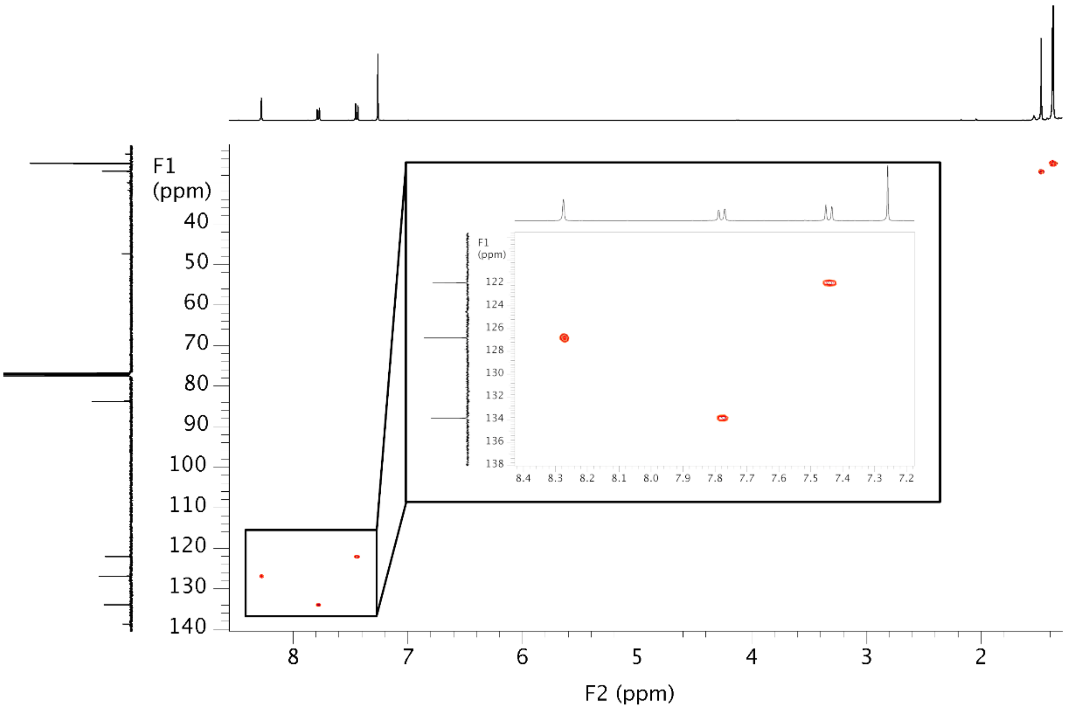

Figure S 68. ^{1}H-^{13}C gc2HSQC (400 MHz, $CDCl_3$) spectrum of compound **10-CMe₂**.

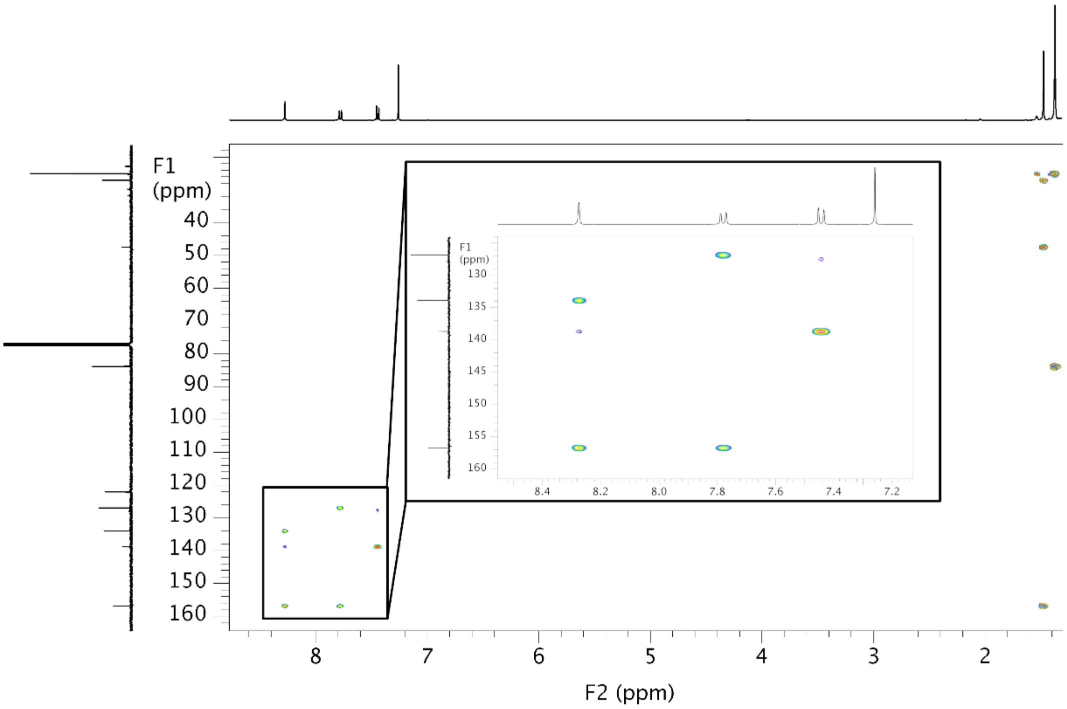

Figure S 69. ^{1}H-^{13}C gc2HMBC (400 MHz, $CDCl_3$) spectrum of compound **10-CMe₂**.

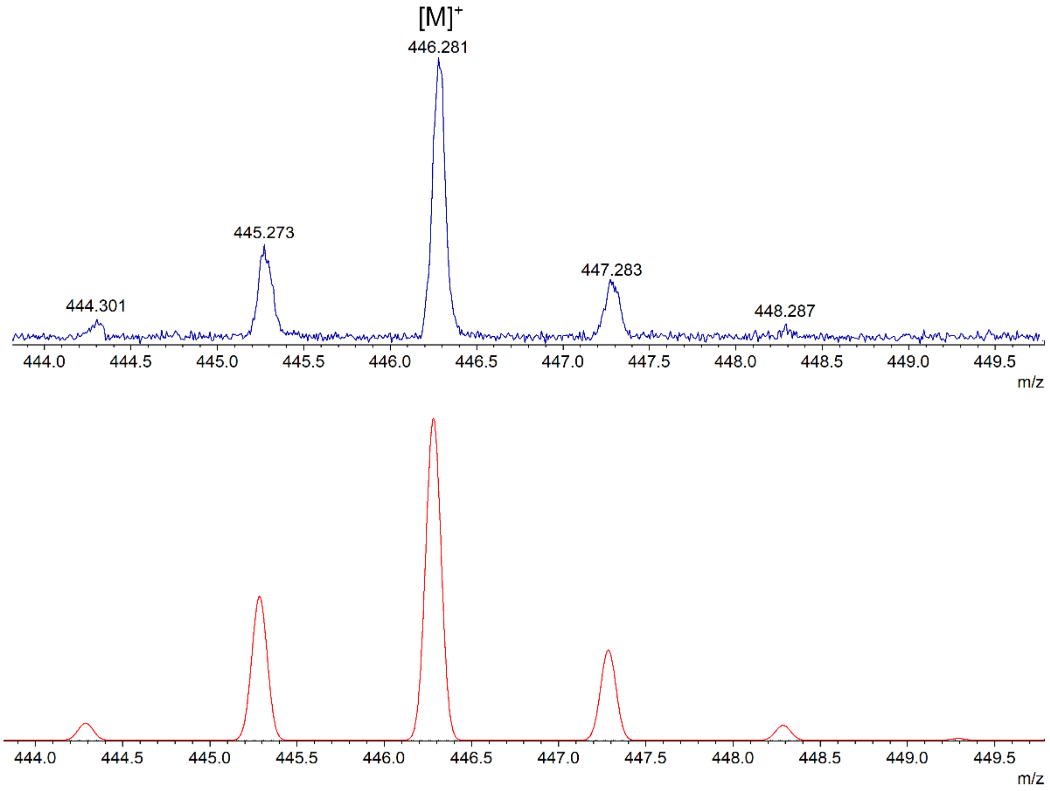

Figure S 70. HRMS (MALDI-TOF) of compound **10-CMe₂**, $[M]^+$. Calculated (red), measured (blue).

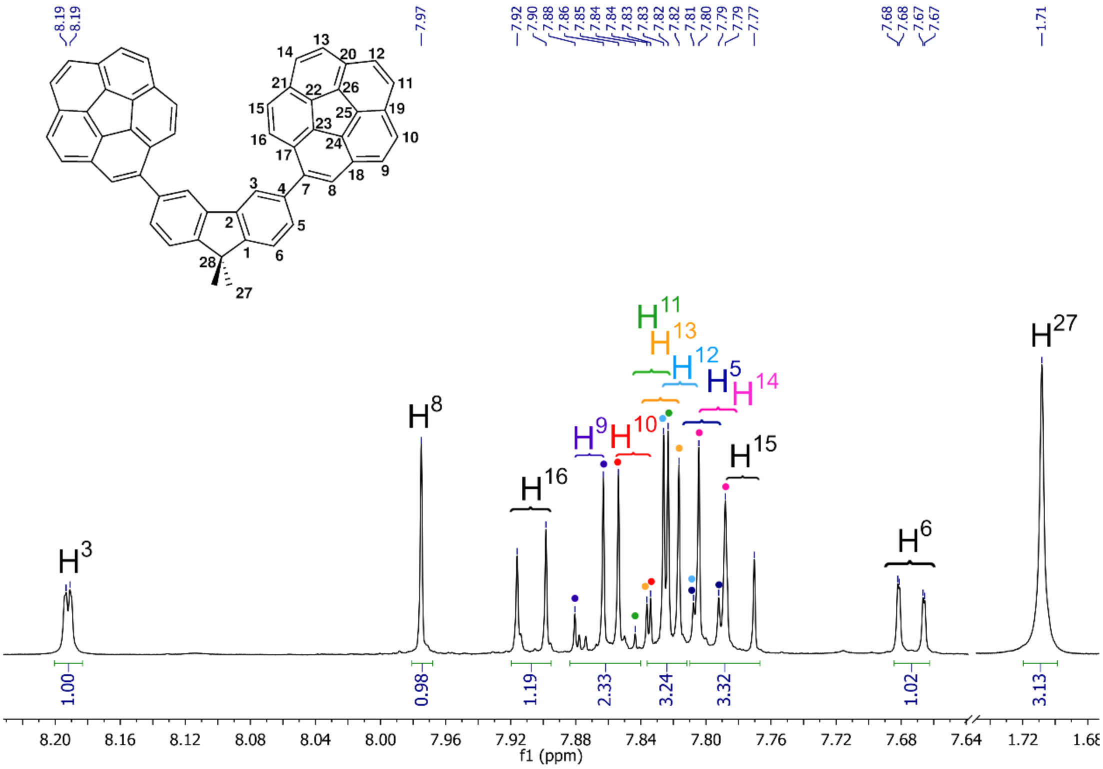

Figure S 71. ^{1}H-NMR (500 MHz, $CDCl_3$) spectrum of compound **11-CMe$_2$**.

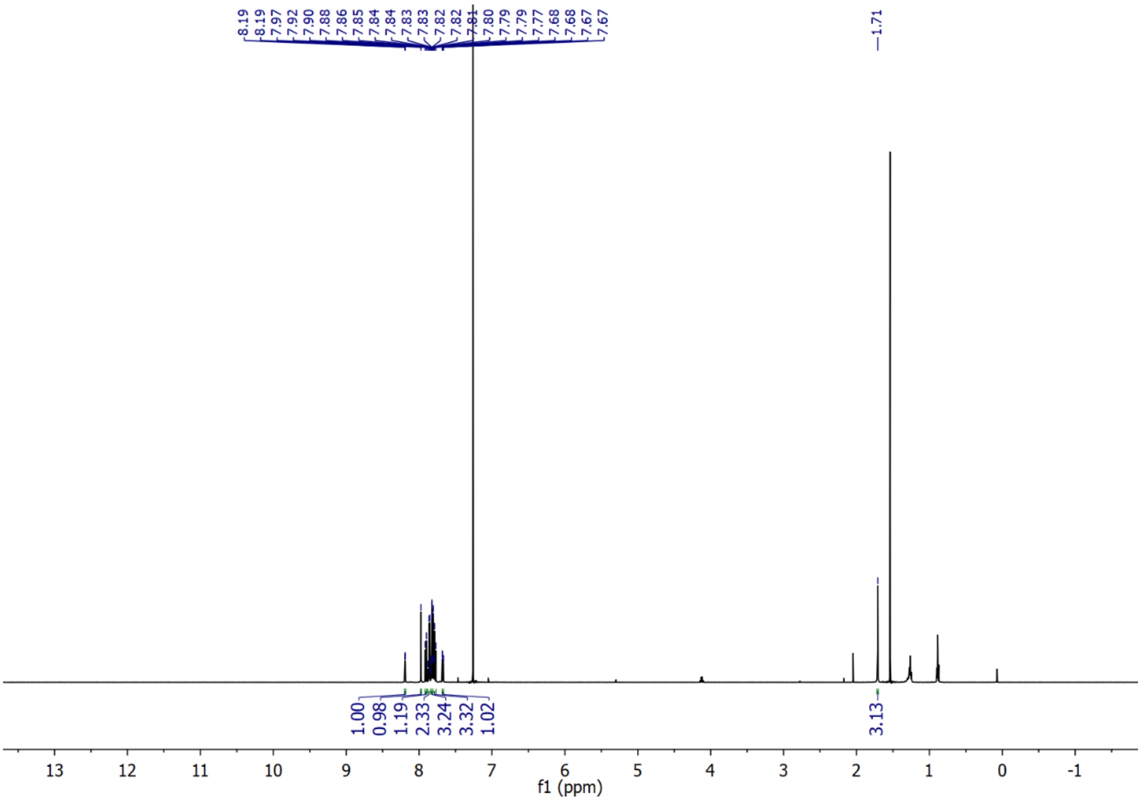

Figure S 72. Full ^{1}H-NMR (500 MHz, $CDCl_3$) spectrum of compound **11-CMe$_2$**.

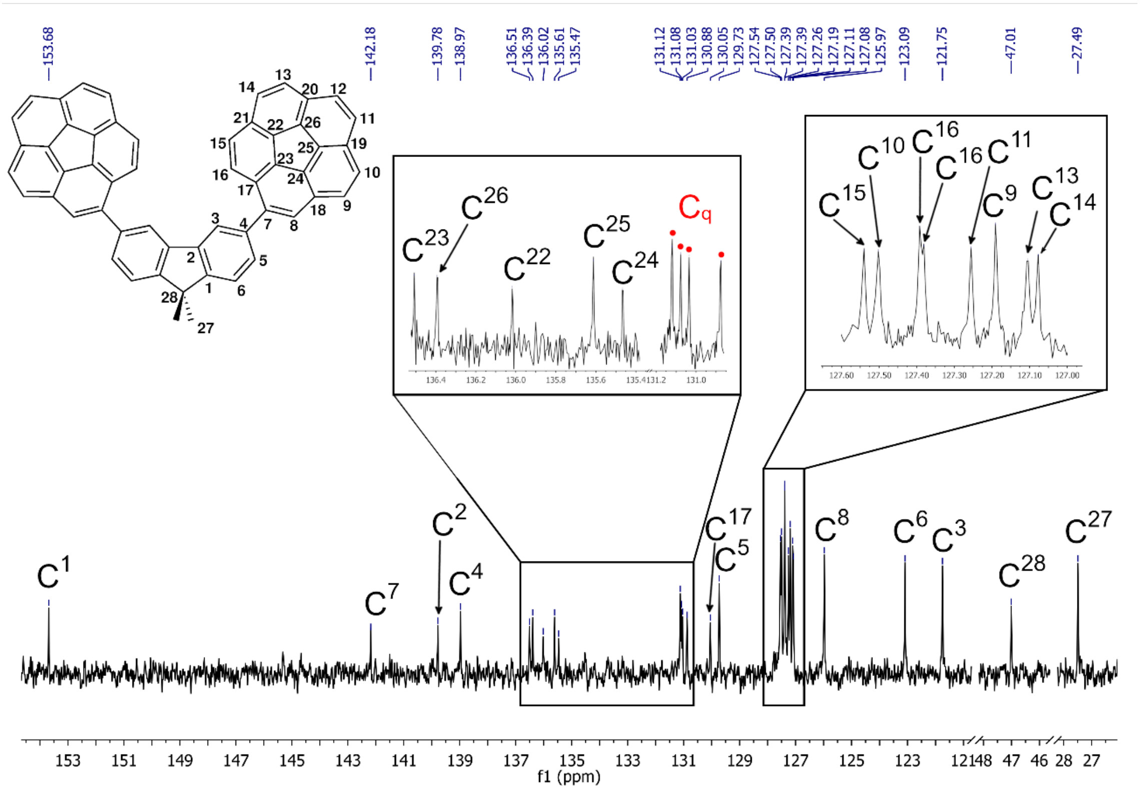

Figure S 73. $^{13}C\{^{1}H\}$-NMR (126 MHz, $CDCl_3$) spectrum of compound **11-CMe₂**.

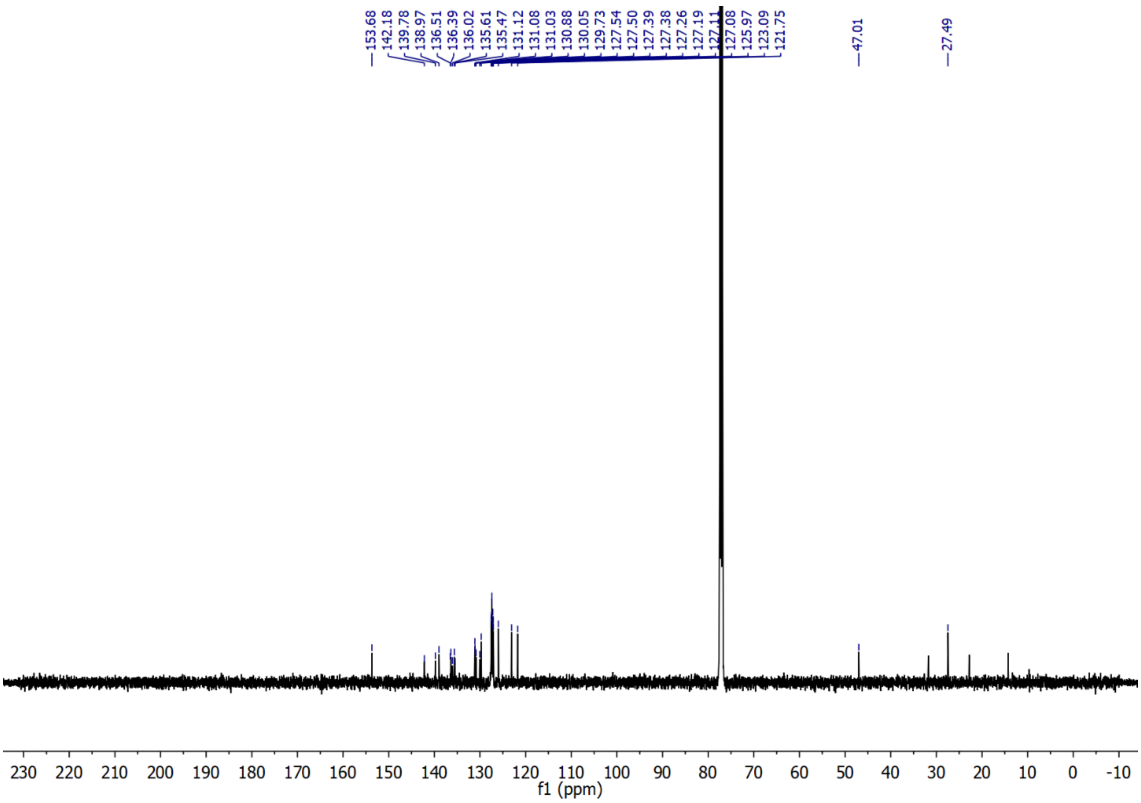

Figure S 74. Full $^{13}C\{^{1}H\}$-NMR (126 MHz, $CDCl_3$) spectrum of compound **11-CMe₂**.

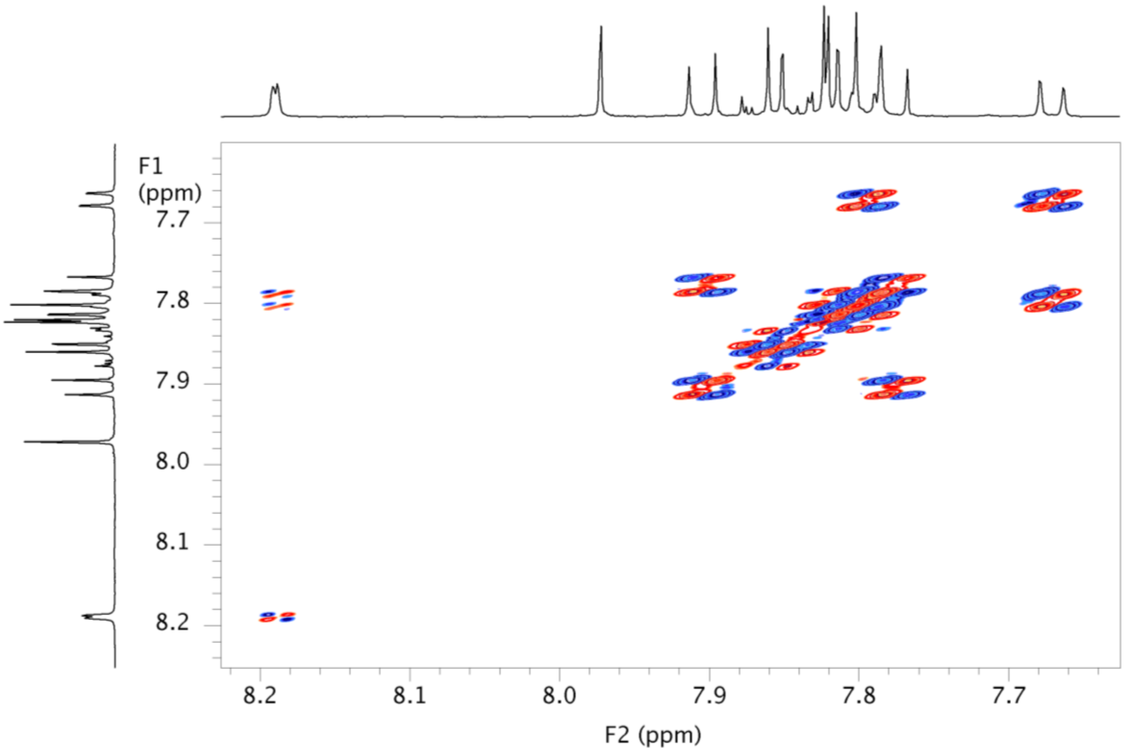

Figure S 75. ^{1}H-^{1}H gDQFCOSY (500 MHz, $CDCl_3$) spectrum of compound **11-CMe$_2$**.

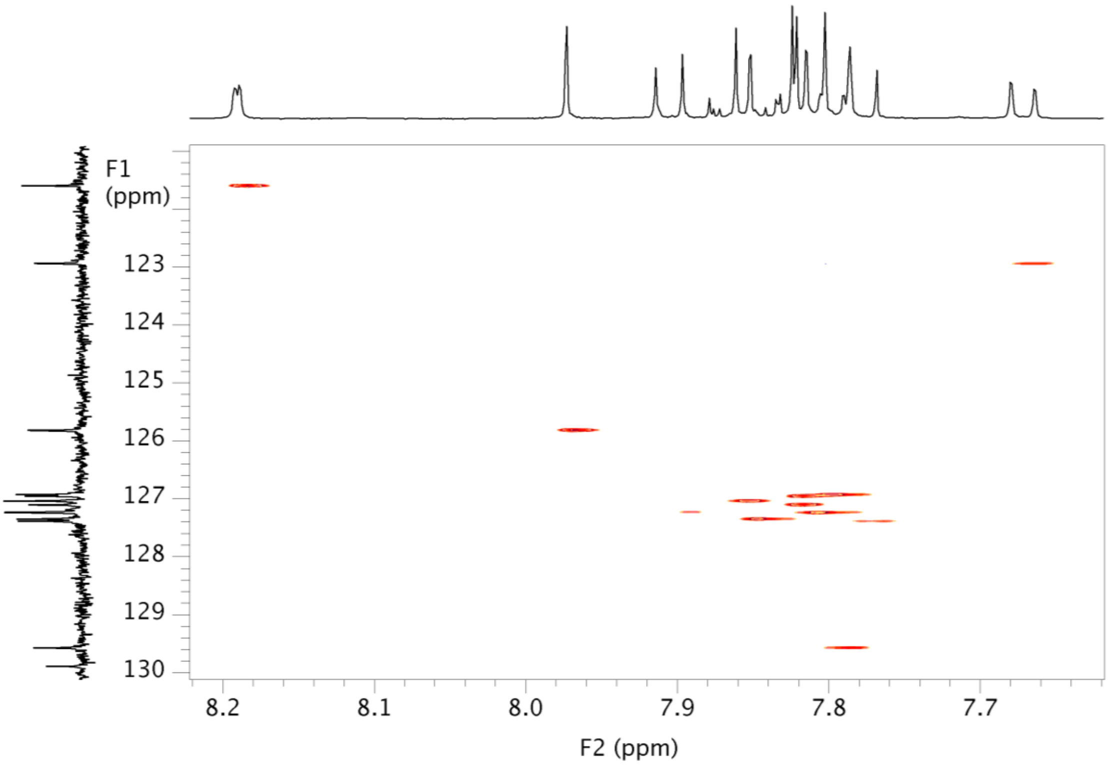

Figure S 76. ^{1}H-^{13}C bsgHSQC (500 MHz, $CDCl_3$) spectrum of compound **11-CMe$_2$**.

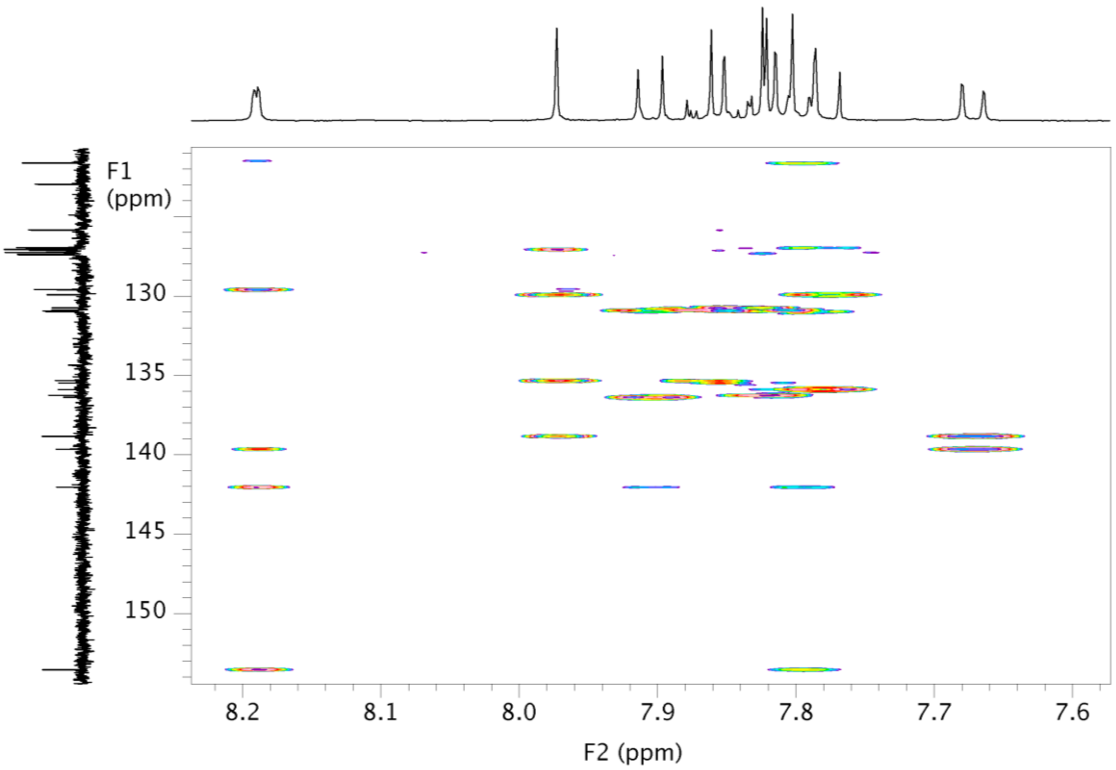

Figure S 77. ^{1}H-^{13}C bsgHMBC (500 MHz, $CDCl_3$) spectrum of compound **11-CMe₂**.

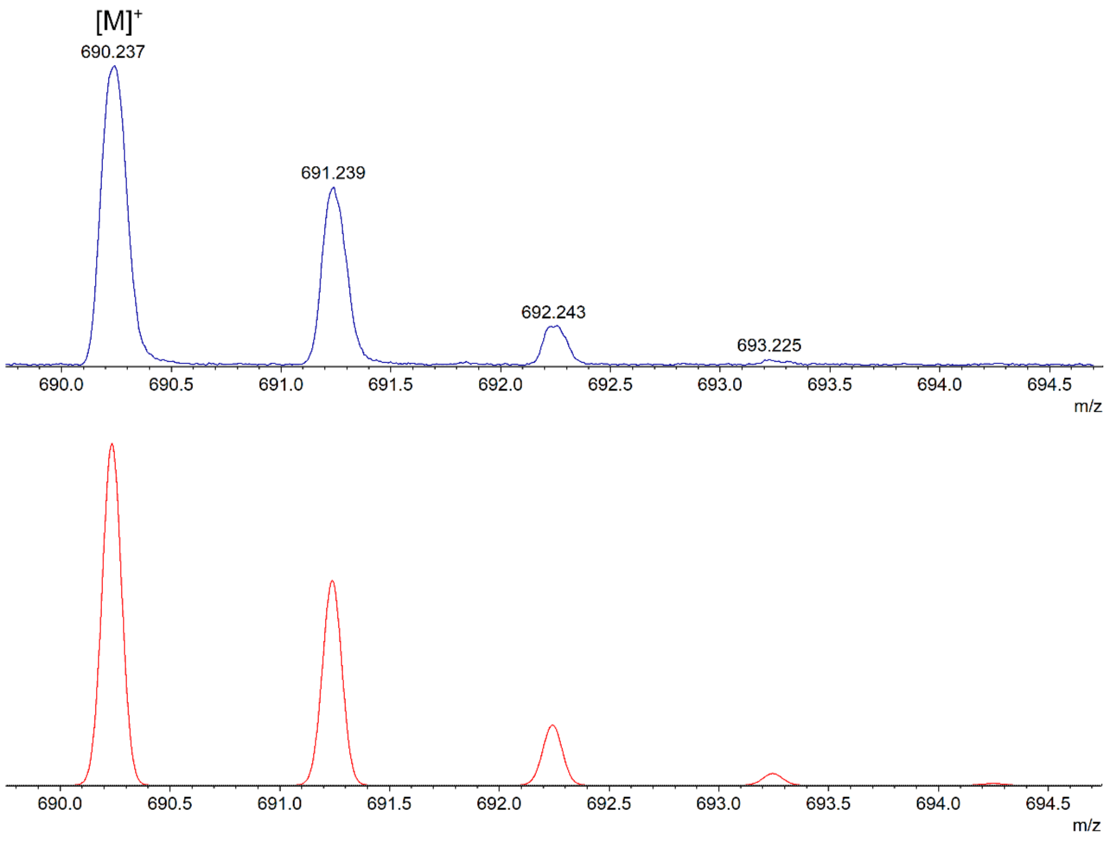

Figure S 78. HRMS (MALDI-TOF) of compound **11-CMe$_2$**, $[M]^+$. Calculated (red), measured (blue).

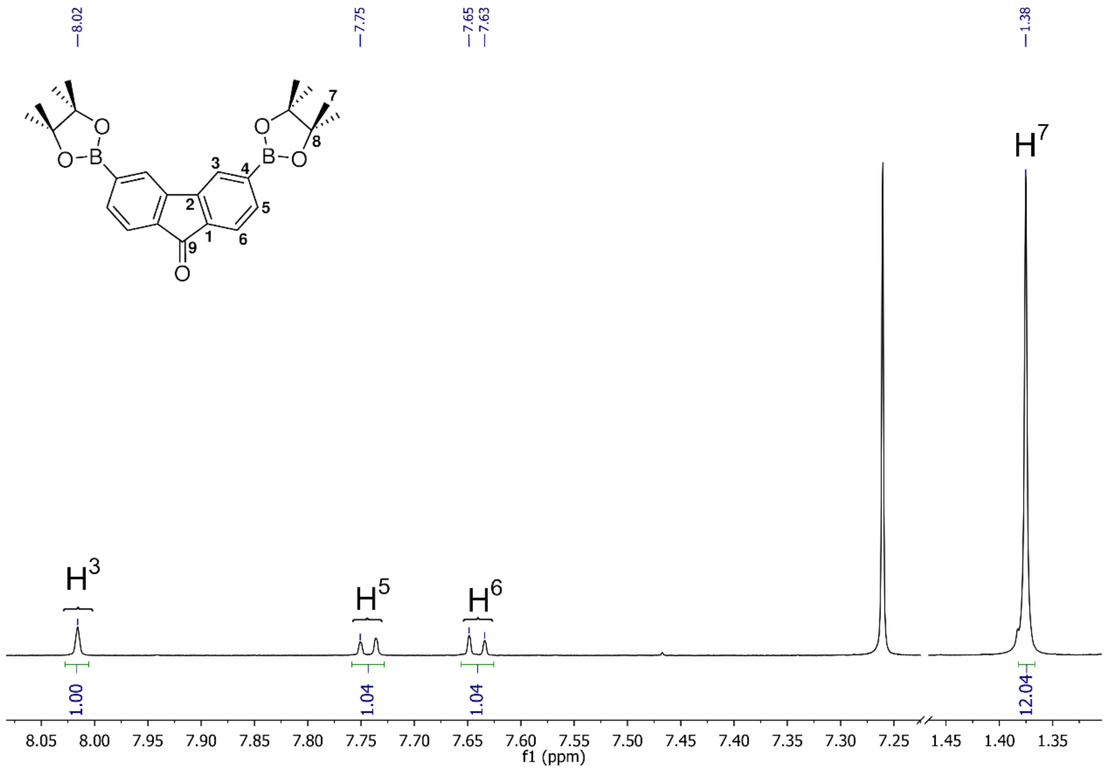

Figure S 79. ^{1}H-NMR (400 MHz, $CDCl_3$) spectrum of compound **10-CO**.

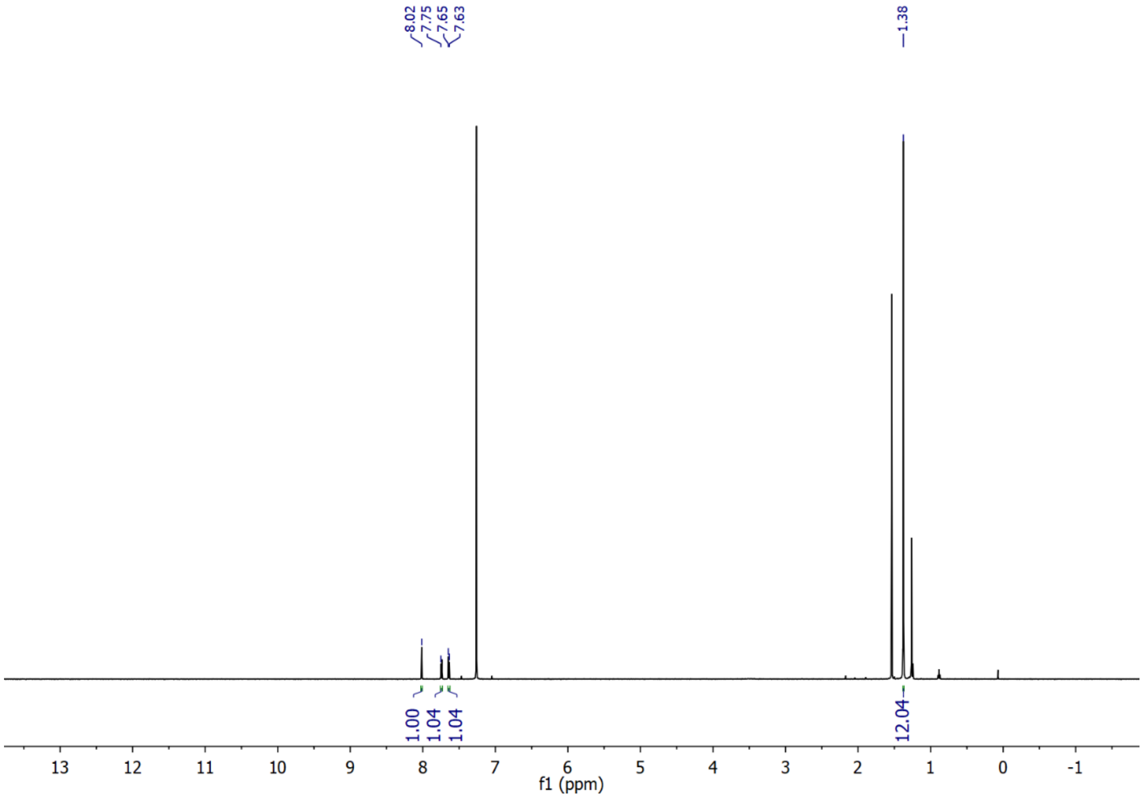

Figure S 80. Full ^{1}H-NMR (400 MHz, $CDCl_3$) spectrum of compound **10-CO**.

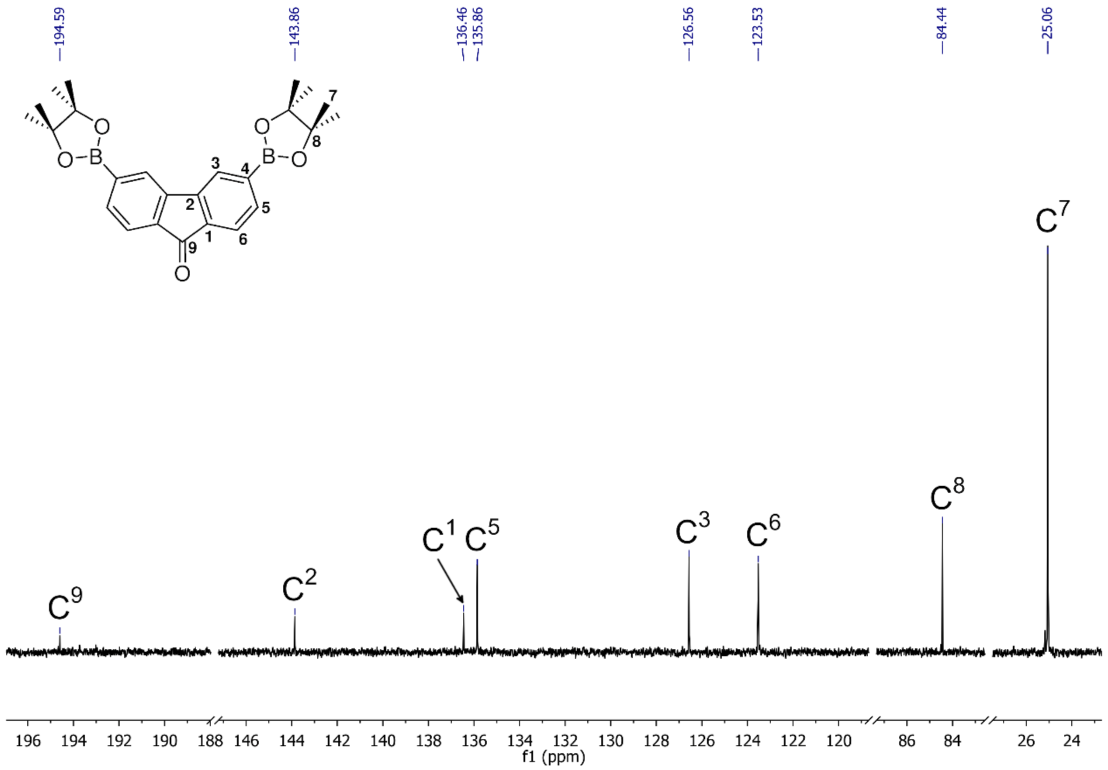

Figure S 81. $^{13}C\{^1H\}$-NMR (101 MHz, $CDCl_3$) spectrum of compound **10-CO**.

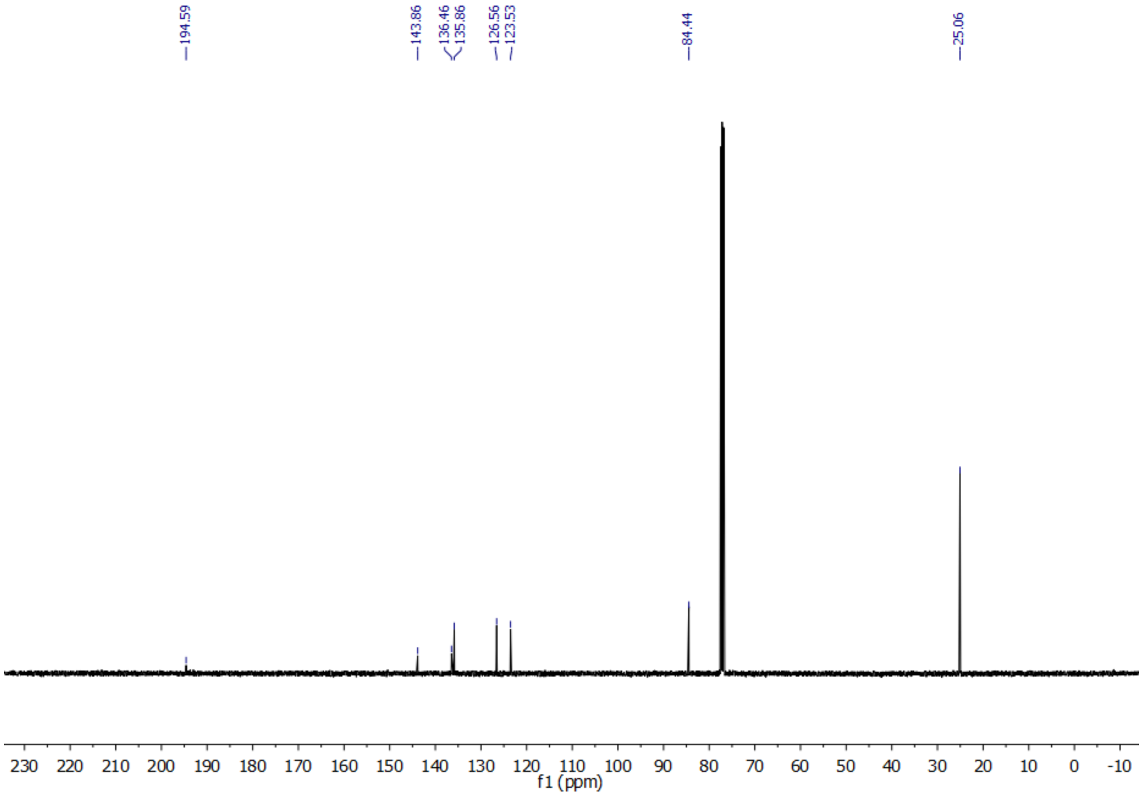

Figure S 82. Full $^{13}C\{^1H\}$-NMR (101 MHz, $CDCl_3$) spectrum of compound **10-CO**.

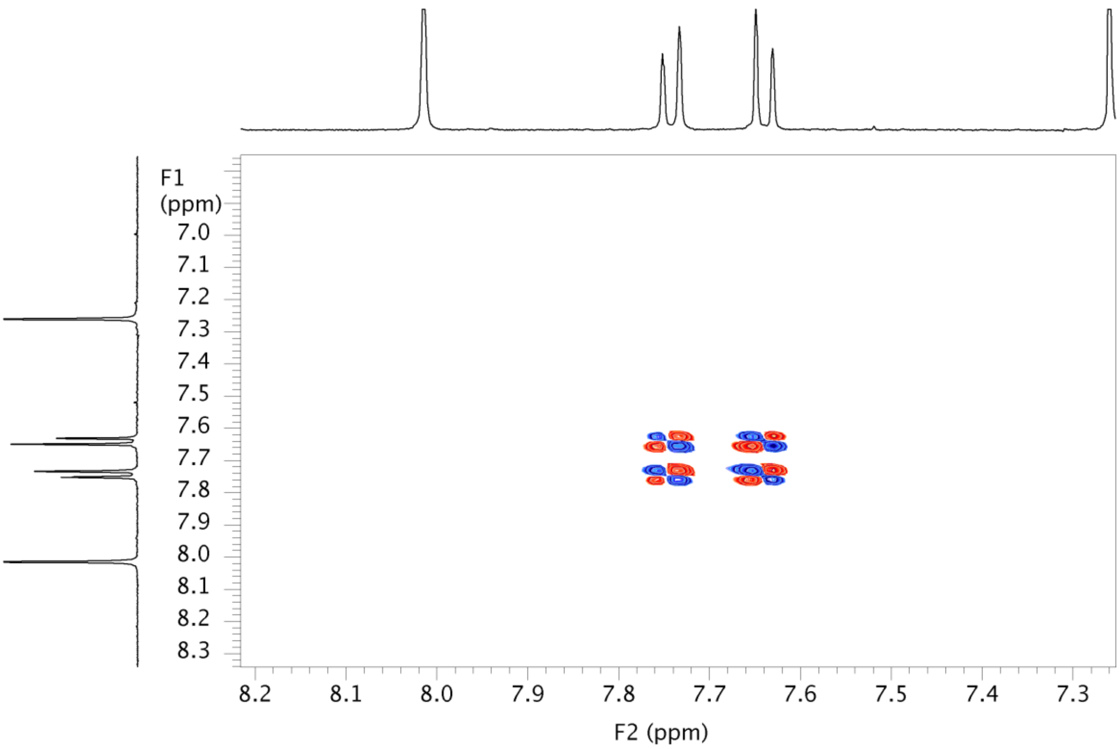

Figure S 83. ^{1}H-^{1}H gDQFCOSY (400 MHz, $CDCl_3$) spectrum of compound **10-CO**.

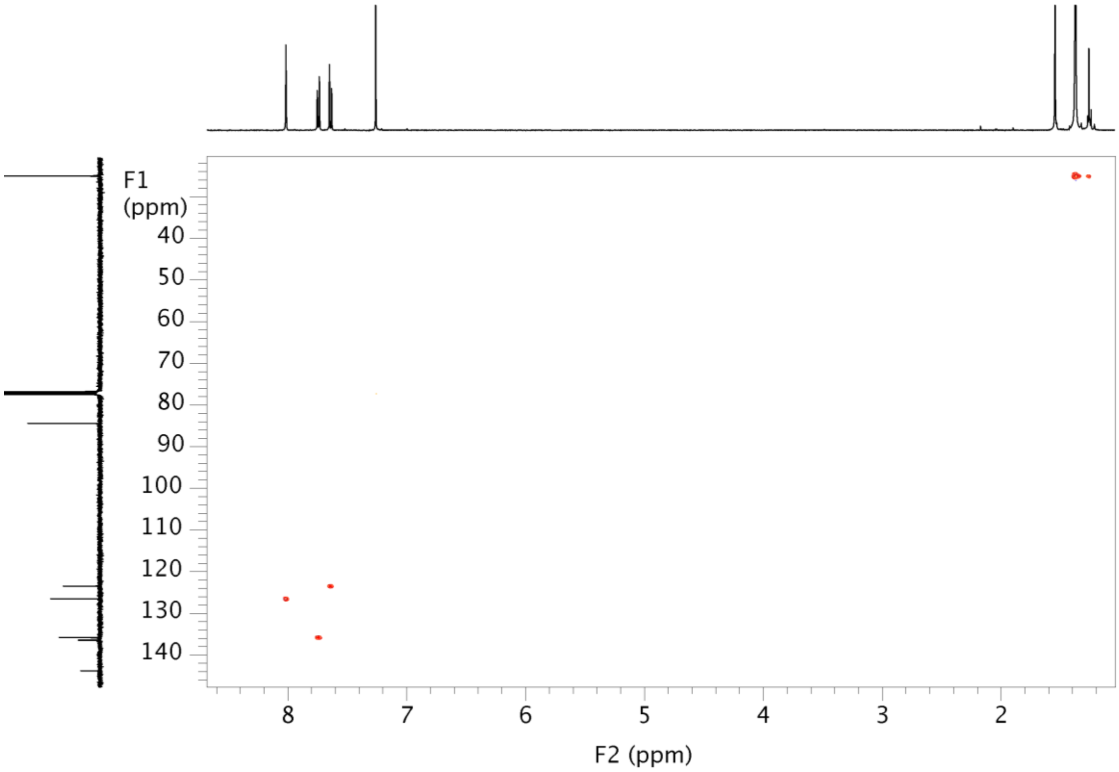

Figure S 84. ^{1}H-^{13}C gc2HSQC (400 MHz, $CDCl_3$) spectrum of compound **10-CO**.

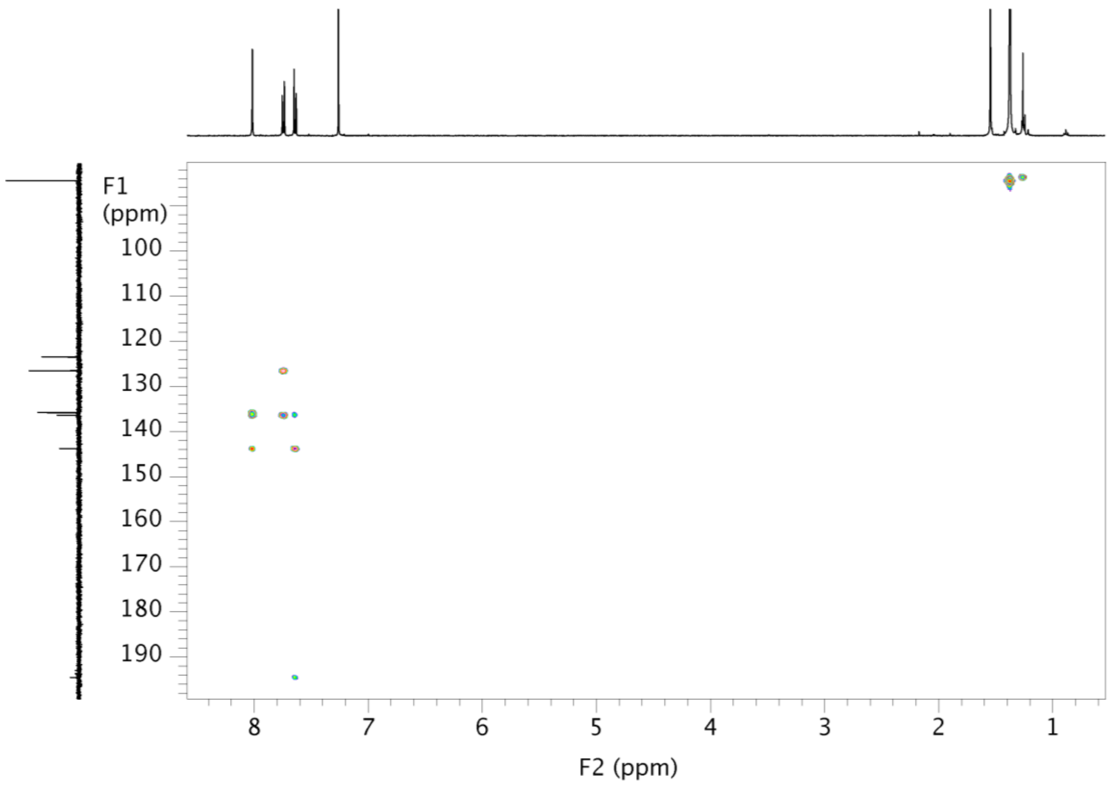

Figure S 85. ^{1}H-^{13}C gc2HMBC (400 MHz, $CDCl_3$) spectrum of compound **10-CO**.

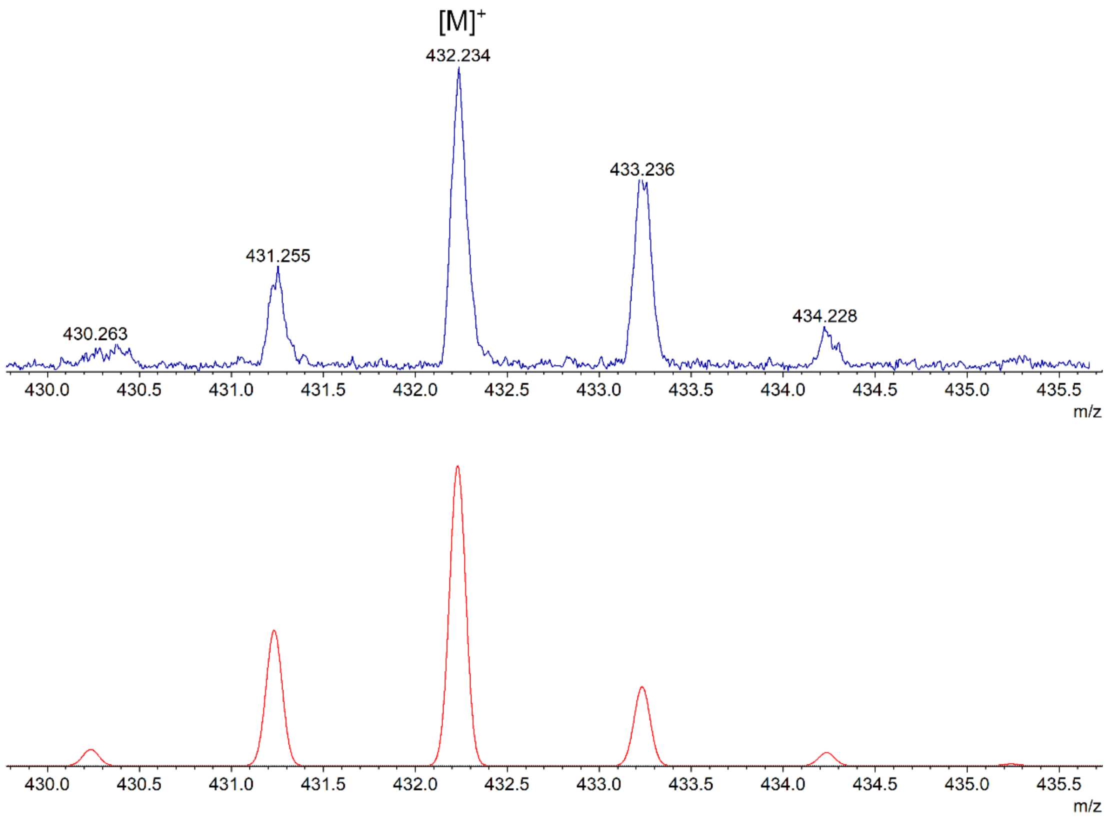

Figure S 86. HRMS (MALDI-TOF) of compound **10-CO**, $[M]^+$. Calculated (red), measured (blue).

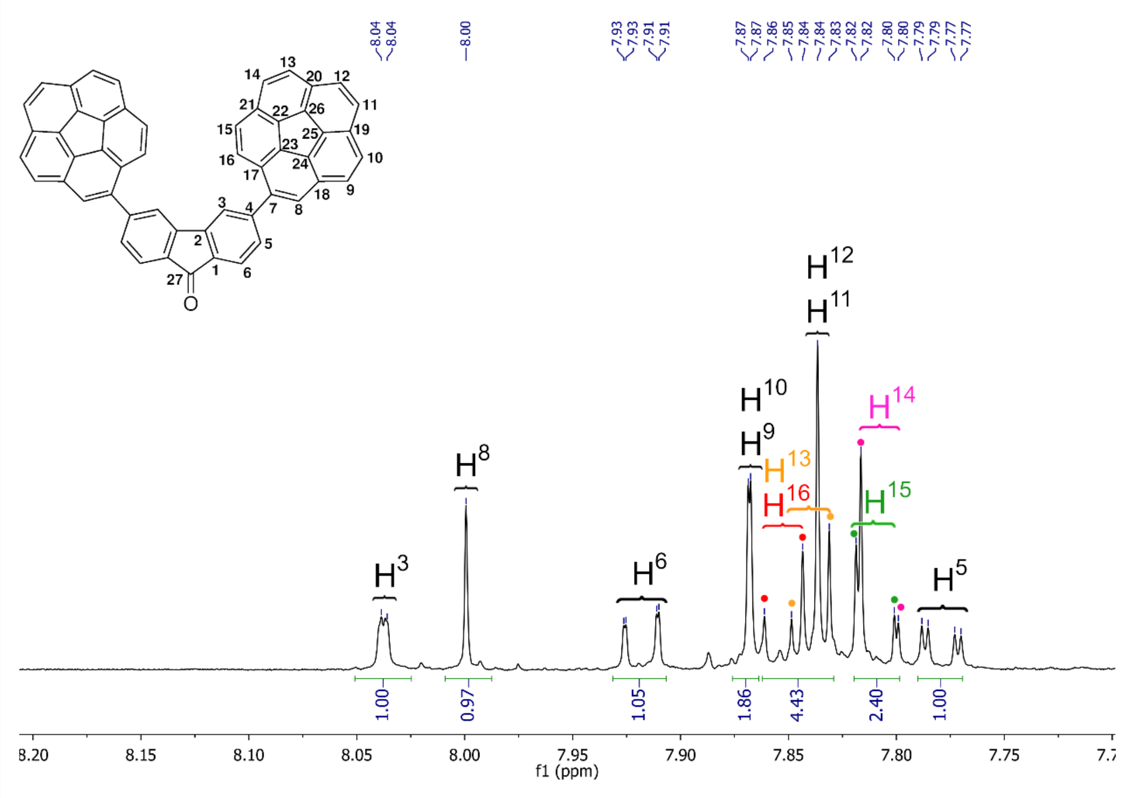

Figure S 87. ^{1}H-NMR (500 MHz, $CDCl_3$) spectrum of compound **11-CO**.

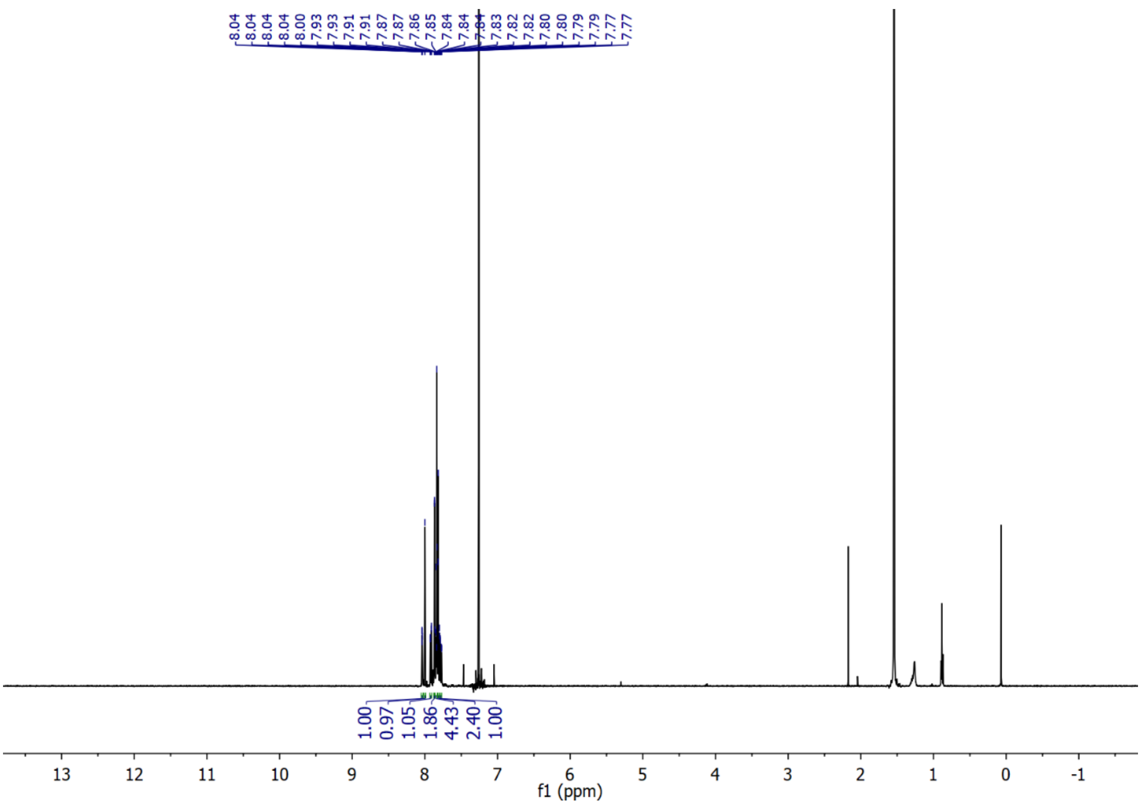

Figure S 88. Full ^{1}H-NMR (500 MHz, $CDCl_3$) spectrum of compound **11-CO**.

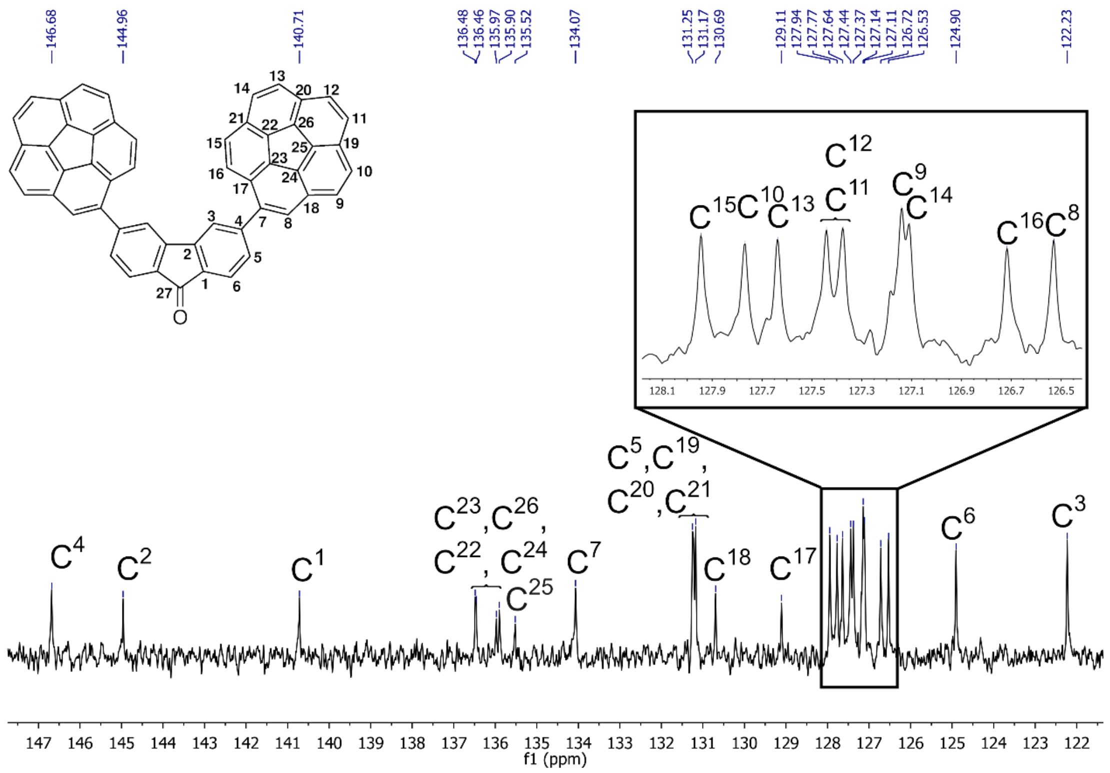

Figure S 89. $^{13}C\{^1H\}$-NMR (101 MHz, $CDCl_3$) spectrum of compound **11-CO**.

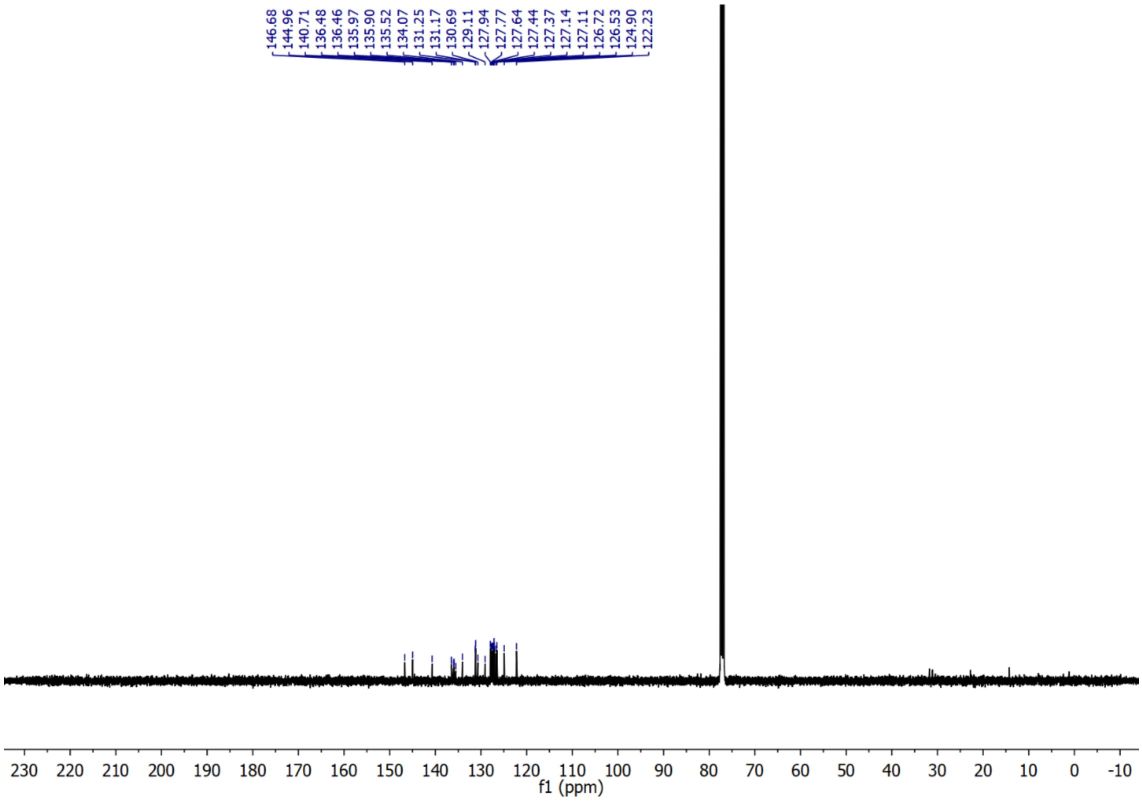

Figure S 90. Full $^{13}C\{^1H\}$-NMR (101 MHz, $CDCl_3$) spectrum of compound **11-CO**.

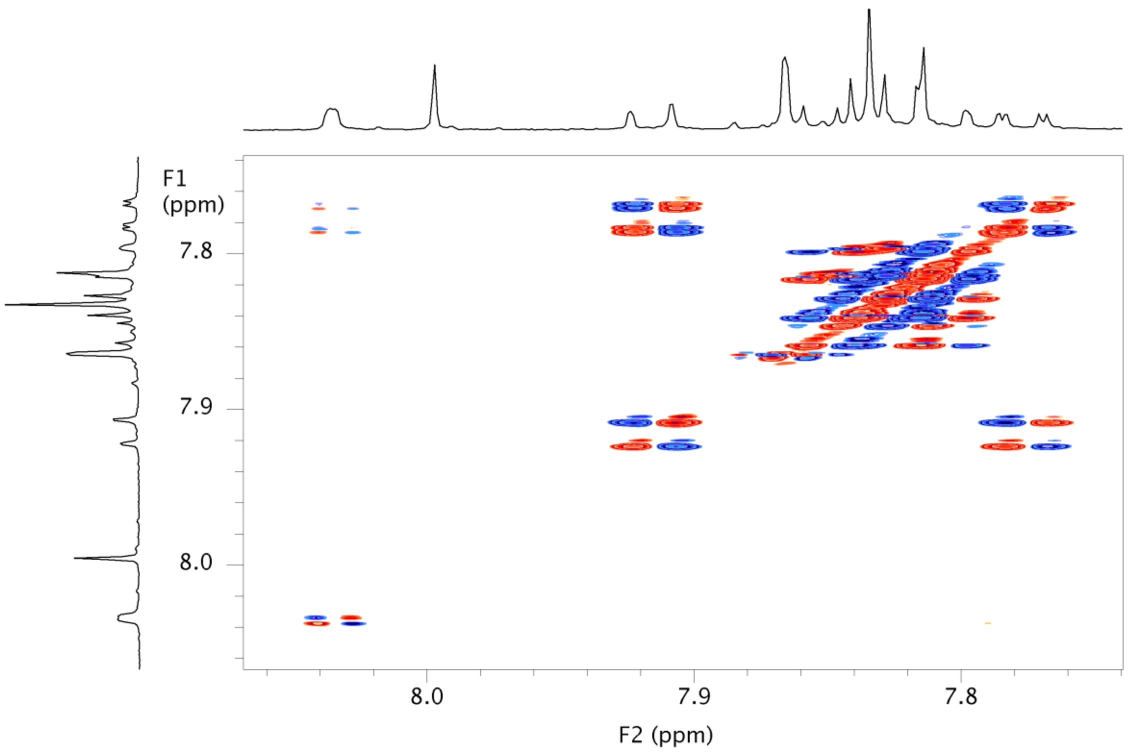

Figure S 91. ^{1}H-^{1}H gDQFCOSY (500 MHz, $CDCl_3$) spectrum of compound **11-CO**.

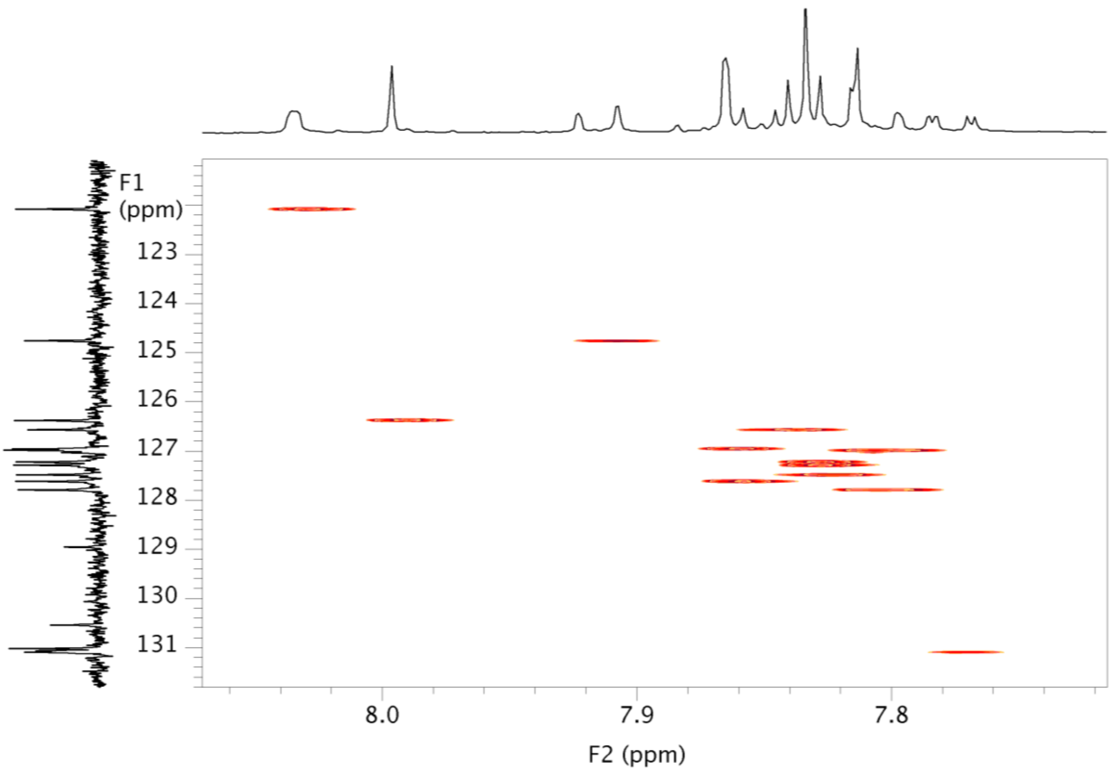

Figure S 92. ^{1}H-^{13}C bsgHSQCAD (500 MHz, $CDCl_3$) spectrum of compound **11-CO**.

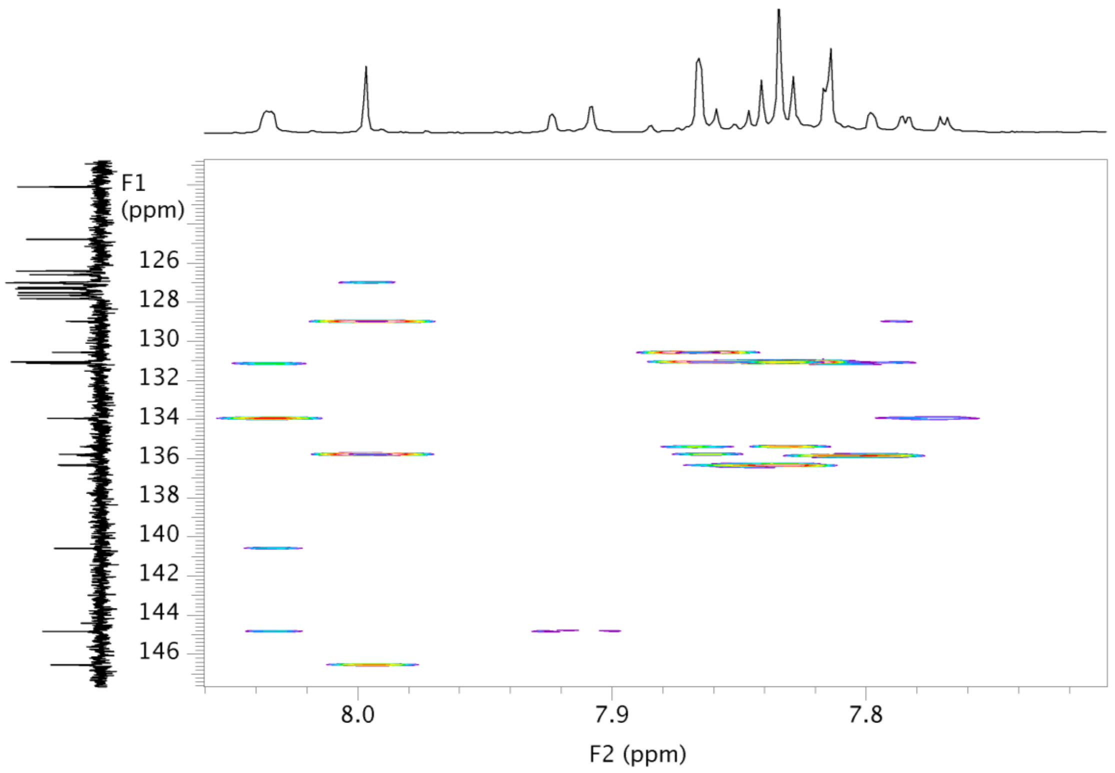

Figure S 93. ^{1}H-^{13}C bsgHMBC (500 MHz, $CDCl_3$) spectrum of compound **11-CO**.

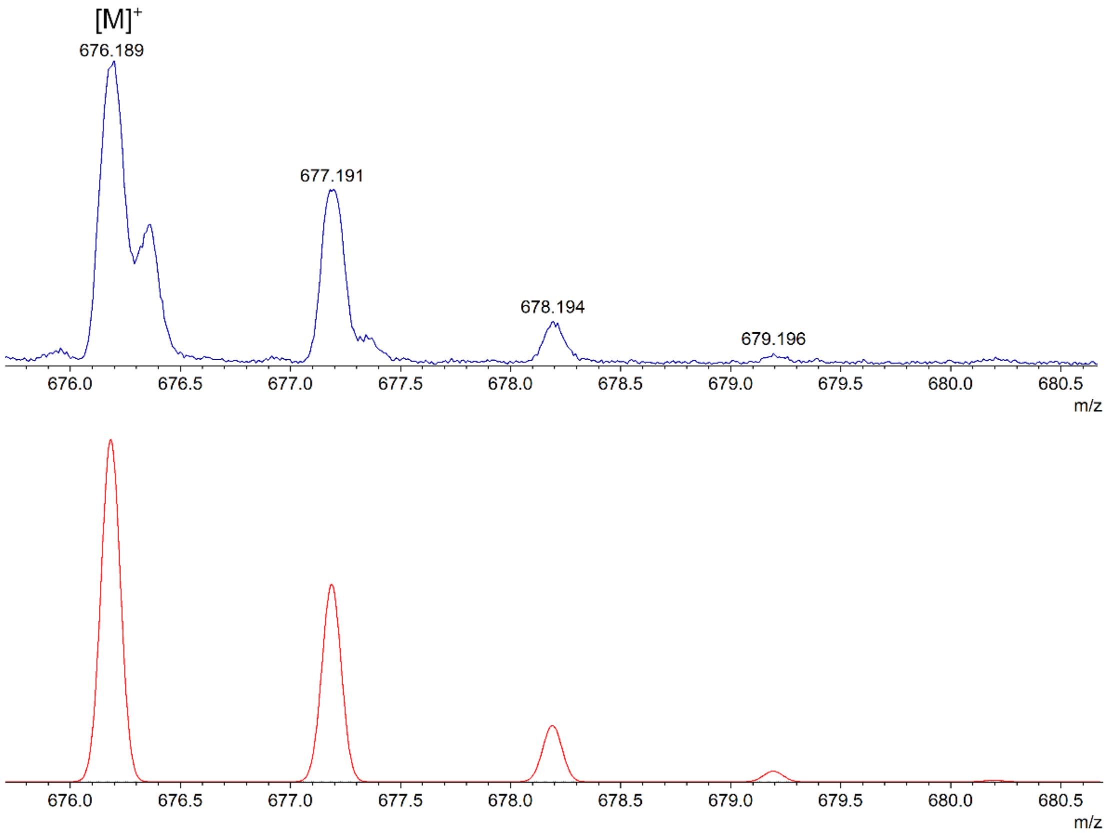

Figure S 94. HRMS (MALDI-TOF) of compound **11-CO**, $[M]^+$. Calculated (red), measured (blue).

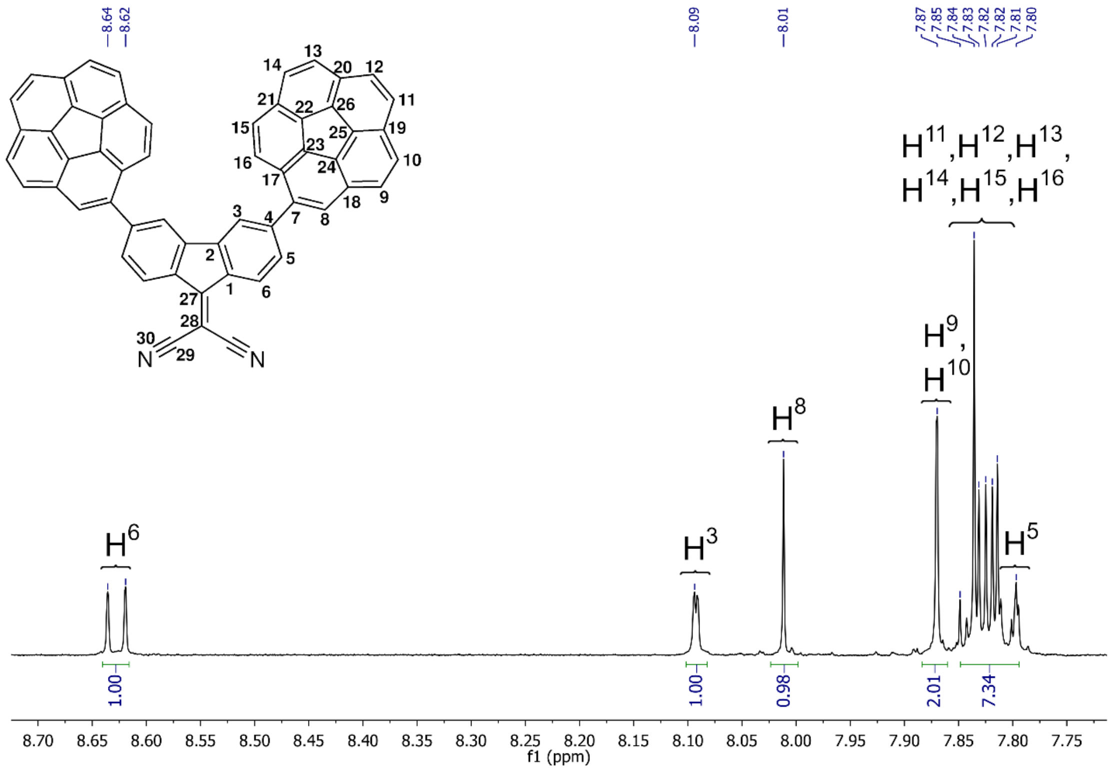

Figure S 95. ^{1}H-NMR (500 MHz, $CDCl_3$) spectrum of compound **11-C(CN)2**.

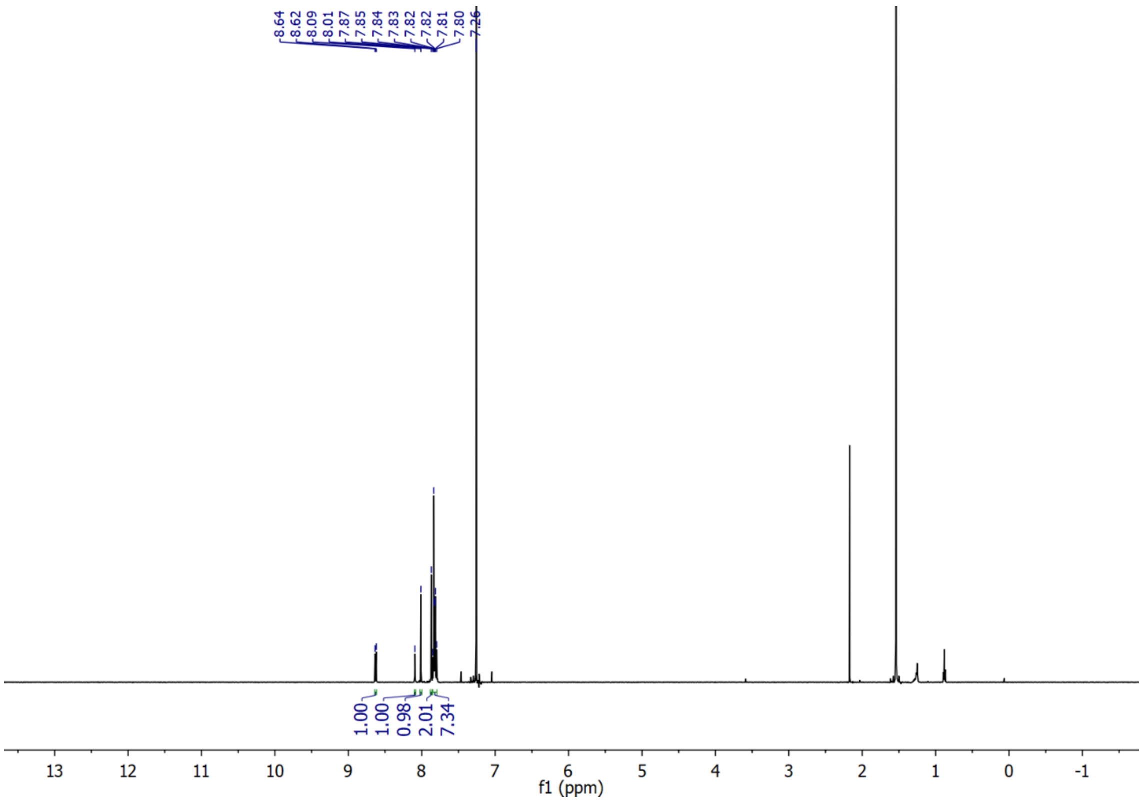

Figure S 96. Full ^{1}H-NMR (500 MHz, $CDCl_3$) spectrum of compound **11-C(CN)2**.

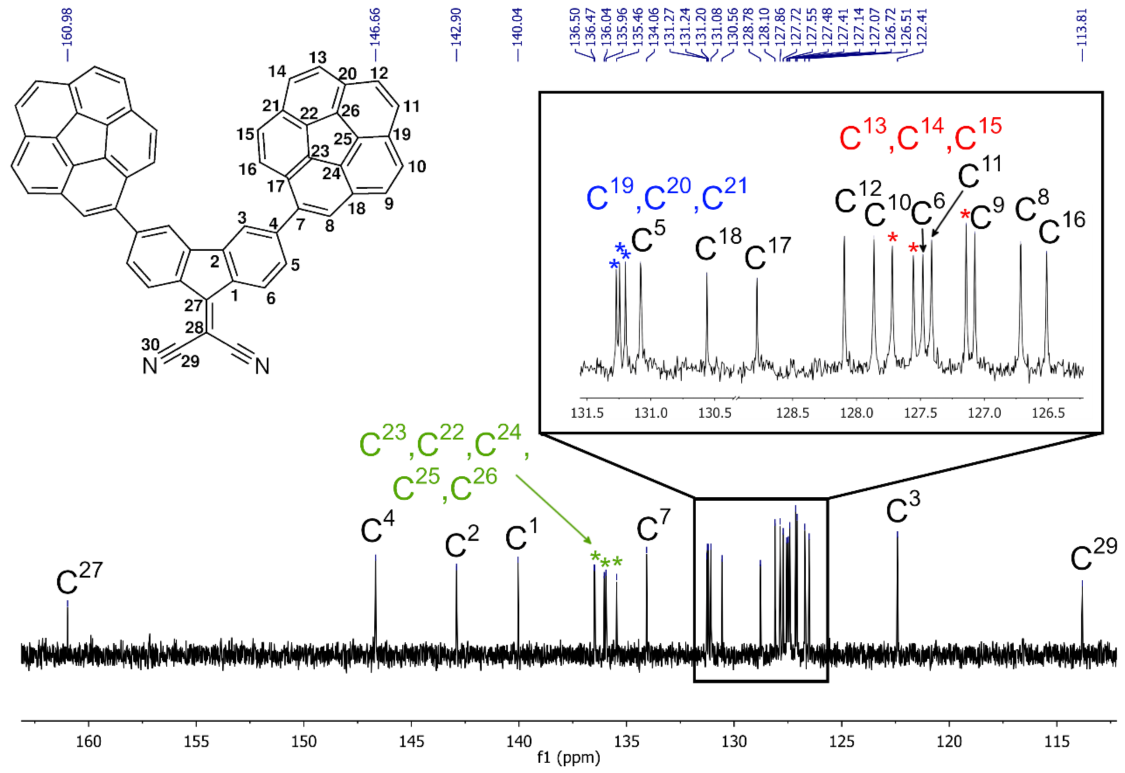

Figure S 97. $^{13}C\{^1H\}$-NMR (126 MHz, $CDCl_3$) spectrum of compound **11-C(CN)₂**.

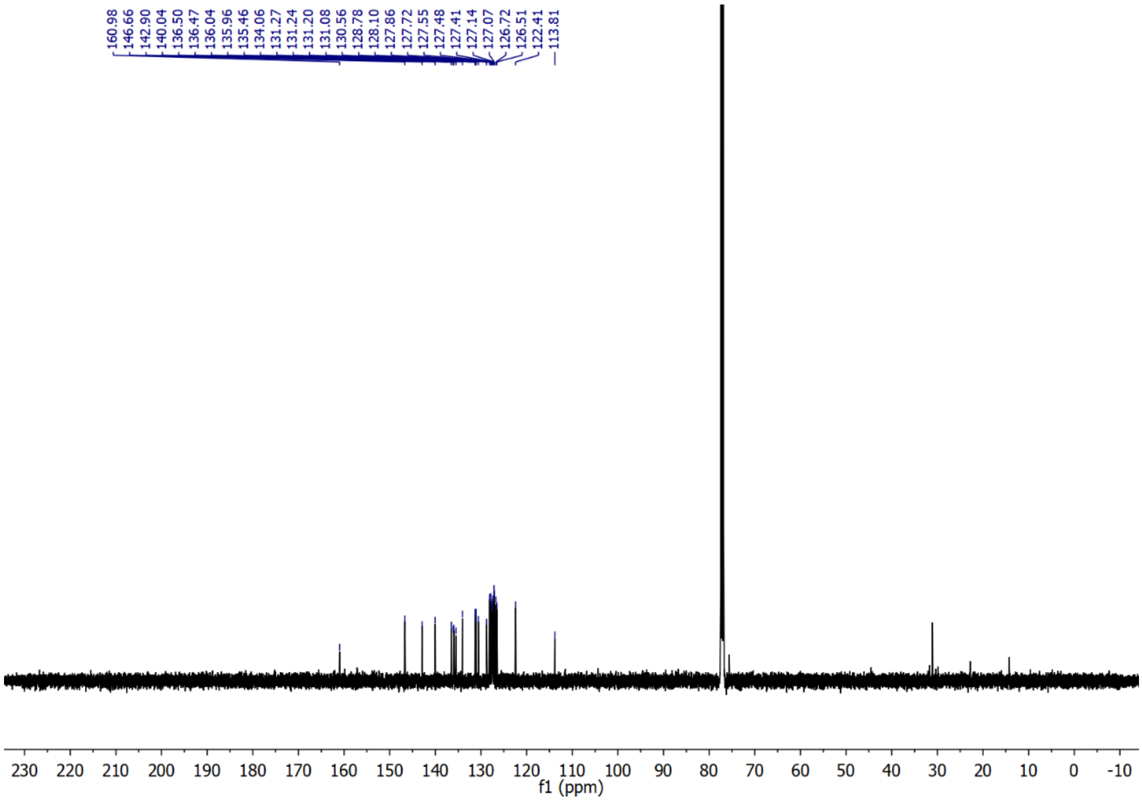

Figure S 98. Full $^{13}C\{^1H\}$-NMR (126 MHz, $CDCl_3$) spectrum of compound **11-C(CN)₂**.

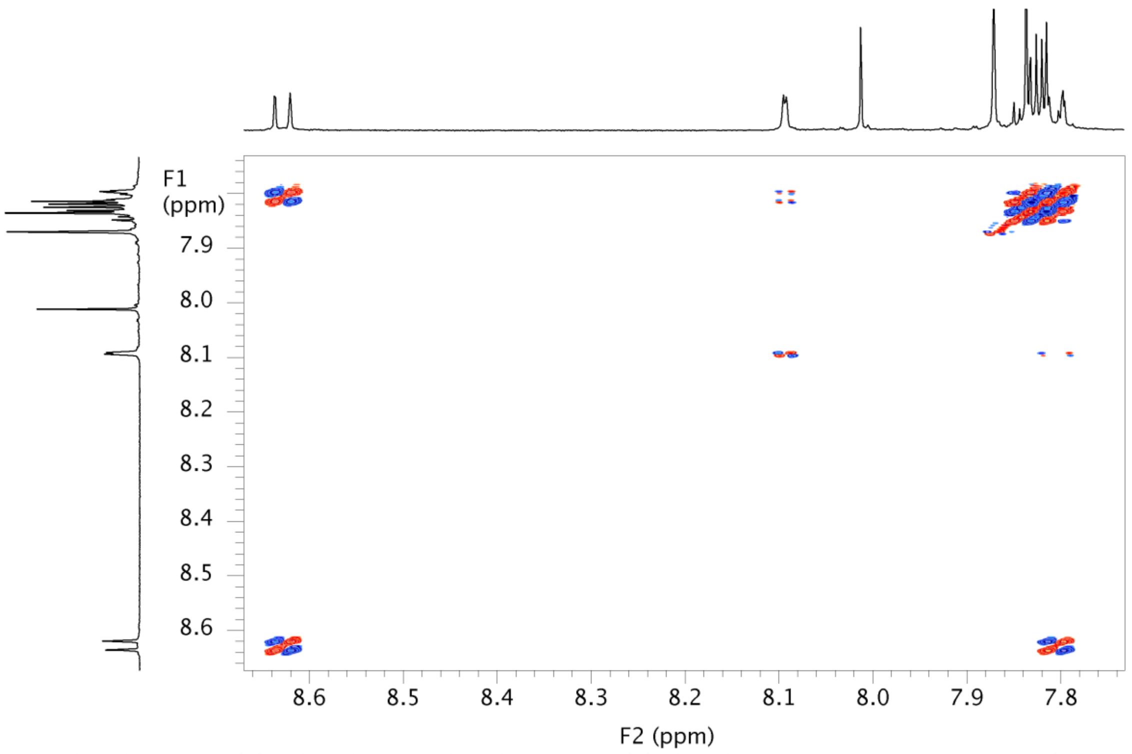

Figure S 99. ^{1}H-^{1}H gDQFCOSY (500 MHz, $CDCl_3$) spectrum of compound **11-C(CN)$_2$**.

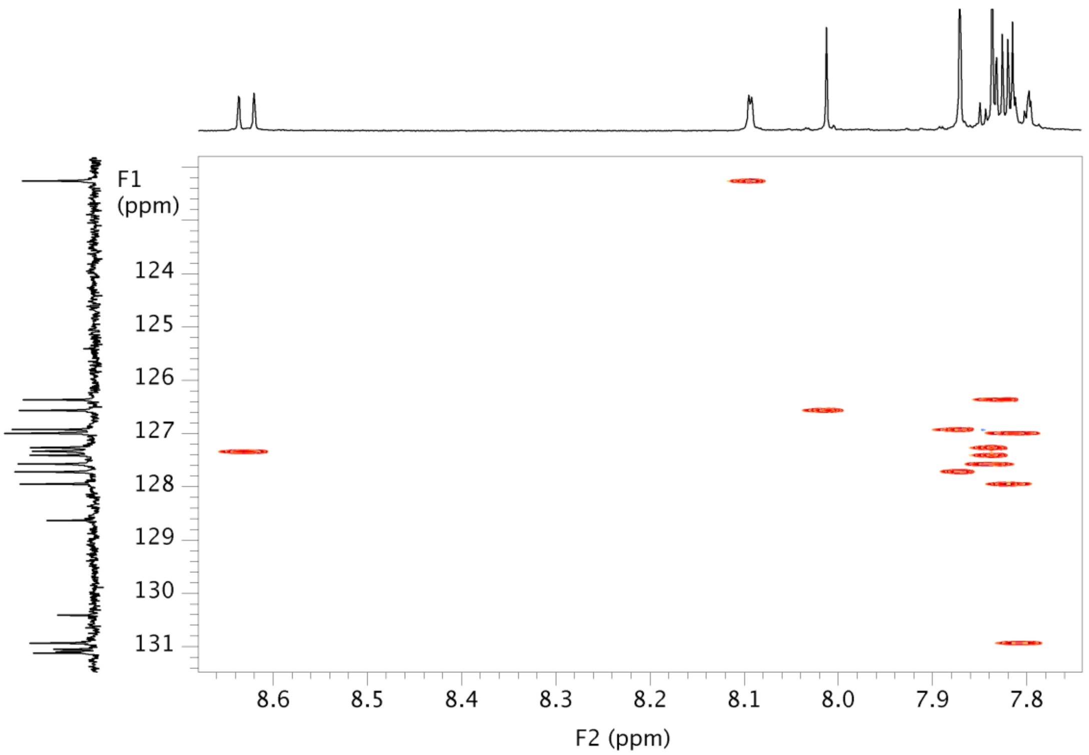

Figure S 100. ^{1}H-^{13}C bsgHSQCAD (500 MHz, $CDCl_3$) spectrum of compound **11-C(CN)$_2$**.

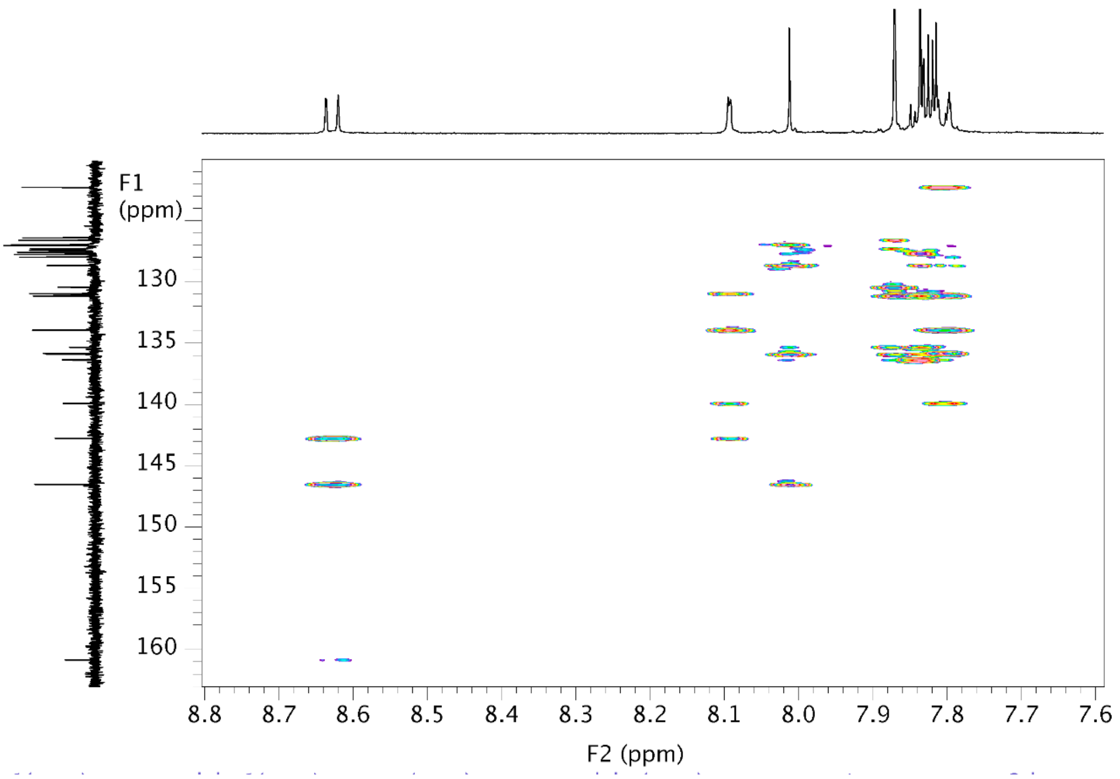

Figure S 101. ^{1}H-^{13}C bsgHMBC (500 MHz, $CDCl_3$) spectrum of compound **11-C(CN)₂**.

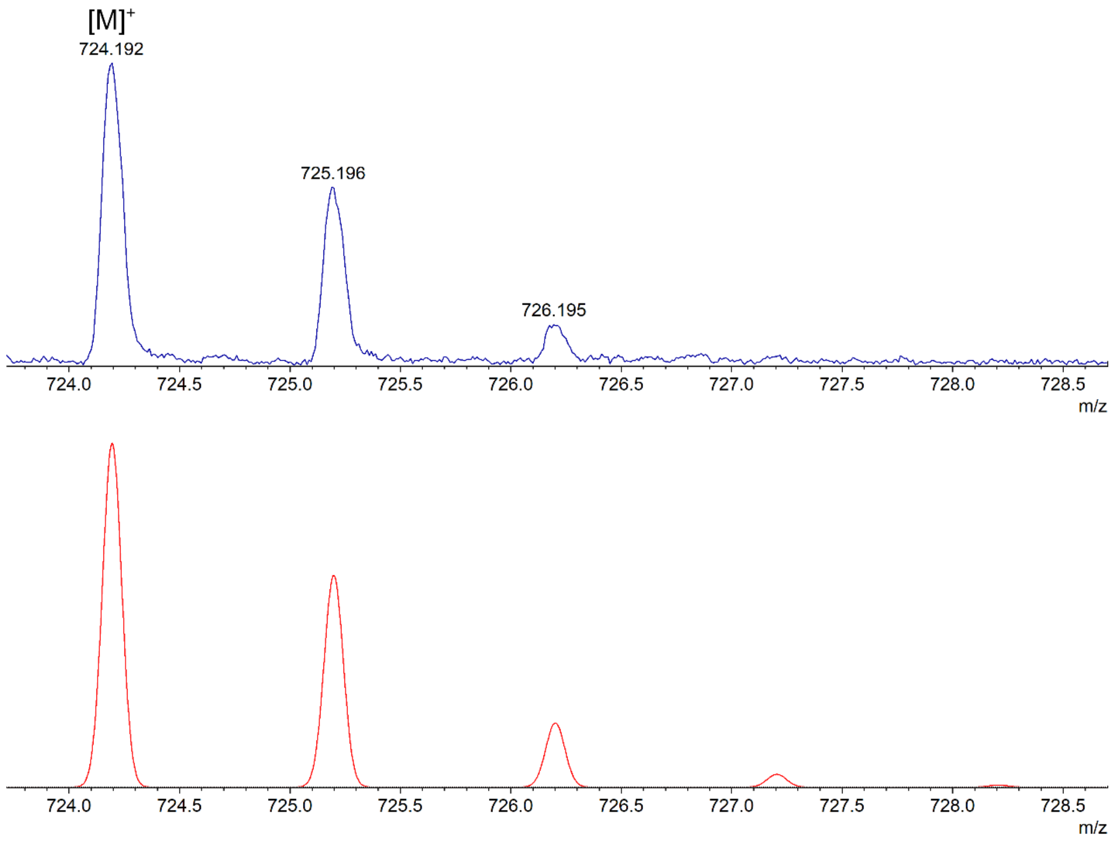

Figure S 102. HRMS (MALDI-TOF) of compound **11-C(CN)$_2$**, $[M]^+$. Calculated (red), measured (blue).

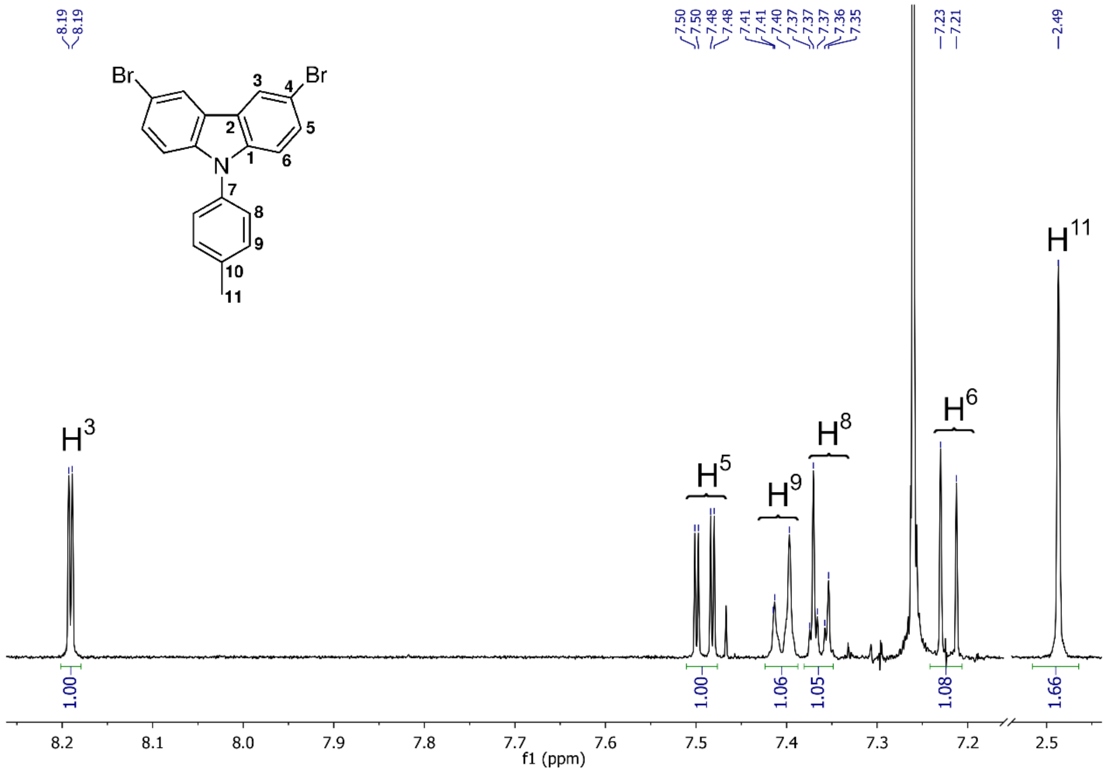

Figure S 103. ^{1}H-NMR (500 MHz, $CDCl_3$) spectrum of compound **14**.

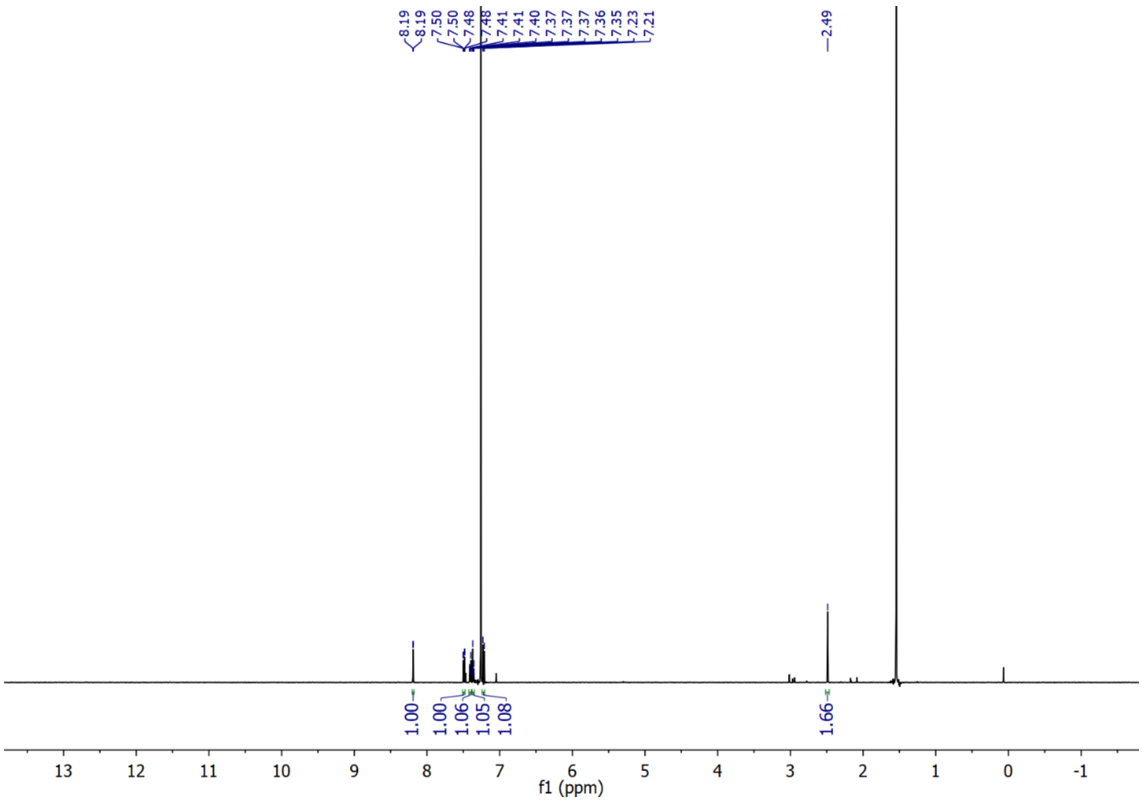

Figure S 104. Full ^{1}H-NMR (500 MHz, $CDCl_3$) spectrum of compound **14**.

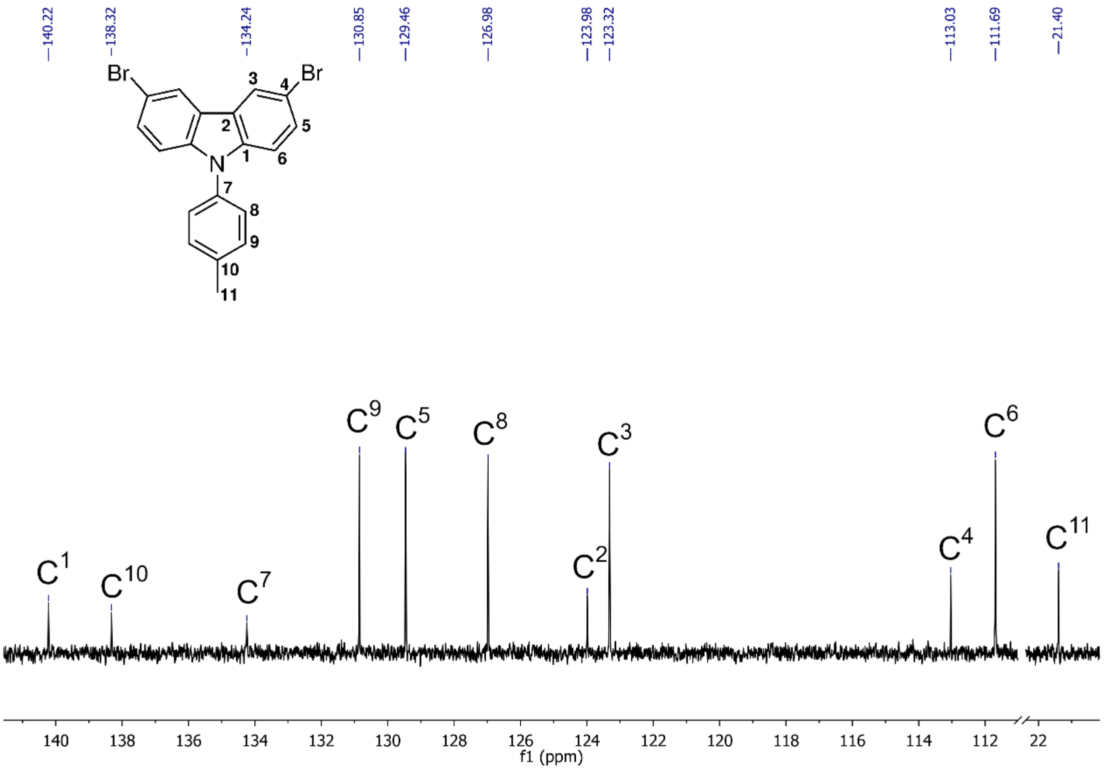

Figure S 105. $^{13}C\{^{1}H\}$-NMR (101 MHz, $CDCl_3$) spectrum of compound **14**.

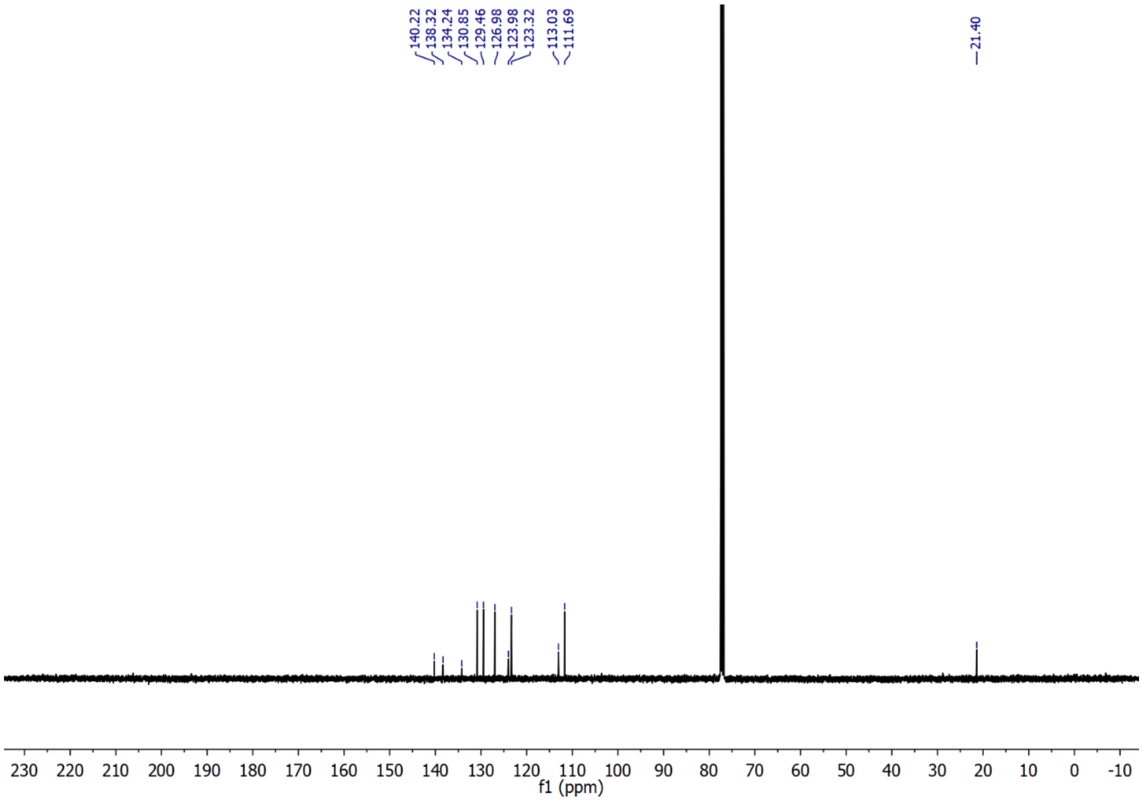

Figure S 106. Full $^{13}C\{^{1}H\}$-NMR (101 MHz, $CDCl_3$) spectrum of compound **14**.

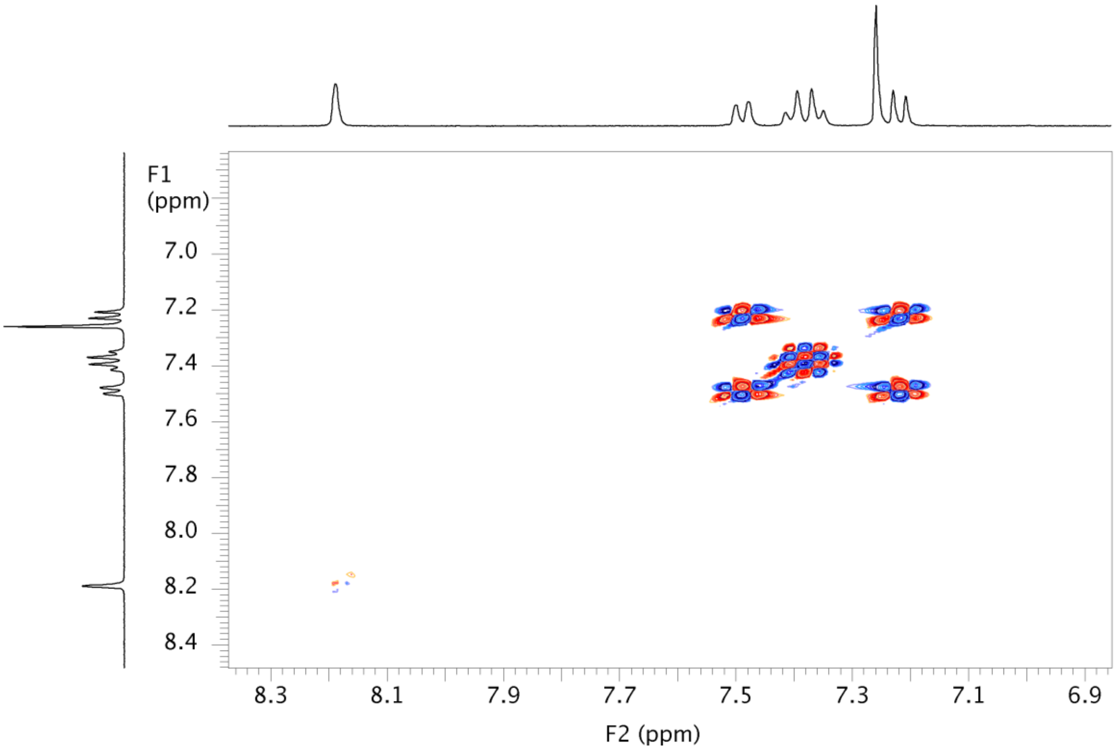

Figure S 107. ^{1}H-^{1}H gDQFCOSY (400 MHz, $CDCl_3$) spectrum of compound **14**.

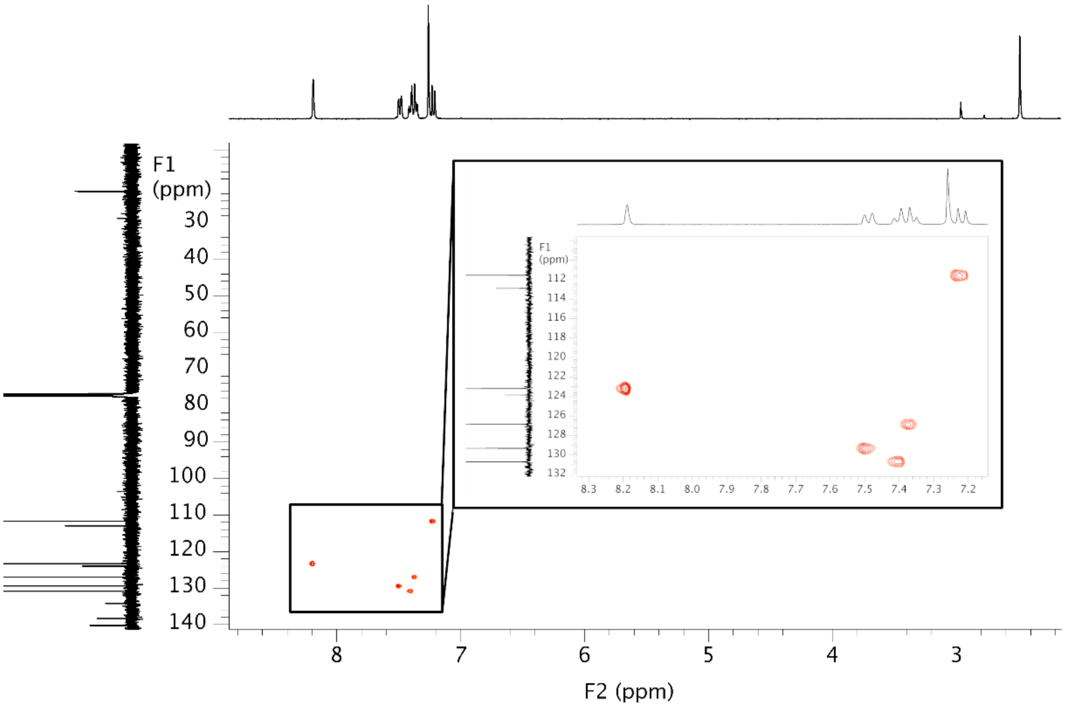

Figure S 108. ^{1}H-^{13}C gc2HSQC (400 MHz, $CDCl_3$) spectrum of compound **14**.

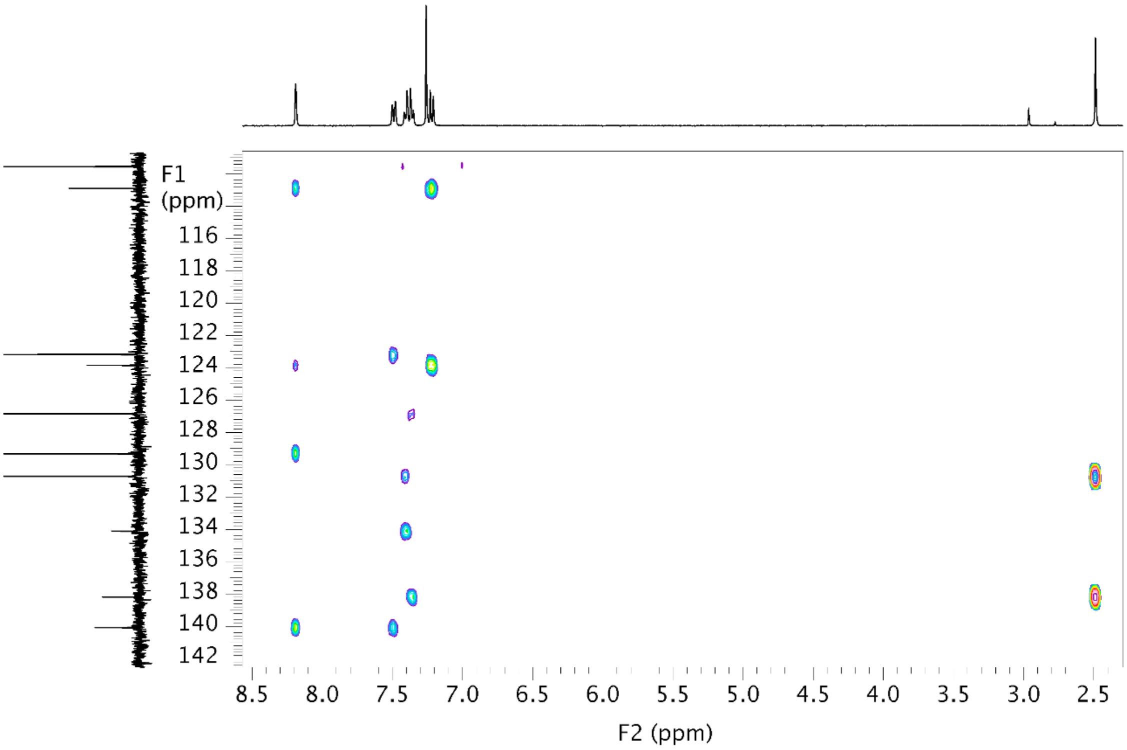

Figure S 109. ^{1}H-^{13}C gc2HSMBC (400 MHz, $CDCl_3$) spectrum of compound **14**.

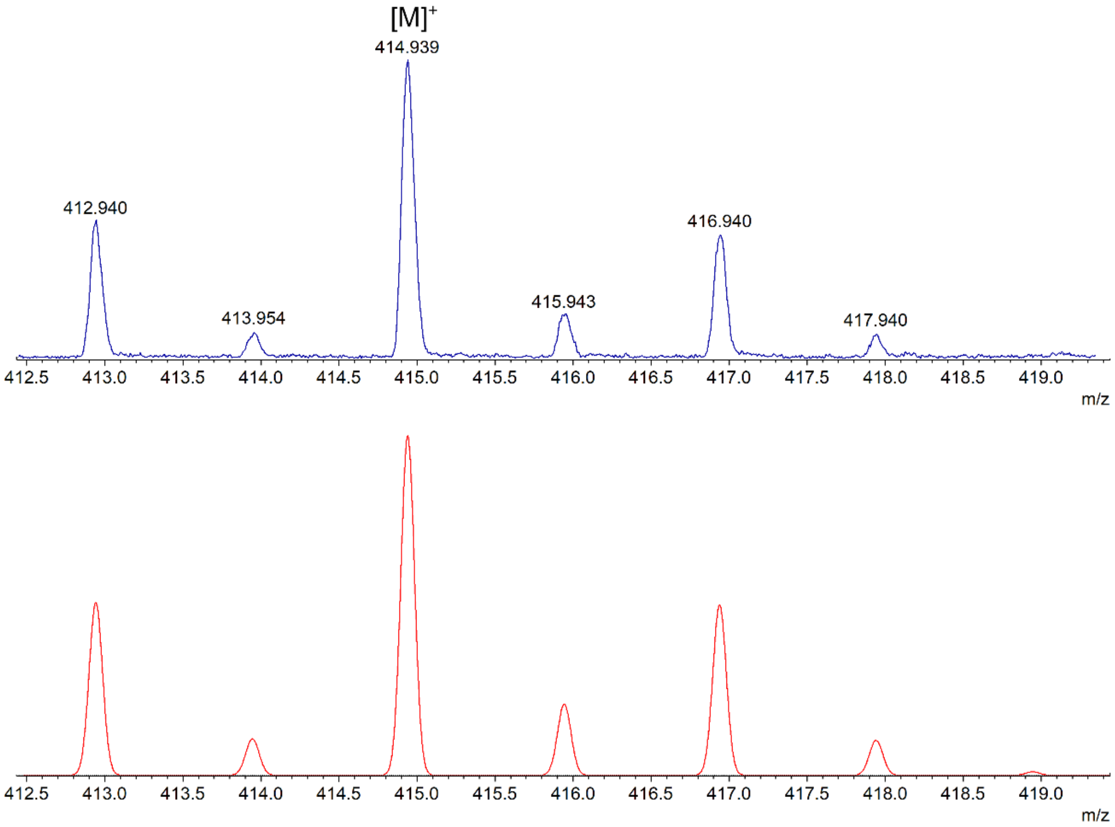

Figure S 110. HRMS (MALDI-TOF) of compound **14**, $[M]^+$. Calculated (red), measured (blue).

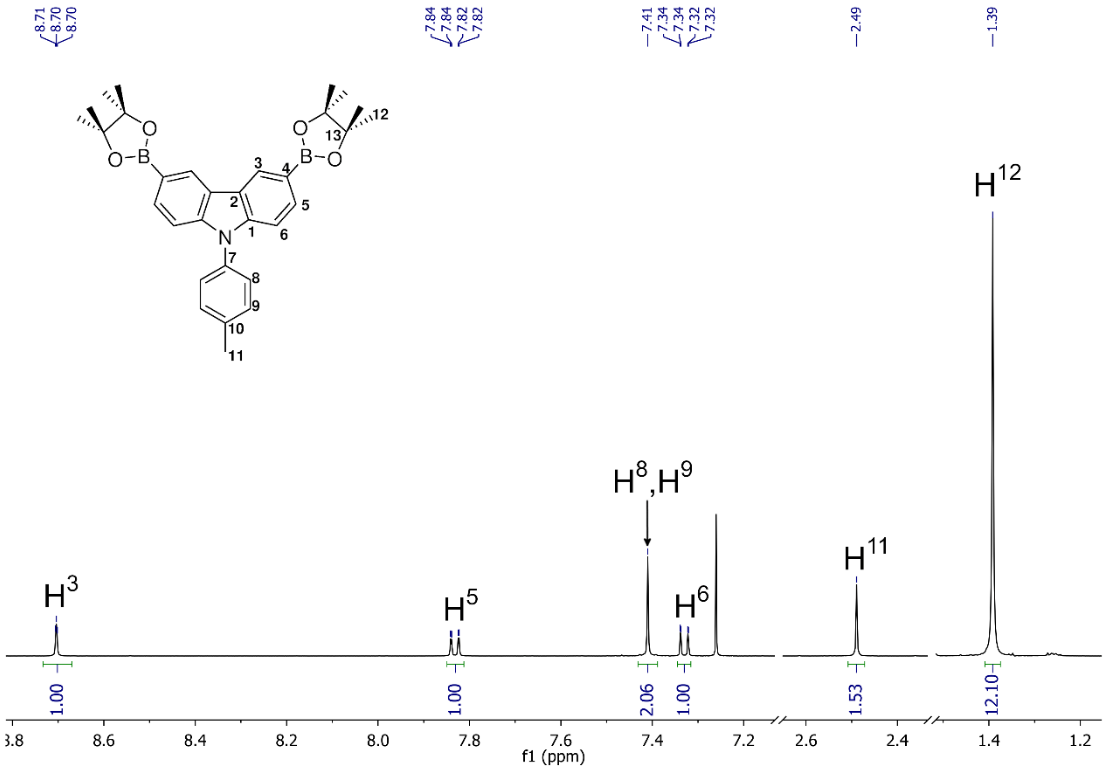

Figure S 111. ^{1}H-NMR (500 MHz, $CDCl_3$) spectrum of compound **15**.

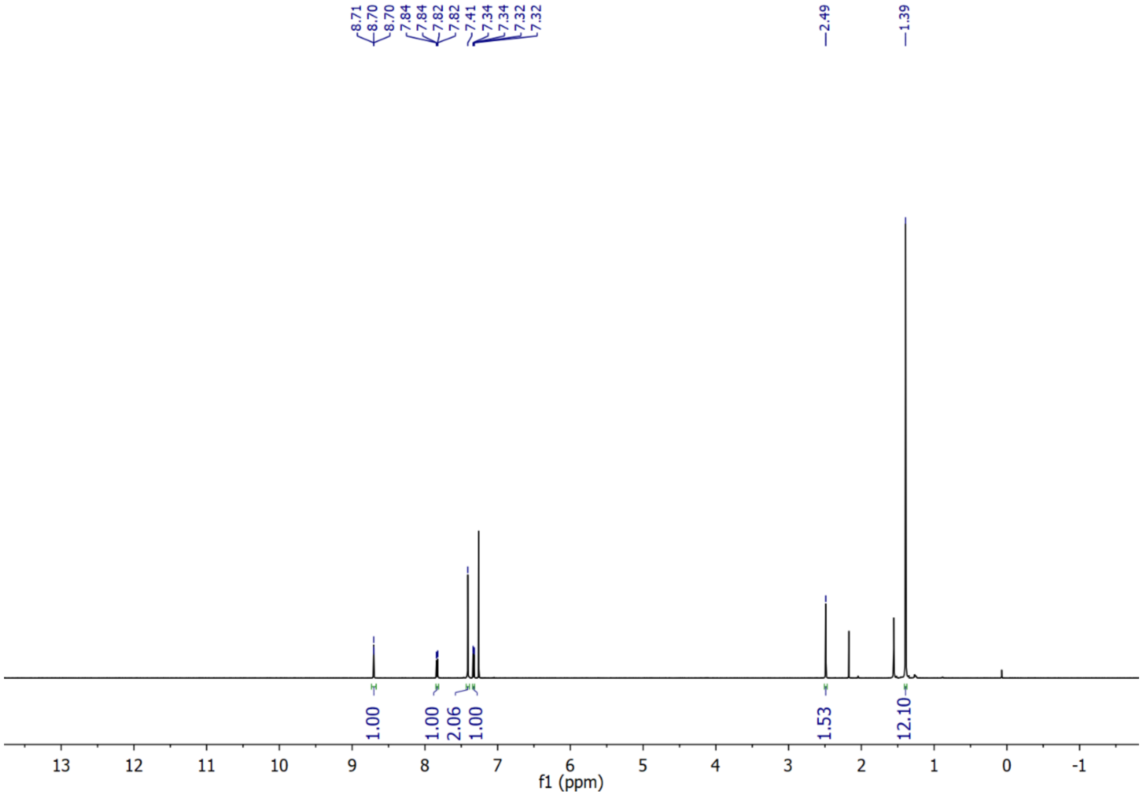

Figure S 112. Full ^{1}H-NMR (500 MHz, $CDCl_3$) spectrum of compound **15**.

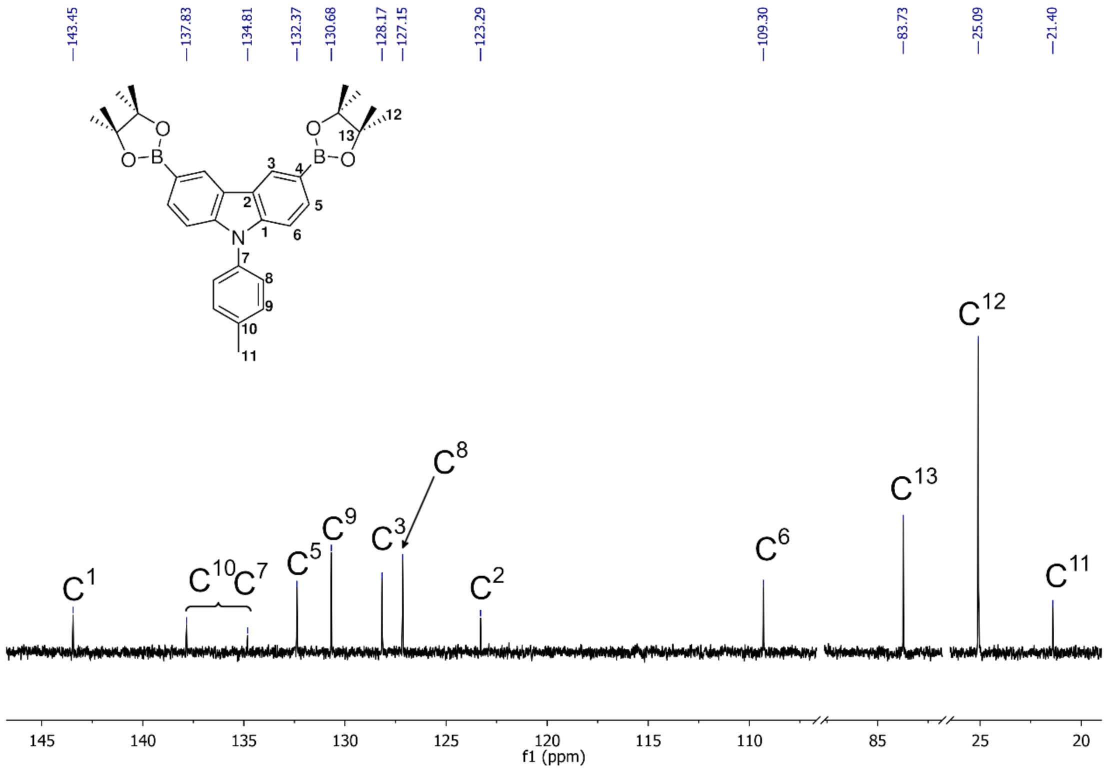

Figure S 113. $^{13}C\{^{1}H\}$-NMR (101 MHz, $CDCl_3$) spectrum of compound **15**.

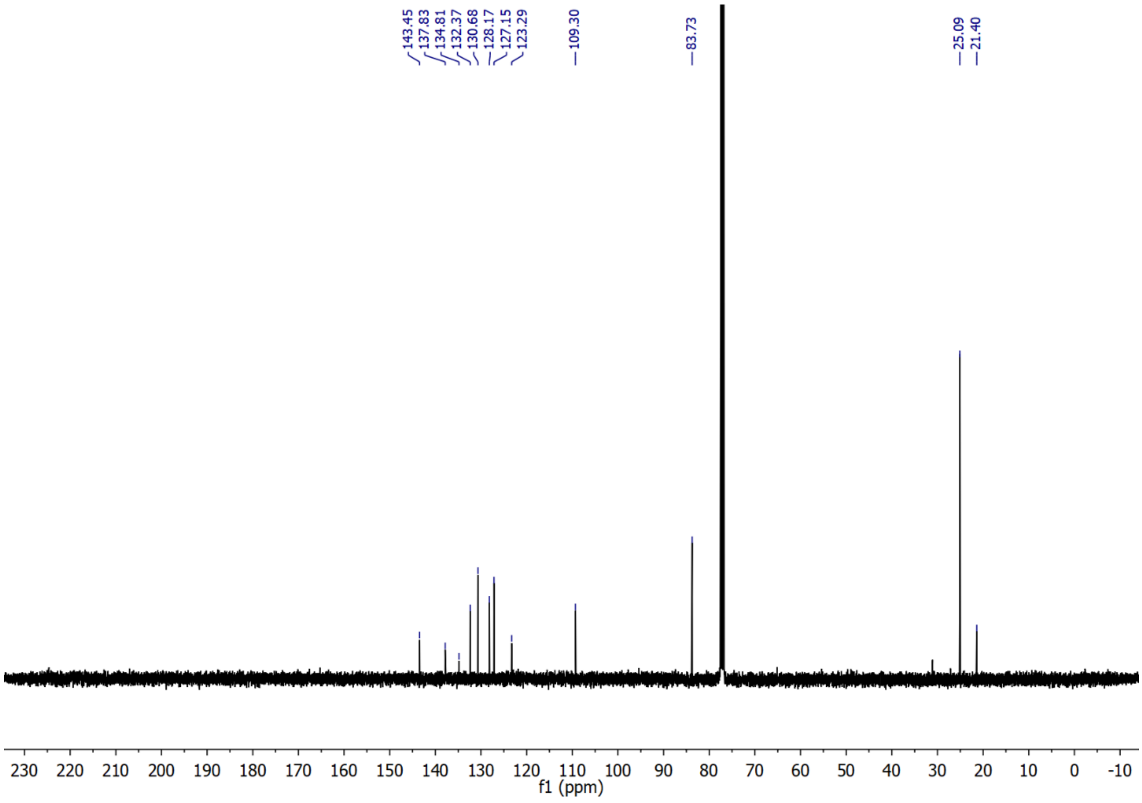

Figure S 114. Full $^{13}C\{^{1}H\}$-NMR (101 MHz, $CDCl_3$) spectrum of compound **15**.

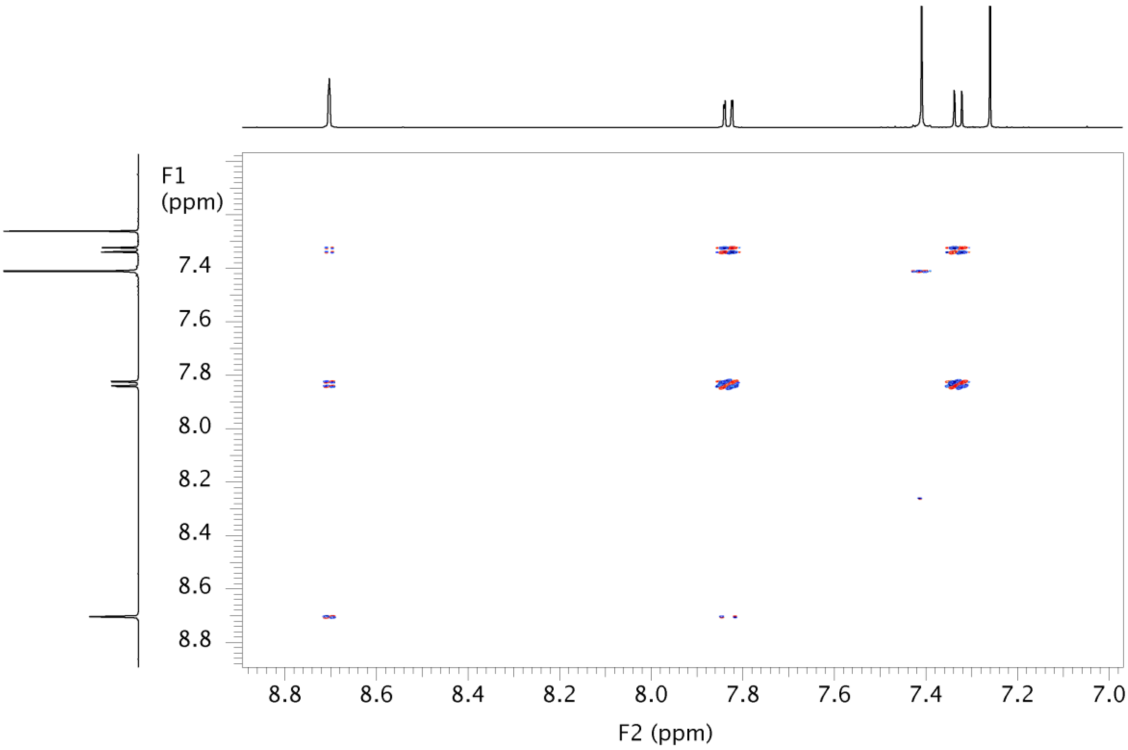

Figure S 115. ^{1}H-^{1}H gDQFCOSY (500 MHz, $CDCl_3$) spectrum of compound **15**.

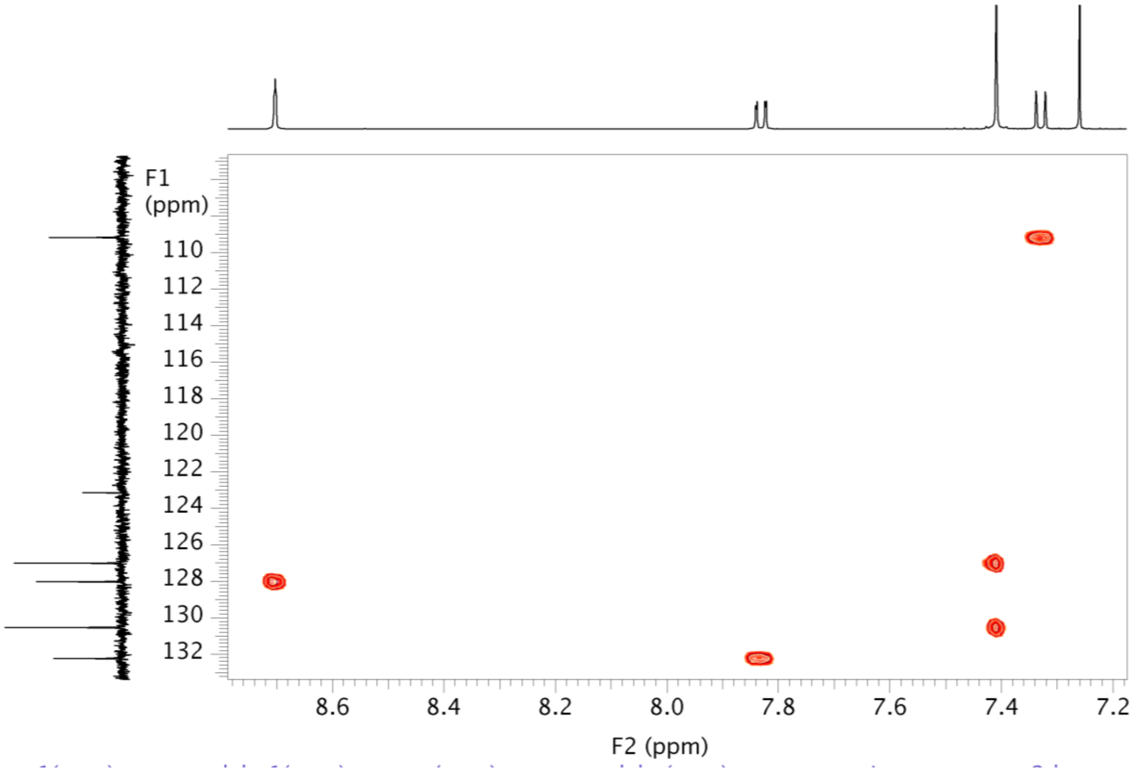

Figure S 116. ^{1}H-^{13}C gc2HSQC (400 MHz, $CDCl_3$) spectrum of compound **15**.

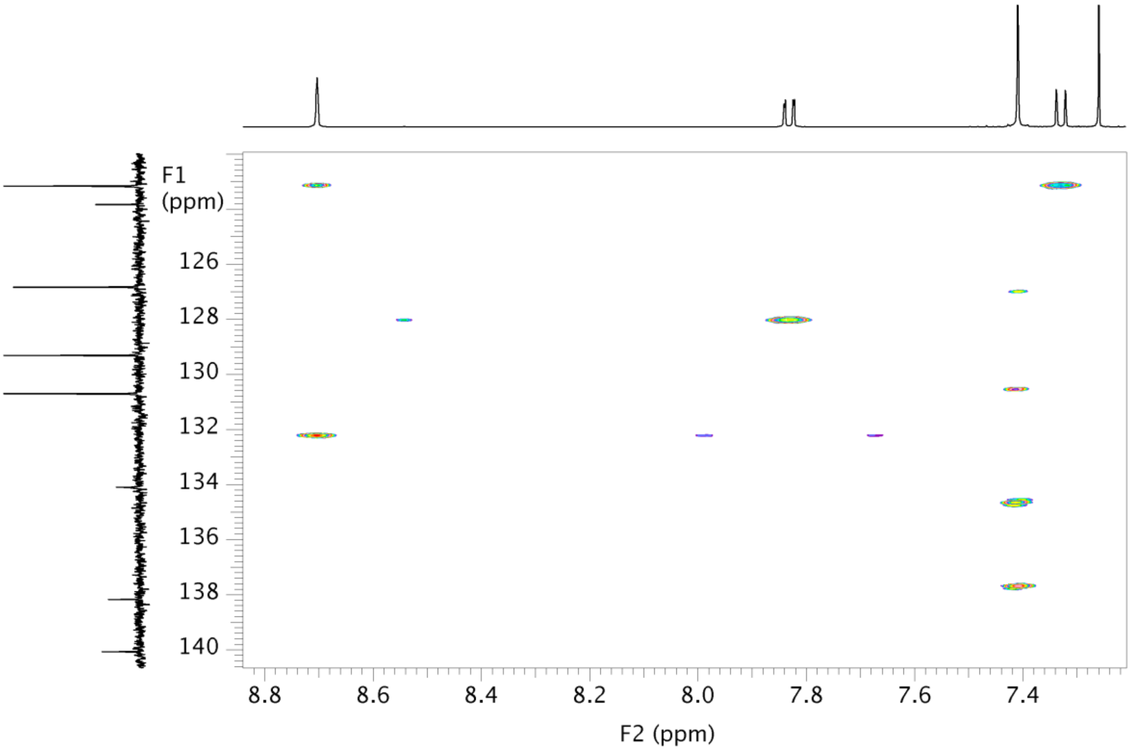

Figure S 117. ^{1}H-^{13}C bsgHSMBC (500 MHz, $CDCl_3$) spectrum of compound **15**.

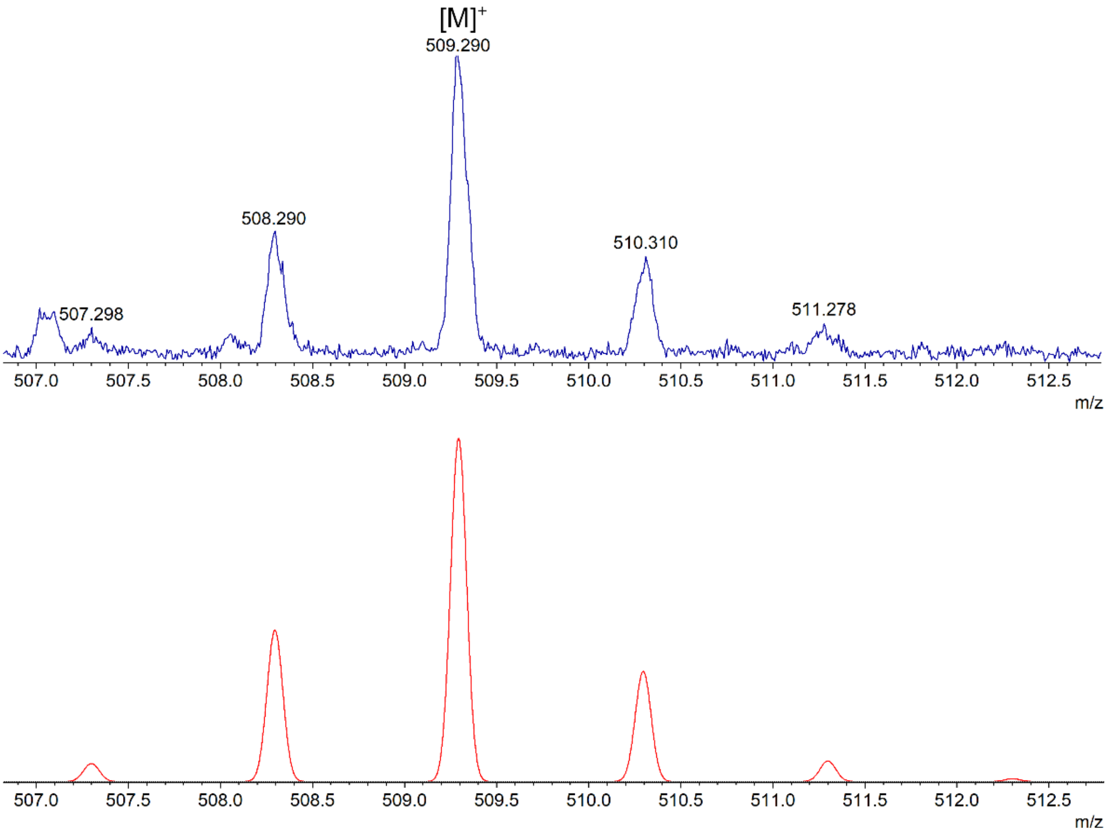

Figure S 118. HRMS (MALDI-TOF) of compound **15**, $[M]^{+}$. Calculated (red), measured (blue).

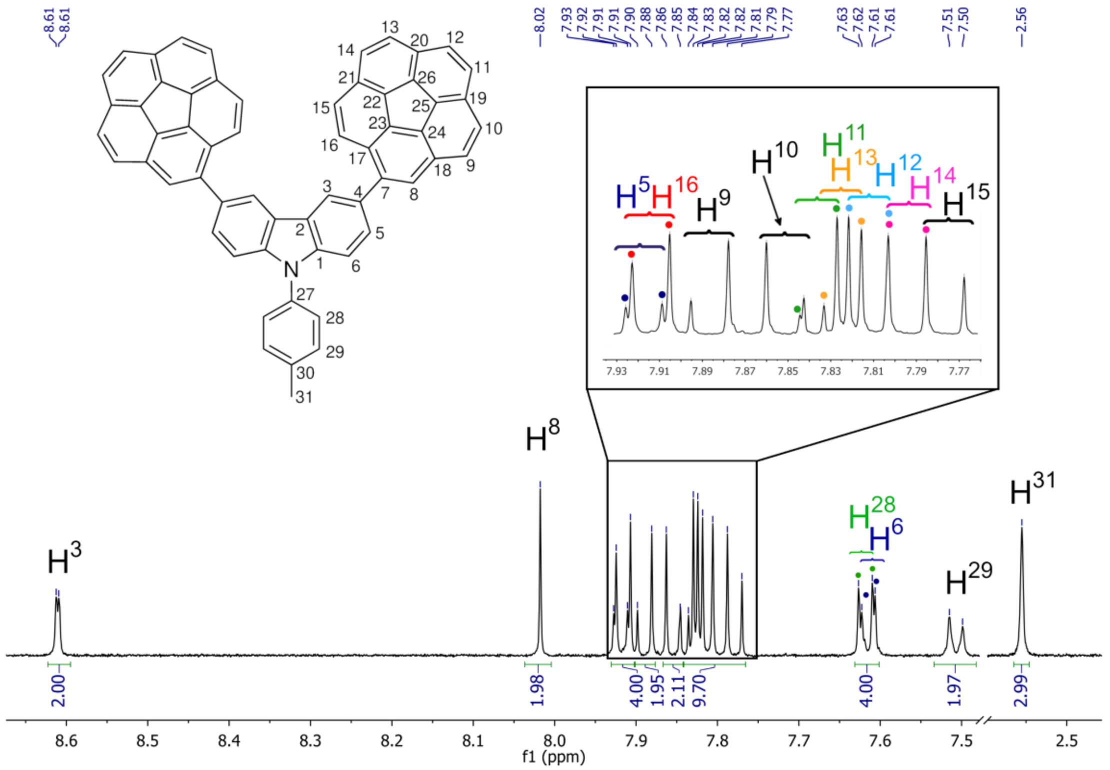

Figure S 119. ^{1}H-NMR (500 MHz, $CDCl_3$) spectrum of compound **16**.

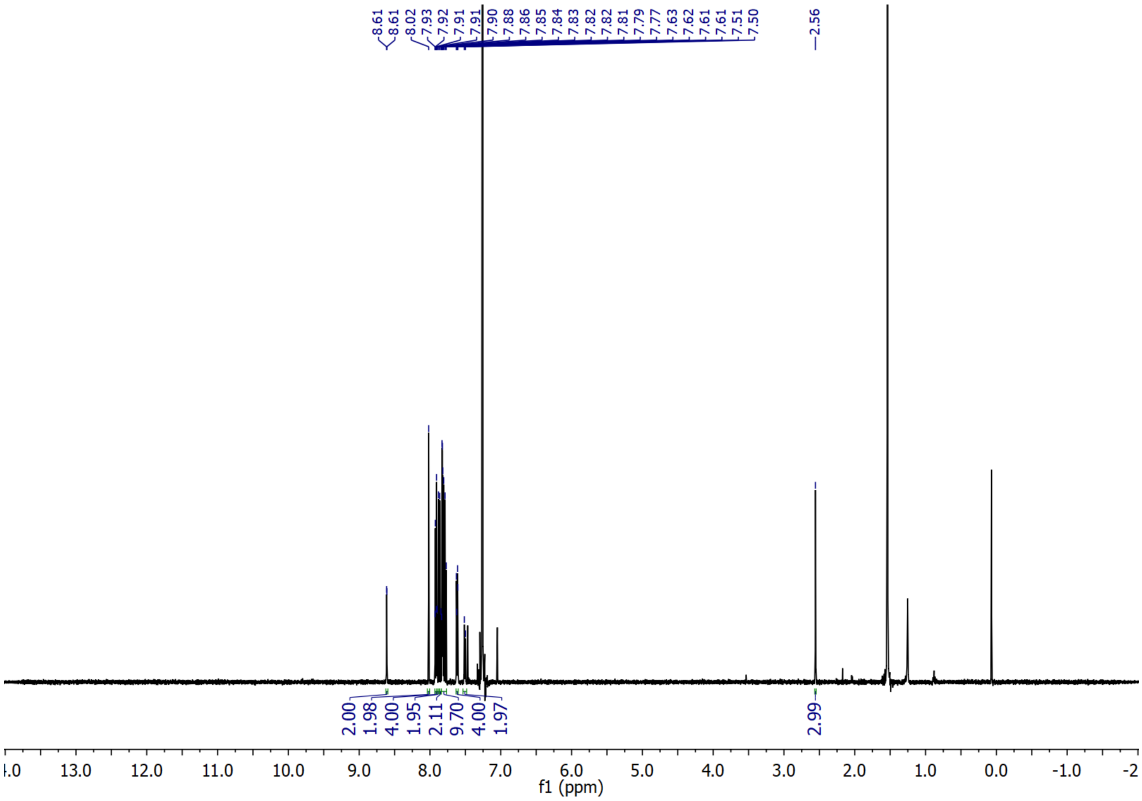

Figure S 120. Full ^{1}H-NMR (500 MHz, $CDCl_3$) spectrum of compound **16**.

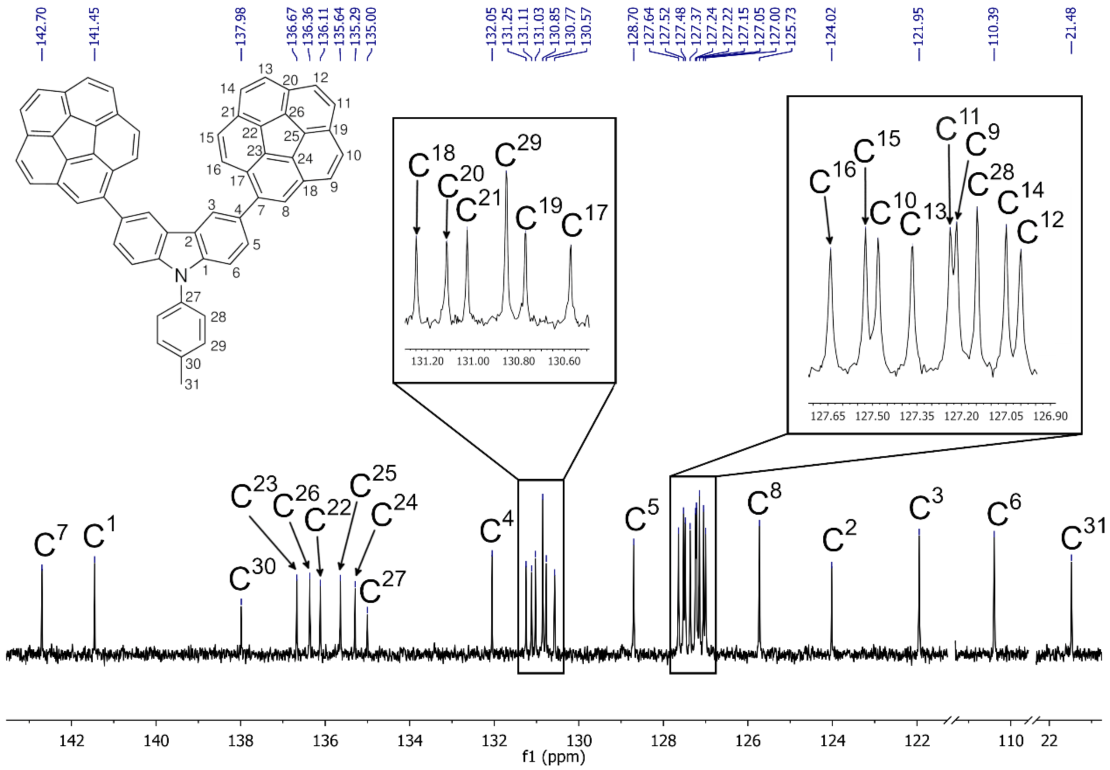

Figure S 121. $^{13}C\{^{1}H\}$-NMR (126 MHz, $CDCl_3$) spectrum of compound **16**.

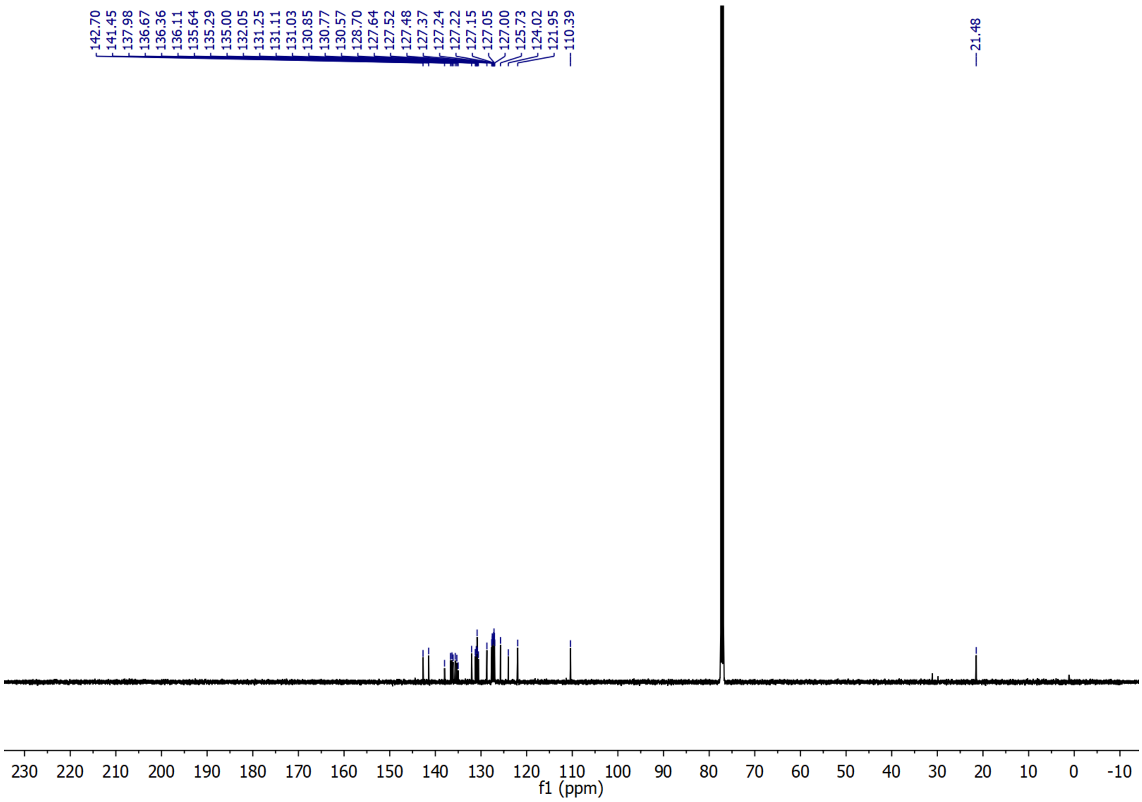

Figure S 122. Full $^{13}C\{^{1}H\}$-NMR (126 MHz, $CDCl_3$) spectrum of compound **16**.

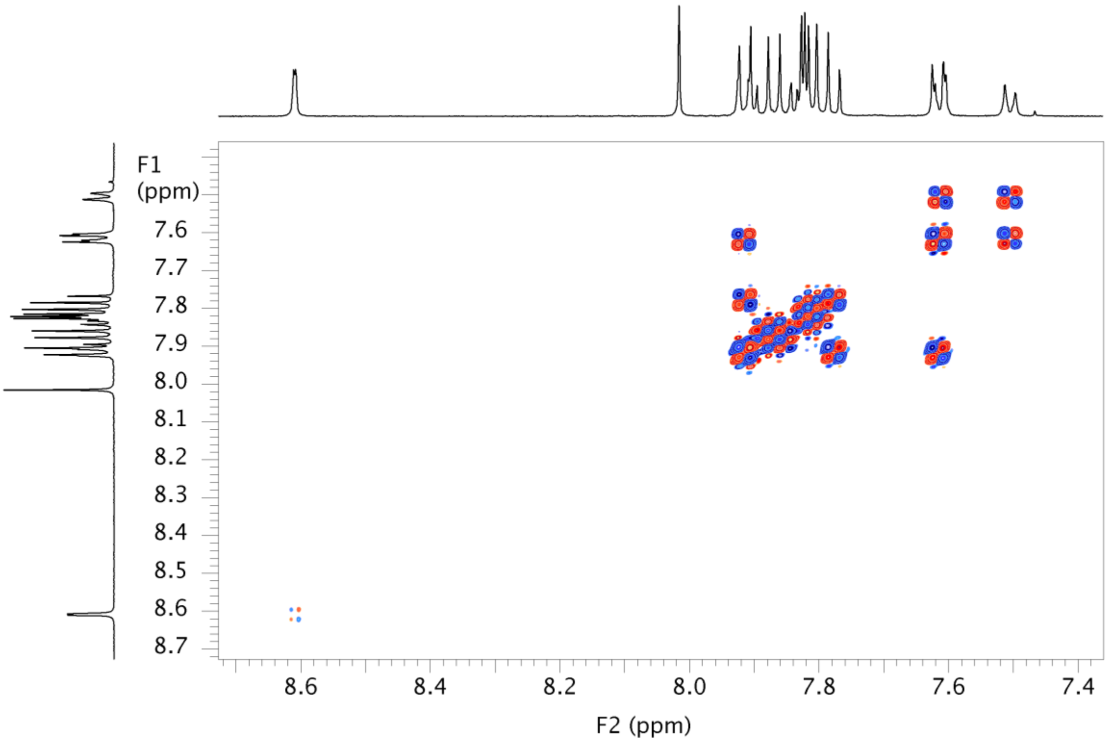

Figure S 123. ^{1}H-^{1}H gDQFCOSY (500 MHz, $CDCl_3$) spectrum of compound **16**.

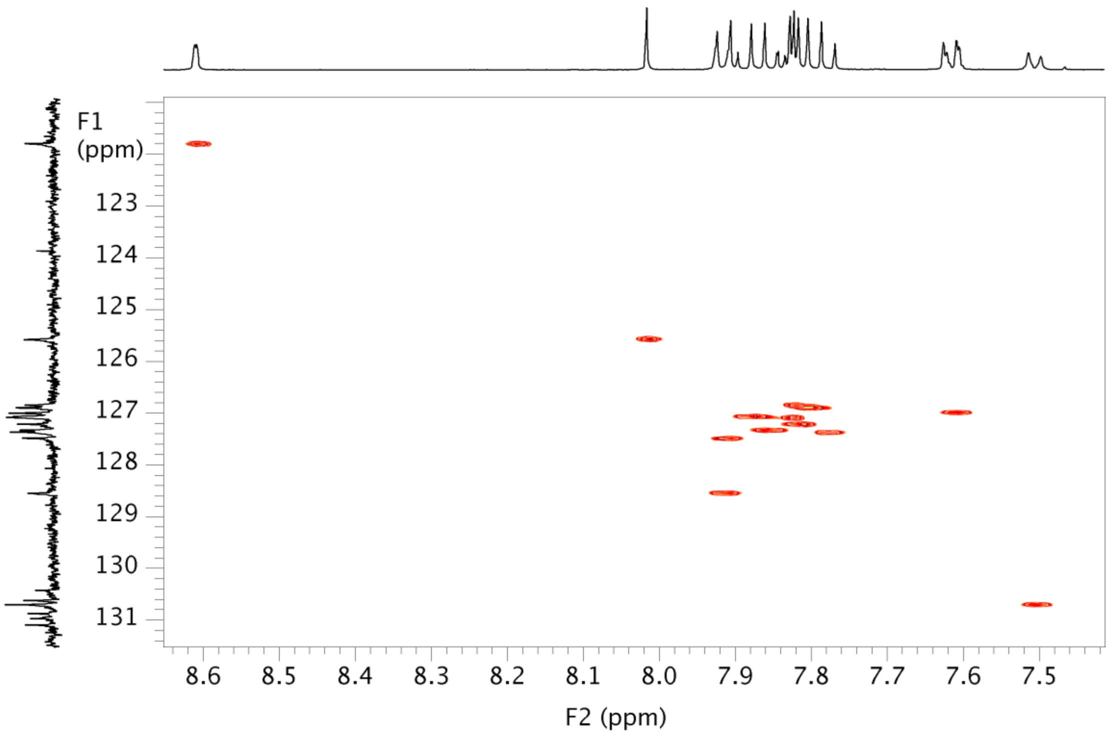

Figure S 124. ^{1}H-^{13}C bsgHSQCAD (500 MHz, $CDCl_3$) spectrum of compound **16**.

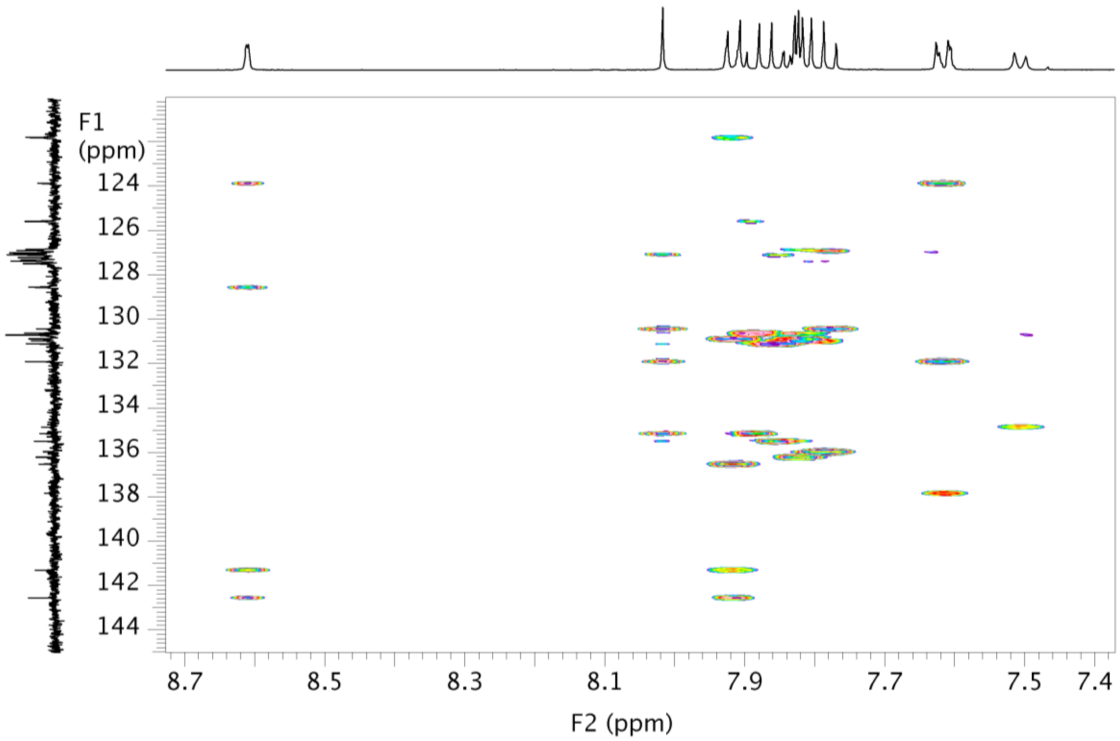

Figure S 125. ^{1}H-^{13}C bsgHMBC (500 MHz, $CDCl_3$) spectrum of compound **16**.

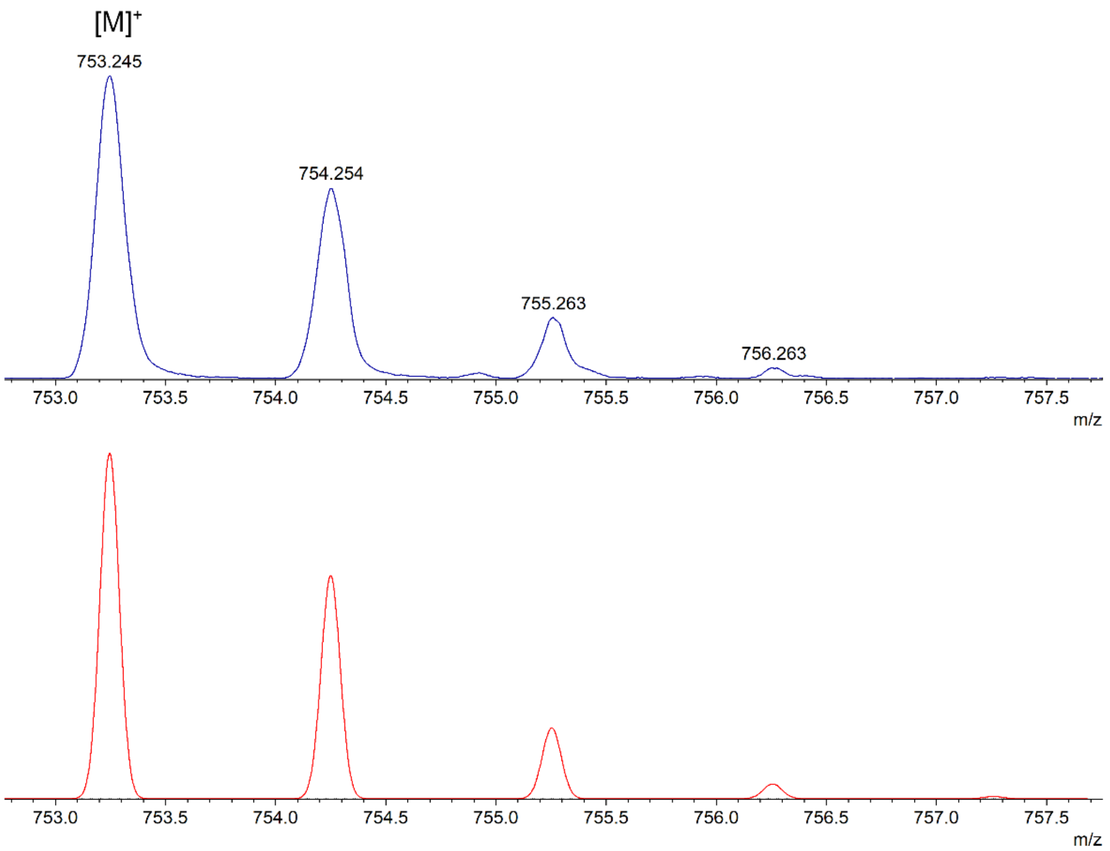

Figure S 126. HRMS (MALDI-TOF) of compound **16**, $[M]^{+}$. Calculated (red), measured (blue).

UV-Vis absorption and emission spectra

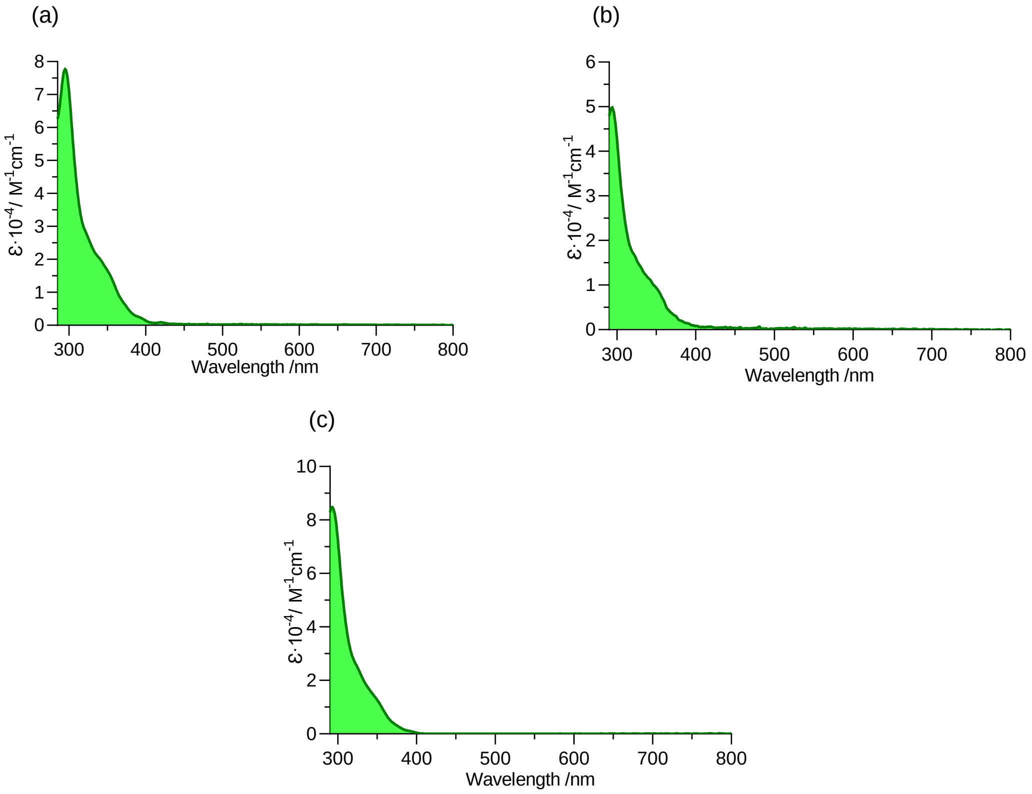

Figure S 127. UV-Vis absorption spectra of complexes (a) **4-S** (10^{-5} M), (b) **4-SO** (10^{-5} M) and (c) **4-SO$_2$** (10^{-5} M) in toluene.

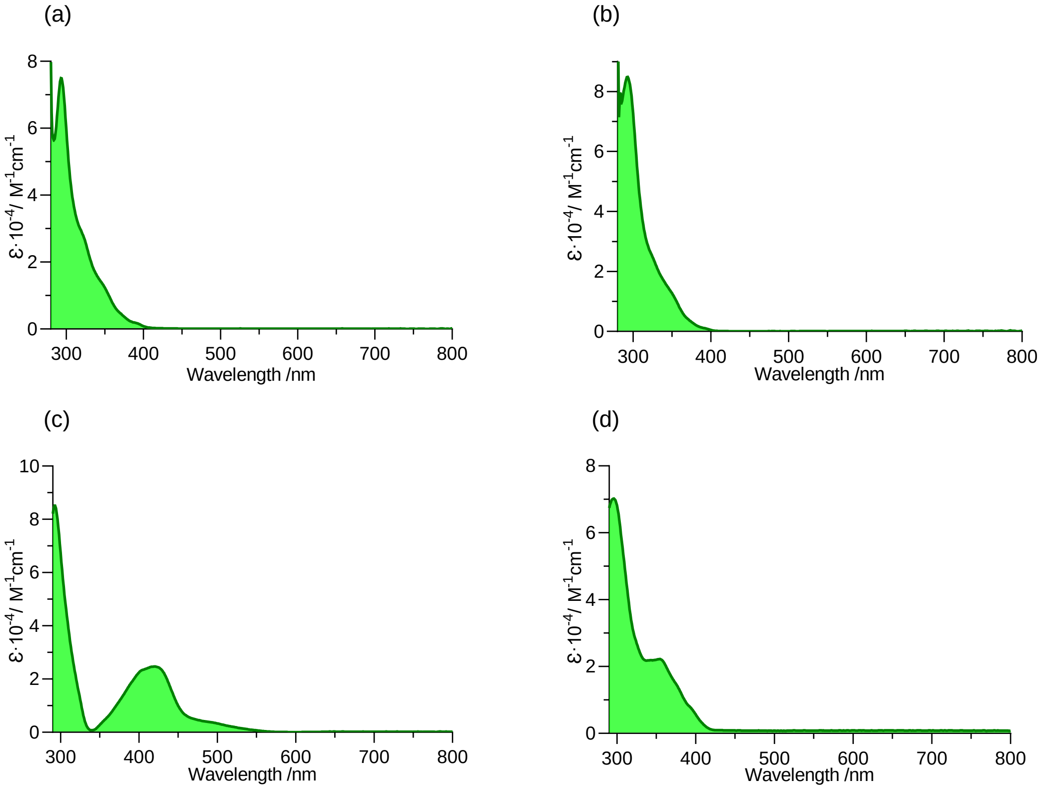

Figure S 128. UV-Vis absorption spectra of complexes (a) **11-CMe$_2$** (10^{-5} M), (b) **11-CO** (10^{-5} M), (c) **11-C(CN)$_2$** (10^{-5} M) and (d) **16** (10^{-5} M) in toluene.

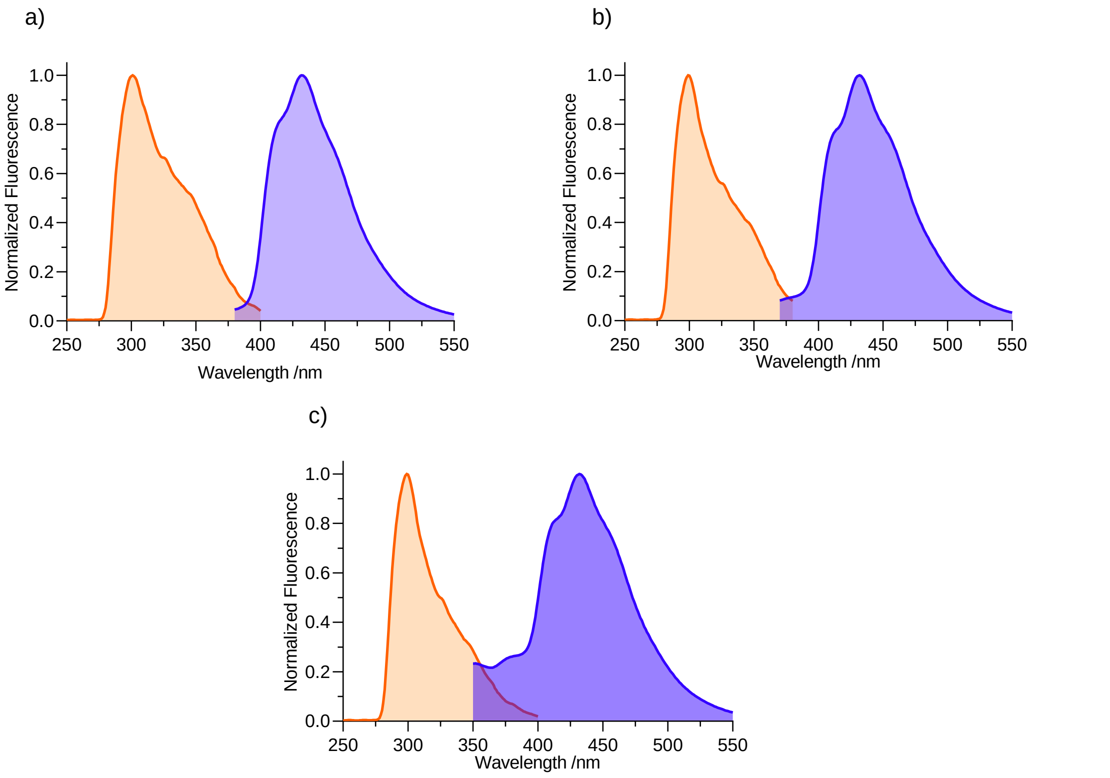

Figure S 129. Normalized excitation and emission spectra in toluene of: (a) **4-S** ($5 \cdot 10^{-6}$ M) (λ_{exc} = 301 nm), (b**) 4-SO** ($5 \cdot 10^{-6}$ M) (λ_{exc} = 299 nm) and (c) **4-SO₂** (10^{-6} M) (λ_{exc} = 299 nm).

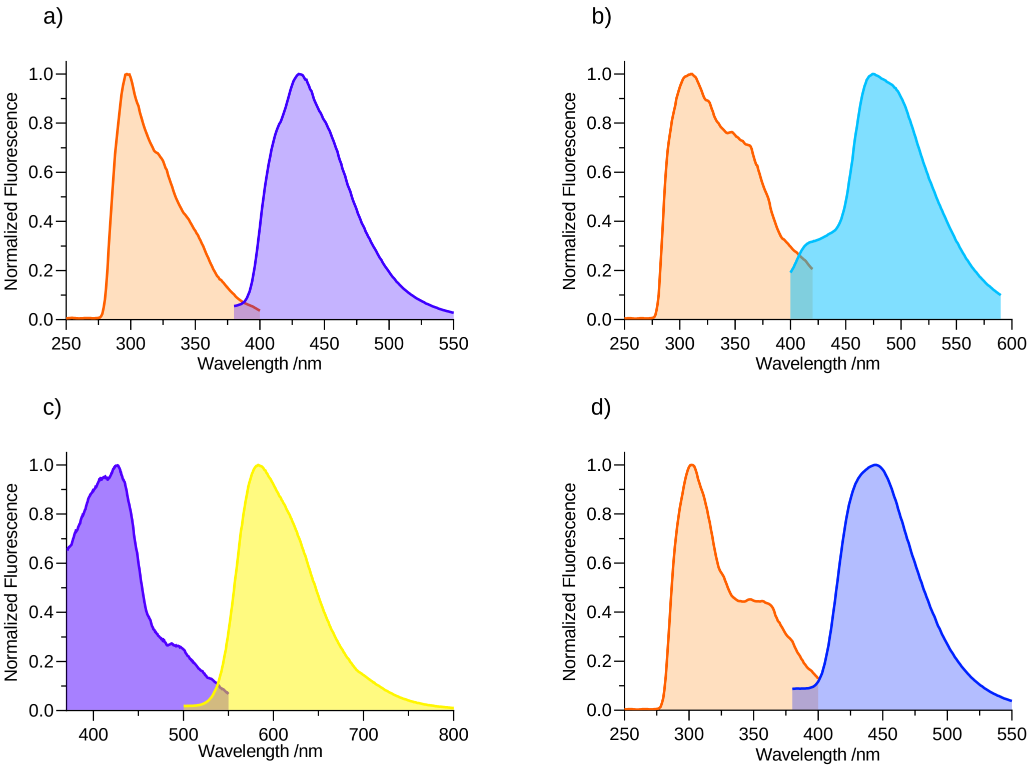

Figure S 130. Emission spectra in toluene of: (a) **11-CMe₂** ($5 \cdot 10^{-6}$ M) (λ_{exc} = 297 nm), (b) **11-CO** (10^{-5} M) (λ_{exc} = 310 nm), (c) **11-C(CN)₂** (10^{-5} M) (λ_{exc} = 426 nm) and (d) **16** (10^{-6} M) (λ_{exc} = 302 nm).

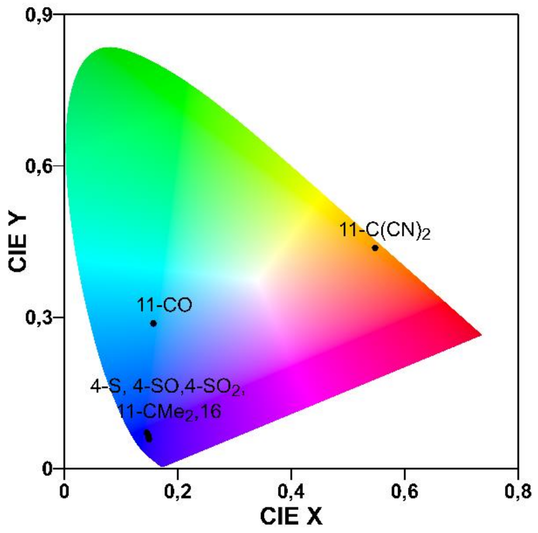

Figure S 131. Representation of the emission of molecules **4-S**, **4-SO**, **4-SO$_2$**, **11-CMe$_2$**, **11-CO**, **11-C(CN)$_2$**, and **16** in toluene in the CIE 1931 color space.

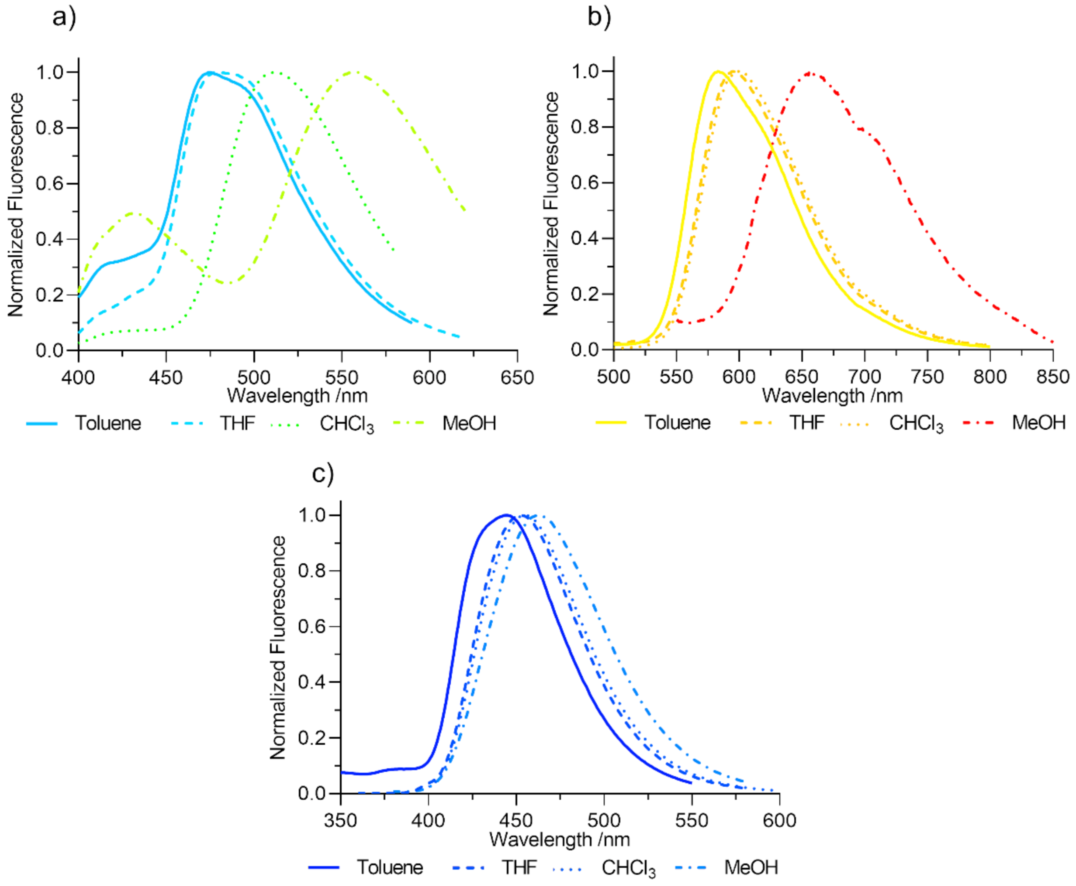

Figure S 132. Normalized emission spectra of (a) **11-CO**, (b) **11-C(CN)$_2$** and (c) **16** in solvents of incresing polarity (toluene, THF, $CHCl_3$ and MeOH).

Fluorescence decay lifetimes and Quantum Yields

Fluorescence decay lifetimes and quantum yields were measured in toluene at concentration 10^{-5} M.

Compound	Φ	τ /ns	χ^2
**4-S**	0.23	8.51	1.07
**4-SO**	0.17	9.15	1.07
**4-SO_2**	0.19	10.54	1.05
**11-CMe_2**	0.20	9.17	1.09
**11-CO**	0.04	-[a]	-[a]
**11-$C(CN)_2$**	0.01	-[a]	-[a]
**16**	0.53	5.08	1.11

[a] Below the instrument lower detection limit (1 ns).

Cyclic Voltammograms

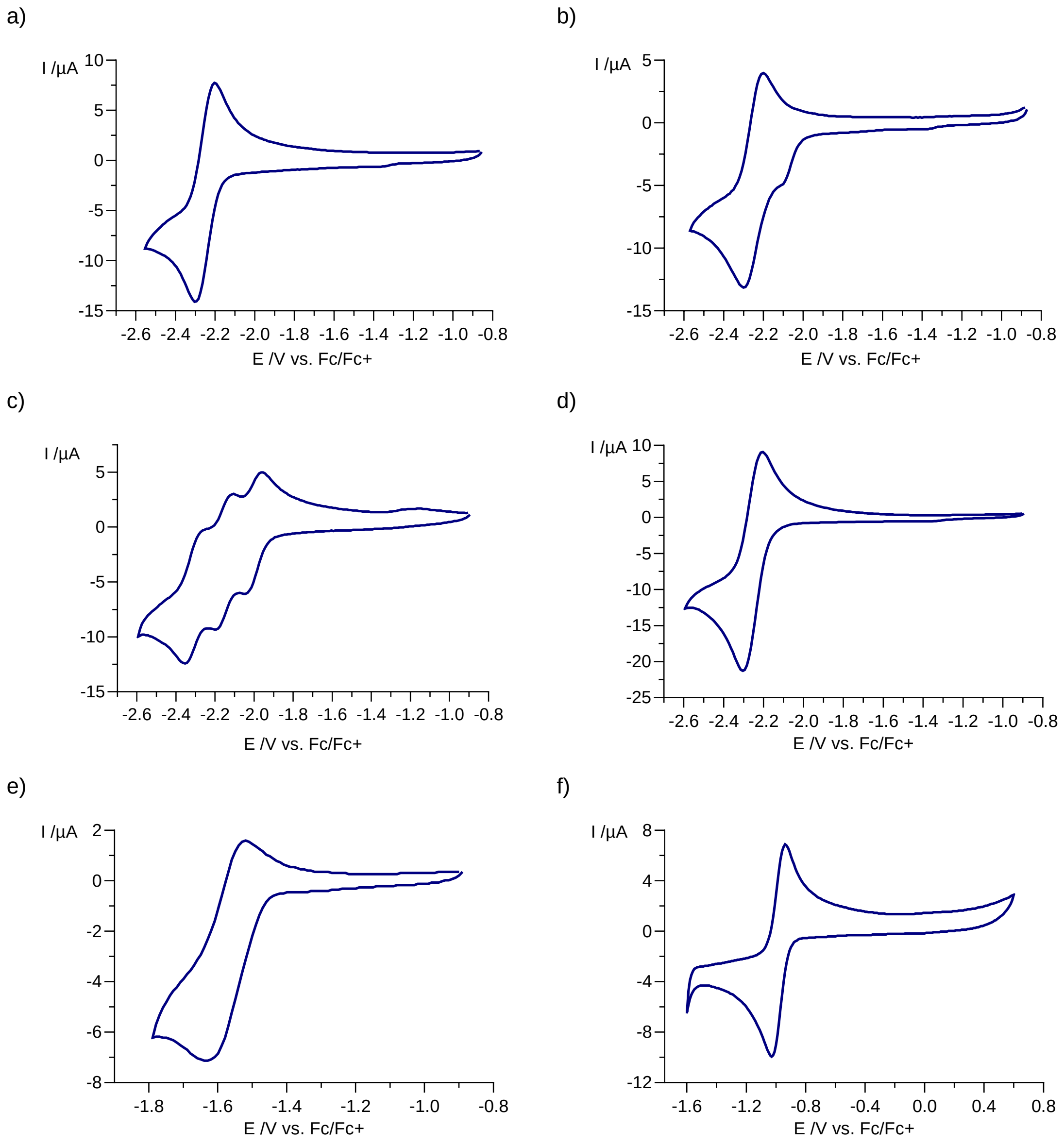

g)

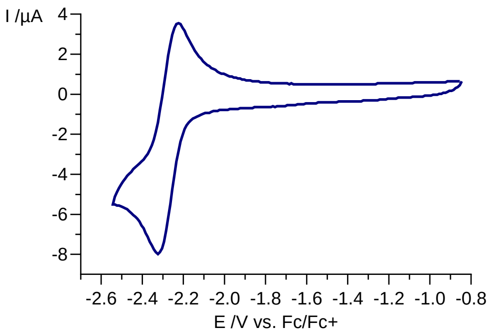

Figure S 133. Cyclic voltammograms carried out at room temperature in a one-compartment cell equipped with a glassy carbon electrode, a silver wire counter electrode, and an Ag/AgCl wire as pseudo-reference electrode of the first corannulene reductions of (a) **4-S**, (b) **4-SO**, (c) **4-SO₂**, (d) **11-CMe₂**, (e) **11-CO**, (f) **11-C(CN)₂** and (g) **16** in deaerated DMF at a concentration of 1 mM containing a solution of NBu_4PF_6 (0.1 M). Scan rate of 100 mV · s^{-1}. Potentials are referenced against Fc/Fc^+ and plotted using IUPAC convention.

Table S 1. Reduction peaks potentials at a concentration of 1 mM containing a solution of NBu_4PF_6 (0.1 M). Scan rate of 100 mV · s^{-1}. Potentials are referenced against Fc/Fc^+.

Compound	E^1 /V	E^2 /V	E^3 /V	E^4 /V
**Corannulene**	-2.31[a]	-2.86[a]	-	-
**4-S**	-2.24	-2.73[b]		
**4-SO**	-2.23	-2.73	-3.10	
**4-SO$_2$**	-2.00	-2.15	-2.29	-2.67
**11-CMe$_2$**	-2.26	-2.76[b]	-3.45[b]	-
**11-CO**	-1.49	-2.24[b]	-	-
**11-C(CN)$_2$**	-0.98	-1.64	-2.42	-3.04[b]
**16**	-2.28	-2.83[b]	-	-

[a] Reduction peaks of corannulene were reported in the literature[1]

[b] Reduction peaks are measured by square-wave voltammetry (SWV).

Table S 2. Estimated HOMO and LUMO levels of compounds **4**, **11** and **16** from experimental absorption and electrochemical data

Compound	λ_{onset} / nm	E_{gap}^{opt} /eV[a]	E^1 / V	E_{LUMO} / eV[b]	E_{LUMO}^{DFT} / eV[c]	E_{HOMO} / eV[d]	E_{HOMO}^{DFT} / eV
**4-S**	386	3.21	-2.24	-2.22	-2.29	-5.43	-4.92
**4-SO**	379	3.27	-2.23	-2.34	-2.53	-5.61	-5.13
**4-SO$_2$**	377	3.29	-2.00	-2.48	-2.66	-5.77	-5.30
**11-CMe$_2$**	378	3.28	-2.26	-2.23	-2.24	-5.51	-4.98
**11-CO**	403	3.08	-1.49	-2.93	-3.04	-6.01	-5.19
**11-C(CN)$_2$**	556	2.23	-0.98	-3.48	-3.70	-5.71	-5.30
**16**	412	3.01	-2.28	-2.21	-2.20	-5.22	-4.58

[a] Estimated by the absorption onset values: $E_{gap}^{opt} = 1240/\lambda_{onset}$

[b] Estimated based on the onset of the first reduction potential measured by CV: $E_{LUMO}=-4.4 - (E_{red}^{onset} - E_{1/2}^{Fc})$

[c] Estimated according to: $E_{HOMO} = E_{LUMO} - E_{gap}^{opt}$

[d] Calculated by DFT at the level of theory: B97D3/6-31G(d,p)/PCM(toluene)

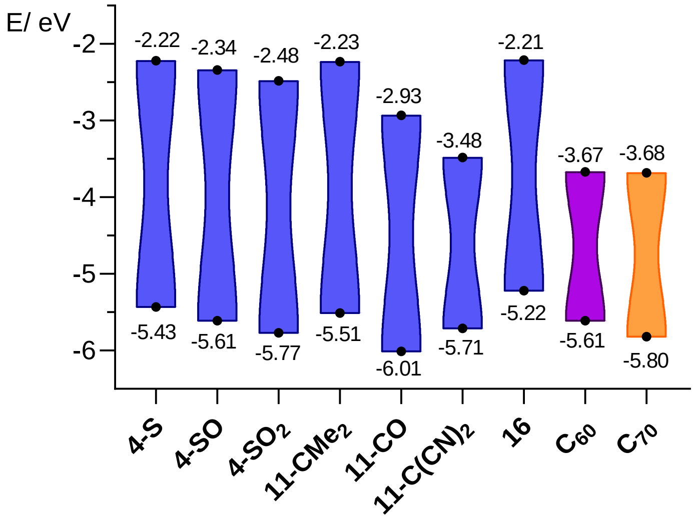

Figure S 134. HOMO/LUMO energies representation in eV of compounds **4-S**, **4-SO**, **4-SO$_2$**, **11-CMe$_2$**, **11-CO**, **11-C(CN)$_2$**, and **16**, C_{60} and C_{70}.

X-ray Crystallographic Tables

The structure of compound **15** is similar to that reported by Wu *et al.*[2] bearing a tolyl substituent instead of a phenyl group.

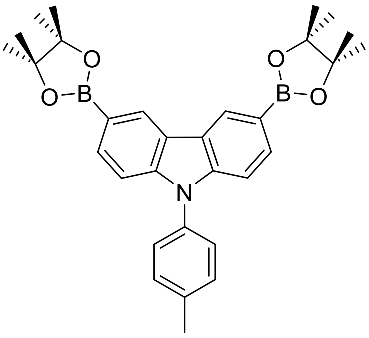

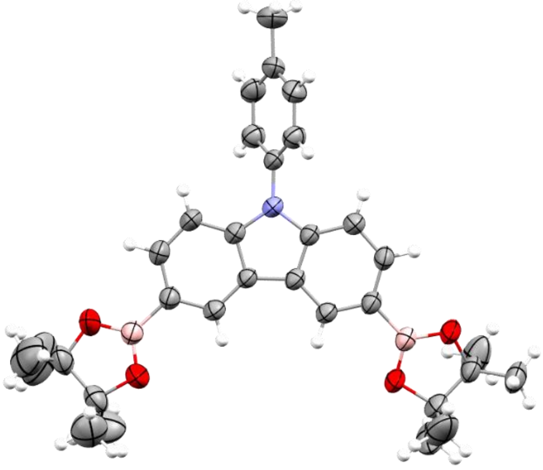

Figure S 135. Schematic representation and crystal structure of compound **15**. (50% ellipsoid contour probability). Crystallization conditions: slow diffusion of hexane onto a saturated solution of the solute in CH_2Cl_2 at -20 ºC.

Table S 3. Crystallographic data of compound **15**.

Identification code	**15**
CCDC Number	2207685
Empirical formula	$C_{31}H_{37}B_2NO_4$
Formula weight	509.23
Temperature [K]	293(2)
Crystal system	orthorhombic
Space group	Pbca
a [Å]	13.8465(6)
b [Å]	13.5213(10)
c [Å]	31.894(2)
α [°]	90
β [°]	90
γ [°]	90
V [$Å^3$]	5971.3(7)
Z	8
ρcalc[g/cm^3]	1.133
μ [mm^{-1}]	0.073
F(000)	2176.0
Crystal size [mm^3]	0.503 × 0.373 × 0.27
Radiation	Mo Kα
2θ range for data collection [°]	5.694 to 59.132
Reflections collected	22511
Independent reflections	7307 [R_{int} = 0.0429, R_{sigma} = 0.0526]
Data/restraints/parameters	7307/0/353
GOF on F^2	1.033
Final R indexes [I>=2σ (I)]	R_1 = 0.0638, wR_2 = 0.1446
Final R indexes [all data]	R_1 = 0.1361, wR_2 = 0.1832
Max/min Δρ [eÅ-3]	0.23/-0.18
Flack parameter	-

Association constants measurements

In order to estimate the association constants (K_a) of compounds **4-S**, **4-SO**, **4-SO$_2$**, **11-CMe$_2$**, **11-CO**, **11-C(CN)$_2$** and **16** with fullerenes C_{60} and C_{70}, the following procedure was carried out: a solution of each compound (1.0 × 10^{-4} M) in deuterated toluene was prepared and a known volume was transferred to an NMR tube capped with a septum (0.50 mL). The titration was carried out by injecting known portions of a stock solution of C_{60} (1.0 × 10^{-3} M) in deuterated toluene to cover a wide range of equivalents. The same protocol was followed for C_{70}. A ^{1}H-NMR spectrum was recorded at room temperature after each addition. Once all data had been obtained, the changes in chemical shifts (Δδ) of selected protons were plotted as a function of the molar fraction of the guest, and the resulting curve was fitted by a nonlinear method using the global analysis approach according to the following equations assuming the different equilibria:

A 1:1 equilibrium:

$$K_a = \frac{[HG]}{[H][G]} \quad \text{eq. 1}$$

Changes in chemical shifts upon NMR titration are expressed:

$$\Delta\delta = \Delta\delta_{max}\left(\frac{[HG]}{H_0}\right) \quad \text{eq. 2}$$

Where:

[HG] is the concentration of the guest of the complex, and is calculated using the following equation:

$$[HG] = \frac{1}{2}\left([G_0] + [H_0] + \frac{1}{K_a}\right) - \sqrt{\left([G_0] + [H_0] + \frac{1}{K_a}\right)^2 + 4[G_0][H_0]} \quad \text{eq. 3}$$

Where:

$[G_0]$ is the total concentration of the guest (C_{60} or C_{70}).

$[H_0]$ is the total concentration of the host (**4-S**, **4-SO**, **4-SO$_2$**, **11-CMe$_2$**. **11-CO**, **11-C(CN)$_2$**, **16**).

$\Delta\delta_{max}$ is Δδ at maximum complexation (100% supramolecular complex formation).

K_a is the estimated association constant for 1:1 equilibrium.

A 2:1 equilibrium:

$$K_1 = \frac{[HG]}{[H][G]} \quad \text{eq. 4}$$

$$K_2 = \frac{[H_2G]}{[HG][H]} \quad \text{eq. 5}$$

$$\beta = K_1K_2 = \frac{[H_2G]}{[H]^2[G]} \quad \text{eq. 6}$$

Changes in chemical shifts upon NMR titration are expressed:

$$\Delta\delta = \frac{\delta_{\Delta HG}K_1[G]_0[H] + 2\delta_{\Delta HG_2}K_1K_2[G]_0[H]^2}{[H]_0(1 + K_1[H] + K_1K_2[H]^2)} \quad \text{eq. 7}$$

Where [H] is the concentration of free guest, and is calculated using the following cubic equation

$$[H]^3\,(A) + [H]^2\,(B) + [H]\,(C) - [G]_0 = 0 \quad \text{eq. 8}$$

With

$$A = (K_1 K_2)$$

$$B = \{K_1(2K_2[G]_0 - K_2[H]_0 + 1)\}$$

$$C = \{K_1([G]_0 - [H]_0) + 1\}$$

Where:

$[G_0]$ is the total concentration of the guest (C_{60} or C_{70}).

$[H_0]$ is the total concentration of the host (**4-S**, **4-SO**, **4-SO$_2$**, **11-CMe$_2$**. **11-CO**, **11-C(CN)$_2$**, **16**).

$\Delta\delta_{\Delta HG}$ is Δδ at maximum complexation of the first equilibrium [HG]

$\Delta\delta_{\Delta H2G}$ is Δδ at maximum complexation of the second equilibrium [H_2G]

K_1 is the estimated association constant for the first equilibrium

K_2 is the estimated association constant for the second equilibrium

In each case, $\Delta\delta_{max}$ and K_a for a 1:1 equilibrium, $\Delta\delta\Delta_{HG}$, $\Delta\delta_{\Delta HG2}$, K_1, and K_2 for a 1:2 equilibrium and $\Delta\delta_{\Delta HG}$, $\Delta\delta_{\Delta H2G}$, K_1, and K_2 for a 2:1 equilibrium were extracted by using the non-linear fitting tool provided by the open access web portal Supramolecular.org (*http://supramolecular.org*). Links to all the fittings of the data are provided below.

Binding studies with guest C_{60}

Titrations with C_{60} were fitted to a 1 to 1 and a 2 to 1 models with different flavors being the 1 to 1 model the best fit for all our systems:

4-S vs C_{60}

1 to 1

http://app.supramolecular.org/bindfit/view/dfc886b8-1d51-4c5f-abad-739d04ca6e00

2 to 1 full

http://app.supramolecular.org/bindfit/view/4bc02ea1-44b7-474a-bc6a-af6d91ba8a4c

2 to 1 additive

http://app.supramolecular.org/bindfit/view/d2d41b19-e728-4735-abd5-a47ef57696c7

2 to 1 non cooperative

http://app.supramolecular.org/bindfit/view/0b0c572b-c28c-4cd9-939b-332c0693d0aa

2 to 1 statistical

http://app.supramolecular.org/bindfit/view/773af304-9354-47a5-8a46-b4bb3ae3c0c9

Model	K_1	K_2	Cov_{fit} ratio	$SS_y/10^{-5}$	$\chi_y/10^{-4}$
1 to 1	$(1.28 \pm 0.02) \times 10^3$	-	1	4.65	7.72
2 to 1 full	$(5.83 \pm 0.46) \times 10^3$	$(1.69 \pm 0.22) \times 10^4$	1.57	2.91	6.31
2 to 1 to additive	$(8.45 \pm 0.22) \times 10^2$	$(5.53 \pm 0.16) \times 10^2$	1.02	4.56	7.70
2 to 1 non coop	$(1.21 \pm 0.01) \times 10^3$	3.02×10^2	1.39	3.28	6.65
2 to 1 to statistical	$(1.06 \pm 0.01) \times 10^3$	2.64×10^3	1.01	4.58	7.66

4-SO vs C_{60}

1 to 1

http://app.supramolecular.org/bindfit/view/acc865e8-7925-4b7a-98da-727f1ca2b798

2 to 1 full

http://app.supramolecular.org/bindfit/view/8508abe2-42de-4c5f-835a-79a7253e9061

2 to 1 additive

http://app.supramolecular.org/bindfit/view/df8bfc48-b069-40c7-97f5-634b0aaba7dd

2 to 1 non cooperative

http://app.supramolecular.org/bindfit/view/eb6716d1-813c-4ca2-9d5f-6a9255a704f4

2 to 1 statistical

http://app.supramolecular.org/bindfit/view/237d6870-f7b7-440c-8988-28f4018842dd

Model	K_1	K_2	Cov_{fit} ratio	$SS_y/10^{-6}$	$\chi_y/10^{-4}$
1 to 1	$(1.04 \pm 0.01) \times 10^3$	-	1.00	17.4	4.7
2 to 1 full	$(1.54 \pm 0.02) \times 10^3$	$(-1.99 \pm 0.19) \times 10^3$	2.00	8.02	3.3
2 to 1 to additive	$(1.93 \pm 0.04) \times 10^3$	$(-8.12 \pm 1.02) \times 10^2$	1.19	14.9	4.4
2 to 1 non coop	$(2.40 \pm 0.03) \times 10^3$	6.01×10^2	1.90	8.4	3.4
2 to 1 to statistical	$(8.61 \pm 0.02) \times 10^2$	2.15×10^2	0.97	17.9	4.8

4-SO_2 vs C_{60}

1 to 1

http://app.supramolecular.org/bindfit/view/369848e2-2c6a-48c2-81dc-c3ed3b9edd55

2 to 1 full

http://app.supramolecular.org/bindfit/view/cf8de329-2cdc-45ea-bf5b-8b2892707df0

2 to 1 additive

http://app.supramolecular.org/bindfit/view/ef90ba55-2ac3-4342-a379-18601f8a4d3b

2 to 1 non cooperative

http://app.supramolecular.org/bindfit/view/d6ea7d85-0c61-46d0-9f57-410e890af0b2

2 to 1 statistical

http://app.supramolecular.org/bindfit/view/461ab221-becf-4584-9e04-8ecec8b32c11

Model	K_1	K_2	Cov_{fit} ratio	$SS_y/10^{-5}$	$\chi_y/10^{-3}$
1 to 1	$(1.30 \pm 0.03) \times 10^3$	-	1	7.77	1.22
2 to 1 full	$(1.77 \pm 0.19) \times 10^3$	$(7.84 \pm 5.57) \times 10^3$	1.18	6.35	1.15
2 to 1 to additive	$(8.34 \pm 0.33) \times 10^2$	$(5.84 \pm 2.67) \times 10^2$	1.03	7.52	1.21
2 to 1 non coop	$(1.10 \pm 0.02) \times 10^3$	2.75×10^2	1.02	7.56	1.24
2 to 1 to statistical	$(1.07 \pm 0.02) \times 10^3$	2.68×10^2	1.02	7.59	1.21

11-CMe_2 vs C_{60}

1 to 1

http://app.supramolecular.org/bindfit/view/4cd463f3-18aa-4e9e-bdbb-62dd4c43240f

2 to 1 full

http://app.supramolecular.org/bindfit/view/8ebed6f9-11f7-4d65-b592-7eeba56dc28b

2 to 1 additive

http://app.supramolecular.org/bindfit/view/d1092c6d-e4e3-4b7a-8c6e-77c0f7a0e891

2 to 1 non cooperative

http://app.supramolecular.org/bindfit/view/6f2e1e1e-4842-47f4-9d69-095047f9a34d

2 to 1 statistical

http://app.supramolecular.org/bindfit/view/ed6f9538-872d-4946-8e51-18658b442888

Model	K_1	K_2	Cov_{fit} ratio	$SS_y/10^{-6}$	$\chi_y/10^{-4}$
1 to 1	$(5.20 \pm 0.03) \times 10^2$	-	1	10.88	4.19
2 to 1 full	$(3.23 \pm 0.03) \times 10^0$	$(-4.23 \pm 0.21) \times 10^5$	2.76	3.93	2.63
2 to 1 to additive	$(1.46 \pm 0.33) \times 10^2$	$(-1.09 \pm 0.06) \times 10^3$	2.02	5.45	2.99
2 to 1 non coop	$(1.78 \pm 0.02) \times 10^3$	4.44×10^2	1.97	5.46	3.07
2 to 1 to statistical	$(4.49 \pm 0.02) \times 10^2$	1.12×10^2	0.98	11.15	4.24

11-CO vs C_{60}

1 to 1

http://app.supramolecular.org/bindfit/view/f794b7e2-c5f9-4d77-a741-75711e5c59c7

2 to 1 full

http://app.supramolecular.org/bindfit/view/41db2b31-f18b-44b6-922d-2be118a8be87

2 to 1 additive

http://app.supramolecular.org/bindfit/view/b48b3f4e-13fb-448a-b688-bd632426af4d

2 to 1 non cooperative

http://app.supramolecular.org/bindfit/view/65a3e45f-93f2-46ed-aeb9-11281563a776

2 to 1 statistical

http://app.supramolecular.org/bindfit/view/3880f83d-3eb5-46e2-a500-0ce6b9d27b80

Model	K_1	K_2	Cov_{fit} ratio	$SS_y/10^{-5}$	$\chi_y/10^{-4}$
1 to 1	$(5.07 \pm 0.08) \times 10^2$	-	1.00	3.78	7.81
2 to 1 full	$(2.61 \pm 0.04) \times 10^0$	$(-8.65 \pm 0.71) \times 10^5$	2.54	1.49	5.12
2 to 1 to additive	$(1.74 \pm 0.33) \times 10^3$	$(-1.31 \pm 0.15) \times 10^3$	1.27	2.98	6.99
2 to 1 non coop	$(1.89 \pm 0.02) \times 10^3$	4.73×10^2	1.53	2.47	6.53
2 to 1 to statistical	$(4.36 \pm 0.02) \times 10^2$	1.09×10^2	0.99	3.81	7.84

11-C(CN)$_2$ vs C_{60}

1 to 1

http://app.supramolecular.org/bindfit/view/d6ef1001-0503-4f9a-b31b-df3a3da77845

2 to 1 full

http://app.supramolecular.org/bindfit/view/934729bc-d449-492f-9f80-6dc55cab8e82

2 to 1 additive

http://app.supramolecular.org/bindfit/view/9762eb2f-2a96-4c7e-af05-f64dfb89bd33

2 to 1 non cooperative

http://app.supramolecular.org/bindfit/view/b2f0ef48-98da-44ed-8fdf-346f160bf303

2 to 1 statistical

http://app.supramolecular.org/bindfit/view/970cfc73-f609-4ba9-a5eb-d3b7eff64835

Model	K_1	K_2	Cov_{fit} ratio	$SS_y/10^{-6}$	$\chi_y/10^{-4}$
1 to 1	$(7.35 \pm 0.05) \times 10^2$	-	1	18.68	5.49
2 to 1 full	$(3.33 \pm 0.03) \times 10^{-1}$	$(-2.24 \pm 0.09) \times 10^6$	2.87	6.45	3.36
2 to 1 to additive	$(1.59 \pm 0.02) \times 10^3$	$(-9.16 \pm 0.15) \times 10^2$	1.627	11.71	4.38
2 to 1 non coop	$(1.93 \pm 0.02) \times 10^3$	4.82×10^2	1.707	10.80	4.32
2 to 1 to statistical	$(6.24 \pm 0.04) \times 10^2$	1.56×10^2	0.96	19.45	5.`

16 vs C_{60}

1 to 1

http://app.supramolecular.org/bindfit/view/774dcc23-7f51-48b9-bc00-7a27a7b98ada

2 to 1 full

http://app.supramolecular.org/bindfit/view/cc95e461-c1a7-4a90-a8e3-b88d18234fac

2 to 1 additive

http://app.supramolecular.org/bindfit/view/82c4da04-3364-4de1-9d02-3cc0476aeea9

2 to 1 non cooperative

http://app.supramolecular.org/bindfit/view/004e63af-906a-4bbc-bcbf-886a0a742284

2 to 1 statistical

http://app.supramolecular.org/bindfit/view/da46a77b-15ca-401c-a60e-86d401cf09d7

**Model**	**K_1**	**º**	**Cov_{fit} ratio**	**$SS_y/10^{-6}$**	**$\chi_y/10^{-4}$**
1 to 1	$(5.28 \pm 0.04) \times 10^2$	-	1.00	12.02	3.92
2 to 1 full	$(3.49 \pm 0.04) \times 10^{-1}$	$(-1.98 \pm 0.13) \times 10^6$	1.81	6.64	3.02
2 to 1 to additive	$(1.12 \pm 0.02) \times 10^3$	$(-6.91 \pm 0.76) \times 10^2$	1.14	10.70	3.73
2 to 1 non coop	$(1.28 \pm 0.01) \times 10^3$	3.19×10^2	1.48	8.07	3.30
2 to 1 to statistical	$(4.52 \pm 0.03) \times 10^2$	1.13×10^2	0.99	12.16	3.95

Table S 4. Estimated K_a values calculated from selected protons in each compound (in M^{-1}).

	**K_a**
**4-S**	$(1.28 \pm 0.02) \times 10^3$
**4-SO**	$(1.04 \pm 0.01) \times 10^3$
**4-SO_2**	$(1.30 \pm 0.03) \times 10^3$
**11-CMe_2**	$(5.20 \pm 0.03) \times 10^2$
**11-CO**	$(5.07 \pm 0.08) \times 10^2$
**11-$C(CN)_2$**	$(7.35 \pm 0.05) \times 10^2$
**16**	$(5.28 \pm 0.04) \times 10^2$

Hammett constants[3] were chosen according to a simplified version of the substitution pattern of a phenylene corannulene group. *para* substitution (σ_p) corresponded to the E-C_6H_5 substituent, being E the bridgehead group (S in host **4-S**, for instance), whereas meta substitution (σ_m) corresponded to phenyl (C_6H_5) substituent in all cases.

Table S 5. Hammett constants of compounds **4**, **11** and **16** along with their corresponding experimental binding Gibbs free energy.

Compound	σ_p	σ_m	$\sigma_p + \sigma_m$	ΔG_a / kJ · mol^{-1}
**4-S**	0.07	0.06	0.13	-17.73 ± 0.04
**4-SO**	0.49	0.06	0.55	-17.22 ± 0.02
**4-SO$_2$**	0.77	0.06	0.83	-17.77 ± 0.06
**11-CMe$_2$**	-0.18	0.06	-0.12	-15.5 ± 0.1
**11-CO**	0.43	0.06	0.49	-15.4 ± 0.4
**11-C(CN)$_2$**	0.84	0.06	0.90	-16.3 ± 0.2
**16**	-0.22	0.06	-0.16	-15.5 ± 0.2

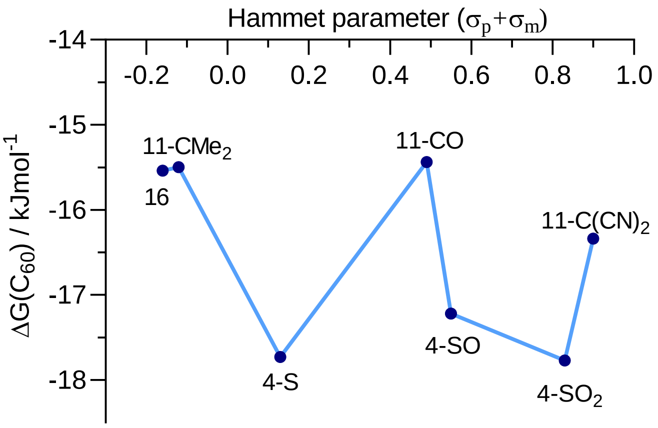

Figure S 136. Plot of experimental supramolecular Gibbs free energy versus Hammet parameter of the formation of the inclusion complexes between the hosts prepared in this work and C_{60}..

Binding studies with guest C_{70}

4-S vs C_{70}

1 to 1

http://app.supramolecular.org/bindfit/view/b57830c9-96ab-45a8-86fe-bb9f9b41d6bb

2 to 1 full

http://app.supramolecular.org/bindfit/view/16cd511f-acf3-4d2e-b125-8f368bc4c263

2 to 1 additive

http://app.supramolecular.org/bindfit/view/047432e5-6979-4d30-b236-40c5fe039e73

2 to 1 non cooperative

http://app.supramolecular.org/bindfit/view/ecd1e311-0d0f-491b-adda-10068b7d6462

2 to 1 statistical

http://app.supramolecular.org/bindfit/view/ba251818-9517-4931-899b-aadd61d6bf55

Model	K_1	K_2	Cov_{fit} ratio	$SS_y/10^{-5}$	$\chi_y/10^{-4}$
1 to 1	$(1.98 \pm 0.05) \times 10^3$	-	1.00	33.47	18.03
2 to 1 full	$(2.86 \pm 0.02) \times 10^3$	$(9.38 \pm 0.29) \times 10^2$	17.30	1.66	4.13
2 to 1 to additive	$(5.16 \pm 0.20) \times 10^2$	$(1.93 \pm 0.31) \times 10^3$	1.01	33.28	18.06
**2 to 1 non coop**	**$(2.78 \pm 0.02) \times 10^3$**	**6.96×10^2**	**17.27**	**1.66**	**4.11**
2 to 1 to statistical	$(1.55 \pm 0.03) \times 10^3$	3.87×10^3	1.00	33.58	18.06

Comparing the fits of 1 to 1 and 2 to 1 models (in different flavors), it appears 2 to 1 full and 2 to 1 non cooperative have a cov_{fit} ratio over 3 (roughly 17). This means that models 2 to 1 full and non-cooperative fit statistically better the data than model 1 to 1 and, according to an F-test, 2 to 1 non cooperative flavor is inferred (see the main text).

4-SO vs C_{70}

1 to 1

http://app.supramolecular.org/bindfit/view/1fa72a4d-19ee-41e2-a65c-a89582e3e585

2 to 1 full

http://app.supramolecular.org/bindfit/view/2b769e00-46fa-4cc6-8a3f-49e3206428b5

2 to 1 additive

http://app.supramolecular.org/bindfit/view/3a854430-068e-434e-ab1b-3f50907ab1b6

2 to 1 non cooperative

http://app.supramolecular.org/bindfit/view/8beb464e-d59c-4ba3-9b10-20bc50d091ad

2 to 1 statistical

http://app.supramolecular.org/bindfit/view/4d8e91ae-03d8-4788-b3b8-2ba457aae7a2

Model	K_1	K_2	Cov_{fit} ratio	$SS_y/10^{-5}$	$\chi_y/10^{-4}$
1 to 1	$(2.14 \pm 0.03) \times 10^3$	-	1.00	3.68	2.83
2 to 1 full	$(2.52 \pm 0.03) \times 10^3$	$(-1.50 \pm 0.27) \times 10^3$	1.75	2.04	2.20
2 to 1 to additive	$(3.10 \pm 0.07) \times 10^3$	$(-6.14 \pm 0.99) \times 10^3$	1.23	2.97	2.57
2 to 1 non coop	$(5.09 \pm 0.02) \times 10^2$	1.27×10^2	1.63	2.18	2.25
2 to 1 to statistical	$(1.65 \pm 0.02) \times 10^3$	4.12×10^2	0.87	4.21	3.02

4-SO_2 vs C_{70}

1 to 1

http://app.supramolecular.org/bindfit/view/28f86a27-e64d-446e-8250-ef9700c52718

2 to 1 full

http://app.supramolecular.org/bindfit/view/2a9c16e4-177f-4dec-8ffa-2419e694c2ea

2 to 1 additive

http://app.supramolecular.org/bindfit/view/a503e70f-5042-4116-bbf7-cbb4d2a78de7

2 to 1 non cooperative

http://app.supramolecular.org/bindfit/view/436215eb-13b6-4a12-8e4c-8ddb059df528

2 to 1 statistical

http://app.supramolecular.org/bindfit/view/14b49dc5-1321-468e-b43e-906cffe61359

Model	K_1	K_2	Cov_{fit} ratio	$SS_y/10^{-5}$	$\chi_y/10^{-4}$
1 to 1	$(1.95 \pm 0.03) \times 10^3$	-	1	7.24	10.81
2 to 1 full	$(7.27 \pm 0.06) \times 10^{-2}$	$(4.00 \pm 0.29) \times 10^6$	6.68	1.07	4.33
2 to 1 to additive	$(3.75 \pm 0.10) \times 10^3$	$(-1.15 \pm 0.31) \times 10^3$	1.71	4.17	8.27
2 to 1 non coop	$(2.10 \pm 0.01) \times 10^1$	5.24×10^0	6.41	1.12	4.38
2 to 1 to statistical	$(1.54 \pm 0.02) \times 10^3$	3.84×10^2	0.90	8.07	11.41

11-CMe_2 vs C_{70}

1 to 1

http://app.supramolecular.org/bindfit/view/c7c6f544-1793-4c55-9d53-e6c33a8b3536

2 to 1 full

http://app.supramolecular.org/bindfit/view/4dcfb693-f84f-497b-8438-a6ee9ea56472

2 to 1 additive

http://app.supramolecular.org/bindfit/view/a007e4bf-b054-4766-9bf1-5b9ac02c35b1

2 to 1 non cooperative

http://app.supramolecular.org/bindfit/view/1c078750-6298-490b-96b2-43c088a15704

2 to 1 statistical

http://app.supramolecular.org/bindfit/view/71b66700-8106-47de-ad5f-5aa18d040aff

Model	K_1	K_2	Cov_{fit} ratio	$SS_y/10^{-5}$	$\chi_y/10^{-4}$
1 to 1	$(1.55 \pm 0.02) \times 10^3$	-	1.00	5.32	9.26
2 to 1 full	$(3.49 \pm 0.03) \times 10^{-1}$	$(1.37 \pm 0.04) \times 10^6$	3.55	1.50	5.12
2 to 1 to additive	$(2.91 \pm 0.05) \times 10^3$	$(-1.03 \pm 0.75) \times 10^3$	1.90	2.80	6.77
2 to 1 non coop	$(6.88 \pm 0.02) \times 10^1$	1.72×10^1	3.43	1.55	5.16
2 to 1 to statistical	$(1.25 \pm 0.01) \times 10^3$	3.14×10^2	0.89	5.93	9.78

11-CO vs C_{70}

1 to 1

http://app.supramolecular.org/bindfit/view/6507a91f-6f9b-43a2-82a5-84a55804ad1c

2 to 1 full

http://app.supramolecular.org/bindfit/view/f6e6d41f-f235-4493-a584-cefdc20de637

2 to 1 additive

http://app.supramolecular.org/bindfit/view/c854b85f-1d5b-45f8-b9af-e6f935ef5fbe

2 to 1 non cooperative

http://app.supramolecular.org/bindfit/view/9fb30874-8e91-4418-b0cb-5ed5f8307d1d

2 to 1 statistical

http://app.supramolecular.org/bindfit/view/259bf192-b1fc-45d8-9184-dd6ab9fd69b0

Model	K_1	K_2	Cov_{fit} ratio	$SS_y/10^{-6}$	$\chi_y/10^{-4}$
1 to 1	$(1.23 \pm 0.01) \times 10^3$	-	1.00	39.35	7.97
2 to 1 full	$(7.56 \pm 0.05) \times 10^{-1}$	$(-5.58 \pm 0.13) \times 10^5$	6.65	5.99	3.24
2 to 1 to additive	$(2.63 \pm 0.03) \times 10^3$	$(-1.12 \pm 0.05) \times 10^3$	2.98	13.34	4.68
2 to 1 non coop	$(-7.59 \pm 0.01) \times 10^1$	-1.90×10^1	6.76	5.89	3.19
2 to 1 to statistical	$(1.00 \pm 0.01) \times 10^3$	2.51×10^2	0.91	43.38	8.37

11-C(CN)$_2$ vs C_{70}

1 to 1

http://app.supramolecular.org/bindfit/view/23e95ef5-6ae3-4b89-a0cc-931b88217984

2 to 1 full

http://app.supramolecular.org/bindfit/view/612314d3-dc69-4919-9144-113e89a875cb

2 to 1 additive

http://app.supramolecular.org/bindfit/view/4652014a-eb2f-404d-84c3-c098959a3c9f

2 to 1 non cooperative

http://app.supramolecular.org/bindfit/view/a84caca1-5d75-43c9-b2ea-1e4847446ab3

2 to 1 statistical

http://app.supramolecular.org/bindfit/view/14b35ff2-4209-4e03-82a7-2e5d94ffd663

Model	K_1	K_2	Cov_{fit} ratio	$SS_y/10^{-6}$	$\chi_y/10^{-4}$
1 to 1	$(1.69 \pm 0.01) \times 10^3$	-	1.00	32.89	5.92
2 to 1 full	$(3.11 \pm 0.02) \times 10^3$	$(-3.62 \pm 0.01) \times 10^2$	3.89	8.49	3.12
2 to 1 to additive	$(2.86 \pm 0.02) \times 10^3$	$(-8.30 \pm 0.27) \times 10^2$	3.63	9.05	3.12
2 to 1 non coop	$(2.94 \pm 0.01) \times 10^2$	7.34×10^1	3.08	1.07	3.49
2 to 1 to statistical	$(1.34 \pm 0.01) \times 10^3$	3.35×10^2	0.79	4.15	6.65

16 vs C_{70}

1 to 1

http://app.supramolecular.org/bindfit/view/d3c19d8f-84c2-42f2-94fd-b71d6dc555a0

2 to 1 full

http://app.supramolecular.org/bindfit/view/24b56bc5-7358-4155-82dc-24b2977bc8f8

2 to 1 additive

http://app.supramolecular.org/bindfit/view/88b3a5e7-7a59-445d-8e6a-188b445d1ba2

2 to 1 non cooperative

http://app.supramolecular.org/bindfit/view/609494ab-b148-46a8-ac5d-37154722888a

2 to 1 statistical

http://app.supramolecular.org/bindfit/view/ff85babe-57b7-4817-b863-7345f36836b7

Model	K_1	K_2	Cov_{fit} ratio	$SS_y/10^{-5}$	$\chi_y/10^{-4}$
1 to 1	$(1.72 \pm 0.02) \times 10^3$	-		6.02	10.18
2 to 1 full	$(2.65 \pm 0.02) \times 10^2$	$(1.68 \pm 0.06) \times 10^2$	2.64	2.28	6.57
2 to 1 to additive	$(2.68 \pm 0.04) \times 10^3$	$(-7.24 \pm 0.74) \times 10^2$	1.43	4.23	8.62
2 to 1 non coop	$(2.70 \pm 0.01) \times 10^2$	6.74×10^1	2.64	2.29	6.51
2 to 1 to statistical	$(1.37 \pm 0.01) \times 10^3$	3.42×10^2	0.88	6.85	10.87

Table S 6. Estimated K_{11} and K_{21} values calculated from selected protons in each compound (non cooperative model only) (in M^{-1}).

	K_{11}	K_{21}
**4-S**	$2.78 \pm 0.02 \times 10^3$	6.96×10^2
**4-SO**	$5.09 \pm 0.02 \times 10^2$	1.27×10^2
**4-SO$_2$**	$2.10 \pm 0.01 \times 10^1$	5.24×10^0
**11-CMe$_2$**	$6.88 \pm 0.02 \times 10^1$	1.72×10^1
**11-CO**	$1.23 \pm 0.01 \times 10^3$	-
**11-C(CN)$_2$**	$2.94 \pm 0.01 \times 10^2$	7.34×10^2
**16**	$2.70 \pm 0.01 \times 10^2$	6.74×10^2

4-S vs C_{60}

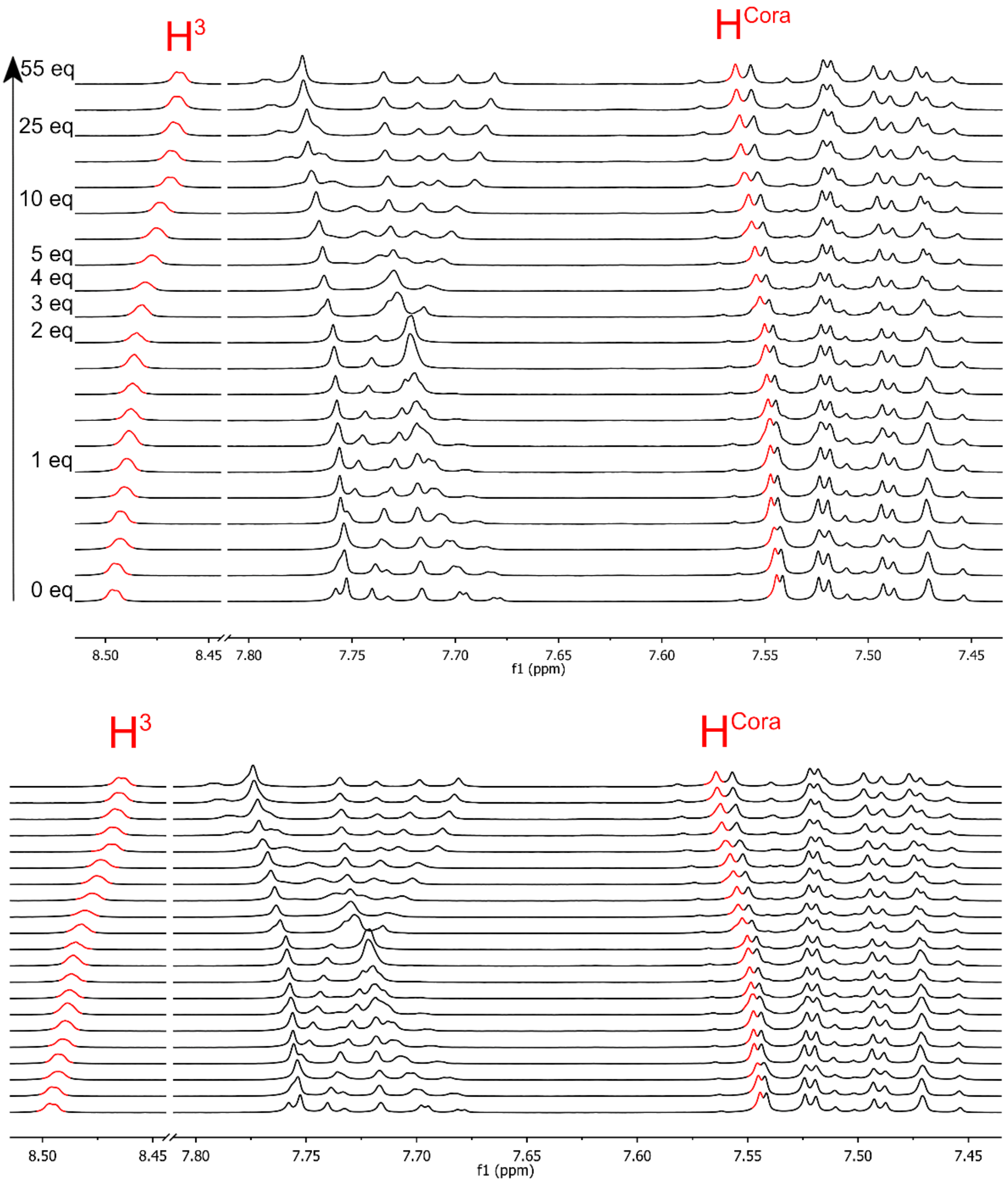

Figure S 137. Stacked ^{1}H-NMR (500 MHz, toluene-d_8) spectra for the titration of **4-S** with variable concentrations of C_{60}. in toluene-d_8 at 298 K.

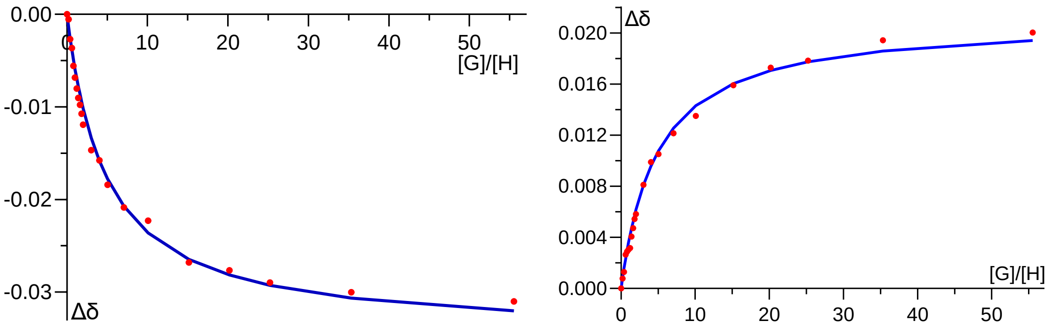

Figure S 138. Nonlinear regressions for selected protons (left plot: H^3, right plot: H^{Cora}) for the titration of **4-S** with C_{60}.

4-S vs C_{70} (2 to 1 non cooperative)

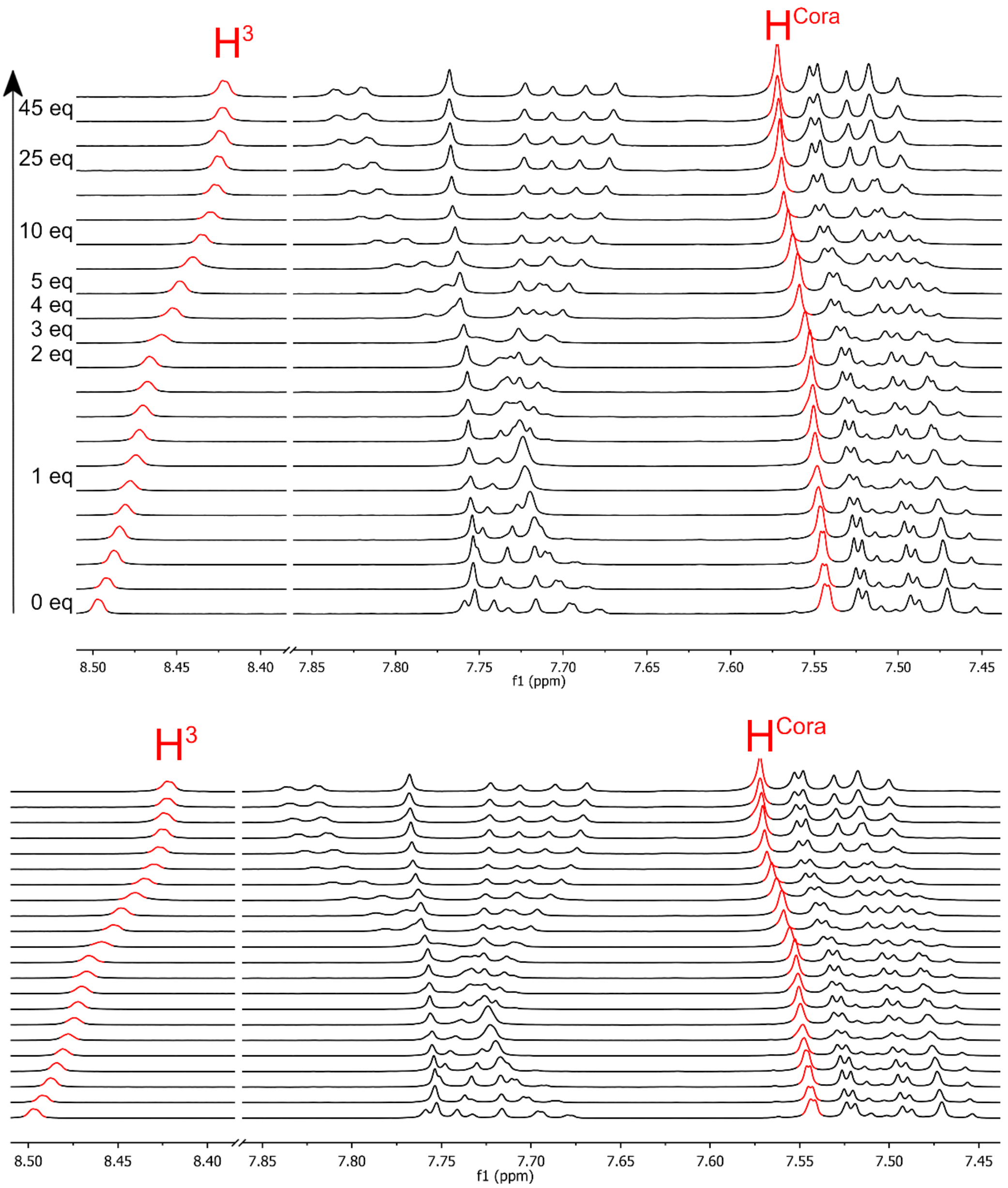

Figure S 139. Stacked ^{1}H-NMR (500 MHz, toluene-d_8) spectra for the titration of **4-S** with variable concentrations of C_{70} in toluene-d_8 at 298 K.

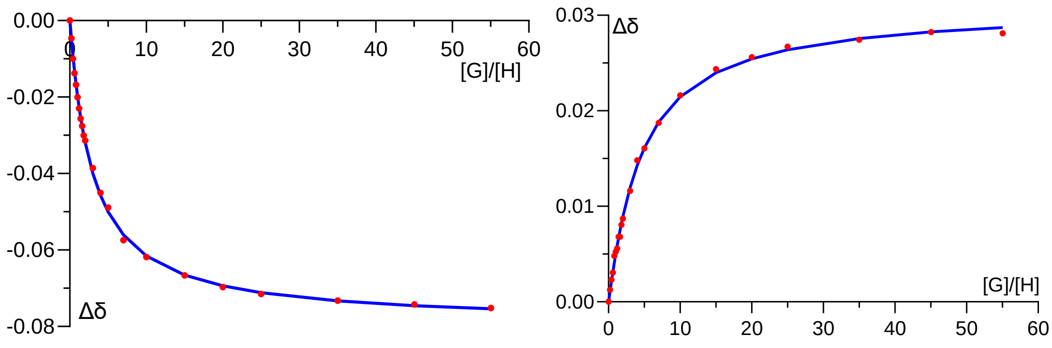

Figure S 140. Nonlinear regressions for selected protons (left plot: H^3, right plot: H^{Cora}) for the titration of **4-S** with C_{70}.

4-SO vs C_{60}

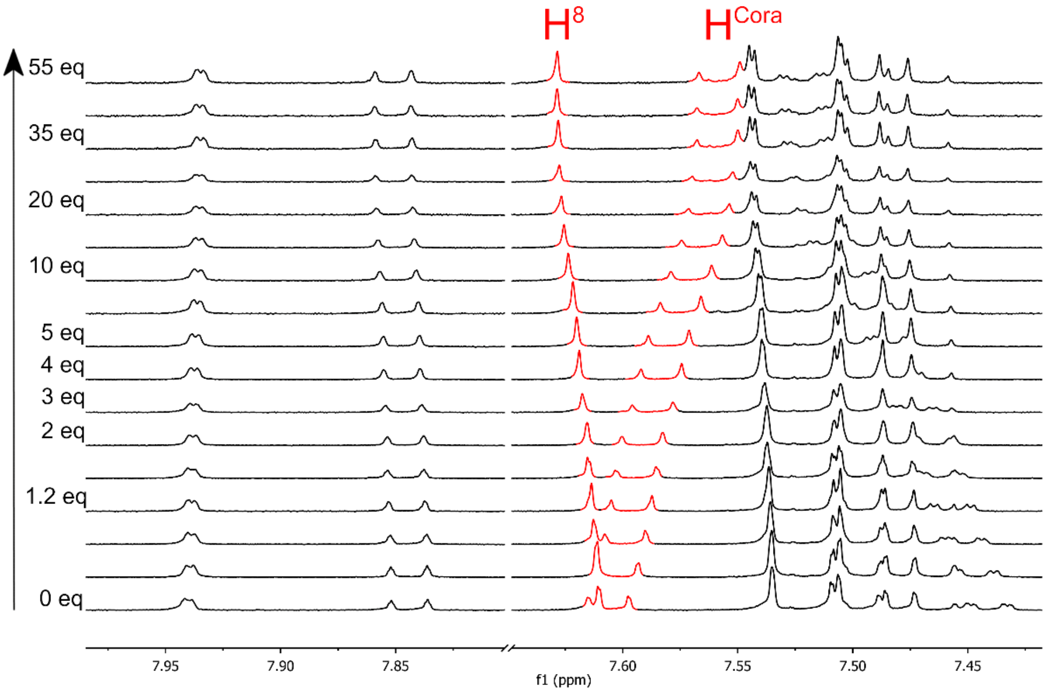

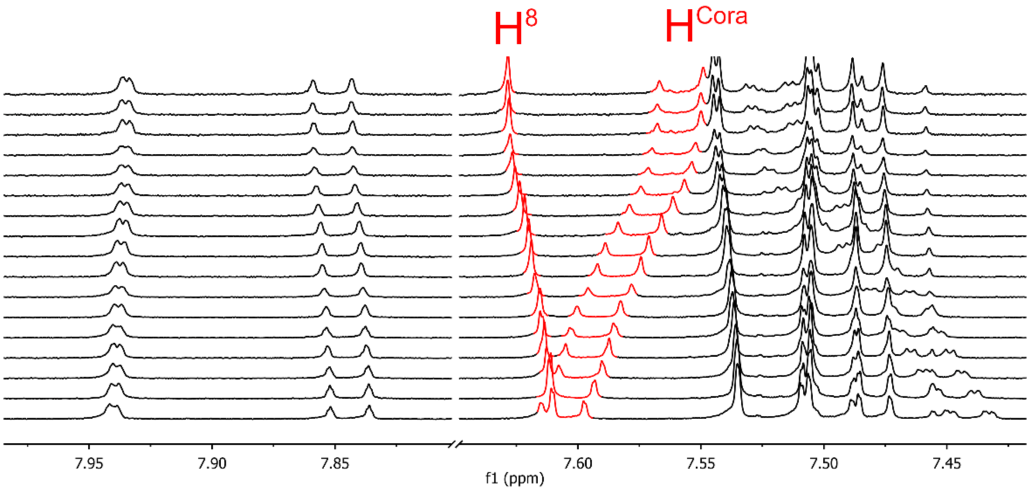

Figure S 141. Stacked ^{1}H-NMR (500 MHz, toluene-d_8) spectra for the titration of **4-SO** with variable concentrations of $C_{60.}$ in toluene-d_8 at 298 K.

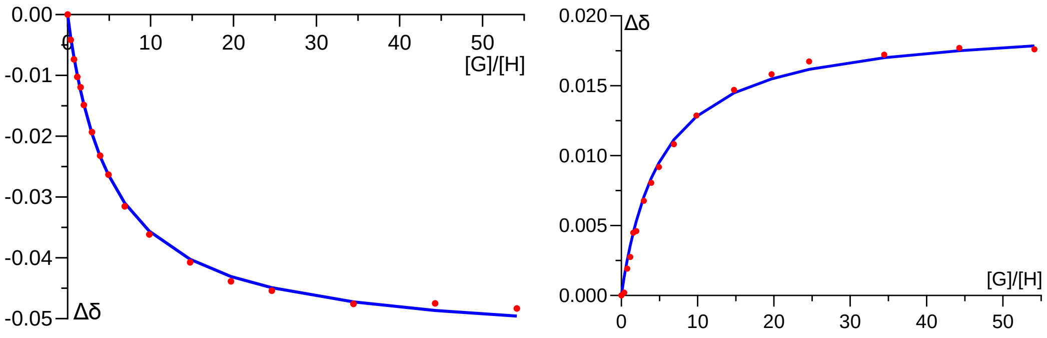

Figure S 142. Nonlinear regressions for selected protons (left plot: H^{8}, right plot: H^{Cora}) for the titration of **4-SO** with C_{60}.

4-SO vs C_{70} (1 to 1)

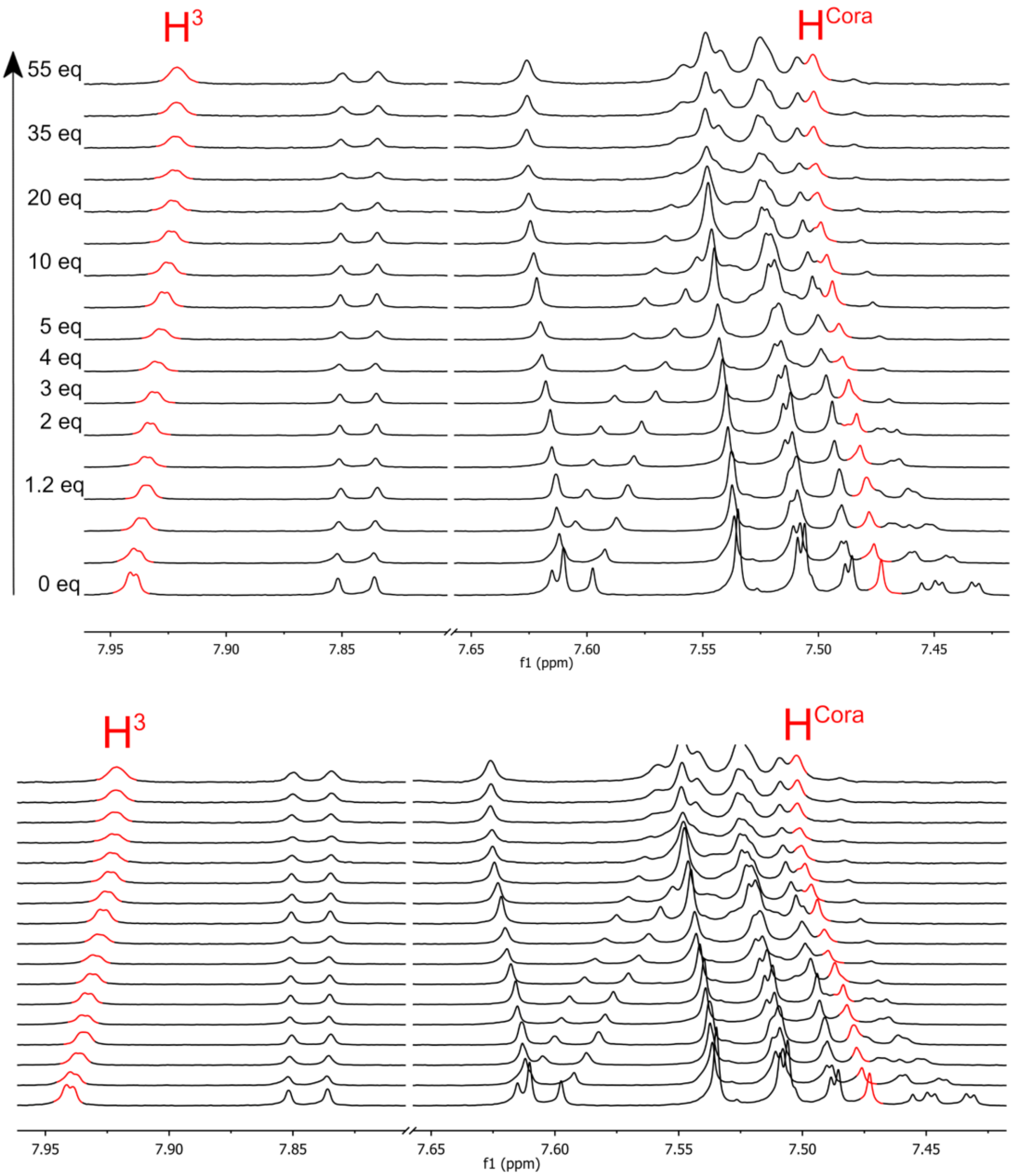

Figure S 143. Stacked ^{1}H-NMR (500 MHz, toluene-d_8) spectra for the titration of **4-SO** with variable concentrations of $C_{60.}$ in toluene-d_8 at 298 K.

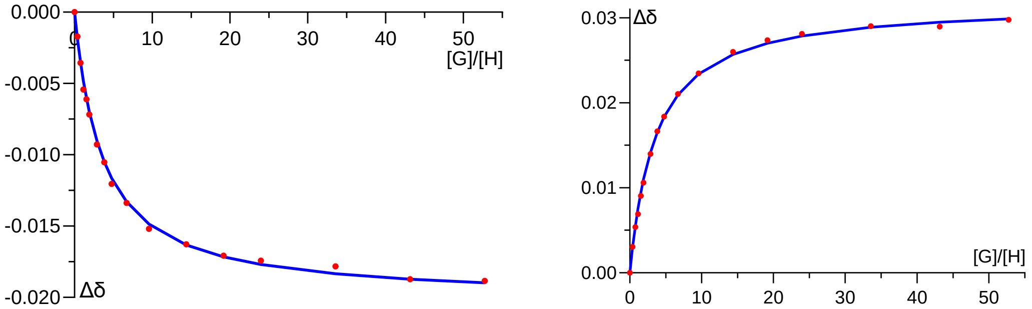

Figure S 144. Nonlinear regressions for selected protons (left plot: H^3, right plot: H^{15}) for the titration of **4-SO** with C_{70}.

4-SO₂ vs C₆₀

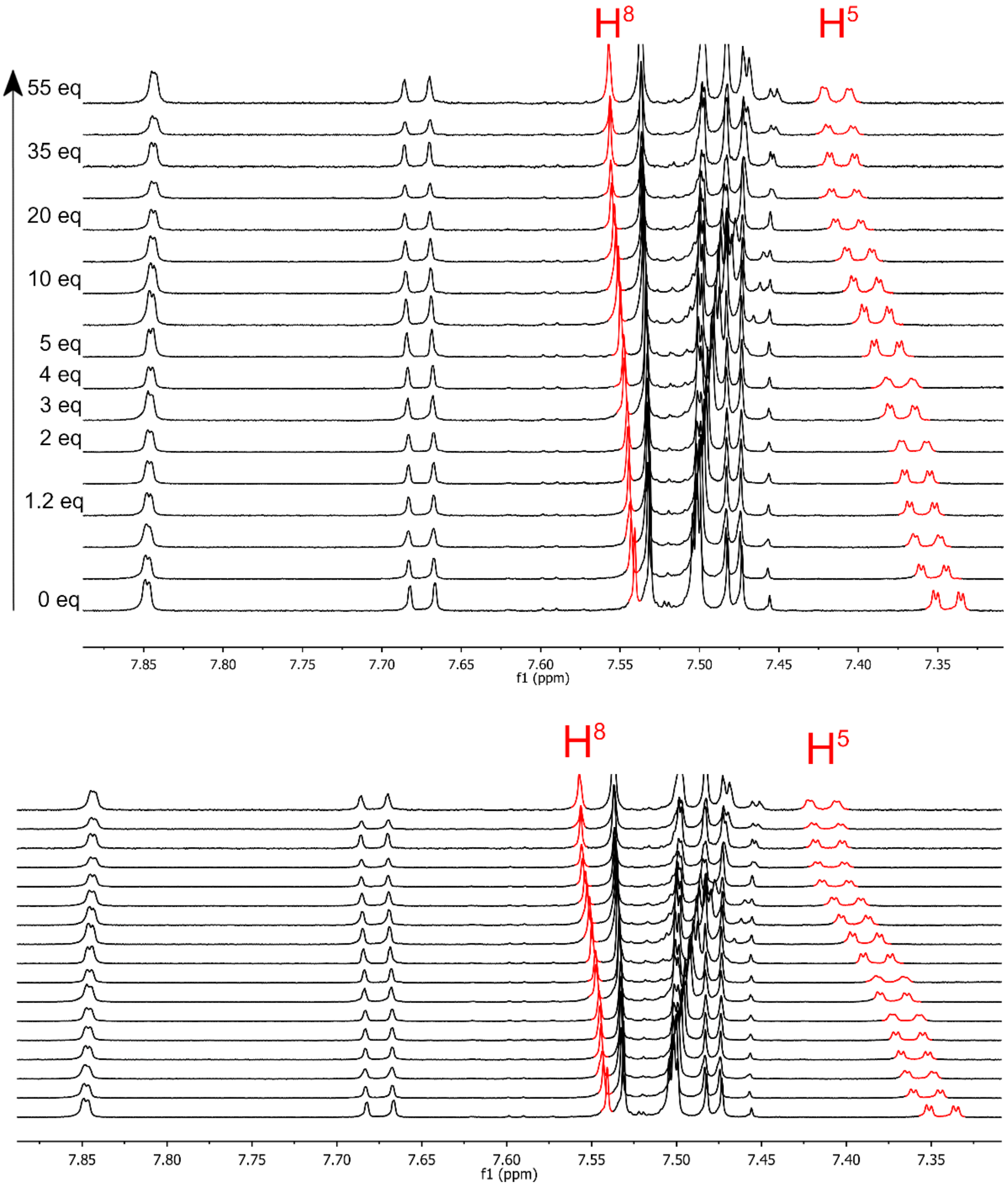

Figure S 145. Stacked ^{1}H-NMR (500 MHz, toluene-d$_8$) spectra for the titration of **4-SO₂** with variable concentrations of C$_{60}$. in toluene-d$_8$ at 298 K.

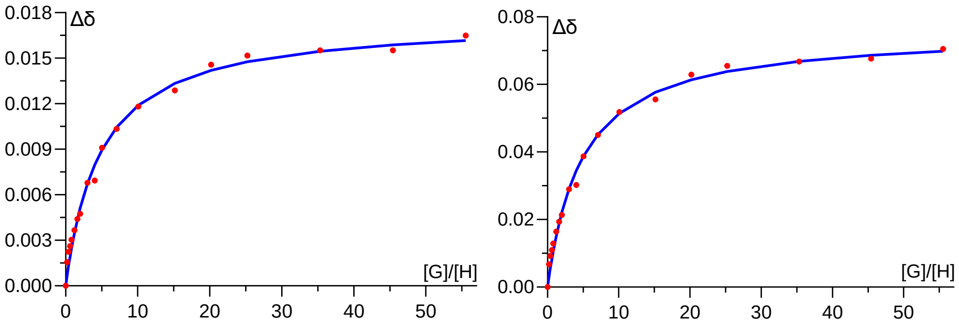

Figure S 146. Nonlinear regressions for selected protons (left plot: H^8, right plot: H^5) for the titration of **4-SO₂** with C$_{60}$.

4-SO₂ vs C_{70} (1 to 1)

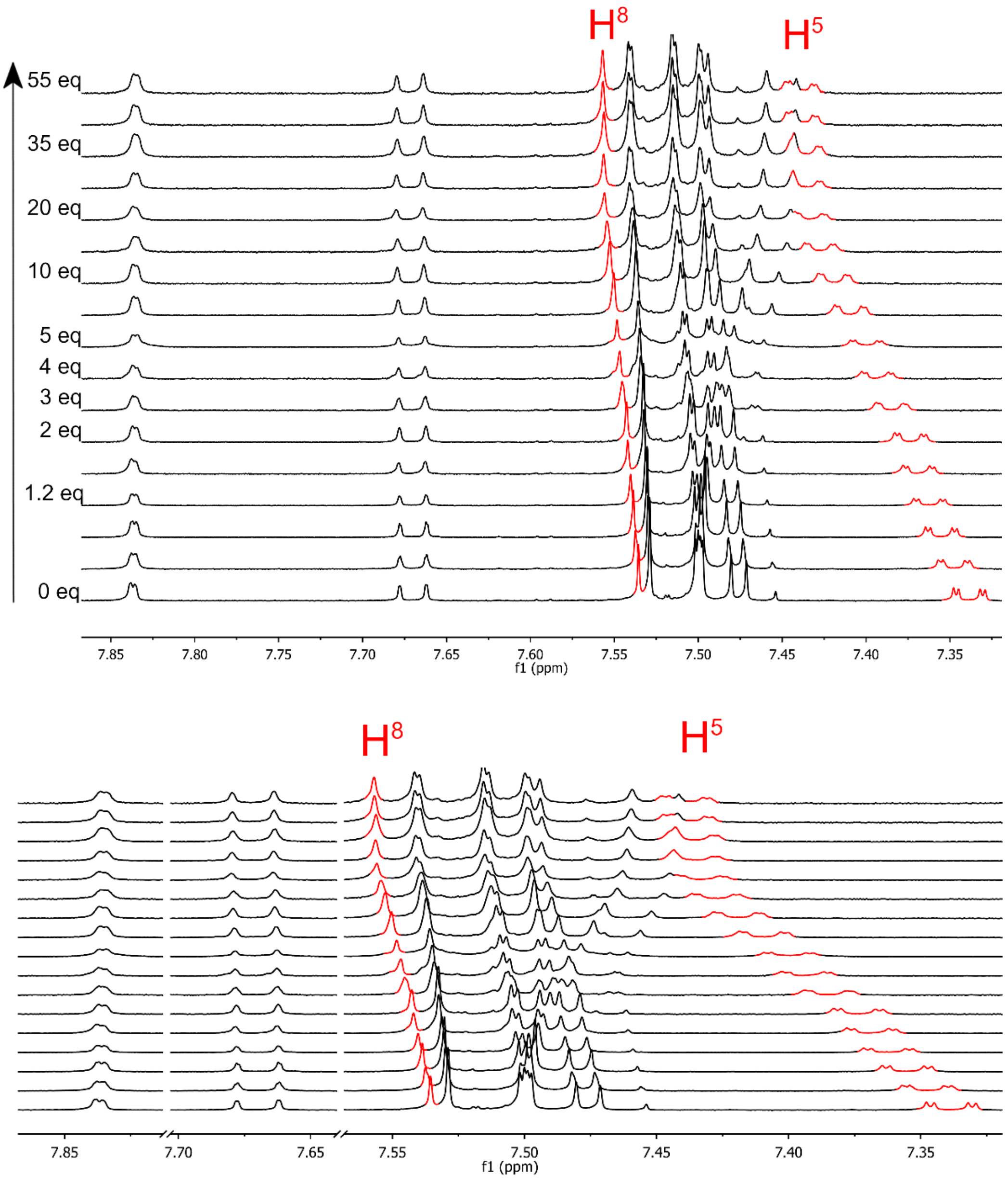

Figure S 147. Stacked ^{1}H-NMR (500 MHz, toluene-d_8) spectra for the titration of **4-SO₂** with variable concentrations of $C_{70.}$ in toluene-d_8 at 298 K.

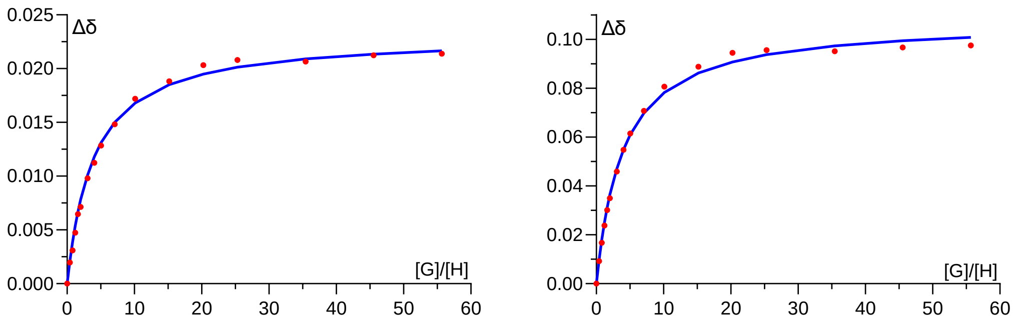

Figure S 148. Nonlinear regressions for selected protons (left plot: H^8, right plot: H^5) for the titration of **4-SO₂** with C_{70}.

11-CMe₂ vs C_{60}

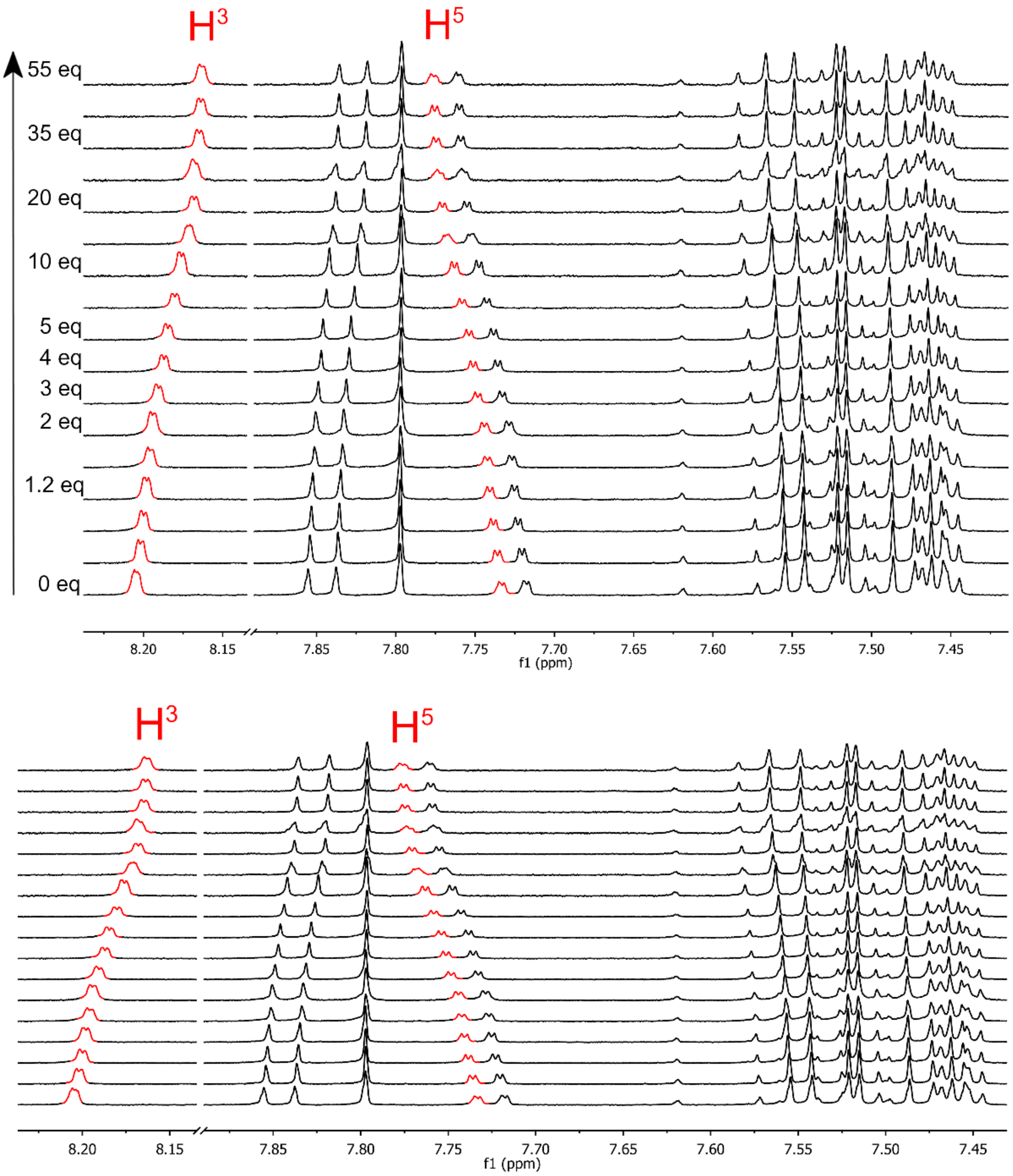

Figure S 149. Stacked ^{1}H-NMR (500 MHz, toluene-d_8) spectra for the titration of **11-CMe₂** with variable concentrations of C_{60}. in toluene-d_8 at 298 K.

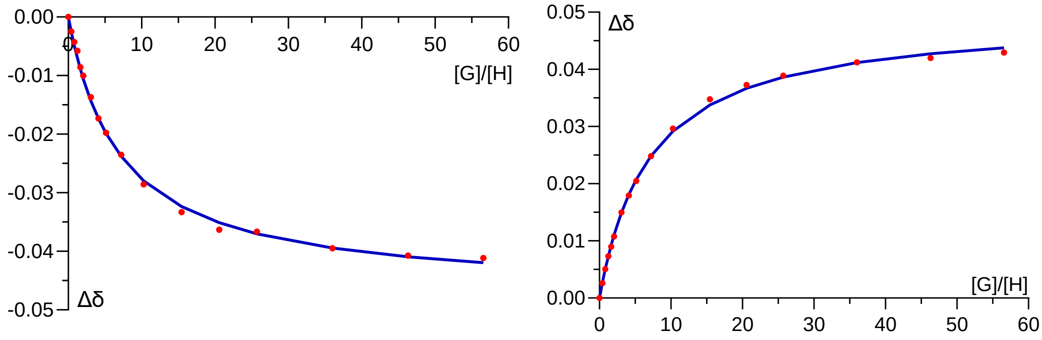

Figure S 150. Nonlinear regressions for selected protons (left plot: H^3, right plot: H^5) for the titration of **11-CMe₂** with C_{60}.

11-CMe₂ vs C_{70} (1 to 1)

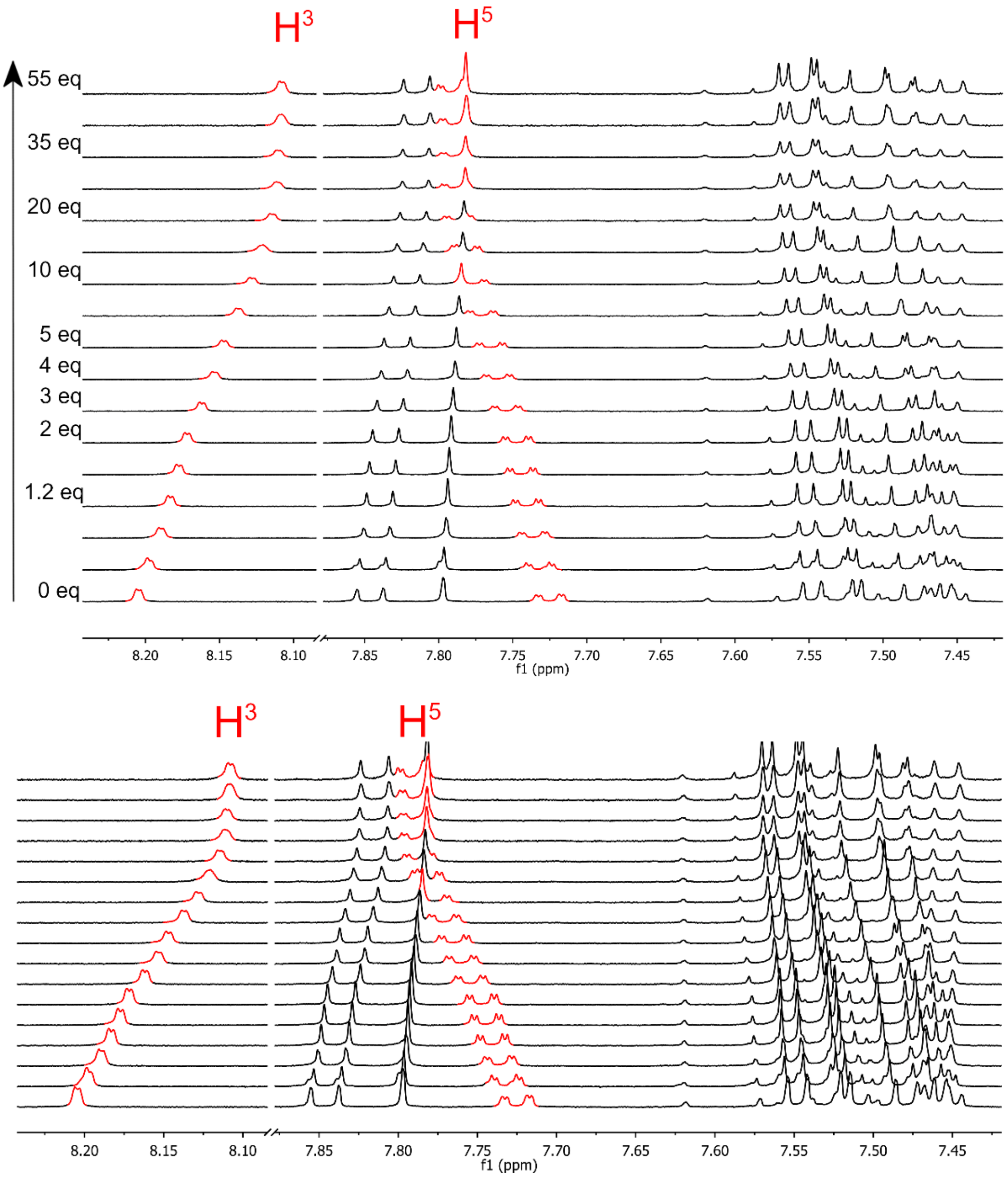

Figure S 151. Stacked ^{1}H-NMR (500 MHz, toluene-d_8) spectra for the titration of **11-CMe₂** with variable concentrations of $C_{70.}$ in toluene-d_8 at 298 K.

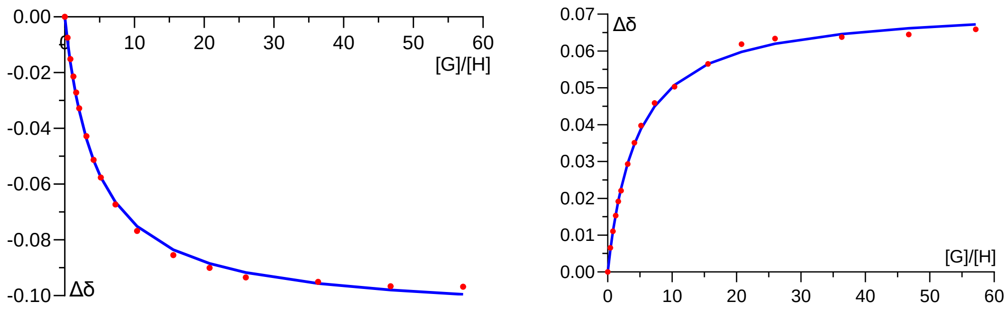

Figure S 152. Nonlinear regressions for selected protons (left plot: H^3, right plot: H^5) for the titration of **11-CMe₂** with C_{70}.

11-CO vs C_{60}

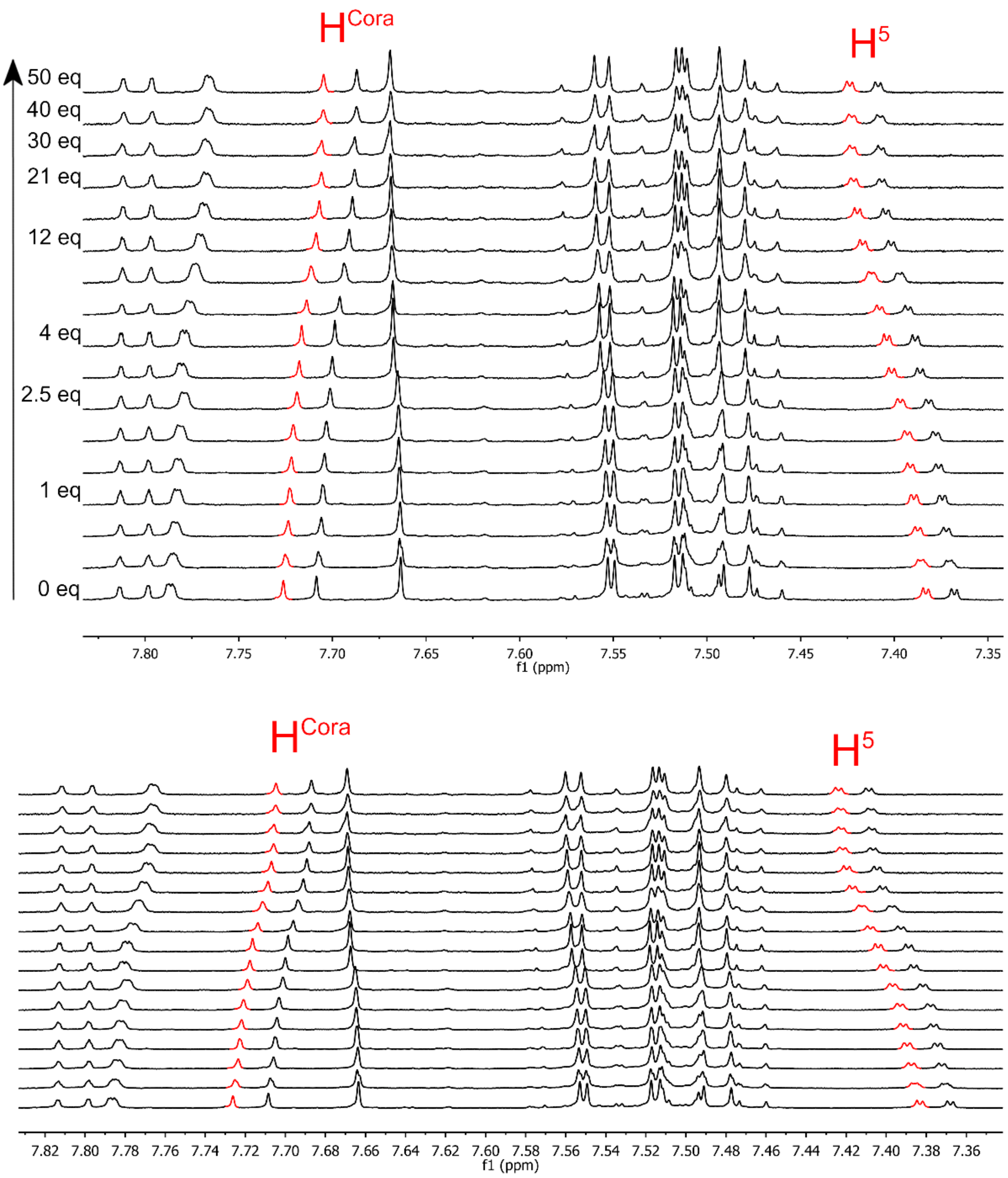

Figure S 153. Stacked ^{1}H-NMR (500 MHz, toluene-d_8) spectra for the titration of **11-CO** with variable concentrations of $C_{60.}$ in toluene-d_8 at 298 K.

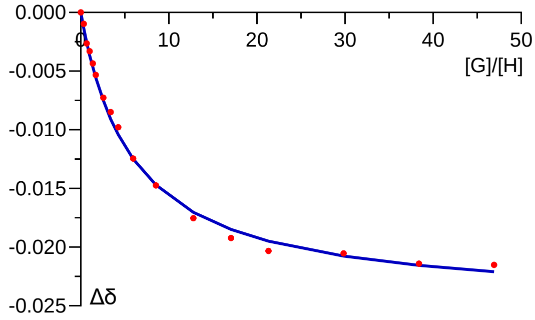

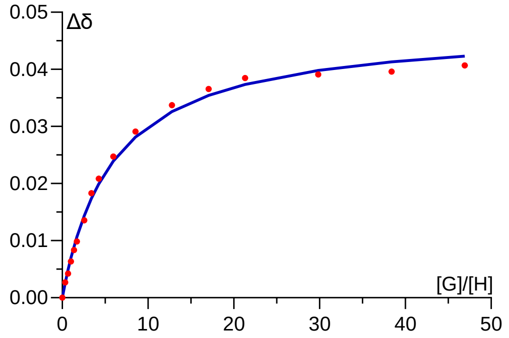

Figure S 154. Nonlinear regressions for selected protons (left plot: H^{Cora}, right plot: H^{5}) for the titration of **11-CO** with C_{60}.

11-CO vs C_{70} (1 to 1)

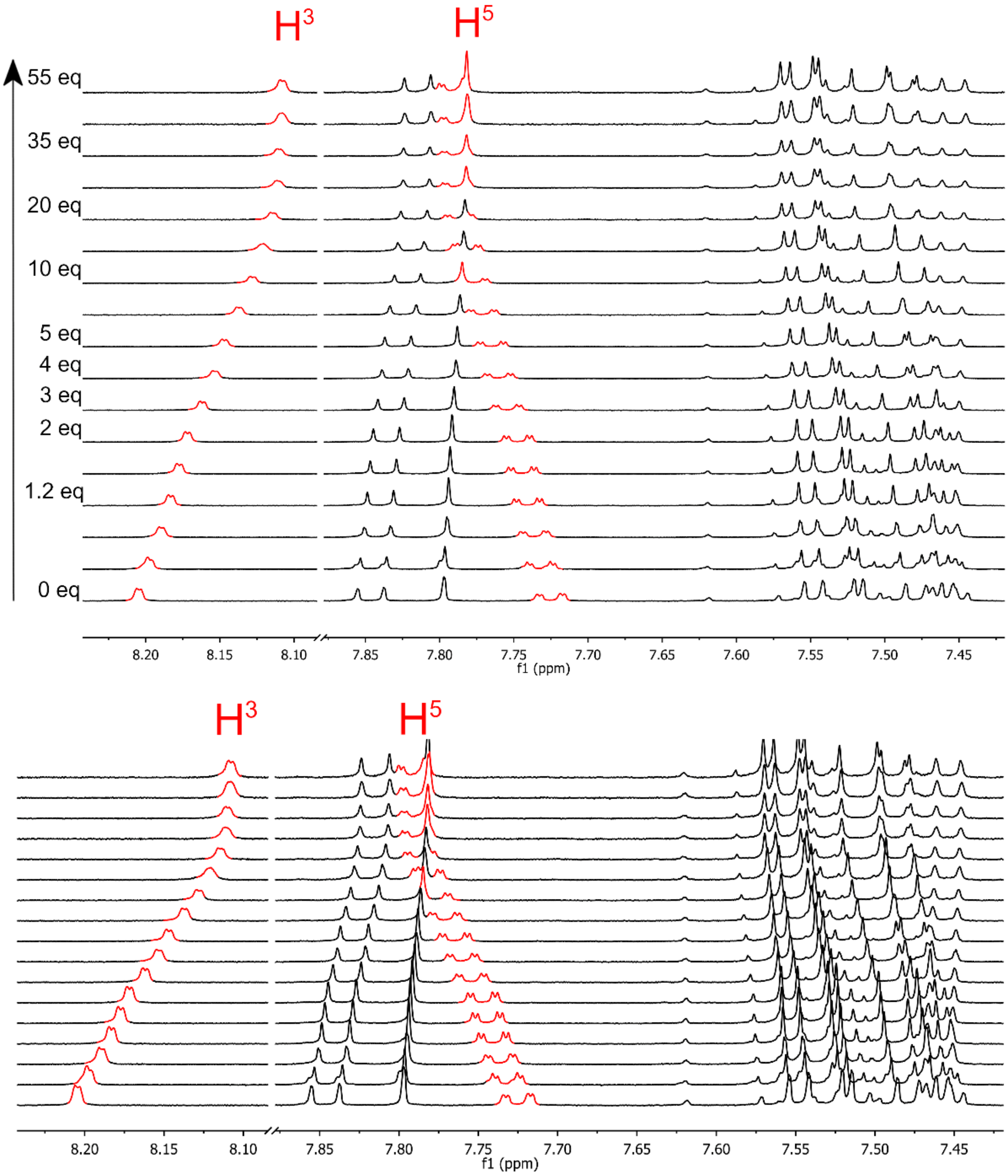

Figure S 155. Stacked ^{1}H-NMR (500 MHz, toluene-d_8) spectra for the titration of **11-CO** with variable concentrations of $C_{70.}$ in toluene-d_8 at 298 K.

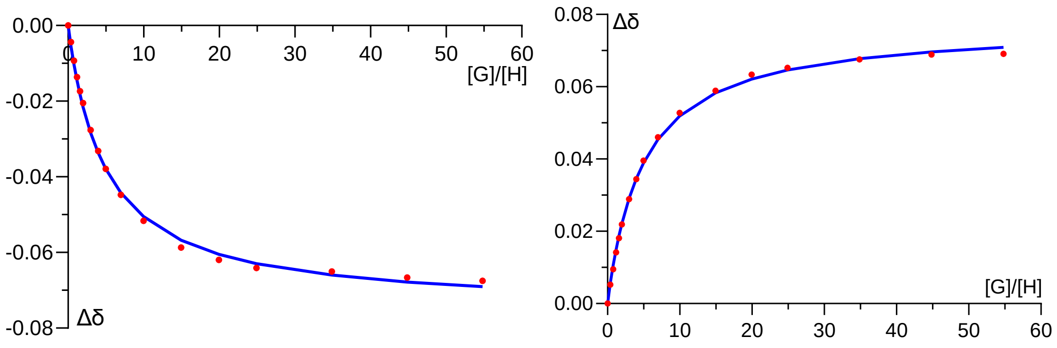

Figure S 156. Nonlinear regressions for selected protons (left plot: H^3, right plot: H^5) for the titration of **11-CO** with C_{70}.

11-C(CN)$_2$ vs C$_{60}$

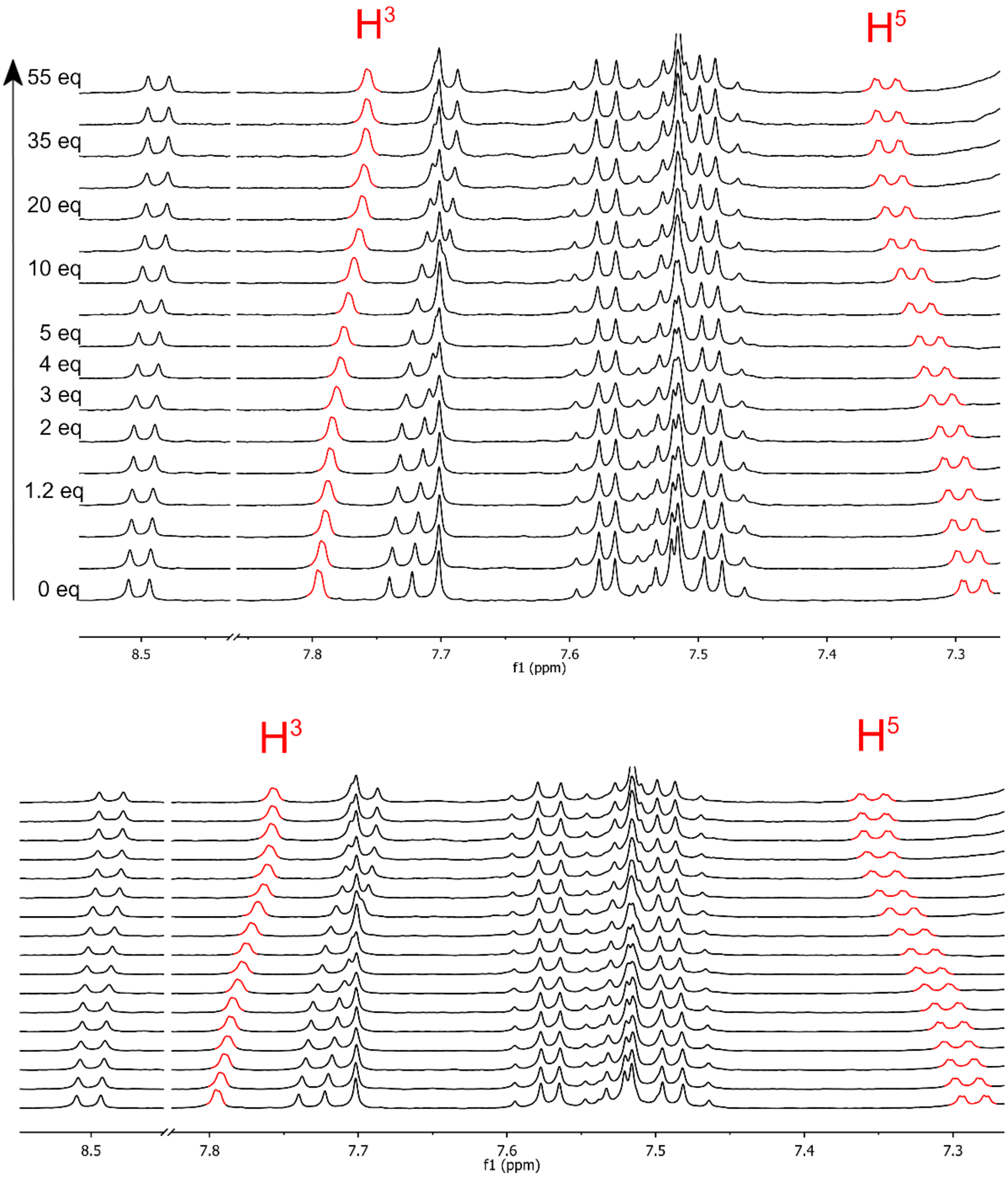

Figure S 157. Stacked ^{1}H-NMR (500 MHz, toluene-d$_8$) spectra for the titration of **11-C(CN)$_2$** with variable concentrations of C$_{60.}$ in toluene-d$_8$ at 298 K.

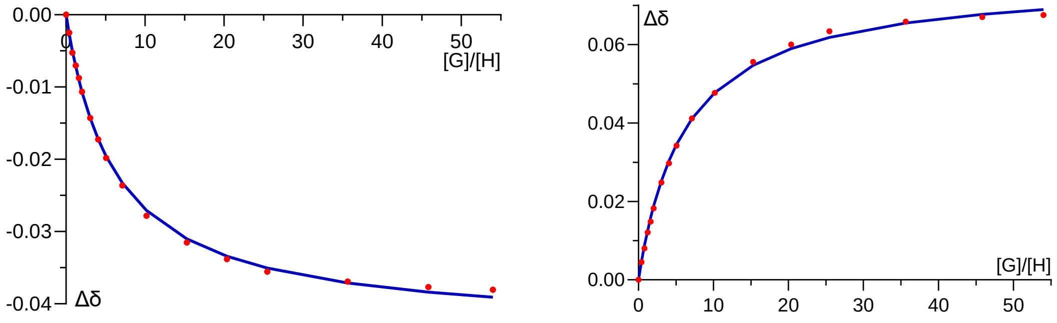

Figure S 158. Nonlinear regressions for selected protons (left plot: H^3, right plot: H^5) for the titration of **11-C(CN)$_2$** with C$_{60}$.

11-C(CN)₂ vs C_{70} (1 to 1)

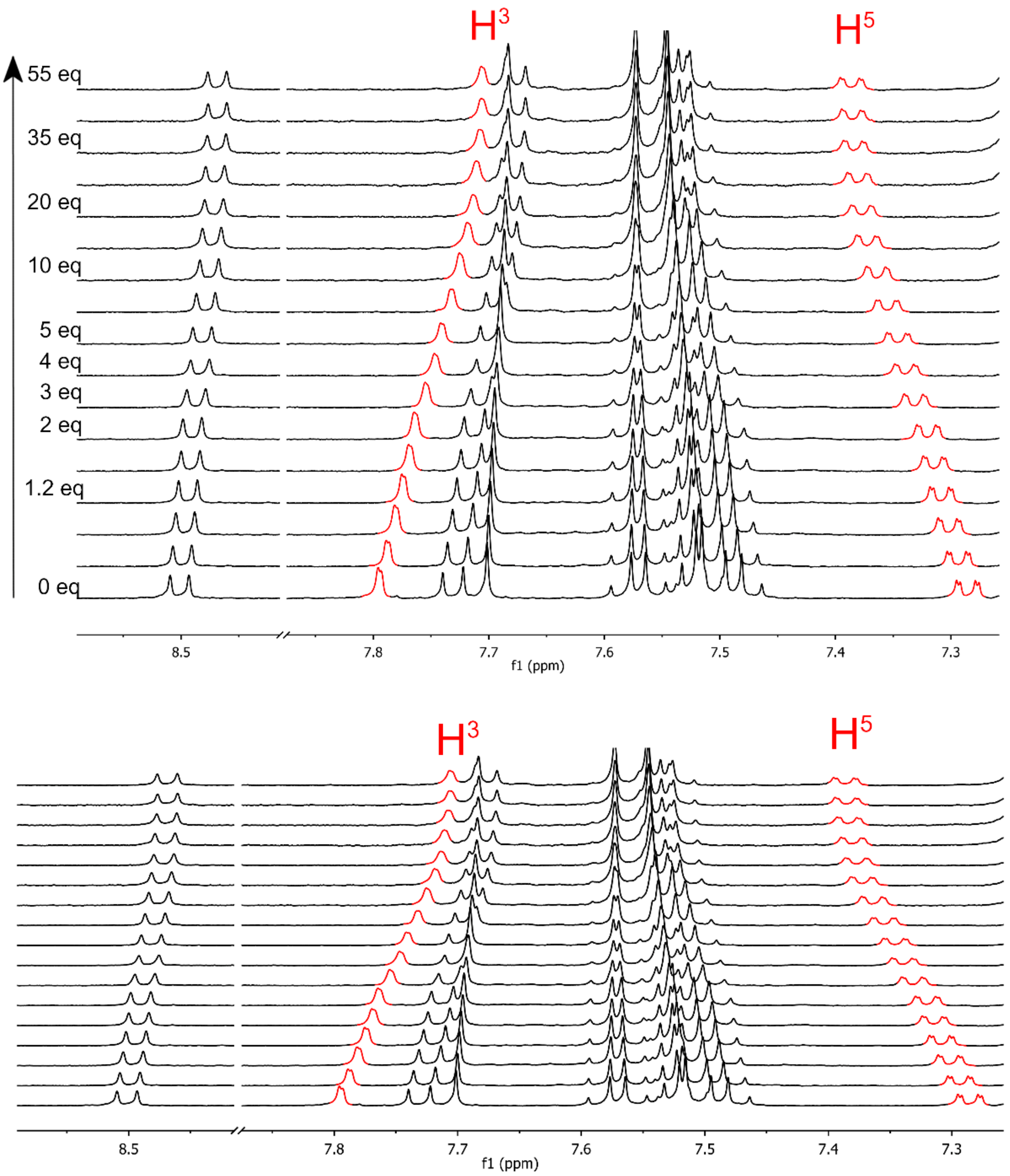

Figure S 159. Stacked ^{1}H-NMR (500 MHz, toluene-d_8) spectra for the titration of **11-C(CN)₂** with variable concentrations of $C_{70.}$ in toluene-d_8 at 298 K.

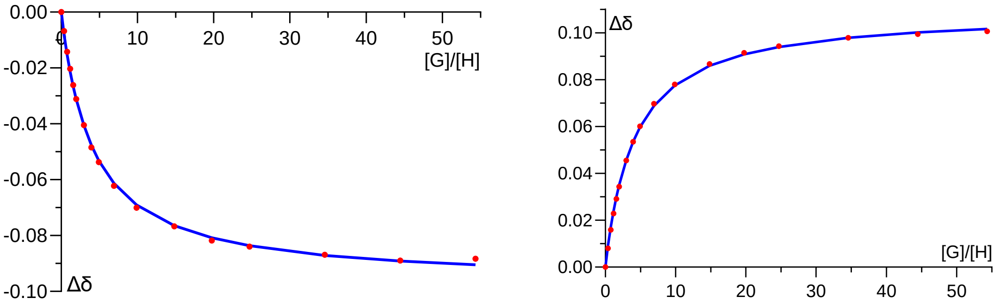

Figure S 160. Nonlinear regressions for selected protons (left plot: H^3, right plot: H^5) for the titration of **11-C(CN)₂** with C_{70}.

16 vs C_{60}

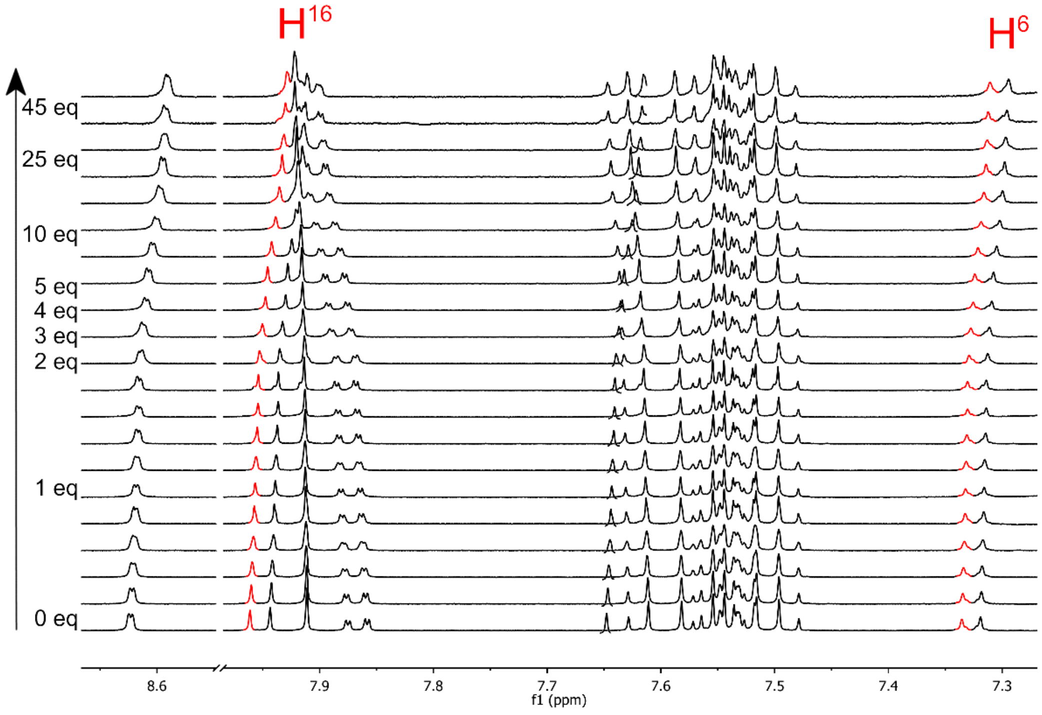

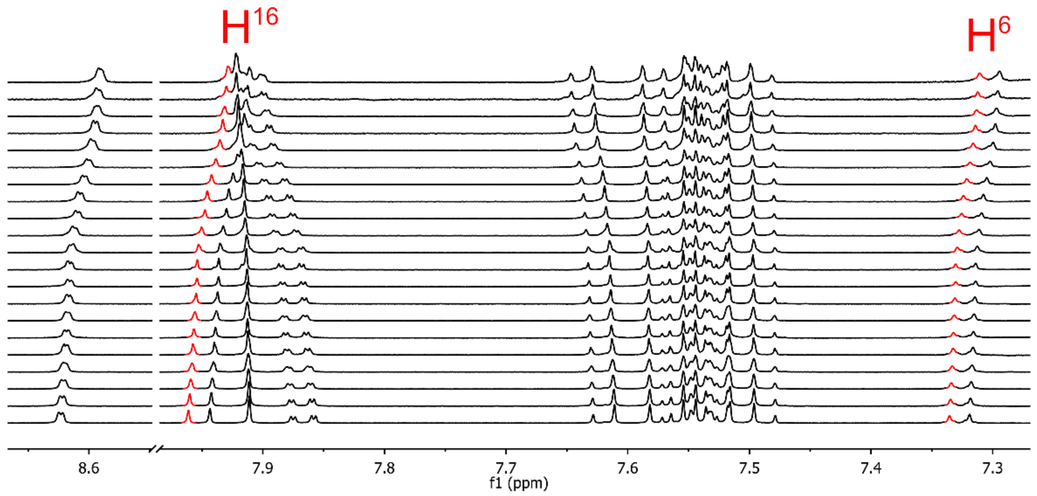

Figure S 161. Stacked ^{1}H-NMR (500 MHz, toluene-d_8) spectra for the titration of **16** with variable concentrations of C_{60}. in toluene-d_8 at 298 K.

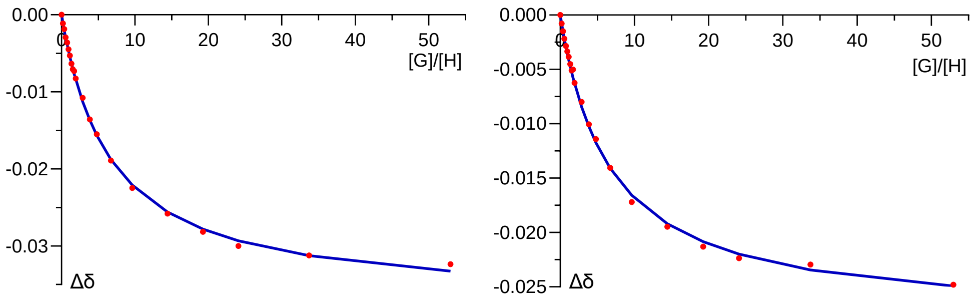

Figure S 162. Nonlinear regressions for selected protons (left plot: H^{16}, right plot: H^{6}) for the titration of **16** with C_{60}.

16 vs C_{70} (1 to 1)

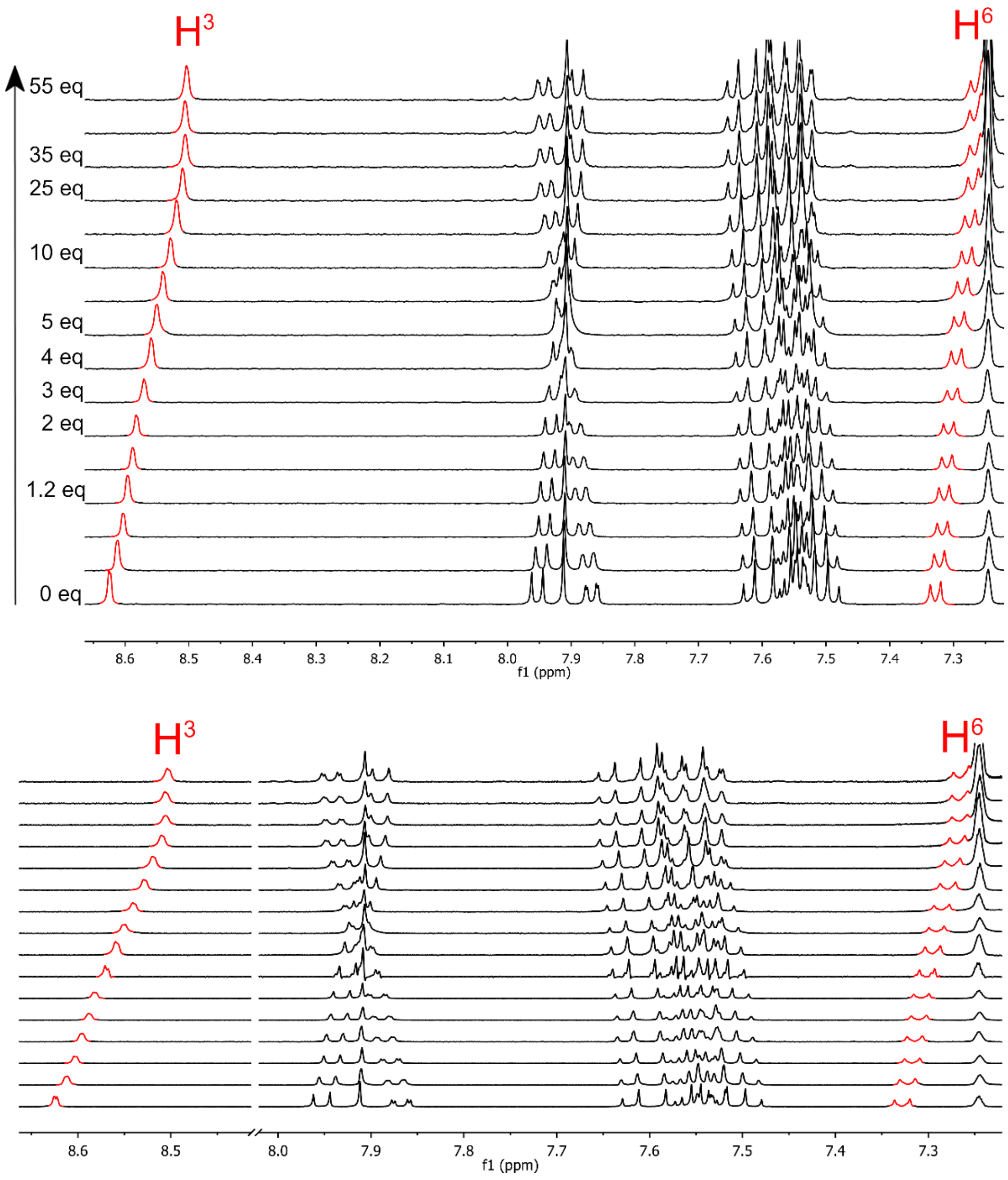

Figure S 163. Stacked ^{1}H-NMR (500 MHz, toluene-d_8) spectra for the titration of **16** with variable concentrations of C_{70}. in toluene-d_8 at 298 K.

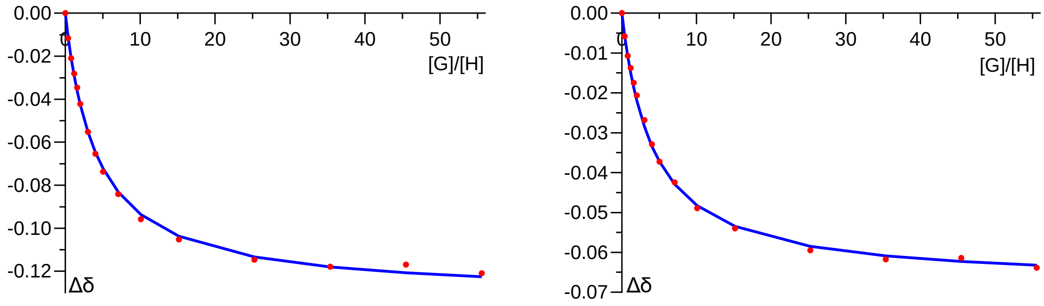

Figure S 164. Nonlinear regressions for selected protons (left plot: H^3, right plot: H^6) for the titration of **16** with C_{70}.

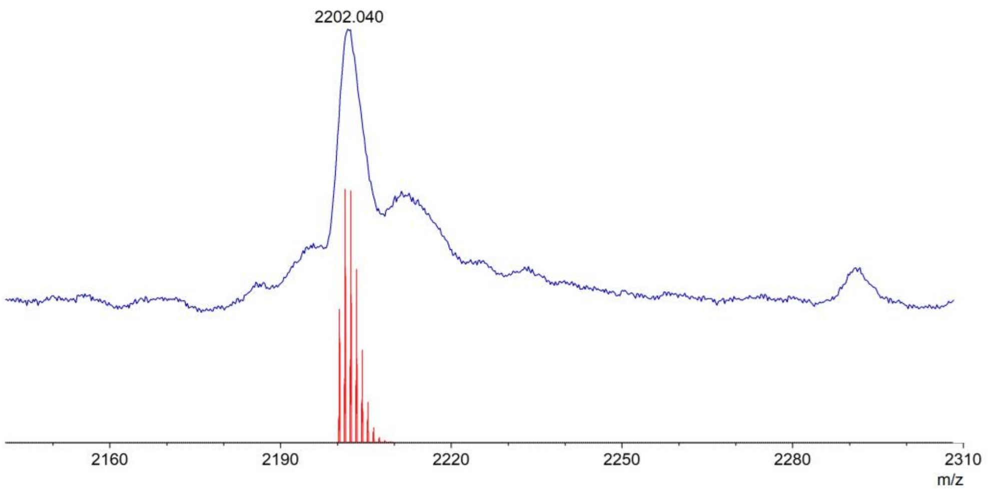

Figure S 165. Detection of adduct of C_{70}@(**4-S**)$_2$ in LRMS (MALDI-TOF, linear detection, positive mode). Calculated (red), measured (blue).

<u>Binding studies of hosts 4-S with guest C_{60} and C_{70} at variable temperature</u>

4-S vs C_{60} - 288 K (1 to 1 model)

http://app.supramolecular.org/bindfit/view/04b1ea52-36bd-4c73-bf4f-1e5e38d6d93f

4-S vs C_{60} - 298 K (1 to 1 model)

http://app.supramolecular.org/bindfit/view/dfc886b8-1d51-4c5f-abad-739d04ca6e00

4-S vs C_{60} - 308 K (1 to 1 model)

http://app.supramolecular.org/bindfit/view/3e9d837f-68c6-49f0-b739-71a1ac243c11

4-S vs C_{70} – 288 K (2 to 1 model, noncooperative flavor)

http://app.supramolecular.org/bindfit/view/ebe22fb8-0233-45f0-9db9-2f20be07d5d2

4-S vs C_{70} – 298 K (2 to 1 model, noncooperative flavor)

http://app.supramolecular.org/bindfit/view/ecd1e311-0d0f-491b-adda-10068b7d6462

4-S vs C_{70} – 308 K (2 to 1 model, noncooperative flavor)

http://app.supramolecular.org/bindfit/view/0b345801-b67a-4810-94ff-b1ab54a43312

The relationship between the association constants and free Gibbs energy is given by:

$$\Delta G = -\mathrm{RTlnK} \quad \text{eq. 9}$$

Expanding the Gibbs free energy term we obtain:

$$\Delta H - T\Delta S = -\mathrm{RTlnK} \quad \text{eq. 10}$$

Rearrangement of the equation provides the following linearized expression:

$$\mathrm{lnK} = -\frac{\Delta H}{RT} + \frac{\Delta S}{R} \quad \text{eq. 11}$$

The plot of *ln K* versus *1/T* (Van't Hoff plot) allows us to estimate the enthalpy and entropy of the complex formation assuming that both magnitudes are independent of temperature within the experimental range (20 K).

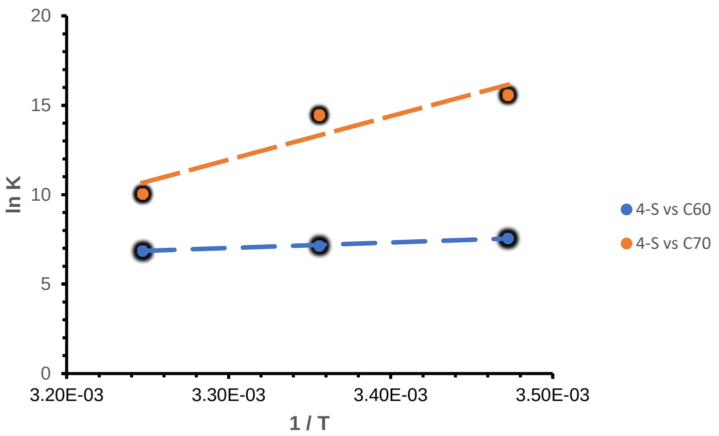

Figure S 166. Van't Hoff plots of assemblies C_{60}@**4-S** and C_{70}@(**4-S**)$_2$ within the range 288 K – 308 K from the association constants obtained in toluene-d_8. For the latter, β constant was used.

Table S 7. Estimated enthalpy and entropy for the formation of supramolecular adducts C_{60}@**4-S** and C_{70}@(**4-S**)$_2$ in toluene-d_8, calculated thermoentropic contribution and final Gibbs free energy.[a]

	ΔH	ΔS	-TΔS	ΔG
**C_{60}@4-S**	-25.7 ± 0.2	-0.027 ± 0.002	7.6 ± 0.2 (288 K)	-18.1 ± 0.3 (288 K)
			7.9 ± 0.2 (298 K)	-17.8 ± 0.3 (298 K)
			8.1 ± 0.2 (308 K)	-17.6 ± 0.3 (308 K)
**C_{70}@(4-S)$_2$**	--203 ± 2	-0.57 ± 0.08	164 ± 1 (288 K)	-39 ± 3 (288 K)
			170 ± 1 (298 K)	-33 ± 3 (298 K)
			175 ± 1 (308 K)	-27 ± 3 (308 K)

[a] in kJ/mol

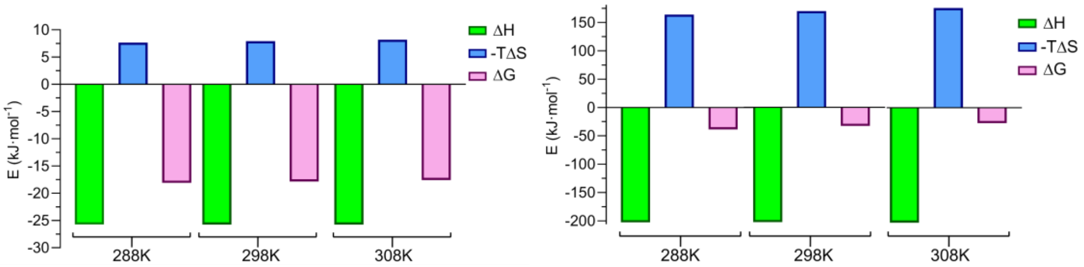

Figure S 167. Bar graph showing the contributions of enthalpy and entropy for the formation of assemblies C_{60}@**4-S** (left) and C_{70}@(**4-S**)$_2$ (right) in toluene-d_8 at three different temperatures.

Quenching Experiments upon fullerene binding

In order to observe the emission quenching of the host with the addition of C_{70} and the formation of a host-guest (HG) complex, the following procedure was carried out: a solution of each compound (range of concentration 10^{-5} - 10^{-6} M) in toluene was prepared. A known volume was transferred to a quartz fluorescence cuvette. The fluorescence was recorded and known portions of a stock solution of C_{70} (range of concentration $3 \cdot 10^{-4}$ - $9 \cdot 10^{-4}$ M) at constant concentration of host in toluene were added to cover a wide range of equivalents. Fluorescence was recorded after each addition. Pictures of the cuvettes under UV-Vis light (365 nm) were taken at the beginning and at the end of the experiments to note the drop of the fluorescence emission intensity. Normalized fluorescence emission spectra show below the decrease in fluorescence with the addition of C_{70} to the sample (equivalents of C_{70} added are shown in the graphics).

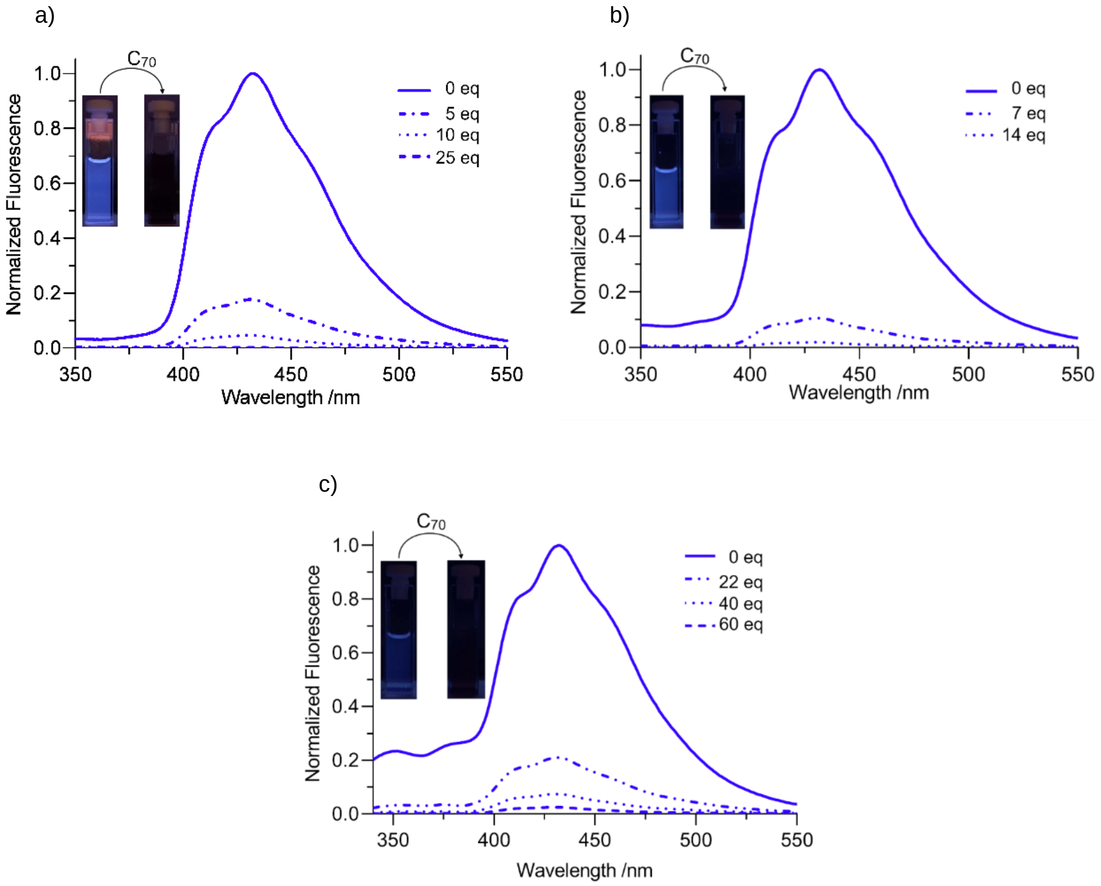

Figure S 168. Normalized quenching emission spectra in toluene of: (a) **4-S** ($5 \cdot 10^{-6}$ M) (λ_{exc} = 301 nm), (b) **4-SO** ($5 \cdot 10^{-6}$ M) (λ_{exc} = 299 nm) and (c) **4-SO₂** (10^{-6} M) (λ_{exc} = 299 nm).

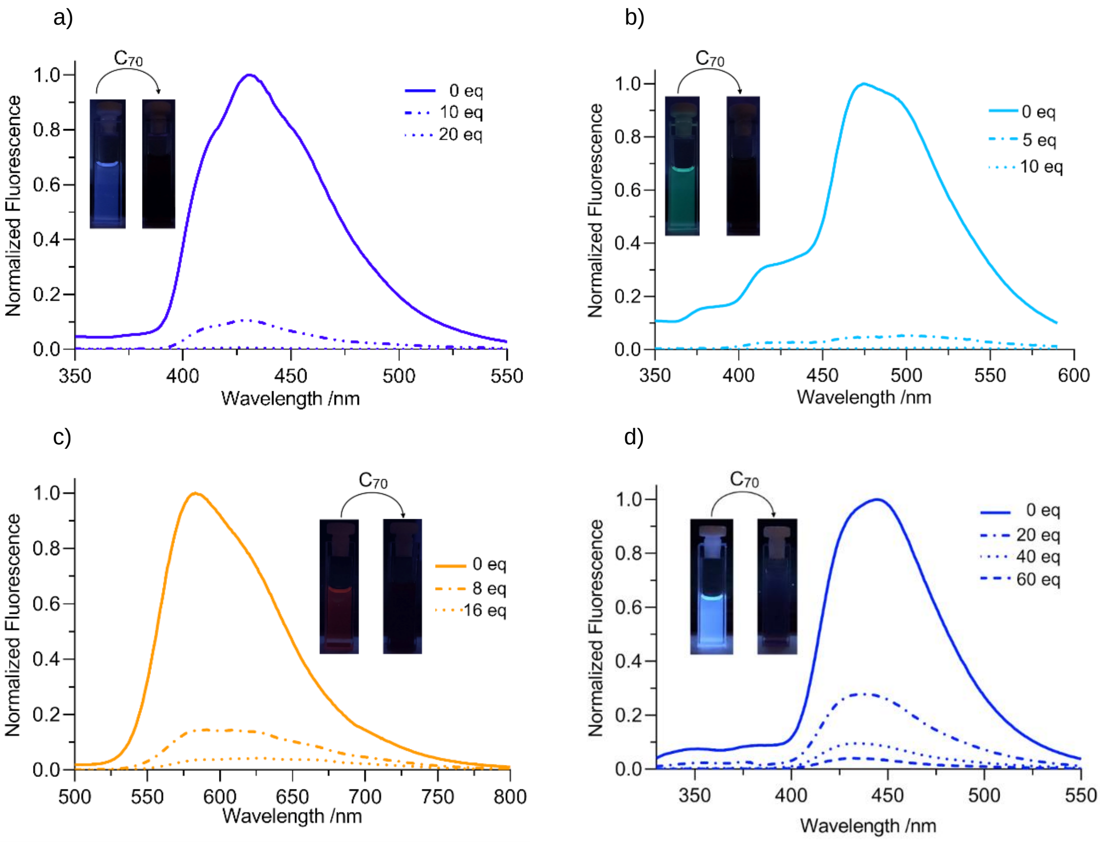

Figure S 169. Normalized quenching emission in toluene of: (a) **11-CMe$_2$** (5·10^{-6} M) (λ_{exc} = 297 nm), (b) **11-CO** (10^{-5} M) (λ_{exc} = 310 nm), (c) **11-C(CN)$_2$** (10^{-5} M) (λ_{exc} = 426 nm) and (d) **16** (10^{-6} M) (λ_{exc} = 302 nm).

Computational Calculations details

Generally, calculations were carried out by DFT methods with Grimme's B97D3 functional containing the Becke-Johnson damping empirical dispersion correction.[16-18] Pople and collaborators' split valence basis set 6-31G(d,p) was chosen.[4] The impact of the solvent was taken into account via the implicit Polarizable Continuum Model (PCM)[21] with toluene (ε=2.37) as the solvent of choice. Gaussian 16 Rev C.01 package was used.[22] All geometries were minimized with no restrictions and the minima were confirmed by vibrational analysis.

Geometries of hosts **4-S**, **4-SO**, **4-SO₂**, **11-CMe₂, 11-CO**, **11-C(CN)₂** and **16** were optimized after several runs with different starting structures to have a variety of conformers. Their relative electronic energy was not higher than ca. 1.3 kcal/mol in any case and the best candidate, based on the lowest energy, was chosen and shown below.

Compound **4-S** (G = -2392.427777 a.u.)

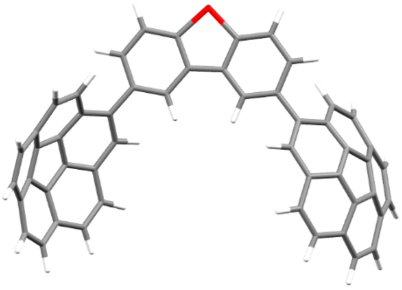

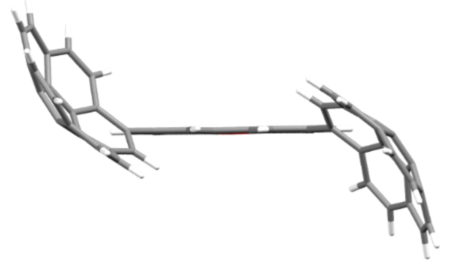

C	0.706032	3.060499	0.176797	C	7.450244	0.108210	-1.247992	C	-7.312021	-1.858210	-0.244908
S	0.000089	5.618930	-0.000099	C	5.852910	-3.588386	1.247341	C	-5.161423	1.019296	-0.346857
C	-1.224591	4.378365	-0.301702	C	7.882527	-2.731454	-0.681977	C	-4.170422	-1.276200	-1.872079
C	1.563884	1.968051	0.381391	C	3.825566	1.025031	0.948167	C	-7.450228	0.108565	1.248029
C	2.914360	2.170958	0.711702	C	5.640377	1.756383	-0.807021	C	-5.853112	-3.588509	-1.247137
C	3.400492	3.498858	0.824114	C	3.370843	-0.088873	1.674027	C	-7.882745	-2.731236	0.682169
C	2.569757	4.602214	0.622734	C	3.744968	-2.632117	2.160033	C	-3.825346	1.024886	-0.948219
C	1.224772	4.378334	0.301607	C	6.731910	1.325095	-1.563113	C	-5.640331	1.756696	0.806772
H	1.188421	0.953296	0.257528	C	8.245488	-0.734652	-2.119770	C	-3.370738	-0.089051	-1.674132
H	4.443078	3.656519	1.095338	C	4.547313	-3.734257	1.861152	C	-3.745173	-2.632405	-2.160139
H	2.960633	5.613429	0.722471	C	6.599578	-4.539106	0.448044	C	-6.731954	1.325556	1.562872
C	-2.914119	2.170871	-0.711943	C	8.451411	-2.088125	-1.850171	C	-8.245776	-0.734177	2.119678
C	-1.563596	1.968018	-0.381721	C	7.567702	-4.130623	-0.471336	C	-4.547625	-3.734466	-1.861172
C	-0.705811	3.060493	-0.177096	H	5.073632	2.616245	-1.161166	C	-6.600079	-4.539092	-0.447941
C	-2.569617	4.602136	-0.622641	H	2.345667	-0.069680	2.045295	C	-8.451793	-2.087662	1.850173
C	-3.400318	3.498739	-0.824064	H	2.732414	-2.807242	2.525905	C	-7.568177	-4.130455	0.471419
H	-1.188083	0.953276	-0.257945	H	6.976525	1.867972	-2.477119	H	-5.073727	2.616718	1.160759
H	-2.960656	5.613304	-0.722215	H	8.609935	-0.329679	-3.064770	H	-2.345623	-0.069805	-2.045558
H	-4.442946	3.656443	-1.095096	H	4.133821	-4.733589	2.003765	H	-2.732748	-2.807752	-2.526263
C	5.984927	-0.034247	0.744072	H	6.332333	-5.595954	0.488217	H	-6.976654	1.868756	2.476664
C	5.506530	-1.150904	1.492548	H	8.970130	-2.694884	-2.593612	H	-8.610485	-0.329118	3.064542
C	7.101246	-0.474478	-0.029122	H	8.023165	-4.882513	-1.117206	H	-4.134251	-4.733816	-2.004009
C	6.324901	-2.276206	1.185473	C	-5.984627	-0.034348	-0.743799	H	-6.333158	-5.596020	-0.488155
C	7.312137	-1.858317	0.245226	C	-5.506333	-1.151064	-1.492201	H	-8.970824	-2.694215	2.593566
C	5.161671	1.019280	0.346893	C	-7.100996	-0.474393	0.029360	H	-8.023858	-4.882348	1.117134
C	4.170525	-1.275986	1.872145	C	-6.324818	-2.276241	-1.185102				

Compound **4-SO** (G = -2467.548586 a.u.)

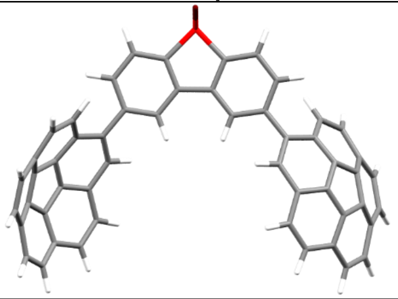

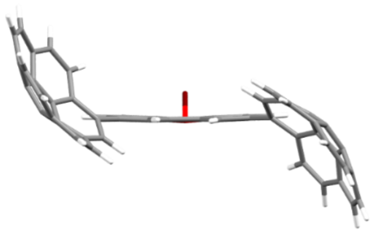

C	0.712347	2.916867	0.304759	C	7.387630	-0.062694	-1.319709	C	-7.311436	-2.005577	-0.301658
S	-0.031179	5.526696	0.240792	C	5.865600	-3.732556	1.260954	C	-5.162474	0.874644	-0.345953
C	-1.232238	4.220577	-0.192288	C	7.829540	-2.898916	-0.745032	C	-4.110548	-1.434479	-1.812749
C	1.563880	1.823483	0.497180	C	3.845584	0.883056	0.992598	C	-7.509322	-0.026155	1.167637
C	2.930138	2.030882	0.783298	C	5.600732	1.597119	-0.830232	C	-5.813285	-3.742436	-1.232194
C	3.423771	3.353637	0.869354	C	3.410515	-0.223227	1.741548	C	-7.916029	-2.871216	0.610575
C	2.577189	4.456855	0.698976	C	3.793906	-2.762202	2.239248	C	-3.804569	0.873907	-0.895596
C	1.233071	4.223268	0.420889	C	6.663694	1.154775	-1.619684	C	-5.686948	1.622256	0.781126
H	1.187748	0.807216	0.390834	C	8.149191	-0.915581	-2.211547	C	-3.319496	-0.245809	-1.592102
H	4.476922	3.510764	1.095370	C	4.582070	-3.869178	1.921672	C	-3.673075	-2.792302	-2.073465

H	2.963797	5.471895	0.779056	C	6.580944	-4.692694	0.444480	C	-6.805996	1.195408	1.498383
C	-2.903452	2.024093	-0.641113	C	8.359633	-2.267478	-1.937756	C	-8.336712	-0.862345	2.015584
C	-1.559395	1.820168	-0.262238	C	7.517553	-4.295158	-0.511521	C	-4.485448	-3.892397	-1.795390
C	-0.713641	2.915297	-0.054691	H	5.026612	2.456893	-1.172341	C	-6.589298	-4.687238	-0.454012
C	-2.557680	4.450958	-0.550968	H	2.398419	-0.198129	2.146815	C	-8.530276	-2.218469	1.750247
C	-3.386139	3.345697	-0.785891	H	2.794679	-2.930748	2.642701	C	-7.592059	-4.271783	0.424102
H	-1.192867	0.805353	-0.115907	H	6.879061	1.689854	-2.545427	H	-5.137693	2.487778	1.148695
H	-2.937759	5.465106	-0.666573	H	8.481887	-0.519795	-3.171927	H	-2.280969	-0.229033	-1.924123
H	-4.415907	3.499991	-1.103223	H	4.171272	-4.865950	2.087585	H	-2.647801	-2.969286	-2.400694
C	5.991515	-0.183417	0.722194	H	6.312037	-5.748167	0.503134	H	-7.087524	1.747240	2.396174
C	5.535232	-1.292254	1.495929	H	8.850225	-2.882228	-2.693527	H	-8.738384	-0.449510	2.941816
C	7.079204	-0.633988	-0.084721	H	7.947624	-5.054103	-1.166406	H	-4.066561	-4.892487	-1.914437
C	6.339602	-2.422739	1.171468	C	-5.967572	-0.184489	-0.764116	H	-6.320301	-5.744116	-0.475122
C	7.294824	-2.016137	0.194088	C	-5.459500	-1.307105	-1.483652	H	-9.076694	-2.819430	2.478296
C	5.159707	0.870590	0.345367	C	-7.112606	-0.619269	-0.031284	H	-8.071466	-5.018561	1.058373
C	4.213565	-1.410247	1.924515	C	-6.288662	-2.430495	-1.199640	O	0.263229	6.456411	-0.931705

Compound **4-SO₂** (G = -2542.716306 a.u.)

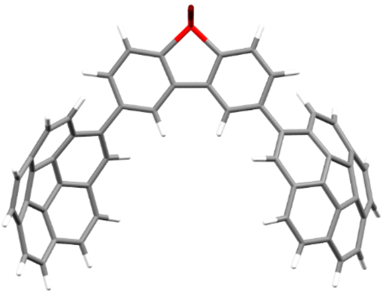

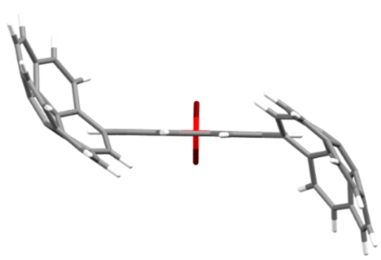

C	-0.716645	2.820083	-0.181424	C	-5.787411	-3.874128	-1.249766	C	5.150445	0.742415	0.349857
S	-0.000007	5.371722	0.000005	C	-7.833850	-3.036892	0.670450	C	4.127091	-1.546230	1.870189
C	1.254550	4.115739	0.312972	C	-3.812378	0.757591	-0.945735	C	7.435128	-0.192405	-1.235461
C	-1.556374	1.720358	-0.380016	C	-5.642724	1.476665	0.800321	C	5.787413	-3.874135	1.249752
C	-2.914461	1.914732	-0.715057	C	-3.340024	-0.351975	-1.666815	C	7.833872	-3.036879	-0.670444
C	-3.415332	3.231984	-0.833404	C	-3.686185	-2.897692	-2.156646	C	3.812366	0.757588	0.945729
C	-2.588666	4.344929	-0.631761	C	-6.732539	1.033391	1.551734	C	5.642731	1.476680	-0.800310
C	-1.254562	4.115740	-0.312971	C	-8.225053	-1.042798	2.104741	C	3.340009	-0.351983	1.666801
H	-1.177367	0.708254	-0.251310	C	-4.478426	-4.007348	-1.859414	C	3.686178	-2.897708	2.156626
H	-4.458669	3.378206	-1.106121	C	-6.527406	-4.832183	-0.452967	C	6.732556	1.033415	-1.551714
H	-2.977144	5.356770	-0.730649	C	-8.414753	-2.398675	1.835573	C	8.225090	-1.042771	-2.104715
C	2.914448	1.914729	0.715054	C	-7.503237	-4.432856	0.462165	C	4.478428	-4.007360	1.859398
C	1.556361	1.720356	0.380016	H	-5.089733	2.344466	1.156759	C	6.527432	-4.832182	0.452964
C	0.716632	2.820082	0.181426	H	-2.313839	-0.322510	-2.034565	C	8.414795	-2.398649	-1.835551
C	2.588654	4.344926	0.631759	H	-2.671322	-3.062465	-2.520543	C	7.503271	-4.432845	-0.462156
C	3.415320	3.231981	0.833400	H	-6.988307	1.575708	2.462852	H	5.089749	2.344489	-1.156744
H	1.177353	0.708253	0.251311	H	-8.598486	-0.640721	3.047311	H	2.313823	-0.322517	2.034547
H	2.977134	5.356767	0.730644	H	-4.054865	-5.002530	-2.000702	H	2.671317	-3.062491	2.520524
H	4.458658	3.378203	1.106113	H	-6.249409	-5.886191	-0.491527	H	6.988335	1.575748	-2.462819
C	-5.958908	-0.321501	-0.748582	H	-8.930218	-3.010299	2.577132	H	8.598545	-0.640689	-3.047274
C	-5.464902	-1.433896	-1.493514	H	-7.953438	-5.188930	1.106719	H	4.054871	-5.002543	2.000691
C	-7.074278	-0.772657	0.019062	C	5.958892	-0.321504	0.748572	H	6.249452	-5.886194	0.491524
C	-6.273724	-2.567144	-1.190771	C	5.464883	-1.433904	1.493493	H	8.930283	-3.010261	-2.577103
C	-7.269149	-2.158744	-0.255246	C	7.074271	-0.772651	-0.019062	H	7.953490	-5.188920	-1.106698
C	-5.150456	0.742412	-0.349860	C	6.273713	-2.567145	1.190754	O	0.310456	6.128968	-1.236882
C	-4.127109	-1.546218	-1.870209	C	7.269144	-2.158737	0.255241	O	-0.310476	6.128953	1.236901
C	-7.435114	-0.192425	1.235474								

Compound **11-CMe₂** conformer 2 (G = -2112.024433 a.u.)

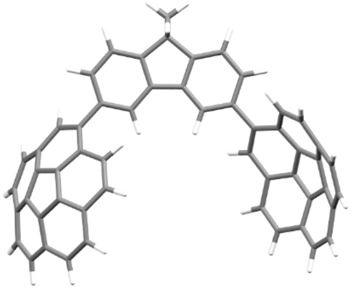

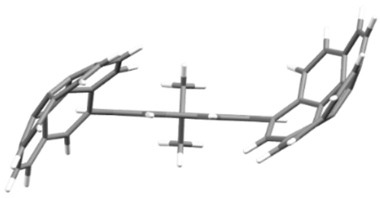

C	0.953833	3.106010	0.047835	C	4.319820	1.382753	-0.298224	C	-5.278566	1.527699	-1.018167
C	0.087055	5.370226	-0.036556	C	3.000070	-0.322138	-1.828074	C	-3.624866	1.000502	0.955336
C	-1.020226	4.350938	0.236735	C	5.410788	1.490002	0.582115	C	-6.286047	1.003876	-1.830377
C	1.917409	2.093396	-0.001818	C	7.290495	0.162649	1.828117	C	-7.704246	-1.135070	-2.337342
C	3.264943	2.430374	-0.249454	C	2.906932	-1.662692	-2.206605	C	-3.221796	-0.034137	1.816435
C	3.613448	3.788324	-0.424073	C	3.946563	-4.045066	-1.843721	C	-3.597233	-2.532607	2.493779
C	2.645787	4.803920	-0.368393	C	7.845071	-1.105596	2.008525	C	-7.911654	-2.464358	-1.967214

C	1.314261	4.462473	-0.138027	C	7.654273	-3.624529	1.306133	C	-7.127739	-4.354124	-0.333998
H	1.643182	1.055470	0.179121	C	4.945086	-4.752936	-1.171573	C	-4.348839	-3.676906	2.222867
H	4.655080	4.038890	-0.621901	C	6.935838	-4.527230	0.519241	C	-6.243989	-4.655060	0.704181
H	2.940082	5.843999	-0.511885	H	2.128356	0.314932	-1.967231	H	-6.452341	1.457505	-2.808440
C	-2.712796	2.142342	0.699789	H	5.408172	2.325499	1.283074	H	-7.983965	-0.824434	-3.344905
C	-1.329264	1.938189	0.511728	H	7.434185	0.910415	2.609137	H	-2.234780	0.041311	2.273819
C	-0.494567	3.036835	0.286151	H	1.959937	-2.022601	-2.611123	H	-2.620352	-2.648963	2.964923
C	-2.386485	4.561312	0.416604	H	3.066816	-4.588446	-2.191144	H	-8.346835	-3.147416	-2.698015
C	-3.224867	3.459889	0.646592	H	8.402662	-1.304067	2.924901	H	-7.506913	-5.171362	-0.949062
H	-0.928292	0.925155	0.515688	H	8.195771	-4.006093	2.172843	H	-3.933591	-4.649055	2.492170
H	-2.806694	5.567052	0.387023	H	4.809155	-5.824074	-1.016074	H	-5.962994	-5.696984	0.863761
H	-4.290473	3.615794	0.809035	H	6.942536	-5.581579	0.799058	H	-4.695196	2.367282	-1.393155
C	5.278304	-0.644012	-1.179524	C	-5.737781	-0.127340	0.649401	C	0.226862	6.373956	1.131433
C	6.371961	-0.523435	-0.274155	C	-6.768065	-0.662432	-0.181434	C	-0.162848	6.125134	-1.363004
C	5.184327	-2.013068	-1.572405	C	-5.313530	-1.161076	1.536696	H	0.401977	5.847401	2.077633
C	6.942980	-1.816372	-0.092985	C	-6.980235	-2.021690	0.195617	H	1.068380	7.056385	0.951796
C	6.208666	-2.737374	-0.895792	C	-6.079532	-2.329712	1.257385	H	-0.686451	6.975486	1.231628
C	4.176537	0.211276	-1.164462	C	-4.898474	0.907607	0.236854	H	0.670880	6.806281	-1.579699
C	6.425039	0.468189	0.704200	C	-7.010020	-0.198493	-1.474981	H	-1.083443	6.720425	-1.297913
C	3.986415	-2.601879	-1.975261	C	-4.017023	-1.218255	2.047221	H	-0.262343	5.420873	-2.198118
C	7.593225	-2.193285	1.082284	C	-7.445262	-2.987484	-0.698012				
C	6.084234	-4.088117	-0.569276	C	-5.593022	-3.618913	1.480495				

Compound **11-CO** (G = -2107.480487 a.u.)

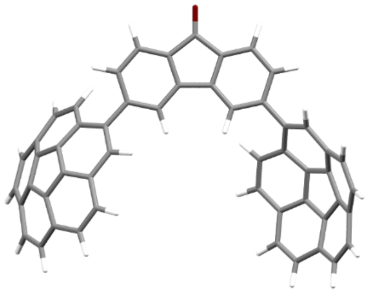

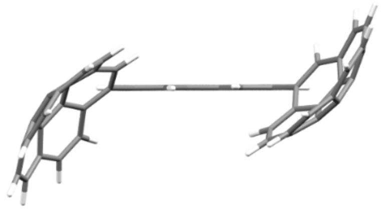

C	0.967596	3.235667	0.038362	C	4.040127	-2.441697	-1.956028	C	-6.125314	-2.147030	1.247811
C	0.093705	5.445532	-0.025834	C	7.657272	-1.972224	1.081290	C	-4.925227	1.085929	0.232109
C	-1.022317	4.476939	0.235701	C	6.157664	-3.895303	-0.546252	C	-7.080035	0.017962	-1.449465
C	1.935483	2.238062	-0.017325	C	4.347724	1.560752	-0.315150	C	-4.034976	-1.068936	2.008775
C	3.285620	2.598094	-0.262561	C	3.033308	-0.169602	-1.826636	C	-7.534566	-2.771557	-0.686984
C	3.625599	3.959434	-0.426349	C	5.443555	1.687899	0.557765	C	-5.650585	-3.443742	1.451780
C	2.648964	4.965906	-0.364515	C	7.339703	0.388054	1.807437	C	-5.320758	1.720859	-1.010775
C	1.325771	4.595348	-0.139078	C	2.951739	-1.514249	-2.192932	C	-3.637710	1.154877	0.929427
H	1.678816	1.194868	0.156166	C	4.014440	-3.883994	-1.812134	C	-6.348886	1.214886	-1.808200
H	4.665306	4.217305	-0.622296	C	7.905590	-0.873812	1.995995	C	-7.801711	-0.902790	-2.306319
H	2.910938	6.015118	-0.496835	C	7.732838	-3.400798	1.317358	C	-3.229155	0.105826	1.771696
C	-2.720630	2.291645	0.681299	C	5.022369	-4.576241	-1.137819	C	-3.623472	-2.391578	2.438384
C	-1.330951	2.073028	0.494971	C	7.018760	-4.316755	0.541918	C	-8.018152	-2.232448	-1.943045
C	-0.494221	3.161982	0.277894	H	2.156333	0.457813	-1.975608	C	-7.227402	-4.144936	-0.339478
C	-2.384291	4.708982	0.410890	H	5.438636	2.530697	1.249756	C	-4.394332	-3.523727	2.171592
C	-3.228697	3.610906	0.633637	H	7.482993	1.145055	2.579376	C	-6.328751	-4.465506	0.679802
H	-0.942317	1.055695	0.494481	H	2.007807	-1.885442	-2.594221	H	-6.528159	1.679267	-2.778753
H	-2.777031	5.724751	0.381166	H	3.138971	-4.438773	-2.151971	H	-8.096834	-0.580196	-3.305562
H	-4.293524	3.768446	0.796199	H	8.470949	-1.058990	2.910257	H	-2.234545	0.165319	2.214516
C	5.315674	-0.465142	-1.181132	H	8.282499	-3.769803	2.184235	H	-2.640587	-2.523451	2.892339
C	6.412470	-0.326861	-0.281998	H	4.897597	-5.647234	-0.972752	H	-8.475382	-2.904090	-2.670835
C	5.233615	-1.837976	-1.562947	H	7.037064	-5.368504	0.830552	H	-7.628135	-4.952393	-0.953645
C	6.997535	-1.612149	-0.093867	C	-5.767253	0.056021	0.650309	H	-3.987421	-4.503228	2.426263
C	6.268106	-2.546554	-0.885198	C	-6.819562	-0.459562	-0.164659	H	-6.058432	-5.512021	0.826330
C	4.206288	0.379927	-1.169352	C	-5.338846	-0.990803	1.520550	H	-4.736430	2.557144	-1.391344
C	6.464847	0.674826	0.685854	C	-7.041403	-1.819063	0.205563	O	0.015990	6.668351	-0.125078

Compound **11-C(CN)$_2$** (G = -2255.923255 a.u.)

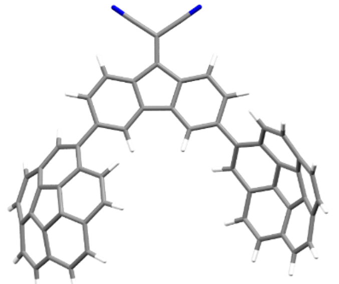

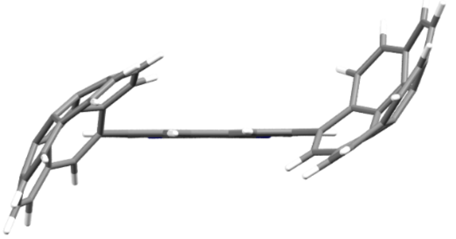

C	0.970793	2.757034	0.066645	C	6.087871	-4.424700	-0.527398	C	-3.934553	-1.653678	1.951902

C	0.100935	4.942123	0.050101	C	4.329432	1.048384	-0.324102	C	-7.500034	-3.289260	-0.698094
C	-1.007773	4.001988	0.297673	C	2.971230	-0.687667	-1.793808	C	-5.550003	-4.019514	1.361054
C	1.925767	1.751498	-0.010148	C	5.445261	1.176776	0.524739	C	-5.327251	1.228432	-0.932571
C	3.279159	2.093173	-0.254747	C	7.354398	-0.124361	1.751896	C	-3.585047	0.609800	0.939818
C	3.624640	3.454838	-0.395809	C	2.871813	-2.035734	-2.142724	C	-6.374717	0.741485	-1.716555
C	2.665828	4.473786	-0.314431	C	3.921208	-4.410279	-1.752706	C	-7.827390	-1.368832	-2.244221
C	1.325354	4.130007	-0.087866	C	7.912412	-1.388996	1.944027	C	-3.144177	-0.466122	1.732238
H	1.650599	0.711688	0.152121	C	7.703591	-3.922214	1.299329	C	-3.502117	-2.986866	2.325092
H	4.663494	3.713129	-0.593393	C	4.936017	-5.102982	-1.089303	C	-8.023956	-2.711631	-1.920450
H	2.976295	5.506912	-0.436281	C	6.966551	-4.840762	0.548785	C	-7.173912	-4.671285	-0.406374
C	-2.686155	1.761878	0.712700	H	2.098126	-0.054383	-1.940120	C	-4.273070	-4.114729	2.041788
C	-1.295234	1.574354	0.512861	H	5.461515	2.026743	1.207630	C	-6.243904	-5.019614	0.574993
C	-0.476815	2.677394	0.312749	H	7.521133	0.641018	2.510619	H	-6.585485	1.237245	-2.664929
C	-2.383553	4.193675	0.491898	H	1.918508	-2.402591	-2.525343	H	-8.154174	-1.014625	-3.222599
C	-3.204193	3.077177	0.696033	H	3.035336	-4.961692	-2.070162	H	-2.138790	-0.413908	2.150825
H	-0.884384	0.566483	0.485319	H	8.495247	-1.568367	2.848320	H	-2.506214	-3.126491	2.747094
H	-2.825557	5.185203	0.495581	H	8.267073	-4.285940	2.159465	H	-8.498087	-3.361549	-2.657001
H	-4.267929	3.225119	0.871035	H	4.805976	-6.170976	-0.909692	H	-7.587296	-5.460576	-1.035531
C	5.261424	-0.995536	-1.184766	H	6.981776	-5.889167	0.849271	H	-3.853094	-5.099277	2.251618
C	6.377031	-0.856587	-0.309048	C	-5.713290	-0.495344	0.682628	H	-5.963152	-6.068459	0.678235
C	5.161303	-2.371678	-1.549914	C	-6.785640	-0.991046	-0.118116	H	-4.760723	2.081508	-1.302457
C	6.955642	-2.144100	-0.118299	C	-5.252230	-1.568423	1.503239	C	0.010706	6.325518	-0.035687
C	6.203052	-3.081085	-0.884304	C	-6.987412	-2.363982	0.211767	C	-1.218064	7.038068	0.111769
C	4.159656	-0.140984	-1.161925	C	-6.039109	-2.720279	1.215081	C	1.137797	7.167730	-0.279924
C	6.459025	0.156390	0.644937	C	-4.891847	0.555503	0.276567	N	2.043822	7.888921	-0.478662
C	3.956008	-2.969991	-1.913662	C	-7.086958	-0.472324	-1.377700	N	-2.211878	7.653968	0.226866
C	7.635533	-2.495974	1.047829								

Compound **16-Ntol** (G = -2319.646181 a.u.)

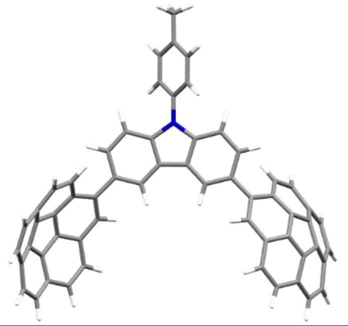

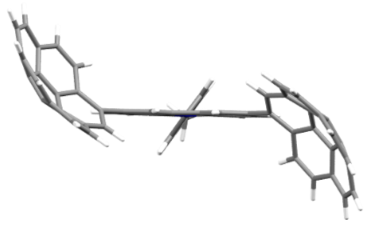

C	0.705080	1.765795	0.169589	C	3.656140	-1.109782	1.666159	C	-4.268550	-3.599266	-2.190330
N	0.000414	3.949208	-0.002825	C	4.265681	-3.599693	2.189309	C	-6.824748	0.538236	1.652216
C	-1.107242	3.132561	-0.268680	C	6.826479	0.535999	-1.652135	C	-8.512185	-1.385032	2.196798
C	1.646050	0.749443	0.374200	C	8.513913	-1.387893	-2.194572	C	-5.162970	-4.629999	-1.897819
C	2.981430	1.081257	0.670396	C	5.160132	-4.630735	1.897987	C	-7.258792	-5.272602	-0.465820
C	3.353073	2.451400	0.738935	C	7.257130	-5.274307	0.468148	C	-8.845030	-2.708645	1.906462
C	2.435401	3.484797	0.537408	C	8.846162	-2.711498	-1.903508	C	-8.172117	-4.795927	0.476659
C	1.107655	3.132274	0.263642	C	8.171437	-4.798158	-0.473646	H	-5.060670	1.679091	1.245768
H	1.355983	-0.295663	0.274309	H	5.062310	1.677452	-1.247616	H	-2.641128	-1.176707	-2.056828
H	4.386092	2.699781	0.977279	H	2.638960	-1.176794	2.053452	H	-3.281208	-3.858329	-2.575074
H	2.742965	4.526410	0.596174	H	3.277917	-3.858393	2.573214	H	-7.003603	1.082132	2.580790
C	-2.981735	1.081830	-0.674030	H	7.006340	1.079567	-2.580708	H	-8.823491	-0.967170	3.155192
C	-1.646164	0.749788	-0.378977	H	8.826202	-0.970423	-3.152816	H	-4.843707	-5.659789	-2.064286
C	-0.704887	1.765971	-0.174953	H	4.840471	-5.660392	2.064507	H	-7.089365	-6.348426	-0.529314
C	-2.435185	3.485278	-0.541231	H	7.087388	-6.350070	0.531836	H	-9.405622	-3.279961	2.647672
C	-3.353177	2.452043	-0.742097	H	9.407290	-3.283189	-2.644023	H	-8.684559	-5.515844	1.116302
H	-1.356211	-0.295358	-0.279182	H	8.684294	-5.518409	-1.112580	C	0.000542	5.371511	-0.000496
H	-2.742860	4.526890	-0.599221	C	-6.241191	-0.836866	-0.692695	C	0.467933	6.073546	1.121844
H	-4.386384	2.700646	-0.979379	C	-5.878393	-1.977744	-1.469152	C	0.471457	7.471694	1.113898
C	6.240399	-0.838251	0.692646	C	-7.380634	-1.189201	0.092126	C	-0.005237	8.197783	0.007078
C	5.876594	-1.978793	1.469122	C	-6.790129	-3.030135	-1.167170	C	-0.482380	7.475569	-1.102314
C	7.380481	-1.191113	-0.091006	C	-7.720892	-2.542683	-0.203013	C	-0.471403	6.077652	-1.118458
C	6.788360	-3.031500	1.168332	C	-5.318768	0.129489	-0.290919	H	0.810551	5.521693	1.995200
C	7.720133	-2.544585	0.204881	C	-4.564504	-2.215674	-1.871851	H	0.835568	8.008723	1.990297
C	5.318595	0.128210	0.289711	C	-7.656544	-0.601066	1.327133	C	0.020395	9.708728	-0.001958
C	4.562265	-2.216277	1.870649	C	-6.440766	-4.378455	-1.260276	H	-0.856143	8.015617	-1.972793
C	7.657675	-0.603431	-1.325942	C	-8.354774	-3.378336	0.717615	H	-0.819549	5.529104	-1.991710
C	6.438584	-4.379705	1.261554	C	-3.996456	0.028691	-0.915860	H	-0.805625	10.119832	-0.596072
C	8.354657	-3.380689	-0.714896	C	-5.710092	0.882803	0.885233	H	-0.049554	10.116460	1.014343
C	3.995670	0.027919	0.913407	C	-3.657920	-1.109326	-1.668582	H	0.957402	10.083106	-0.441769
C	5.711208	0.881093	-0.886282								

Natural Localized Molecular Orbitals (NLMOs)[5] were computed on the optimized structures of all hosts with NBO 7.0[6] as implemented in Gaussian 16 and visualized with Gauss View 6.1.1.[7]

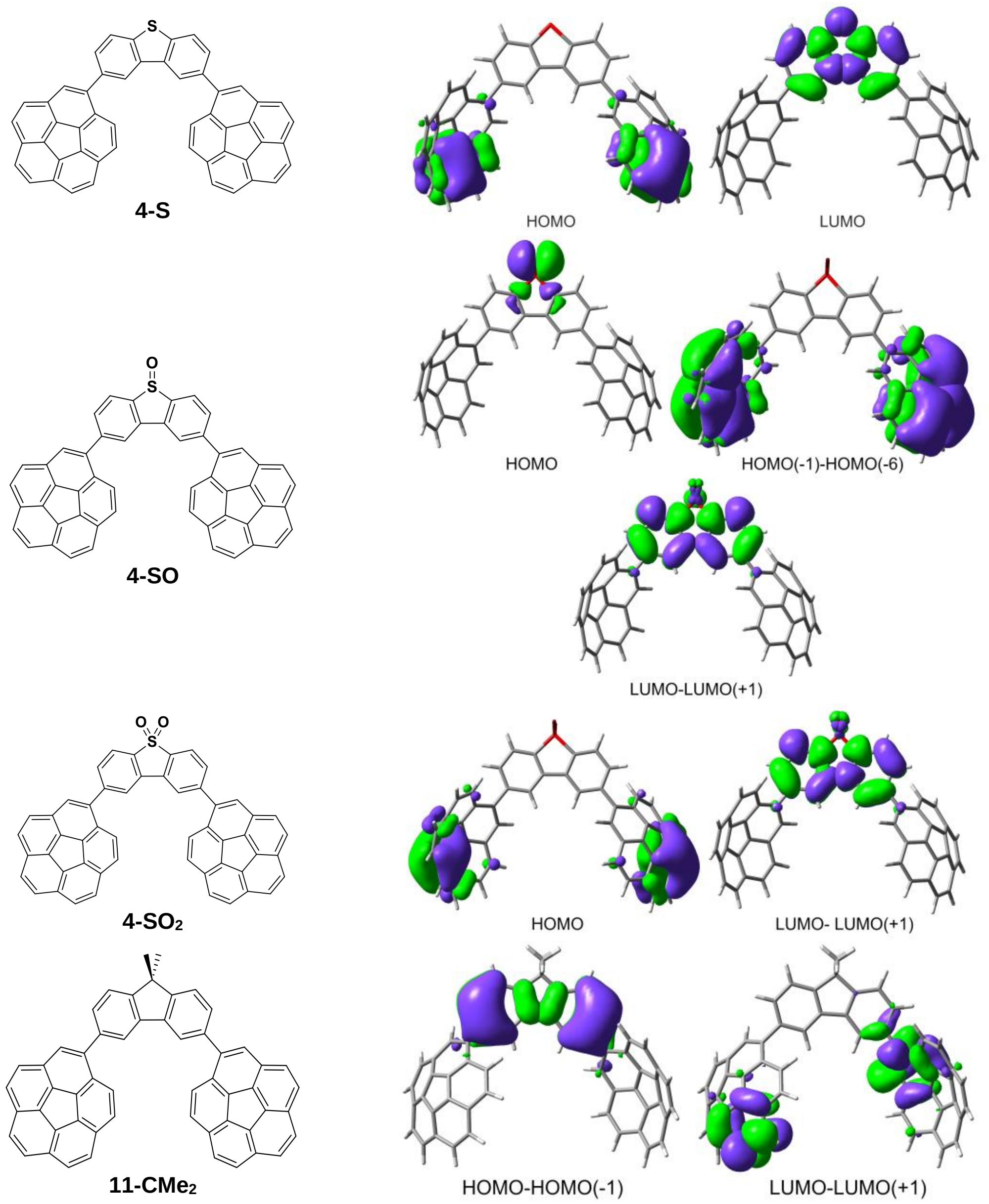

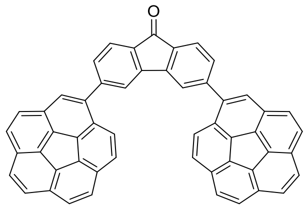

11-CO

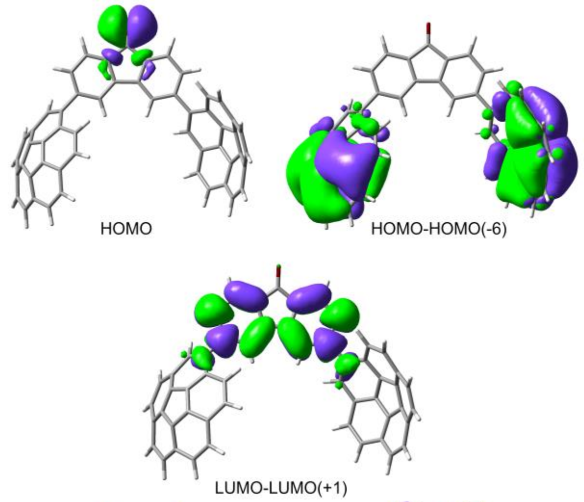

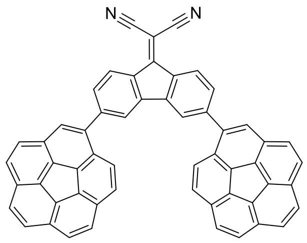

11-C(CN)$_2$

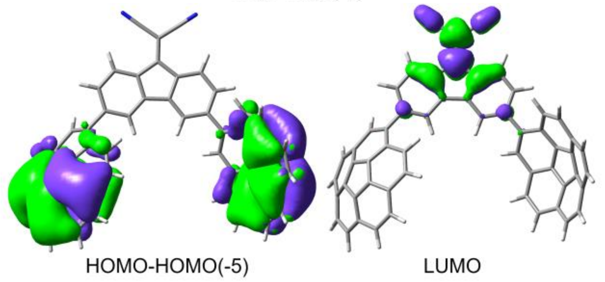

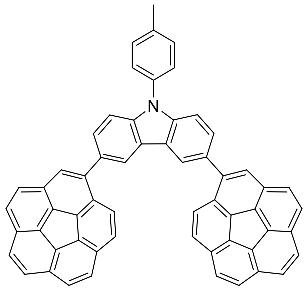

16

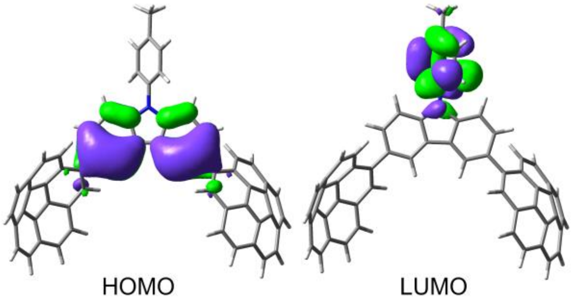

Figure S 170. Natural Localized Molecular Orbitals (NLMOs) of fullerene receptors synthesized in this work. Representation of frontier orbitals (in some cases the sum of quasi-degenerated frontier orbitals due to their proximity in energy) of compounds **4-S**, **4-SO**, **4-SO$_2$**, **11-CMe$_2$**, **11-CMe$_2$**, **11-CO**, **11-C(CN)$_2$** and **16**. Isodensities plotted at 0.02 e·au^{-3}.

The electrostatic surface potential (ESP) of all hosts was plotted as a mapped surface over the computed DFT density in Gauss View 6.1.1.

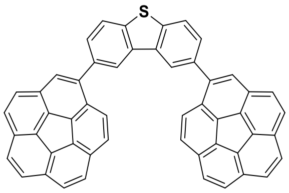

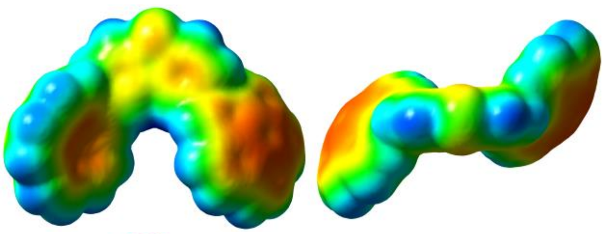

4-S

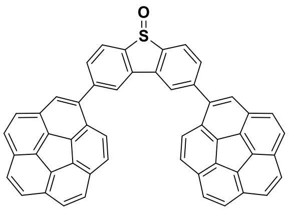

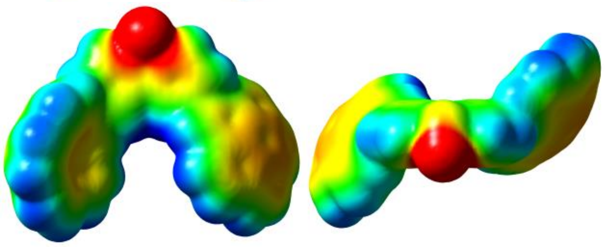

4-SO

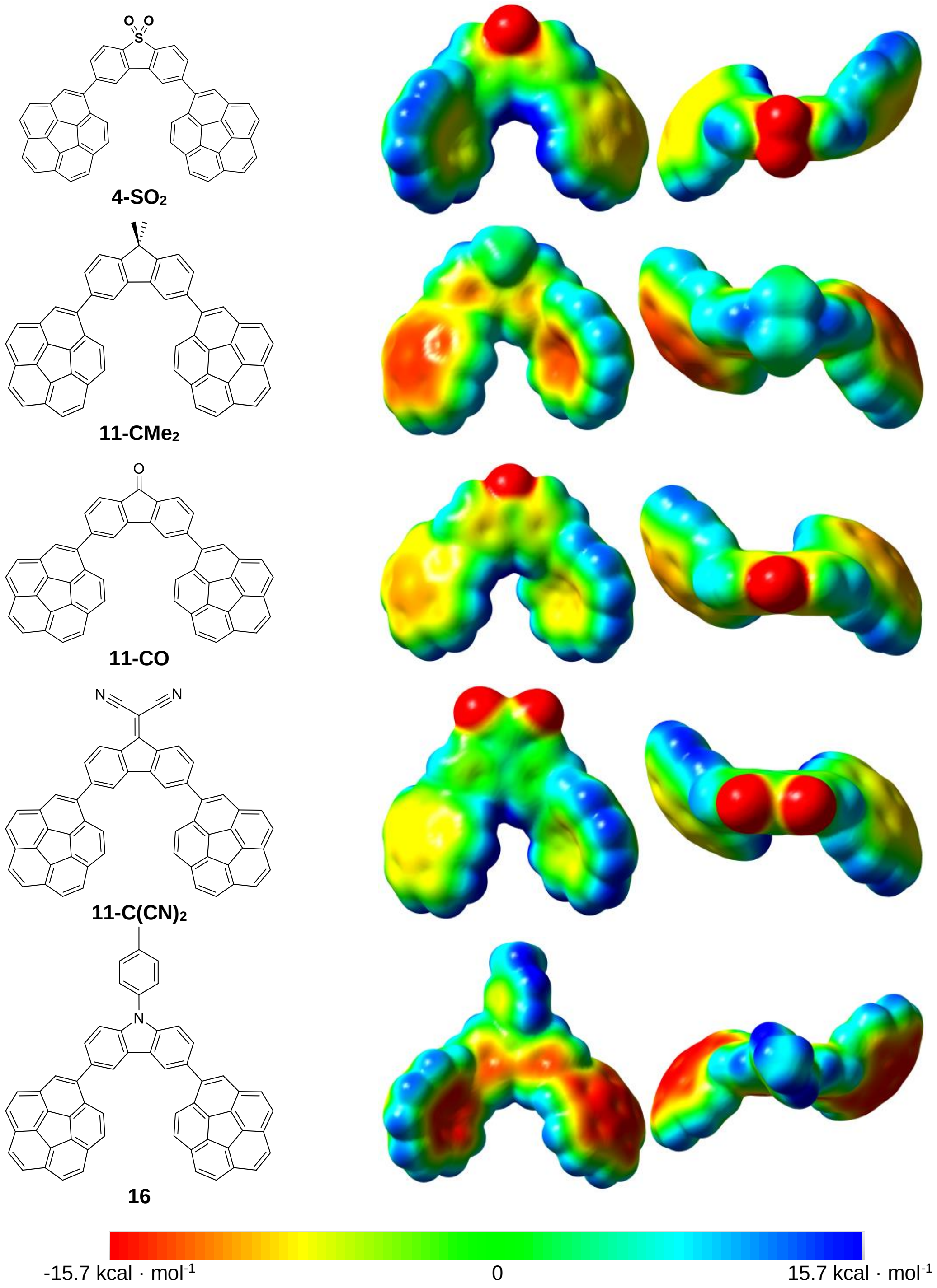

Figure S 171. Electrostatic surface potential (ESP) of fullerene receptors synthesized in this work plotted as a mapped surface over the computed DFT density (with an isovalue of 0.0004 e·au^{-3}).

In order to obtain the Buckminsterfullerene supramolecular adducts geometries, namely C_{60}@**4-S**, C_{60}@**4-SO**, C_{60}@**4-SO₂**, C_{60}@**11-CMe₂,** C_{60}@**11-CO**, C_{60}@**11-C(CN)₂** and C_{60}@**16**, a similar approach was followed. The two most stable geometries of parent hosts were utilized as starting structures to where a molecule of C_{60} was placed within the cavity of both corannulene moieties. In some cases, slight bond rotations had to be done to avoid atom overlapping. Resulting structures were freely minimized in solution and the most stable ones are shown below.

C_{60}@**4-S**

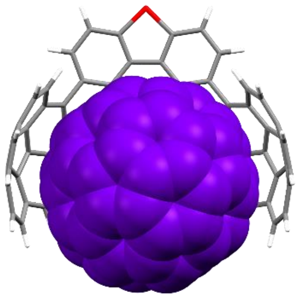

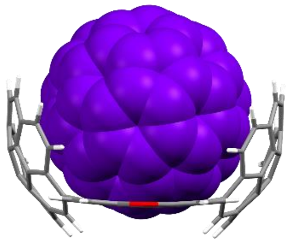

C	0.743711	5.405362	-0.064526	H	4.155392	3.498608	-2.083323	C	3.507496	-2.452023	-0.796145
S	0.016611	7.465201	-1.580851	H	3.028001	3.541322	2.773401	C	3.099045	-2.218987	-2.177873
C	-1.244601	6.454753	-0.859041	H	3.507008	1.409422	4.528297	C	2.792059	-0.798238	-2.323134
C	1.622707	4.488718	0.533327	H	5.223283	1.709779	-3.335364	C	3.011159	-0.156612	-1.031216
C	3.004200	4.567923	0.300204	C	3.454082	-1.176358	-0.088808	C	-2.646239	-1.561117	-2.806366
C	3.506446	5.621508	-0.504790	H	6.311045	-0.862327	-3.138219	C	-1.510039	-1.868033	-3.670099
C	2.658053	6.570827	-1.077642	H	4.444962	-0.831699	4.706015	C	-1.201904	-3.287290	-3.523892
C	1.277855	6.449244	-0.868041	H	5.676216	-2.891133	3.259067	C	-3.138542	-0.291448	-0.733495
H	1.226625	3.672781	1.131764	H	6.750453	-2.700582	-1.601409	C	-2.336226	0.704917	-0.035333
H	4.580073	5.690112	-0.673541	H	6.480575	-3.544018	1.055119	C	-1.393816	1.277170	-0.992400
H	3.062269	7.375740	-1.689221	C	-5.692450	1.970909	0.460356	C	1.933056	-4.407217	-2.170117
C	-2.972873	4.577772	0.308959	C	-6.313589	0.921397	-0.281985	C	0.577667	-4.742689	-2.593253
C	-1.590752	4.485964	0.533884	C	-5.595301	1.547542	1.818676	C	0.133085	-3.720843	-3.536417
C	-0.711201	5.406082	-0.060772	C	-6.604512	-0.144886	0.619258	C	1.213641	-2.753365	-3.696471
C	-2.624613	6.584862	-1.062289	C	-6.160070	0.242355	1.917957	C	2.326027	-3.177064	-2.851410
C	-3.474100	5.637985	-0.487714	C	-4.801981	2.876115	-0.114755	C	0.081450	-5.524738	-0.293324
H	-1.197585	3.659587	1.121310	C	-6.066232	0.710646	-1.638533	C	-1.052176	-5.219334	0.573487
H	-3.027285	7.392980	-1.670639	C	-4.588178	1.998854	2.671558	C	-2.164436	-4.796749	-0.272340
H	-4.548246	5.710575	-0.651478	C	-6.666509	-1.478459	0.213245	C	-1.718410	-4.841791	-1.661276
C	5.715352	1.950048	0.495488	C	-5.757429	-0.681175	2.883091	C	-0.330110	-5.291679	-1.674104
C	5.582687	1.516949	1.848856	C	-4.704541	2.791369	-1.559976	C	-3.021047	-3.770145	0.155890
C	6.341507	0.899197	-0.239694	C	-3.897068	3.539073	0.825785	C	-2.796893	-3.124628	1.446658
C	6.129948	0.204745	1.951024	C	-5.308879	1.763089	-2.284450	C	-3.101214	-1.705587	1.298779
C	6.599463	-0.177828	0.659717	C	-6.346502	-0.633373	-2.105254	C	-3.512430	-1.472217	-0.079359
C	4.847414	2.867612	-0.095240	C	-3.805280	3.099845	2.155726	C	-3.462611	-2.747622	-0.787368
C	4.560294	1.971832	2.680976	C	-4.326185	1.133724	3.806283	C	-2.330970	-0.748326	1.972491
C	6.127424	0.702271	-1.604372	C	-6.633857	-1.675430	-1.223550	C	-1.221479	-1.170761	2.821921
C	5.687646	-0.723227	2.895106	C	-6.466817	-2.444387	1.275216	C	-0.140712	-0.201560	2.665077
C	6.655179	-1.508186	0.243453	C	-4.884971	-0.141485	3.907397	C	-0.586896	0.819105	1.718433
C	3.919494	3.526311	0.827098	C	-6.031785	-2.064521	2.546624	C	-1.940778	0.480434	1.291946
C	4.788458	2.796840	-1.543081	H	-5.104259	1.692951	-3.353109	C	1.193887	-0.635947	2.649004
C	3.795971	3.078945	2.152563	H	-6.223103	-0.865881	-3.163374	C	1.502499	-2.056127	2.796260
C	4.258627	1.100786	3.801116	H	-3.049548	3.562475	2.791339	C	2.633748	-2.362903	1.926581
C	5.400890	1.768839	-2.261073	H	-3.589176	1.439234	4.549770	C	3.024296	-1.133622	1.243466
C	6.406953	-0.640298	-2.074998	H	-6.723983	-2.685546	-1.623861	C	2.135639	-0.068485	1.689141
C	4.797523	-0.182593	3.903835	H	-6.545851	-3.508239	1.049451	C	2.688207	-3.589775	1.248411
C	5.950545	-2.107256	2.552695	H	-4.561472	-0.786401	4.725149	C	1.606383	-4.556409	1.405675
C	6.658940	-1.693101	-1.194738	H	-5.789310	-2.845933	3.267493	C	1.385125	-5.200801	0.114630
C	6.410691	-2.481499	1.289441	H	-4.051762	3.483307	-2.089354	C	2.329127	-4.630938	-0.841736
C	-1.615487	0.634430	-2.284943	C	1.724041	-0.390106	-3.138089	C	3.136072	-3.635557	-0.141613
C	-2.694202	-0.336364	-2.123835	C	0.917848	-1.388060	-3.835898	C	0.515805	-4.262411	2.240205
C	0.320820	1.360488	0.794925	C	-0.470007	-0.936740	-3.823576	C	0.462260	-2.987335	2.948891
C	1.709660	0.911396	0.782247	C	-0.523117	0.339544	-3.116908	C	-0.925956	-2.536094	2.963282
C	2.157657	0.870841	-0.604580	C	0.833441	0.678707	-2.694339	C	-1.730573	-3.533094	2.262090
C	1.046384	1.296074	-1.451229	C	-2.145783	-3.858160	-2.567744	C	-0.839122	-4.599282	1.814921
C	-0.089913	1.598023	-0.586307	C	-3.038298	-2.792478	-2.123762				

C_{60}@**4-SO**

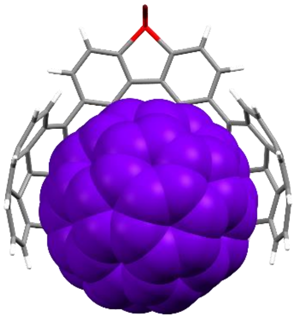
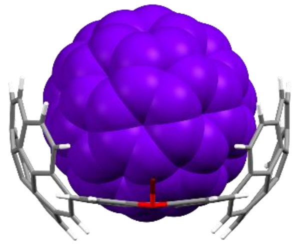

C	0.755939	5.338451	0.197094	H	6.740800	-2.746017	-1.690255	C	-2.696178	-0.352973	-2.094136
S	0.023657	7.584452	-1.132894	H	6.489594	-3.672951	0.940301	C	0.311212	1.239048	0.891845
C	-1.246286	6.410412	-0.550815	C	-5.689550	1.864287	0.524767	C	1.700671	0.792186	0.863805
C	1.626288	4.390165	0.744954	C	-6.309800	0.840886	-0.253641	C	2.150468	0.804741	-0.523058
C	3.008968	4.461994	0.477925	C	-5.603281	1.398583	1.870001	C	1.039950	1.260423	-1.354674
C	3.509238	5.529717	-0.301997	C	-6.609917	-0.251900	0.612145	C	-0.098000	1.527770	-0.480406
C	2.655441	6.519848	-0.806741	C	-6.173558	0.093248	1.925263	C	3.449784	-1.258450	-0.083346
C	1.290422	6.403323	-0.559610	C	-4.793177	2.784380	-0.016286	C	3.506785	-2.506168	-0.838522
H	1.236218	3.553868	1.318910	C	-6.054553	0.671944	-1.614438	C	3.099920	-2.221640	-2.211079
H	4.577759	5.581801	-0.503845	C	-4.602663	1.820269	2.745125	C	2.790121	-0.797133	-2.302588
H	3.047790	7.344723	-1.399789	C	-6.672012	-1.571952	0.164377	C	3.005891	-0.204707	-0.987007
C	-2.970390	4.474958	0.484494	C	-5.779703	-0.860728	2.864014	C	1.938068	-4.410524	-2.287814
C	-1.587188	4.390184	0.745120	C	-4.686714	2.744842	-1.462913	C	0.583931	-4.732150	-2.725321
C	-0.714656	5.340104	0.200476	C	-3.894349	3.413574	0.952770	C	0.138806	-3.676185	-3.629814
C	-2.610903	6.535782	-0.794038	C	-5.290138	1.741754	-2.222464	C	1.217676	-2.701310	-3.751555
C	-3.467534	5.548740	-0.288875	C	-6.335113	-0.656076	-2.124722	C	2.329655	-3.154784	-2.921540
H	-1.200791	3.545836	1.311137	C	-3.812049	2.932897	2.268892	C	0.085996	-5.601701	-0.457416
H	-3.000339	7.363868	-1.384499	C	-4.351369	0.920381	3.854844	C	-1.049380	-5.331499	0.418691
H	-4.536439	5.605532	-0.487559	C	-6.630209	-1.724300	-1.277597	C	-2.161341	-4.879248	-0.412179
C	5.715306	1.834159	0.559079	C	-6.481613	-2.570902	1.197162	C	-1.713165	-4.870662	-1.801116
C	5.593146	1.358280	1.899122	C	-4.913829	-0.355747	3.911469	C	-0.324056	-5.317228	-1.828929
C	6.338184	0.808280	-0.212959	C	-6.054754	-2.232208	2.482846	C	-3.020449	-3.871187	0.053393
C	6.143627	0.044804	1.956430	H	-5.079378	1.705129	-3.291483	C	-2.799336	-3.274648	1.368114
C	6.603974	-0.296056	0.650338	H	-6.205705	-0.855706	-3.188730	C	-3.106043	-1.851639	1.273768
C	4.843044	2.769228	0.003573	H	-3.061357	3.373981	2.925331	C	-3.515539	-1.566999	-0.095384
C	4.578650	1.785813	2.754765	H	-3.619895	1.201301	4.613260	C	-3.462564	-2.814499	-0.851135
C	6.114733	0.654277	-1.581526	H	-6.720008	-2.721189	-1.709756	C	-2.338319	-0.919263	1.984429
C	5.708670	-0.912654	2.874059	H	-6.561641	-3.626969	0.937858	C	-1.229260	-1.371364	2.818859
C	6.657566	-1.612495	0.191928	H	-4.598786	-1.026675	4.711330	C	-0.150115	-0.394820	2.700423
C	3.922840	3.395835	0.955451	H	-5.819486	-3.036296	3.180688	C	-0.596848	0.660209	1.792598
C	4.773718	2.744320	-1.445480	H	-4.028638	3.449629	-1.968081	C	-1.949240	0.335003	1.351416
C	3.808454	2.906723	2.266829	O	0.017078	7.733402	-2.650264	C	1.185365	-0.825907	2.669852
C	4.286615	0.880487	3.849838	C	1.722834	-0.360061	-3.103075	C	1.496405	-2.250195	2.763646
C	5.382394	1.740119	-2.199246	C	0.919577	-1.332213	-3.839669	C	2.629654	-2.521801	1.884832
C	6.392331	-0.672343	-2.096303	C	-0.469014	-0.884256	-3.812147	C	3.018529	-1.266896	1.249086
C	4.826744	-0.405007	3.906964	C	-0.525372	0.364274	-3.057618	C	2.127359	-0.220741	1.733624
C	5.969607	-2.284973	2.486254	C	0.829790	0.689648	-2.620398	C	2.687015	-3.721906	1.160372
C	6.651218	-1.752027	-1.251331	C	-2.141096	-3.854260	-2.670396	C	1.606745	-4.695786	1.279220
C	6.420849	-2.618436	1.208502	C	-3.036113	-2.807708	-2.187607	C	1.388459	-5.291108	-0.035629
H	4.136353	3.460781	-1.960652	C	-2.645321	-1.550758	-2.822554	C	2.332633	-4.683715	-0.968353
H	3.046472	3.349103	2.909144	C	-1.507394	-1.822650	-3.695729	C	3.136725	-3.714186	-0.229775
H	3.541672	1.166145	4.593009	C	-1.196961	-3.245883	-3.602865	C	0.514516	-4.435643	2.122698
H	5.197591	1.715296	-3.273540	C	-3.142415	-0.361900	-0.703843	C	0.457684	-3.188373	2.878993
H	6.289467	-0.860739	-3.165259	C	-2.342637	0.608378	0.032843	C	-0.931374	-2.740583	2.908539
H	4.481642	-1.079409	4.691265	C	-1.400504	1.218905	-0.900447	C	-1.733209	-3.711671	2.168913
H	5.701036	-3.091067	3.169343	C	-1.619271	0.625372	-2.216740	C	-0.839215	-4.758625	1.683067

C_{60}@**4-SO$_2$**

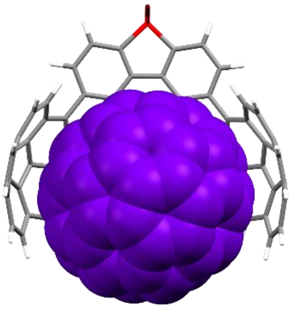
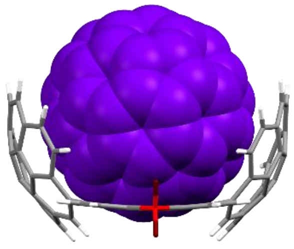

C	-0.776391	5.243212	-0.206302	H	-6.459909	-3.791110	-0.973328	C	-2.623019	-2.621382	-1.850139
S	-0.047313	7.379561	1.193126	C	5.676629	1.784831	-0.587989	C	0.628336	0.535835	-1.800640
C	1.249969	6.316198	0.531988	C	6.309390	0.767613	0.188212	C	1.977505	0.206416	-1.352891

C	-1.633103	4.284935	-0.755599	C	5.572798	1.310657	-1.929017	C	2.357195	-1.060034	-1.967091
C	-3.022799	4.360988	-0.525014	C	6.600555	-0.329505	-0.674890	C	1.245699	-1.515435	-2.796366
C	-3.543695	5.438430	0.226967	C	6.145894	0.006575	-1.984129	C	0.174256	-0.528652	-2.693659
C	-2.702602	6.426446	0.756007	C	4.787032	2.707261	-0.040470	C	3.526965	-1.686271	0.123414
C	-1.334926	6.302975	0.535635	C	6.073355	0.606647	1.553418	C	3.463664	-2.922083	0.897273
H	-1.230317	3.440837	-1.308299	C	4.559858	1.724753	-2.793472	C	3.013960	-3.988448	0.007765
H	-4.617678	5.497220	0.393542	C	6.673000	-1.646477	-0.219963	C	2.798969	-3.409670	-1.315811
H	-3.105633	7.255059	1.335150	C	5.742638	-0.953886	-2.911975	C	3.116504	-1.987829	-1.241921
C	2.956668	4.391683	-0.526662	C	4.700715	2.677059	1.407758	C	1.696654	-4.950055	1.875115
C	1.567932	4.297086	-0.756018	C	3.874288	3.327914	-1.001861	C	0.304061	-5.385205	1.907725
C	0.702038	5.249509	-0.208167	C	5.315602	1.679276	2.165109	C	-0.106671	-5.686535	0.540045
C	2.615874	6.455141	0.752454	C	6.365174	-0.717260	2.068120	C	1.031804	-5.438291	-0.338511
C	3.466396	5.475409	0.223694	C	3.773020	2.838561	-2.313233	C	2.146356	-4.982694	0.487012
H	1.175095	3.446179	-1.306530	C	4.295774	0.817494	-3.894108	C	-0.147847	-3.714463	3.683758
H	3.009986	7.288031	1.331581	C	6.651521	-1.789897	1.223504	C	-1.219143	-2.729396	3.789803
H	4.539736	5.543779	0.390893	C	6.472563	-2.651932	-1.244602	C	-2.333765	-3.186107	2.965138
C	-5.714967	1.721893	-0.625222	C	4.860771	-0.457602	-3.950370	C	-1.951338	-4.454032	2.350412
C	-5.571300	1.235899	-1.959459	C	6.027601	-2.322162	-2.526541	C	-0.600312	-4.779977	2.794308
C	-6.344084	0.699446	0.146201	H	5.120284	1.649637	3.237254	C	-3.096198	-2.257505	2.240287
C	-6.116074	-0.080099	-2.014207	H	6.251623	-0.910580	3.135084	C	-2.775196	-0.834268	2.311434
C	-6.593135	-0.412681	-0.711974	H	3.012890	3.274532	-2.962171	C	-2.984691	-0.259502	0.986958
C	-4.855629	2.665751	-0.064908	H	3.553487	1.091876	-4.644308	C	-3.436081	-1.322829	0.098112
C	-4.546345	1.660527	-2.804104	H	6.750757	-2.783718	1.660636	C	-3.503788	-2.558879	0.871503
C	-6.138572	0.557214	1.518824	H	6.559771	-3.706255	-0.980581	C	-2.120716	0.736263	0.509287
C	-5.664376	-1.042833	-2.918051	H	4.537135	-1.133900	-4.742180	C	-1.007475	1.194959	1.335577
C	-6.647088	-1.725602	-0.243704	H	5.786227	-3.131222	-3.216477	C	0.133606	1.439930	0.459019
C	-3.924044	3.286985	-1.008752	H	4.049935	3.385032	1.918019	C	-0.276311	1.134993	-0.909656
C	-4.807083	2.653582	1.385035	O	-0.055094	8.695920	0.509704	C	-1.669390	0.699626	-0.876695
C	-3.786706	2.787656	-2.313844	O	-0.044543	7.348545	2.675891	C	1.433215	1.127215	0.885101
C	-4.235261	0.747885	-3.887845	C	1.730323	-3.849877	-2.111443	C	1.645960	0.551737	2.210251
C	-5.420664	1.651836	2.138066	C	0.827593	-4.882491	-1.611470	C	2.715477	-0.436582	2.103285
C	-6.416973	-0.766467	2.040442	C	-0.523025	-4.555250	-2.057451	C	3.162999	-0.469450	0.713792
C	-4.769936	-0.539931	-3.942510	C	-0.455491	-3.319670	-2.831796	C	2.371691	0.496097	-0.038107
C	-5.925111	-2.413128	-2.523089	C	0.937073	-2.883389	-2.866289	C	2.654095	-1.623200	2.849032
C	-6.659722	-1.853840	1.200521	C	-2.346496	-4.743320	1.034664	C	1.512915	-1.873254	3.724571
C	-6.391961	-2.738453	-1.248867	C	-3.142037	-3.778396	0.280981	C	1.191329	-3.295167	3.652148
H	-4.181976	3.378344	1.903753	C	-2.690663	-3.810096	-1.108234	C	2.131732	-3.924592	2.729970
H	-3.016572	3.227583	-2.948080	C	-1.618152	-4.794163	-1.211512	C	3.035718	-2.892347	2.233059
H	-3.481215	1.030799	-4.622757	C	-1.406111	-5.371867	0.112175	C	0.481841	-0.925164	3.826051
H	-5.251642	1.636959	3.215113	C	-3.002961	-1.354305	-1.233430	C	0.548944	0.311601	3.053355
H	-6.327969	-0.945877	3.112164	C	-2.102970	-0.322506	-1.732182	C	-0.803138	0.641170	2.609806
H	-4.411551	-1.219008	-4.716699	C	-1.164604	-0.948750	-2.658149	C	-1.705024	-0.394165	3.106601
H	-5.644027	-3.223375	-3.196093	C	-1.486901	-2.371724	-2.731556	C	-0.910316	-1.361613	3.858435
H	-6.751046	-2.844740	1.645981								

C_{60}@**11-CMe$_2$**

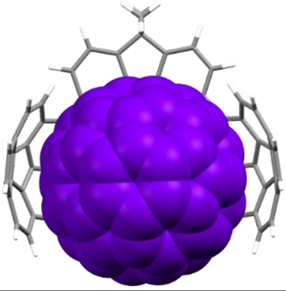

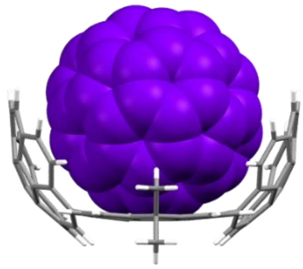

C	-0.737459	5.116576	-0.035089	C	6.391984	0.717387	-0.152213	C	-0.927444	0.788134	-1.455175
C	-0.011851	6.817661	1.531554	C	5.517675	1.385539	-2.178550	C	-0.895803	-0.318483	-2.406953
C	1.177702	6.091579	0.892230	C	6.578437	-0.345896	-1.086029	C	-2.065798	-1.155616	-2.158445
C	-1.628521	4.268412	-0.693073	C	6.035175	0.067000	-2.337830	C	0.320382	1.607783	0.528113
C	-3.010423	4.368793	-0.433282	C	4.900744	2.696728	-0.167267	C	1.627826	1.213335	1.039336
C	-3.463739	5.368091	0.459177	C	6.257446	0.490134	1.217647	C	2.382329	0.620603	-0.061314
C	-2.565045	6.229771	1.111476	C	4.455905	1.870410	-2.941283	C	1.542663	0.653117	-1.255032
C	-1.195618	6.092370	0.880645	C	6.641443	-1.685984	-0.701169	C	0.267147	1.258877	-0.889577
H	-1.259200	3.484881	-1.349363	C	5.518984	-0.837240	-3.267894	C	3.331077	-1.031849	1.519033
H	-4.533068	5.465815	0.642256	C	4.908970	2.577728	1.279217	C	3.436568	-2.482678	1.403483
H	-2.943587	6.982714	1.803354	C	3.931705	3.389266	-1.021115	C	3.384806	-2.826070	-0.014566
C	3.004243	4.381730	-0.424404	C	5.560137	1.534267	1.937356	C	3.247534	-1.587787	-0.773276
C	1.626481	4.295520	-0.707626	C	6.551565	-0.863713	1.643023	C	3.219104	-0.478542	0.173652
C	0.727544	5.123372	-0.035077	C	3.742222	2.977790	-2.351743	C	2.062395	-4.844060	0.552629
C	2.545046	6.224125	1.138608	C	4.067649	1.024429	-4.052582	C	0.820749	-5.339279	-0.032504
C	3.448864	5.363978	0.491016	C	6.733616	-1.901772	0.728540	C	0.704409	-4.786318	-1.378652
H	1.265269	3.533992	-1.392827	C	6.319608	-2.635252	-1.748152	C	1.875243	-3.950942	-1.626953
H	2.917726	6.969781	1.841453	C	4.572827	-0.267092	-4.206468	C	2.714274	-3.985581	-0.432285
H	4.515469	5.450701	0.694402	C	5.782732	-2.230532	-2.971135	C	-0.271579	-5.141858	2.185316
C	-5.738210	1.779920	-0.867788	H	5.433231	1.443697	3.016443	C	-1.545524	-4.529739	2.548416
C	-5.489732	1.358592	-2.208303	H	6.516229	-1.106547	2.705416	C	-2.384923	-4.494821	1.354433
C	-6.403702	0.709977	-0.193614	H	2.950809	3.461539	-2.924306	C	-1.629849	-5.086166	0.254387
C	-5.994801	0.034656	-2.361930	H	3.277183	1.358683	-4.725311	C	-0.323517	-5.485632	0.767694
C	-6.561364	-0.366328	-1.116587	H	6.836423	-2.918521	1.108717	C	-3.223339	-3.393693	1.117507

C	-4.929192	2.702614	-0.201007
C	-4.415283	1.840813	-2.954797
C	-6.300731	0.504049	1.182446
C	-5.452757	-0.875651	-3.270768
C	-6.618559	-1.702102	-0.714782
C	-3.936168	3.379701	-1.037911
C	-4.982822	2.610619	1.246521
C	-3.716630	2.954101	-2.360077
C	-4.003964	0.988015	-4.053046
C	-5.642966	1.571413	1.903734
C	-6.584802	-0.849204	1.619624
C	-4.498207	-0.308154	-4.201987
C	-5.707316	-2.267384	-2.959514
C	-6.737265	-1.900964	0.715519
C	-6.265803	-2.660951	-1.742644
H	-4.398214	3.307057	1.844257
H	-2.909201	3.429063	-2.917196
H	-3.204886	1.321300	-4.716019
H	-5.548047	1.502606	2.987864
H	-6.570066	-1.077214	2.685844
H	-4.067589	-0.943671	-4.976331
H	-5.356143	-3.039429	-3.644858
H	-6.834227	-2.914254	1.106574
H	-6.329923	-3.726328	-1.519881
C	5.734073	1.791159	-0.827138
H	6.391154	-3.702853	-1.538236
H	4.159778	-0.897346	-4.994844
H	5.452774	-2.996385	-3.674015
H	4.296340	3.255606	1.870061
C	-0.008259	8.323676	1.181667
C	-0.020994	6.620683	3.065195
H	-0.001834	8.470063	0.094544
H	-0.900054	8.814815	1.593633
H	0.879584	8.813204	1.603921
H	-0.911957	7.086121	3.507404
H	0.866623	7.082784	3.517533
H	-0.024557	5.553052	3.317790
C	2.445412	-1.554744	-1.922378
C	1.743707	-2.758895	-2.357533
C	0.436632	-2.358749	-2.871843
C	0.331644	-0.906779	-2.754723
C	1.573790	-0.411034	-2.169038
C	-1.741646	-4.552636	-1.039610
C	-2.614606	-3.409892	-1.286393
C	-1.965967	-2.550389	-2.272882
C	-0.689470	-3.162774	-2.632886
C	-0.552220	-4.401082	-1.872378
C	-2.822011	-0.562274	-1.058072
C	-2.117217	0.638054	-0.622641
C	-3.253784	-2.282945	2.065751
C	-3.388547	-1.045651	1.304883
C	-3.442552	-1.388698	-0.111428
C	-3.337615	-2.839630	-0.227689
C	-2.713859	0.110827	1.721810
C	-1.876307	0.077411	2.917252
C	-0.706620	0.916889	2.672271
C	-0.823932	1.468183	1.325343
C	-2.065002	0.969991	0.738654
C	0.550603	0.531811	3.165952
C	0.686060	-0.708173	3.924431
C	1.959827	-1.321894	3.562090
C	2.612730	-0.460631	2.579527
C	1.741914	0.685618	2.335099
C	2.060287	-2.717788	3.451450
C	0.890470	-3.554926	3.695826
C	0.922732	-4.664604	2.747894
C	2.112194	-4.513411	1.916278
C	2.815005	-3.309983	2.350495
C	-0.334922	-2.965246	4.044630
C	-0.439319	-1.514260	4.161294
C	-1.745633	-1.113507	3.648969
C	-2.448992	-2.317581	3.214907
C	-1.576453	-3.461919	3.459393

C_{60}@**11-CO**

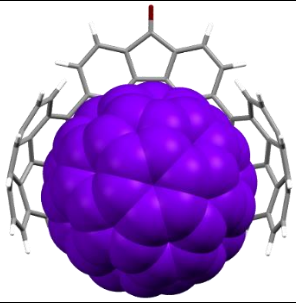

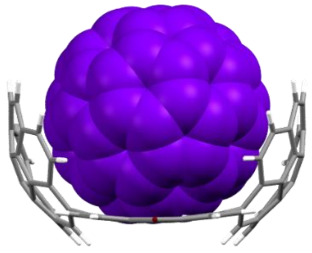

C	-0.686140	5.302197	-0.063436
C	0.062519	7.058365	1.354041
C	1.251148	6.335432	0.785703
C	-1.591541	4.416023	-0.635633
C	-2.968611	4.534874	-0.320715
C	-3.399893	5.579286	0.529067
C	-2.486782	6.487727	1.088010
C	-1.132228	6.332967	0.800446
H	-1.253181	3.595190	-1.263486
H	-4.461955	5.669229	0.751575
H	-2.820901	7.284598	1.751548
C	3.072467	4.545871	-0.374861
C	1.694816	4.449445	-0.691474
C	0.794219	5.312507	-0.081297
C	2.610198	6.487513	1.055259
C	3.515177	5.577437	0.484478
H	1.349330	3.652646	-1.343091
H	2.953921	7.279152	1.720144
H	4.578270	5.656231	0.706301
C	-5.757241	1.991056	-0.454510
C	-5.655279	1.551476	-1.808432
C	-6.392832	0.953051	0.292818
C	-6.231934	0.251227	-1.900369
C	-6.685846	-0.120208	-0.600045
C	-4.856911	2.889348	0.118729
C	-4.636570	1.981723	-2.659001
C	-6.152354	0.749720	1.652413
C	-5.822057	-0.685224	-2.850579
C	-6.750829	-1.449968	-0.181250
C	-3.930754	3.525043	-0.820564
C	-4.765076	2.810959	1.564576
C	-3.838358	3.070250	-2.146861
C	-4.371138	1.106779	-3.783336
C	-5.386503	1.796736	2.293431
C	-6.440944	-0.588308	2.130413
C	-4.936896	-0.165797	-3.873277
C	-6.097307	-2.063933	-2.501571
C	-6.725588	-1.637157	1.256223
C	-6.540276	-2.427885	-1.229154
H	-4.103744	3.494537	2.093487
H	-3.069969	3.509783	-2.783037
H	-3.624430	1.399472	-4.521868
H	-6.821827	-2.643948	1.663688
H	-6.620004	-3.488641	-0.990182
C	5.783712	1.919934	-0.487839
C	6.394844	0.876249	0.271849
C	5.660844	1.462903	-1.834344
C	6.650265	-0.218837	-0.604913
C	6.195404	0.143833	-1.907422
C	4.919351	2.857050	0.077917
C	6.167248	0.703090	1.637305
C	4.650019	1.909731	-2.684870
C	6.692315	-1.542268	-0.164582
C	5.752721	-0.797088	-2.838023
C	4.840176	2.804173	1.525864
C	4.007819	3.507700	-0.868018
C	5.437367	1.783256	2.266814
C	6.434845	-0.632609	2.135039
C	3.893362	3.033989	-2.186346
C	4.344095	1.019530	-3.788639
C	6.685639	-1.702782	1.276504
C	6.452647	-2.531074	-1.197006
C	4.869865	-0.270247	-3.860922
C	6.004592	-2.176095	-2.470551
H	5.245429	1.742951	3.339533
H	6.328911	-0.833669	3.201476
H	3.136610	3.488391	-2.826230
H	3.598687	1.321159	-4.524876
H	6.766688	-2.703818	1.701157
H	6.516310	-3.589900	-0.945088
H	4.516495	-0.932698	-4.651440
H	5.733935	-2.970558	-3.166365
H	4.205238	3.515606	2.050338
O	0.065826	8.014402	2.126360
C	1.693377	-1.990826	-2.703885
C	0.775679	-3.120315	-2.827204
C	-0.569444	-2.590010	-3.024331
C	-0.484755	-1.131920	-3.019457
C	0.913743	-0.761092	-2.823168
C	-2.403848	-4.375423	-0.500711
C	-3.183451	-3.147746	-0.619761
C	-2.730805	-2.439604	-1.813210
C	-1.669687	-3.229504	-2.431044
C	-1.468580	-4.426855	-1.620781
C	-2.647480	-1.040160	-1.806780
C	0.639296	1.555617	-0.089909
C	1.985185	1.030928	0.103258
C	2.354771	0.284667	-1.094348
C	1.239356	0.354151	-2.034318
C	0.177362	1.140081	-1.412701
C	3.509131	-1.373424	0.334581
C	3.426734	-2.829997	0.331100
C	2.964828	-3.252420	-0.988086
C	2.762051	-2.055200	-1.797640
C	3.101737	-0.895032	-0.979790
C	1.633219	-5.047203	0.084012
C	0.235705	-5.416371	-0.115144
C	-0.174524	-4.936919	-1.431858
C	0.969087	-4.272090	-2.047424
C	2.086036	-4.339082	-1.109864
C	-0.201124	-4.945523	2.279615
C	-1.260973	-4.155796	2.898364
C	-2.379146	-4.089068	1.962110
C	-2.009761	-4.837760	0.765029
C	-0.663837	-5.367096	0.961299
C	-3.128935	-2.907639	1.846968
C	-2.790487	-1.745895	2.665389
C	-2.989954	-0.550456	1.853098
C	-3.451423	-0.970735	0.534734
C	-3.536963	-2.427270	0.530971
C	-2.110753	0.536093	1.976095
C	-0.993443	0.469220	2.913358
C	0.151554	1.137010	2.300224
C	-0.260172	1.614037	0.983696
C	-1.657980	1.243810	0.783354
C	1.446493	0.627295	2.488750
C	1.647606	-0.571064	3.299240
C	2.708379	-1.361640	2.680076
C	3.158922	-0.653698	1.485524
C	2.380655	0.573626	1.368258
C	2.627944	-2.763321	2.676752
C	1.482103	-3.429136	3.289018
C	1.143391	-4.589516	2.470987
C	2.078861	-4.641637	1.352085
C	2.996819	-3.512857	1.478609
C	0.462303	-2.669143	3.883682

H	-5.184797	1.730535	3.362850	C	-3.021582	-0.288435	-0.611788	C	0.546629	-1.212049	3.889129
H	-6.323793	-0.810697	3.191430	C	-2.102917	0.837724	-0.483382	C	-0.799393	-0.681808	3.693324
H	-4.610618	-0.823982	-4.679060	C	-1.167731	0.786279	-1.603720	C	-1.715432	-1.811124	3.566451
H	-5.848900	-2.852869	-3.211960	C	-1.505410	-0.373557	-2.423294	C	-0.935471	-3.039413	3.684444

C_{60}@**11-C(CN)$_2$**

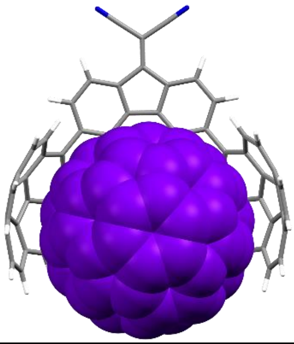

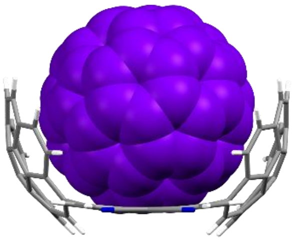

C	0.615214	4.975017	0.658418	C	-5.788638	1.463555	0.813572	C	1.499363	-0.914368	2.417650
C	-0.156818	6.831935	-0.561033	C	-6.405961	0.498508	-0.039246	C	2.654074	-1.496682	1.742179
C	-1.326632	6.065919	-0.089071	C	-5.630462	0.867697	2.101121	C	-0.656312	1.226910	0.287614
C	1.523806	4.048874	1.151969	C	-6.629572	-0.686600	0.721625	C	-1.993815	0.708745	0.029196
C	2.901295	4.200312	0.859667	C	-6.149765	-0.458139	2.045352	C	-2.360113	-0.166639	1.137087
C	3.321003	5.324348	0.113592	C	-4.947137	2.465972	0.332020	C	-1.251850	-0.183843	2.087566
C	2.410611	6.276676	-0.364663	C	-6.206763	0.473520	-1.419825	C	-0.197023	0.677828	1.561910
C	1.042382	6.097558	-0.111438	C	-4.605523	1.234227	2.973113	C	-3.483689	-1.676512	-0.469676
H	1.182321	3.173351	1.698701	C	-6.666189	-1.956320	0.144057	C	-3.382613	-3.124079	-0.621195
H	4.381879	5.450982	-0.093939	C	-5.676504	-1.486643	2.861239	C	-2.922974	-3.679302	0.648767
H	2.777778	7.122203	-0.938295	C	-4.898623	2.566979	-1.114586	C	-2.740718	-2.573062	1.582923
C	-3.110917	4.119812	0.925806	C	-4.020251	3.022592	1.322701	C	-3.091138	-1.336300	0.891593
C	-1.728136	4.028698	1.215544	C	-5.501417	1.622541	-1.946607	C	-1.560358	-5.331811	-0.598836
C	-0.853507	4.962299	0.680413	C	-6.470567	-0.805122	-2.050986	C	-0.159258	-5.702355	-0.429472
C	-2.704538	6.202844	-0.317479	C	-3.870344	2.412547	2.580775	C	0.236238	-5.361466	0.933963
C	-3.577117	5.222041	0.174922	C	-4.265509	0.236220	3.969432	C	-0.920119	-4.780974	1.608205
H	-1.352174	3.183210	1.783395	C	-6.689661	-1.963365	-1.305720	C	-2.030256	-4.761439	0.660343
H	-3.107000	7.033637	-0.888951	C	-6.393216	-3.045780	1.060165	C	0.286232	-4.972759	-2.756724
H	-4.643148	5.308678	-0.026619	C	-4.776159	-1.060312	3.915195	C	1.339180	-4.107906	-3.279330
C	5.724034	1.696378	0.776254	C	-5.921512	-2.822046	2.354917	C	2.450500	-4.127518	-2.332787
C	5.606864	1.117712	2.075837	H	-5.333253	1.698356	-3.021321	C	2.083679	-5.004533	-1.225468
C	6.386982	0.749497	-0.062622	H	-6.386406	-0.890478	-3.134714	C	0.746239	-5.526970	-1.487508
C	6.201905	-0.176859	2.043797	H	-3.104129	2.805063	3.249692	C	3.183941	-2.955978	-2.086361
C	6.681338	-0.406254	0.720104	H	-3.506728	0.467715	4.717147	C	2.835040	-1.717666	-2.778381
C	4.820189	2.637091	0.282741	H	-6.769063	-2.914833	-1.832241	C	3.013618	-0.613386	-1.841656
C	4.568938	1.444054	2.949330	H	-6.450802	-4.072675	0.698457	C	3.472160	-1.166663	-0.572202
C	6.171190	0.684174	-1.439834	H	-4.398687	-1.798235	4.623328	C	3.577477	-2.614160	-0.723441
C	5.791457	-1.211435	2.885951	H	-5.627083	-3.682108	2.956679	C	2.120502	0.468882	-1.854564
C	6.771484	-1.684755	0.167705	H	-4.282463	3.336859	-1.574892	C	1.010019	0.488475	-2.801974
C	3.868565	3.157973	1.266805	C	-0.180040	8.012040	-1.292804	C	-0.147832	1.072178	-2.129870
C	4.752378	2.706909	-1.164839	C	-1.390444	8.656940	-1.690639	C	0.249133	1.410704	-0.766850
C	3.760937	2.566466	2.537668	C	1.003467	8.694850	-1.707777	C	1.650663	1.038987	-0.596465
C	4.299685	0.455059	3.973807	N	1.958746	9.278696	-2.064107	C	-1.434731	0.569131	-2.381570
C	5.400436	1.781538	-1.983069	N	-2.369076	9.209613	-2.033005	C	-1.614613	-0.537865	-3.317063
C	6.486766	-0.593461	-2.048112	C	-1.678927	-2.592531	2.499086	C	-2.668686	-1.403502	-2.794231
C	4.883348	-0.812007	3.942008	C	-0.747035	-3.717110	2.508241	C	-3.136589	-0.833117	-1.534532
C	6.091748	-2.543076	2.401557	C	0.589712	-3.194083	2.771254	C	-2.375375	0.384337	-1.280583
C	6.771993	-1.723173	-1.281600	C	0.485411	-1.744967	2.921479	C	-2.569078	-2.796330	-2.939928
C	6.558781	-2.768108	1.105724	C	-0.916436	-1.372957	2.754895	C	-1.410544	-3.378198	-3.611037
H	4.090233	3.432601	-1.633042	C	2.463874	-4.675721	0.085392	C	-1.061663	-4.615282	-2.919371
H	2.976188	2.926987	3.202940	C	3.226270	-3.458025	0.341016	C	-2.003189	-4.798384	-1.819623
H	3.537203	0.660301	4.725411	C	2.756239	-2.887449	1.599646	C	-2.935371	-3.674360	-1.831762
H	5.217422	1.823959	-3.056972	C	1.702123	-3.752480	2.121460	C	-0.397429	-2.546248	-4.113199
H	6.390195	-0.706583	-3.128248	C	1.522264	-4.858506	1.186297	C	-0.501410	-1.098136	-3.963343
H	4.555154	-1.553956	4.670472	C	3.025521	-0.616855	0.637358	C	0.836214	-0.574986	-3.701698
H	5.844265	-3.404164	3.022824	C	2.092479	0.504683	0.622887	C	1.766507	-1.699690	-3.689301
H	6.889360	-2.681199	-1.788756	C	1.151460	0.322337	1.724323	C	1.003767	-2.918005	-3.943891
H	6.657505	-3.797510	0.760370								

C_{60}@**16**

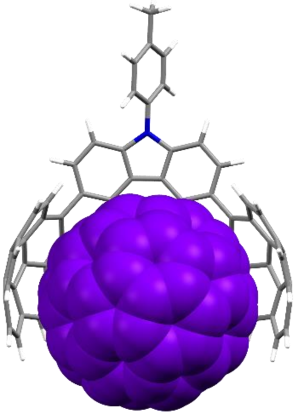

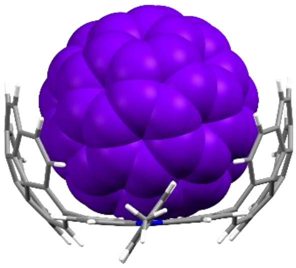

C	4.528265	0.946083	-1.225981	H	-0.443292	-6.113348	3.329361	C	-0.465580	-3.120647	0.576267
N	6.474957	0.310251	-0.183824	H	2.514794	-3.150058	-3.563549	C	0.313322	-2.260524	-0.305391
C	5.807455	-0.861723	-0.574072	H	0.117116	-3.868913	-4.816776	C	1.001032	-1.271690	0.519906
C	3.550544	1.853393	-1.642318	H	-2.516400	-6.762749	2.227433	C	0.648065	-1.522413	1.914620
C	3.686066	3.218366	-1.338178	H	-3.874963	-6.763347	-0.218089	C	-0.260422	-2.664882	1.948712
C	4.866279	3.663592	-0.684920	H	-2.055399	-4.915639	-4.474461	C	0.605776	0.425576	-1.240401
C	5.882569	2.783094	-0.300010	H	-3.715647	-6.112684	-2.560402	C	0.077824	1.782208	-1.134539
C	5.688946	1.415557	-0.542473	H	3.478681	-3.855966	1.271440	C	0.288097	2.241399	0.233395
H	2.651343	1.495778	-2.135050	H	-3.148211	6.546779	2.175500	C	0.948354	1.169192	0.973553
H	4.981084	4.727255	-0.480473	H	-4.510248	6.325881	-0.258605	C	1.144581	0.046334	0.062146
H	6.781325	3.148940	0.191298	C	1.568195	-5.710061	-0.824981	C	-1.903700	3.392250	0.144376
C	3.965925	-2.839327	-1.335663	C	0.720714	-6.320602	0.149590	C	-3.011422	3.367159	1.093997
C	3.694867	-1.493123	-1.634401	C	0.870986	-5.709080	-2.069555	C	-2.478640	2.989336	2.399239
C	4.599378	-0.500534	-1.240911	C	-0.494785	-6.697628	-0.495081	C	-1.040619	2.779357	2.254600
C	6.116738	-2.202205	-0.305592	C	-0.402122	-6.317332	-1.866400	C	-0.687790	3.028420	0.860790
C	5.183618	-3.171946	-0.683880	C	2.526503	-4.751194	-0.494803	C	-4.540363	1.681100	2.828381
H	2.748744	-1.220938	-2.096156	C	0.779403	-5.999439	1.505903	C	-4.692275	0.311054	3.306434
H	7.034895	-2.478590	0.208014	C	1.092259	-4.739026	-3.047377	C	-3.474013	-0.054575	4.023364
H	5.388995	-4.219107	-0.465777	C	-1.713297	-6.776685	0.180945	C	-2.568908	1.089427	3.988995
C	1.011350	5.846704	-0.879433	C	-1.521470	-5.997441	-2.636426	C	-3.227642	2.162021	3.249546
C	0.312451	5.743124	-2.119698	C	2.729963	-4.568501	0.930219	C	-5.885058	-0.259959	1.208953
C	0.107498	6.385920	0.085638	C	2.937188	-3.874805	-1.594061	C	-5.684518	-1.379796	0.294874
C	-1.017178	6.217294	-1.922610	C	1.902624	-5.166581	1.881389	C	-5.026709	-2.452705	1.034737
C	-1.144275	6.616990	-0.559552	C	-0.427171	-6.293268	2.254043	C	-4.821808	-1.996155	2.405708
C	2.066949	5.002939	-0.531513	C	2.234789	-3.887539	-2.810694	C	-5.352206	-0.640772	2.513295
C	0.631712	4.779949	-3.076325	C	0.001761	-4.570041	-3.989427	C	-4.051557	-3.243243	0.405920
C	0.206154	6.109661	1.449906	C	7.690574	0.353388	0.553100	C	-3.695354	-2.991193	-0.988116
C	-2.099677	5.757300	-2.674441	C	8.827194	-0.311435	0.066593	C	-2.258409	-3.200498	-1.130303
C	-2.360837	6.584239	0.123005	C	10.016652	-0.281517	0.800881	C	-1.725768	-3.579602	0.171989
C	2.560927	4.144324	-1.611585	C	10.107645	0.421195	2.016343	C	-2.833249	-3.605230	1.122485
C	2.295250	4.881308	0.896451	C	8.960620	1.090921	2.480886	C	-1.509913	-2.374721	-1.981017
C	1.856548	4.057417	-2.824822	C	7.757863	1.051712	1.769335	C	-2.168493	-1.303266	-2.723147
C	-0.438416	4.469931	-4.005435	H	8.774552	-0.834056	-0.886842	C	-1.263234	-0.158198	-2.760312
C	1.414119	5.412890	1.838611	H	10.894775	-0.799985	0.414739	C	-0.046058	-0.525525	-2.039796
C	-1.020817	6.298747	2.198794	C	11.390619	0.432119	2.814842	C	-0.198858	-1.895551	-1.559676
C	-1.739645	4.936252	-3.813929	H	9.005379	1.640270	3.421810	C	-1.773913	1.145313	-2.653985
C	-3.393004	5.928665	-2.042797	H	6.869567	1.550423	2.152206	C	-3.211862	1.356789	-2.510578
C	-2.243790	6.525405	1.566845	H	11.503045	1.366315	3.379538	C	-3.413427	2.475914	-1.594678
C	-3.517881	6.323766	-0.710211	H	12.267352	0.313988	2.165714	C	-2.100890	2.954465	-1.171543
H	3.116772	4.260985	1.250099	H	11.407528	-0.393798	3.542286	C	-1.089555	2.133706	-1.825659
H	2.209231	3.337060	-3.563464	C	-0.405577	1.750492	2.967887	C	-4.479460	2.454447	-0.682400
H	-0.252005	3.764311	-4.815711	C	-1.186409	0.886872	3.849889	C	-5.382878	1.308672	-0.645335
H	1.584374	5.188761	2.892243	C	-0.654514	-0.467870	3.742110	C	-5.737811	1.057988	0.748262
H	-1.012751	6.151131	3.279118	C	0.454120	-0.443404	2.792542	C	-5.052380	2.047593	1.573484
H	-2.524655	4.576427	-4.480017	C	0.609673	0.928437	2.314844	C	-4.274898	2.911902	0.690026
H	-4.292543	5.635040	-2.584563	C	-3.649363	-2.347331	3.094211	C	-5.189254	0.231143	-1.524816
C	-1.614072	-6.665620	1.623202	C	-2.637243	-3.171024	2.441695	C	-4.082285	0.255151	-2.476016
C	-2.884976	-6.665351	-0.664511	C	-1.323001	-2.691328	2.864022	C	-3.551115	-1.100216	-2.585511
C	-1.242179	-5.171321	-3.794202	C	-1.525737	-1.569039	3.775496	C	-4.330037	-1.962109	-1.700549
C	-2.793434	-6.291486	-2.006591	C	-2.962802	-1.357739	3.919139	C	-5.342050	-1.138813	-1.045036
H	2.040160	-4.898706	2.929390								

Key geometrical features of optimized adduct structures, as penetration depths (a and b in Figure S 172), clamping depth (c) and centroids relative angle (α)[8] were measured by generating the corresponding centroids, as depicted in Figure S 172 in Mercury.[9]

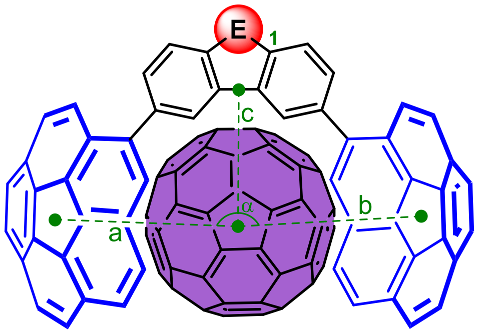

Figure S 172. General depiction of the location of key centroids, distances and angle in C_{60} inclusion complexes.

Table S 8. Summary of penetration depths (a and b), clamping depth (c), centroids relative angle (α) and E-C[1] distances of optimized supramolecular inclusion complexes

Adduct	a / Å	b / Å	c / Å	α / °	E - C¹ / Å
C_{60}@**4-S**	6.85	6.85	7.38	125.0	1.77
C_{60}@**4-SO**	6.85	6.84	7.41	125.3	1.83
C_{60}@**4-SO₂**	6.85	6.84	7.40	127.7	1.80
C_{60}@**11-CMe₂**	6.88	6.90	7.09	122.6	1.53
C_{60}@**11-CO**	6.88	6.86	7.23	126.6	1.50
C_{60}@**11-C(CN)₂**	6.88	6.87	7.23	125.9	1.48
C_{60}@**16**	6.87	6.87	7.19	127.6	1.40

Energy Decomposition Analysis[10] was calculated in the gas phase over the optimized structures with the Slater-type Orbitals (STO) triple-zeta containing two polarization functions, TZ2P, and the Perdew, Burke, Ernzerhof's PBE functional[11] in ADF suite.[12] Empirical correction to dispersion was taken into account by applying Grimme's dispersion model with Becke-Johnson damping GD3BJ.[13] The interaction energy contributions, as defined by the software, are as follows:

$$E_{int} = V_{elstat} + E_{Pauli} + E_{oi} + E_{disp} \quad \text{eq. 12}$$

Where V_{elstat} refers to the electrostatic classical-like Coulombic interactions (we will generally denote as E_{EL}), E_{Pauli} represents the Pauli exchange-type repulsions between filled orbitals (we will generally denote as E_{Rep}), E_{oi} (orbital interactions or charge transfer) cover the attractive interactions between filled and unfilled orbitals of different fragments (we will generally denote as E_{CT}) and E_{disp} corresponds to London-type weak interactions between polarizable electron clouds.

Natural Energy Decomposition Analysis (NEDA)[14] was calculated in the gas phase over the optimized structures with a more extended basis set 6-31+G(d,p) that includes diffuse functions and the Perdew, Burke, Ernzerhof's PBE0 hybrid functional as modified by Adamo.[15] in Gaussian 16 applying a tight convergence criterion. The interaction energy contributions, as defined by the software, are as follows:

$$E_{int} = EL + EX + CT \quad \text{eq. 13}$$

Where EL, EX and CT refer to E_{EL}, E_{Rep} and E_{CT}, respectively. NEDA does not explicitly account for E_{disp}, so, again, it was introduced with GD3BJ correction. Only E_{int} values are shown below.

Basis set superposition error was considered with the Boys–Bernardi functional counterpoise scheme[16] at the same level of theory used for NEDA. Interaction energy is given as:

$$E_{int}(AB) = E_{AB}^{\alpha\beta}(AB) - E_{AB}^{\alpha\beta}(A) - E_{AB}^{\alpha\beta}(B) \quad \text{eq. 14}$$

Where subscripts denote the geometry used (AB, the inclusion complex in all cases) while the superscripts refer to the basis set (αβ, the one pertaining to the supramolecular assembly in all cases). A and B correspond to host and guest entities.

With the same level of theory used for optimization, with the addition of diffuse functions to the basis set, deformation energies of the hosts (*A*) were calculated according to:

$$E_{def}(A) = E_{AB}(A) - E_{A}(A) \quad \text{eq. 15}$$

Subscripts denote the geometry used (*A* host and *AB* the inclusion complex).

Finally, electronic binding energies were then calculated according to:

$$E_{bind}(AB) = E_{int}(AB) + E_{def}(A) + E_{def}(B) \quad \text{eq. 16}$$

Where deformation energy of guest *B* (C_{60} in all cases) was negligible and assumed to be zero, providing the final equation for the binding energy:

$$E_{bind}(AB) = E_{int}(AB) + E_{def}(A) \quad \text{eq. 17}$$

Table S 9. Summary of EDA contributions, interaction energies, deformation energies of hosts and electronic binding energies of computed structures in all supramolecular adducts.[a]

Adduct	E_{EL}[b]	E_{CT}[b]	E_{Disp}[b]	E_{Rep}	E_{int}(AB)	E_{def} (A)	E_{bind} (AB)
C_{60}@**4-S**	-134.98(34.9)	-73.64(19.0)	-178.66(46.1)	244.97	-142.30	-0.69	-142.99
C_{60}@**4-SO**	-128.45(33.2)	-73-76(19.1)	-178.91(46.2)	244.51	-136.61	-1.58	-138.19
C_{60}@**4-SO$_2$**	-131.13(33.9)	-73.89(19.1)	-178.66(46.1)	244.18	-139.49	-0.67	-140.16
C_{60}@**11-CMe$_2$**	-126.82(32.8)	-71.71(18.5)	-180.04(46.5)	235.64	-142.93	3.83	-139.10
C_{60}@**11-CO**	-127.24(32.9)	-71.42(18.4)	-178.36(46.1)	237.02	-140.00	1.82	-138.18
C_{60}@**11-C(CN)$_2$**	-118.37(30.6)	-70.84(18.3)	-178.61(46.1)	235.64	-132.17	1.44	-130.74
C_{60}@**16**	-133.85(34.6)	-72.80(18.8)	-179.45(46.3)	239.16	-146.94	3.33	-143.61

[a] In kJ / mol.
[b] Values in parenthesis indicate the percentage of contribution among all the attractive (negative) energies.

Table S 10. Summary of interaction energies as provided by NEDA and Counterpoise, deformation energies of hosts and electronic binding energies of computed structures in all supramolecular adducts.[a]

Adduct	E_{int}^{NEDA}(AB)	E_{int}^{CP}(AB)	E_{def} (AB)	E_{bind}^{NEDA} (AB)	E_{bind}^{CP} (AB)
C_{60}@**4-S**	-139.67	-139.66	-0.69	-140.36	-140.35
C_{60}@**4-SO**	-139.55	-139.54	-1.58	-141.13	-141.12
C_{60}@**4-SO$_2$**	-139.08	-139.08	-0.67	-139.75	-139.74
C_{60}@**11-CMe$_2$**	-141.85	-141.84	3.83	-138.02	-138.01
C_{60}@**11-CO**	-139.32	-139.33	1.82	-137.50	-137.51
C_{60}@**11-C(CN)$_2$**	-139.60	-139.58	1.44	-138.16	-138.14
C_{60}@**16**	-142.49	-142.51	3.33	-139.16	-139.17

[a] In kJ / mol.

Regarding the geometry of the supramolecular adduct formed between host 4-S and C_{70}, three starting structures of complex C_{70}@**4-S**, whose difference relies on the relative cartesian orientations of C_{70} (

Figure S **173**), were optimized at the same level of theory used for other optimizations. This was done for the two most stable conformers of compound **4-S** to provide an assortment of six inclusion complexes whose relative Gibbs free energy was not higher than 1.5 kcal·mol^{-1}. Only the most stable one (lowest in energy) is shown.

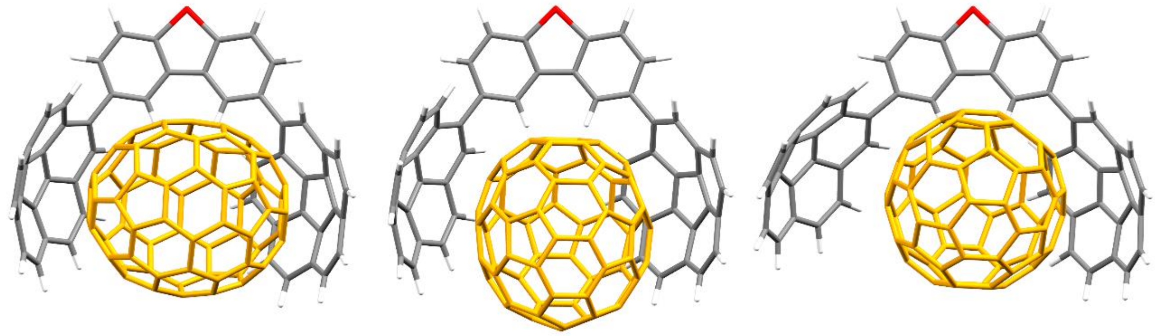

Figure S 173. Starting geometry of C_{70}@**4-S** with different orientation of C_{70}.

C_{70}@**4-S** G = -5057.679205 a.u

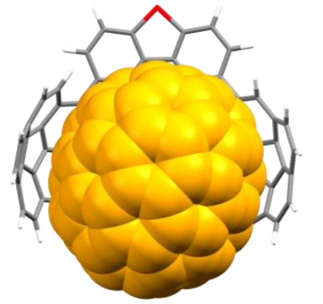

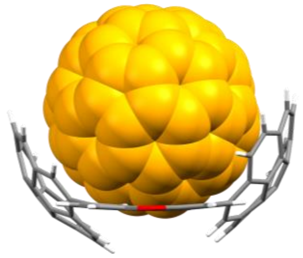

C	-1.616184	5.512325	-0.381665	C	4.912146	2.542002	2.289425	C	0.139224	-5.365909	-2.056153
S	-1.013986	7.651106	-1.841912	C	6.054392	2.030962	0.353830	C	0.464757	-4.526175	-3.162931
C	0.297345	6.779957	-1.031393	C	5.670272	1.359944	2.527177	C	-0.620689	-3.821256	-3.763876
C	-2.417216	4.481760	0.134278	C	6.377166	1.043153	1.329786	C	-2.136666	-4.349299	-1.845724
C	-3.785016	4.415577	-0.178239	C	4.234008	3.704817	0.208381	C	-1.147323	-5.278866	-1.404761
C	-4.349658	5.435352	-0.985725	C	3.716721	2.815159	2.952858	C	-1.907742	-3.734038	-3.112813
C	-3.576651	6.478408	-1.500989	C	6.072306	1.768164	-1.015766	C	3.174487	-2.569529	1.993436
C	-2.206088	6.502451	-1.211846	C	5.286277	0.391346	3.454492	C	3.837175	-2.439820	0.703198
H	-1.957485	3.694007	0.721909	C	6.738781	-0.261296	0.993247	C	2.473008	-3.742919	2.311799
H	-5.414783	5.397269	-1.209448	C	3.096306	4.220302	0.975527	C	3.789228	-3.488474	-0.218325
H	-4.031141	7.244233	-2.127311	C	4.390152	3.591053	-1.229144	C	3.590552	-3.196832	-1.629063
C	2.121633	5.121797	0.317329	C	2.862232	3.771097	2.285794	C	3.536130	-1.855245	-2.113183
C	0.743451	4.882413	0.427718	C	3.424164	1.927831	4.062165	C	2.830299	-1.651616	-3.336532
C	-0.177599	5.681935	-0.264034	C	5.264301	2.669853	-1.810690	C	1.812944	-3.934214	-3.227199
C	1.667749	7.056111	-1.127997	C	6.650658	0.488194	-1.378182	C	2.737443	-4.224856	-2.179415
C	2.566962	6.215678	-0.467244	C	4.174596	0.775985	4.303180	C	1.977161	-2.679817	-3.887091
H	0.393246	4.033946	1.008967	C	5.858905	-0.920578	3.226989	C	0.636432	-2.393579	3.285025
H	2.029197	7.898445	-1.715416	C	6.969934	-0.477147	-0.421891	C	1.367447	-1.179769	2.949576
H	3.635727	6.410492	-0.541677	C	6.550269	-1.231566	2.054251	C	1.174783	-3.653594	2.971276
C	-5.436473	1.637004	1.814308	H	3.724600	4.153905	-1.881585	C	2.607080	-1.267750	2.311526
C	-6.158248	0.979127	0.777142	H	1.957359	4.116653	2.786463	C	2.938922	-0.344009	1.239775
C	-5.257002	0.703864	2.879571	H	2.532829	2.104001	4.665166	C	2.058529	0.714221	0.869661
C	-6.410307	-0.360679	1.190250	H	5.247630	2.555430	-2.894915	C	2.180872	1.198732	-0.465563
C	-5.856855	-0.530065	2.493055	H	6.741990	0.225414	-2.432630	C	3.590280	-0.751156	-1.138735
C	-4.566480	2.703526	1.588404	H	3.841110	0.093093	5.084813	C	3.694427	-1.067931	0.247178
C	-6.045339	1.349951	-0.561165	H	5.651536	-1.723240	3.935039	C	2.938694	0.474626	-1.458415
C	-4.194668	0.777319	3.778228	H	7.297026	-1.459664	-0.764075	C	0.747431	0.806406	1.537991
C	-6.545685	-1.413262	0.285890	H	6.856590	-2.265111	1.891139	C	0.413046	-0.165620	2.529184
C	-5.418691	-1.763245	2.972656	C	-1.046641	-4.426966	2.194736	C	-0.346110	1.372217	0.815566
C	-4.608779	3.248160	0.228547	C	-1.597098	-3.117022	2.511989	C	1.152492	1.555736	-2.564211
C	-3.624228	2.932644	2.671151	C	0.315554	-4.691331	2.413538	C	1.075670	1.855723	-1.142308
C	-5.331607	2.584257	-0.781786	C	-0.773885	-2.120857	3.047771	C	-0.168712	1.933575	-0.514198
C	-6.403685	0.308445	-1.507546	C	-0.909001	-0.748643	2.587421	C	-0.023948	1.336455	-3.302187
C	-3.440027	2.012124	3.704588	C	-1.925252	-0.372487	1.658781	C	1.038486	-0.586628	-4.448142
C	-3.878380	-0.472296	4.442403	C	-1.669461	0.792121	0.877678	C	2.251105	-0.361376	-3.674612
C	-6.639684	-1.006213	-1.103411	C	0.454948	-5.857925	0.231273	C	2.306123	0.686119	-2.750264
C	-6.291052	-2.725884	0.848242	C	-0.955005	-5.573694	0.005501	C	-0.081686	0.242254	-4.264851
C	-4.461788	-1.681484	4.059476	C	1.082956	-5.422792	1.410611	C	-1.573733	-1.735093	-4.325298
C	-5.759942	-2.892757	2.128622	C	-1.689790	-4.872607	0.966151	C	-0.409283	-2.589383	-4.505084
H	-2.982479	3.811013	2.648582	C	-2.639361	-3.855395	0.546586	C	0.869755	-2.027193	-4.565423
H	-5.217687	2.953946	-1.800992	C	-2.912539	-3.611077	-0.833098	C	-1.415826	-0.344448	-4.201926
H	-6.384668	0.529234	-2.575077	C	-3.401091	-2.311651	-1.160563	C	-3.076542	-0.303935	-2.362867

H	-2.643182	2.197379	4.425845	C	-2.789220	-1.420022	1.093945	C	-3.230229	-1.747129	-2.489413
H	-3.086756	-0.492370	5.191812	C	-2.576581	-2.771063	1.496875	C	-2.493782	-2.447386	-3.450256
H	-6.793417	-1.765102	-1.871102	C	-3.339277	-1.228063	-0.205978	C	-2.184661	0.387228	-3.200479
H	-6.417632	-3.608192	0.220264	C	3.066375	-4.710112	0.108362	C	-1.390655	1.723748	-1.274429
H	-4.101089	-2.600497	4.521766	C	2.407713	-5.153593	-1.110823	C	-2.310927	0.999732	-0.408678
H	-5.492693	-3.898874	2.452111	C	2.416392	-4.837313	1.347881	C	-3.134181	0.005682	-0.941418
C	5.154002	2.964611	0.948818	C	1.128416	-5.715707	-1.050282	C	-1.323847	1.426550	-2.644961

A second host **4-S** was added to the three most stable optimized structure of adduct C_{70}@**4-S** at the non-occupied region of the outer surface of fullerene and resulting 2:1 assembly geometry, namely C_{70}@(**4-S**)$_2$, was freely minimized in gas phase with the general level of theory described above. Solvent effects were taken into account by carrying out single point calculations on the optimized structures at the general level of theory as described above. Only the most stable one (lowest in energy) is shown.

C_{70}@**(4-S)$_2$** E = -7451.6237119 a. u

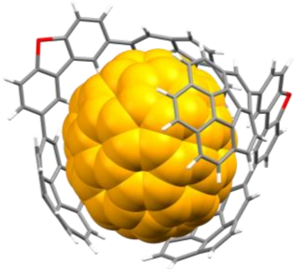

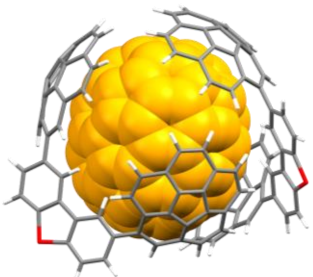

C	-4.649215	-4.955762	2.208447	H	-5.357304	4.381854	-4.292981	H	-2.299577	7.308367	-0.584345
S	-6.260331	-6.760430	1.114522	H	-4.770325	5.947327	-2.046546	C	-1.804797	6.331551	2.123521
C	-6.636798	-5.042129	0.895073	C	0.620680	3.387236	0.802463	C	5.933361	-2.293351	-3.653494
C	-3.518479	-4.461953	2.870858	C	-0.044928	2.522157	1.767931	C	-0.962328	6.961507	1.126498
C	-2.546507	-5.333233	3.389115	C	-0.128492	4.061477	-0.173106	H	2.881235	-0.329641	-7.380593
C	-2.751329	-6.729360	3.247893	C	-1.435529	2.374730	1.733344	C	3.624164	-0.728330	-6.689155
C	-3.870748	-7.247435	2.589231	C	-2.023125	1.058189	1.932947	C	6.071016	1.174613	2.362848
C	-4.813892	-6.356616	2.057450	C	-1.218922	-0.076703	2.255944	C	5.406127	3.383079	3.958425
H	-3.362367	-3.390433	2.917985	C	-1.755372	-1.352289	1.908140	C	-1.273311	5.643019	3.213576
H	-2.014420	-7.413589	3.666653	C	1.568697	3.506017	-1.889619	C	6.344877	-0.159707	-2.669543
H	-3.998403	-8.324015	2.487161	C	2.336498	2.797582	-0.877697	C	6.249528	-1.599171	-2.486106
C	-6.882466	-2.239854	0.712653	C	0.354836	4.123035	-1.546934	C	4.123997	4.430098	2.069646
C	-5.849822	-2.811026	1.471902	C	1.870198	2.736564	0.439075	H	4.275781	1.239189	-6.149168
C	-5.701955	-4.201189	1.554061	C	1.983125	1.497950	1.190312	H	0.358686	3.887296	4.873080
C	-7.692950	-4.494324	0.153178	C	2.633396	0.352292	0.641135	C	4.204112	-5.411605	-2.638560
C	-7.800351	-3.103626	0.060860	C	2.245209	-0.912493	1.169829	H	-2.882757	6.297295	1.965928
H	-5.121645	-2.167156	1.956077	C	0.243971	0.080807	2.291881	C	4.391397	4.942782	0.789940
H	-8.416780	-5.135694	-0.347188	C	0.799893	1.363837	2.009288	H	5.900960	1.565809	-3.854112
H	-8.619959	-2.668709	-0.509078	C	1.060320	-1.047006	1.986694	H	4.921719	2.147270	0.808025
C	-0.044480	-3.178606	5.256514	C	-0.680373	3.278553	-3.633934	C	6.200857	-2.406402	-1.262799
C	1.177497	-3.726471	4.772622	C	0.576425	2.630324	-3.978122	C	1.417419	7.134960	0.473349
C	0.213988	-1.836185	5.671622	C	-0.792774	4.008770	-2.437148	C	5.489783	-1.642817	-4.844508
C	2.185917	-2.724053	4.873699	C	1.679868	2.743664	-3.124456	C	4.424423	0.171772	-5.982848
C	1.588794	-1.554722	5.426265	C	2.523585	1.585091	-2.878065	C	2.248853	6.854224	-1.753914
C	-1.302199	-3.605112	4.831219	C	2.300160	0.346013	-3.547857	C	2.610274	-4.367626	-5.977704
C	1.215934	-4.741616	3.818143	C	2.777885	-0.825697	-2.892003	C	-0.256020	7.405247	-1.217912
C	-0.766911	-0.847014	5.692749	C	3.119509	0.415882	-0.748985	H	5.144462	4.248667	4.565168
C	3.287399	-2.672956	4.021476	C	2.930065	1.620470	-1.489618	C	0.392629	7.007317	1.456628
C	2.058860	-0.268369	5.160686	C	3.183043	-0.790527	-1.507778	C	7.251054	-0.883740	2.621259
C	-1.290462	-4.789435	3.966445	C	-3.016356	3.006268	-2.014536	C	0.937221	6.304307	2.571720
C	-2.369792	-2.663243	5.131187	C	-2.892424	2.244633	-3.248712	C	-1.259817	7.277646	-0.256573
C	-0.068341	-5.321703	3.506373	C	-1.989517	3.874213	-1.612191	C	2.222429	4.212020	3.867855
C	2.469247	-4.830852	3.088253	C	-1.749418	2.377660	-4.042342	C	2.958909	4.895263	2.823413
C	-2.114908	-1.348978	5.522986	C	-1.155351	1.197527	-4.648959	H	4.139784	5.871714	-1.968380
C	-0.258725	0.508439	5.640577	C	-1.762306	-0.089308	-4.530867	C	6.199612	2.373782	4.506049
C	3.453663	-3.845457	3.183814	C	-0.893379	-1.213918	-4.664107	H	-1.954254	5.093705	3.864723
C	3.902346	-1.366280	3.905354	C	1.140330	0.226120	-4.447475	C	3.562216	-4.672275	-4.927593
C	1.086205	0.781400	5.387270	C	0.281219	1.353538	-4.608588	H	5.539804	-4.219670	-0.339024
C	3.319904	-0.219126	4.448045	C	0.542475	-1.057757	-4.623078	C	0.889495	4.510173	4.152421
H	-3.406141	-2.959027	4.984324	C	-2.217477	3.080502	0.725276	H	5.219765	4.504287	0.232760
H	-0.121259	-6.109400	2.754433	C	-3.275747	2.178144	0.296510	C	5.293807	2.215574	1.827888
H	2.601721	-5.627662	2.355586	C	-1.578243	3.907210	-0.212736	H	5.496673	0.010640	-0.171078
H	-2.960357	-0.668010	5.630771	C	-3.670975	2.143972	-1.042650	C	6.579991	-1.844417	0.059300
H	-0.958737	1.343673	5.693043	C	-3.957256	0.869864	-1.677953	C	5.679097	-3.714789	-1.295350
H	4.320794	-3.905858	2.524630	C	-3.935420	-0.350935	-0.944649	C	4.680952	-2.560902	-5.574346
H	4.782179	-1.253004	3.278018	C	-3.701444	-1.540414	-1.698067	C	2.299700	5.995677	2.277255
H	1.386041	1.818305	5.251255	C	-2.971419	-0.227327	-3.697750	C	1.145561	7.229654	-0.891936
H	3.777265	0.744231	4.226335	C	-3.479456	0.931315	-3.039907	H	8.288835	-2.769722	2.904214
C	-7.423886	1.229113	-0.700629	C	-3.224687	-1.480283	-3.060504	C	5.332639	-0.262134	-4.940282
C	-7.119823	2.031162	0.440031	C	-3.517187	-0.312778	0.466380	C	4.953887	3.328047	2.615236
C	-7.257309	2.044712	-1.860339	C	-3.160254	0.940057	1.046216	S	7.492659	-0.124904	4.199224
C	-6.768579	3.335819	-0.011233	C	-2.888702	-1.466598	1.019917	C	3.396593	6.235449	-1.258136
C	-6.857875	3.347054	-1.434257	C	-2.322940	-3.425847	-2.064691	C	3.557114	5.935237	0.151708
C	-7.258069	-0.156272	-0.719472	C	-3.143069	-2.733405	-1.082068	C	5.165987	-4.335047	-2.493488

C	-6.623917	1.486243	1.622819	C	-2.738561	-2.692910	0.253391	C	5.410696	-3.618521	-3.662367
C	-6.898084	1.522543	-3.102870	C	-1.126946	-4.052184	-1.669939	H	2.135521	6.947788	-2.834499
C	-5.900404	4.168609	0.696301	C	0.018377	-3.157778	-3.677793	C	6.533133	1.274231	3.704357
C	-6.088539	4.197821	-2.227473	C	-1.220381	-2.503644	-4.075687	H	3.985981	-6.049696	-1.781403
C	-6.984027	-0.766036	0.585234	C	-2.367058	-2.634265	-3.285790	H	-0.548308	7.530899	-2.260967
C	-7.079472	-0.720902	-2.044063	C	0.068723	-3.915864	-2.494220	C	6.144961	-0.573861	0.472054
C	-6.681983	0.045655	1.693065	C	2.264801	-2.920256	-1.935074	C	3.657989	-2.154195	-6.429666
C	-5.906109	2.421102	2.467440	C	2.208169	-2.135829	-3.158712	H	6.548447	2.441358	5.535101
C	-6.911093	0.076916	-3.176405	C	1.104632	-2.248085	-4.007513	H	7.714674	-3.595982	0.634433
C	-6.294332	2.477394	-4.010936	C	1.218478	-3.794798	-1.604139	H	2.656911	-6.329614	-3.799713
C	-5.560670	3.698476	2.024961	C	1.320364	-3.044094	0.753242	C	2.655497	-3.168473	-6.692021
C	-5.268339	5.198328	-0.102745	C	2.405783	-2.138206	0.404980	H	2.689207	3.369926	4.375117
C	-5.909387	3.751890	-3.594802	C	2.866896	-2.078098	-0.912795	C	0.155876	5.519669	3.419056
C	-5.359542	5.213872	-1.494993	C	0.735990	-3.860567	-0.228658	H	6.709772	0.463823	-1.856542
H	-6.952080	-1.796498	-2.149564	C	-1.512293	-3.351090	0.672469	C	7.693765	-2.154507	2.231235
H	-6.409979	-0.449555	2.625225	C	-0.900135	-2.506753	1.689081	C	2.593143	6.508692	0.980009
H	-5.525894	2.083342	3.432265	C	0.486632	-2.354938	1.728126	H	1.766437	-5.038153	-6.143258
H	-6.664086	-0.410948	-4.119883	C	-0.715054	-4.019043	-0.269886	H	1.845726	-2.949312	-7.388711
H	-6.028161	2.157442	-5.018812	C	7.358879	-2.618863	0.957904	C	5.897290	0.475647	-3.828682
H	-4.921849	4.313427	2.660355	C	4.632807	-3.783062	-4.843872	C	3.440725	-5.571367	-3.796283
H	-4.617118	5.926136	0.381802	C	6.474871	-0.078885	1.743030				

Deformation energy of the host (*A*) was here calculated (with the same level of theory as above) according to:

$$E_{def}(A) = E_{A_2B}(A) - 2E_A(A) \qquad \text{eq. 18}$$

Electronic binding energy was calculated by using already reported eq. 17 denoting the inclusion complex as A_2B.

Alternatively, electronic binding energies for assemblies C_{70}@**4-S** and C_{70}@(**4-S**)$_2$ were estimated by a different method[17] as follows:

$$\Delta E_{bind}(AB) = E_{AB}(AB) - E_A(A) - E_B(B) \qquad \text{eq. 19}$$

$$\Delta E_{bind}(A_2B) = E_{A_2B}(A_2B) - 2E_A(A) - E_B(B) \qquad \text{eq. 20}$$

Consequently, a difference in binding energy between the ternary and binary complex can be calculated according to:

$$\Delta\Delta E_{bind}(A_2B) = \Delta E_{bind}(A_2B) - \Delta E_{bind}(AB) \qquad \text{eq. 21}$$

Table S 11. Summary of Counterpoise interaction energies, deformation energies of hosts, electronic binding energies obtained by both above-described methods, and binding energy differences between ternary and binary supramolecular adducts.

Adduct	E_{int} / kJ ·mol^{-1} [a]	E_{def}/ kJ ·mol^{-1}	ΔE_{bind}/ kJ ·mol^{-1} [c]	$\Delta\Delta E_{bind}$/ kJ ·mol^{-1} [d]	ΔE_{bind}/ kJ ·mol^{-1} [e]	$\Delta\Delta E_{bind}$/ kJ ·mol^{-1} [f]
C_{70}@**4-S**	-145.77	10.33[b]	-135.44	-	-199.49	-
C_{70}@**(4-S)$_2$**	-278.82	-29.14	-307.97	-37.09	-426.51	-37.03

[a] Obtained by the Counterpoise method.
[b] Calculated with eq. 15.
[c] Calculated with eq. 17.
[d] Calculated with data of ΔE_{bind} in the fourth column.
[e] Calculated with eq. 19 and 20 where appropriate.
[f] Calculated with data of ΔE_{bind} in the sixth column.

Note: interaction energies in these ternary assemblies, as obtained by Counterpoise method, were calculated in two ways. On the one hand, two fragments were only considered where the host is the dimer (**4-S**)$_2$. On the other hand, three fragments were set treating each host **4-S** independently. The first option provided the E_{int} corresponding to the host-C_{70} interaction (and is reflected in Table S 11), whereas the second option included all the interactions (with a value of - 312.52 kJ·mol^{-1}). By subtracting the latter from the former it is possible to estimate the E_{int} between the two units of host **4-S**, which resulted in a value of -33.72 kJ·mol^{-1}.

Non-covalent interactions were obtained according to Yang and collaborators' scheme with the help of the NCIPlot package[18] by locating critical points defined as regions of decreased reduced density gradient where electronic density values are low. Calculations were carried out with promolecular densities, gradient isosurfaces were plotted with an isovalue of 0.3 a.u. and colored on a RGB scale according to values of the sign of λ_2 (second eigenvalue of the electron-density Hessian). Red indicates repulsion, green means weak attraction, and blue represents strong attraction. Graphics were visualized in Chimera[19] with the help of Tangram NCIPlot GUI built by Insilichem Group.[20]

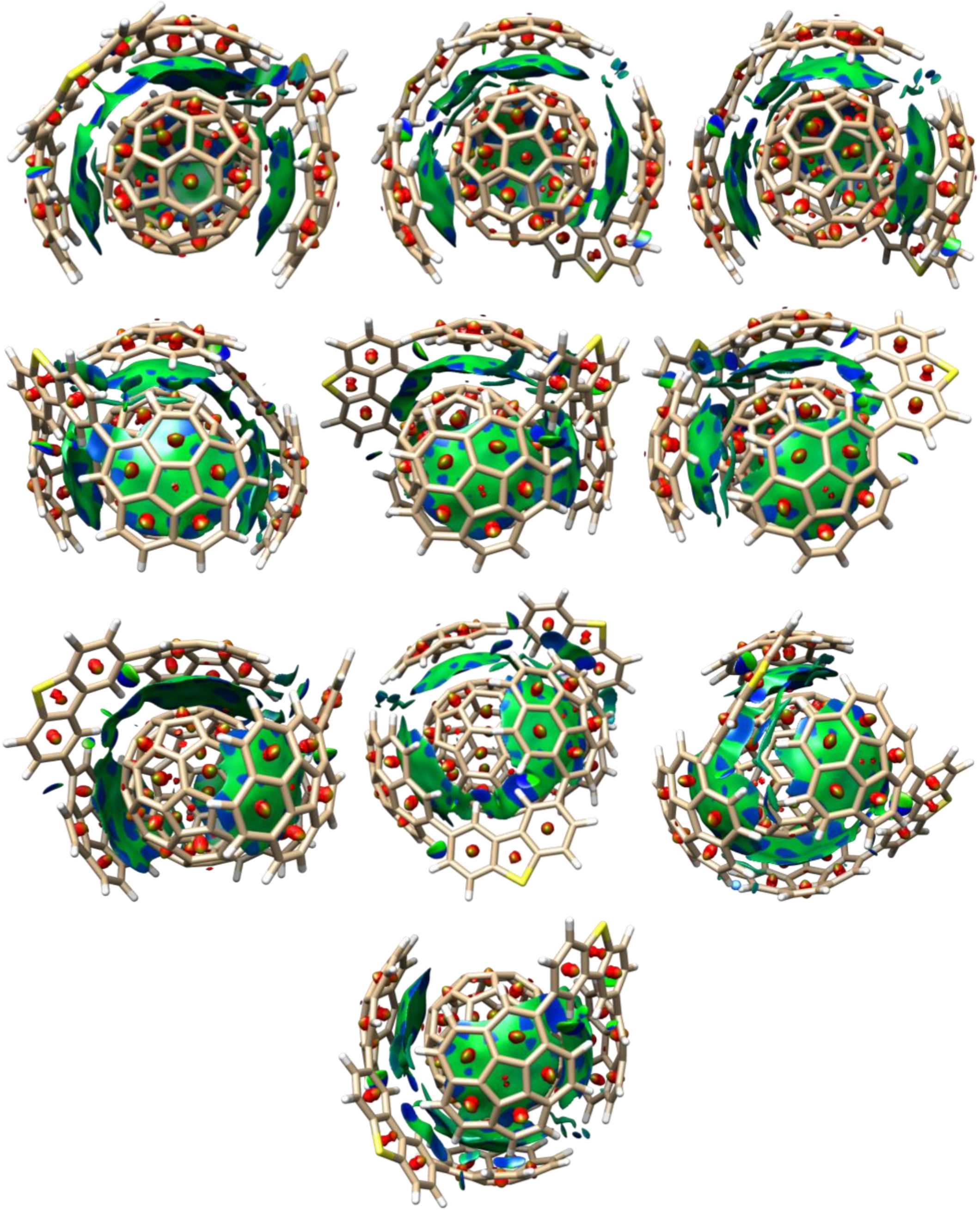

Figure S 174. Different orientations of the non-covalent interactions represented as gradient isosurfaces (with an isovalue of 0.3 a.u.) for supramolecular assembly C_{70}@(**4-S**)$_2$.

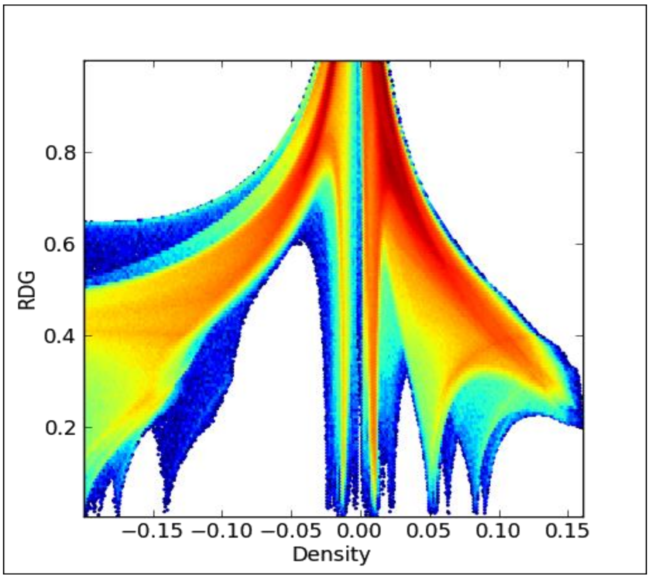

Figure S 175. Plot of the reduced density gradient versus the electron density multiplied by the sign of the second Hessian eigenvalue (λ_2) of supramolecular assembly C_{70}@(**4-S**)$_2$.

References

1. Barat, V.; Budanovic, M.; Halilovic, D.; Huh, J.; Webster, R. D.; Mahadevegowda, S. H.; Stuparu, M. C., *Chem. Commun.* **2019,** *55*, 3113-3116.
2. Wu, W.; Tang, J., *Acta Crystallogr. E* **2011,** *67*, o1919.
3. (a) Hansch, C.; Leo, A.; Taft, R. W., *Chem. Rev.* **1991,** *91*, 165-195; (b) Lewis, M.; Bagwill, C.; Hardebeck, L. K. E.; Wireduaah, S., *Comput. Struct. Biotechnol. J.* **2012,** *1*, e201204004.
4. (a) Ditchfield, R.; Hehre, W. J.; Pople, J. A., *J. Chem. Phys.* **1971,** *54*, 724-728; (b) Hehre, W. J.; Ditchfield, R.; Pople, J. A., *J. Chem. Phys.* **1972,** *56*, 2257-2261; (c) Francl, M. M.; Pietro, W. J.; Hehre, W. J.; Binkley, J. S.; Gordon, M. S.; DeFrees, D. J.; Pople, J. A., *J. Chem. Phys.* **1982,** *77*, 3654-3665; (d) Frisch, M. J.; Pople, J. A.; Binkley, J. S., *J. Chem. Phys.* **1984,** *80*, 3265-3269.
5. Reed, A. E.; Weinhold, F., *J. Chem. Phys.* **1985,** *83*, 1736-1740.
6. (a) Glendening, E. D.; Landis, C. R.; Weinhold, F., *J. Comput. Chem.* **2019,** *40*, 2234-2241; (b) NBO 7.0. E. D. Glendening, J, K. Badenhoop, A. E. Reed, J. E. Carpenter, J. A. Bohmann, C. M. Morales, P. Karafiloglou, C. R. Landis, and F. Weinhold, Theoretical Chemistry Institute, University of Wisconsin, Madison (2018).
7. GaussView, Version 6.1.1, Roy Dennington, Todd Keith, and John Millam, Semichem Inc., Shawnee Mission, KS, 2019.
8. Liu, Y.-Z.; Yuan, K.; Scheiner, S., *Diamond Relat. Mater.* **2021,** *114*, 108293.
9. Macrae, C. F.; Sovago, I.; Cottrell, S. J.; Galek, P. T. A.; McCabe, P.; Pidcock, E.; Platings, M.; Shields, G. P.; Stevens, J. S.; Towler, M.; Wood, P. A., *J. Appl. Crystallogr.* **2020,** *53*, 226-235.
10. (a) Su, P.; Li, H., *J. Chem. Phys.* **2009,** *131*, 014102; (b) Zhao, L.; von Hopffgarten, M.; Andrada, D. M.; Frenking, G., *WIREs Comput. Mol. Sci.* **2018,** *8*, e1345.
11. (a) Perdew, J. P.; Burke, K.; Ernzerhof, M., *Phys. Rev. Lett.* **1996,** *77*, 3865-3868; (b) Perdew, J. P.; Burke, K.; Ernzerhof, M., *Phys. Rev. Lett.* **1997,** *78*, 1396-1396.
12. (a) ADF 2022.1, SCM, Theoretical Chemistry, Vrije Universiteit, Amsterdam, The Netherlands, http://www.scm.com. Optionally, you may add the following list of authors and contributors: E.J. Baerends, T. Ziegler, A.J. Atkins, J. Autschbach, O. Baseggio, D. Bashford, A. Bérces, F.M. Bickelhaupt, C. Bo, P.M. Boerrigter, C. Cappelli, L. Cavallo, C. Daul, D.P. Chong, D.V. Chulhai, L. Deng, R.M. Dickson, J.M. Dieterich, F. Egidi, D.E. Ellis, M. van Faassen, L. Fan, T.H. Fischer, A. Förster, C. Fonseca Guerra, M. Franchini, A. Ghysels, A. Giammona, S.J.A. van Gisbergen, A. Goez, A.W. Götz, J.A. Groeneveld, O.V. Gritsenko, M. Grüning, S. Gusarov, F.E. Harris, P. van den Hoek, Z. Hu, C.R. Jacob, H. Jacobsen, L. Jensen, L. Joubert, J.W. Kaminski, G. van Kessel, C. König, F. Kootstra, A. Kovalenko, M.V. Krykunov, P. Lafiosca, E. van Lenthe, D.A. McCormack, M. Medves, A. Michalak, M. Mitoraj, S.M. Morton, J. Neugebauer, V.P. Nicu, L. Noodleman, V.P. Osinga, S. Patchkovskii, M. Pavanello, C.A. Peeples, P.H.T. Philipsen, D. Post, C.C. Pye, H. Ramanantoanina, P. Ramos, W. Ravenek, M. Reimann, J.I. Rodríguez, P. Ros, R. Rüger, P.R.T. Schipper, D. Schlüns, H. van Schoot, G. Schreckenbach, J.S. Seldenthuis, M. Seth, J.G. Snijders, M. Solà, M. Stener, M. Swart, D. Swerhone, V. Tognetti, G. te Velde, P. Vernooijs, L. Versluis, L. Visscher, O. Visser, F. Wang, T.A. Wesolowski, E.M. van Wezenbeek, G. Wiesenekker, S.K. Wolff, T.K. Woo, A.L. Yakovlev; (b) Rüdiger, E. C.; Porz, M.; Schaffroth, M.; Rominger, F.; Bunz, U. H. F., *Chem. Eur. J.* **2014,** *20*, 12725-12728.
13. (a) Grimme, S.; Antony, J.; Ehrlich, S.; Krieg, H., *J. Chem. Phys.* **2010,** *132*, 154104; (b) Grimme, S.; Ehrlich, S.; Goerigk, L., *J. Comput. Chem.* **2011,** *32*, 1456-1465.
14. (a) Glendening, E. D.; Streitwieser, A., *J. Chem. Phys.* **1994,** *100*, 2900-2909; (b) Glendening, E. D., *J. Am. Chem. Soc.* **1996,** *118*, 2473-2482; (c) Schenter, G. K.; Glendening, E. D., *J. Phys. Chem.* **1996,** *100*, 17152-17156.
15. (a) Adamo, C.; Barone, V., *J. Chem. Phys.* **1999,** *110*, 6158-6170; (b) Ernzerhof, M.; Scuseria, G. E., *J. Chem. Phys.* **1999,** *110*, 5029-5036.
16. (a) Boys, S. F.; Bernardi, F., *Mol. Phys.* **1970,** *19*, 553-566; (b) van Duijneveldt, F. B.; van Duijneveldt-van de Rijdt, J. G. C. M.; van Lenthe, J. H., *Chem. Rev.* **1994,** *94*, 1873-1885; (c) Kirschner, K. N.; Sorensen, J. B.; Bowen, J. P., *J. Chem. Educ.* **2007,** *84*, 1225.
17. Sure, R.; Grimme, S., *J. Chem. Theory Comput.* **2015,** *11*, 3785-3801.
18. (a) Johnson, E. R.; Keinan, S.; Mori-Sánchez, P.; Contreras-García, J.; Cohen, A. J.; Yang, W., *J. Am. Chem. Soc.* **2010,** *132*, 6498-6506; (b) Contreras-García, J.; Johnson, E. R.; Keinan, S.; Chaudret, R.; Piquemal, J.-P.; Beratan, D. N.; Yang, W., *J. Chem. Theory Comput.* **2011,** *7*, 625-632.
19. Pettersen, E. F.; Goddard, T. D.; Huang, C. C.; Couch, G. S.; Greenblatt, D. M.; Meng, E. C.; Ferrin, T. E., *J. Comput. Chem.* **2004,** *25*, 1605-1612.
20. Rodríguez-Guerra Pedregal, J. Development and Application of a Computational Platform for Complex Molecular Design, Universitat Autònoma de Barcelona, 2018. ISBN 9788449082382. https://ddd.uab.cat/record/201498. (https://github.com/insilichem/tangram_nciplot).
